# Genetic and Proteomic characterization of Bile Salt Export Pump (BSEP) in Snake Liver

**DOI:** 10.1038/srep43556

**Published:** 2017-04-03

**Authors:** Xinle Tan, Fei Gao, Hexiu Su, Yajun Gong, Jie Zhang, Mitchell A. Sullivan, Jiachun Chen

**Affiliations:** 1 Tongji School of Pharmacy, Huazhong University of Science and Technology, Wuhan, Hubei 430030, China; 2 The University of Queensland, School of Chemistry & Molecular Bioscience, Brisbane, QLD 4072, Australia; 3 Department of Hematology, Affiliated Union Hospital, Tongji Medical College, Huazhong University of Science and Technology, Wuhan, Hubei 430030, China; 4 Program in Genetics and Genome Biology, The Hospital for Sick Children, Toronto, ON, M5G 1X8, Canada

## Abstract

Snake gallbladder, a traditional Chinese medicine, has been believed in various Asian countries to improve visual acuity and alleviate rheumatism. Bile acids, a major component of the gallbladder, are toxic to the liver and kidney in humans and animals due to its detergent effects, while also exhibiting therapeutic effects due to an increase in the gallbladder contractions of muscle strips in patients with cholesterol gallstones. Secretion of bile acids in human and mammals depends on the bile salt export pump (BSEP), a liver-specific adenosine triphosphate (ATP)-binding cassette transporter encoded by *ABCB11*. However, the presence of BSEP in snakes has not been thoroughly explored. Here we confirm the existence of BSEP and its coding DNA sequence in snakes on both the proteomic and genetic level. This work provides information on the snake *ABCB11* sequence and helps further potential genetic manipulation to affect bile salt metabolism. Our study provides the foundation for research on bile acid production from snakes by using modern genetic and proteomic methodologies.

Snake gallbladder has been widely used in traditional Chinese medicine with multiple pharmacological effects, such as relieving coughs and asthma, as well as improving human immunity and visual acuity^1^.However, the quality of snake gallbladder has been hard to control and assess due to its diverse sources and undefined international standard. Hence, this study aims at providing new research to assess it from the aspect of bile salt secretion regulation.

As the main component of the gallbladder, bile has two major functions: facilitating intestinal digestion through absorbing dietary fat and fat-soluble vitamins, as well as eliminating harmful substances.

Bile enterohepatic circulation plays a primary role in maintaining bile function. Cholecystokinin has been shown to be secreted from the duodenum after a meal to stimulate gallbladder contraction and the release of bile acids into the intestinal tract. Later, bile acids facilitate the solubilization of fatty acids and monoacylglycerols and help to digest and absorb dietary lipids and fat-soluble vitamins. It was shown bile acids are subsequently reabsorbed and transported back to the liver^2^. There are various bile salt transporters involved in this process, including but not limited to NTCP (sodium-taurocholate cotransporting polypeptide) and OATPs (Na^+^-independent organic anion transporting polypeptides).

Bile salt export pump (BSEP) is the major transporter responsible for bile salt secretion^3^. It provides the driving force of bile flow from hepatocytes to the gallbladder, between which high concentration gradients have to be overcome. BSEP, as an ATP-dependent export pump, was discovered to transport bile salt across the gradient at the mammalian canalicular membrane^4^.

The structure and function of BSEP has been well researched in humans^5^, rodents^6^, mammals^7^ and Raja echinacea^8^. However no study has demonstrated the existence of snake BSEP. Considering that BSEP has not been identified in snakes and it may be meaningful for snake gallbladder quality control (if it exists in snakes), this research aims at exploring the existence of BSEP in snakes from both genetic and proteomic aspects.

## Results

### Sequence analysis

**Figure 1:**
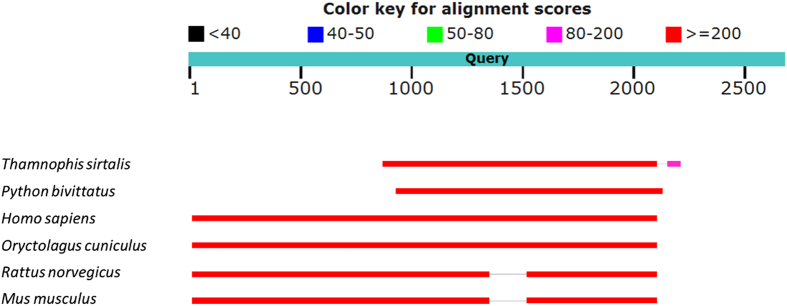
Sequence alignment results from snakes (*Thamnophis sirtalisand Python bivittatus*), human (*Homo sapiens*), rabbit (*Oryctolagus cuniculus*), rat (*Rattus norvegicus*) and mouse (*Mus musculus*).

The first sequence of the snake species *Elaphe carinat* was obtained using designed primers based on the conserved region of predicted sequences from 3 other snakes (see Fig. 1. in Supplementary Information) as described in the material and methods section. In the following PCR steps, there were 4 other sequences obtained (see Supplementary Information “3. Sequence information of later fragments”). In total, 2165 base pairs were identified consecutively. This is a smaller sequence than those predicted in *Ophiophagus Hannah* (3078 bp), *Thamnophis sirtalis* (2418 bp) and *Python bivittatus* (2238 bp).

RACE (rapid amplification of cDNA ends) was applied to obtain the remaining sequence. Primer information is listed in Table 1 in Supplementary Information, a nest PCR was used to acquire an accurate 3′ ending sequence. In the resulting sequence (listed in Supplementary information “3′RACE results”), the whole fragment-ending codon TGA and poly A sequence are both present. This indicates that the full sequence was obtained. In total a 2666 bp fragment was obtained in the end.

A sequence alignment with sequences from snakes, human, rabbit, rat and mouse was performed, with the results being shown in Fig. 1. The query sequence (resulting sequence) has a high similarity between the conserved regions of the snakes, which was expected because the resulting sequence was primed from it. To our surprise, the results indicate that the extending part of the sequences beyond the conserved region is more similar to other mammals (human, rabbit, rat and mouse) than other snakes. Specific blast results are given in Supplementary Information Figs 2~7.

**Figure 2:**
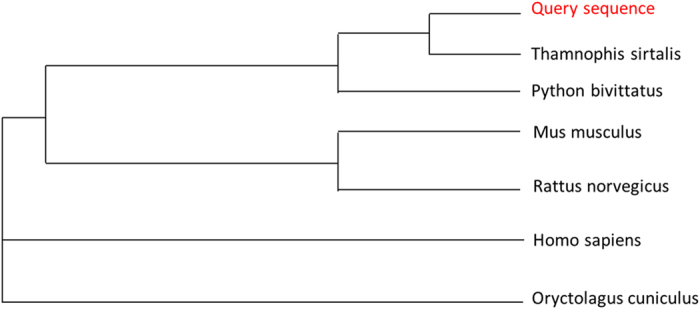
Dendrogram of ABCB11 from some known species. Query sequence, the resulting sequence from this research.

To better compare the homology of all known sequences, a dendrogram was mapped (see Fig. 2). It clearly demonstrates that the resulting sequence has high homology with two known snake sequences (*Thamnophis sirtalis* and *Python bivittatus*), which is expected as the sample is also from a snake liver (*Elaphe carinata*).

### Verification using 6 different snakes

**Figure 3:**
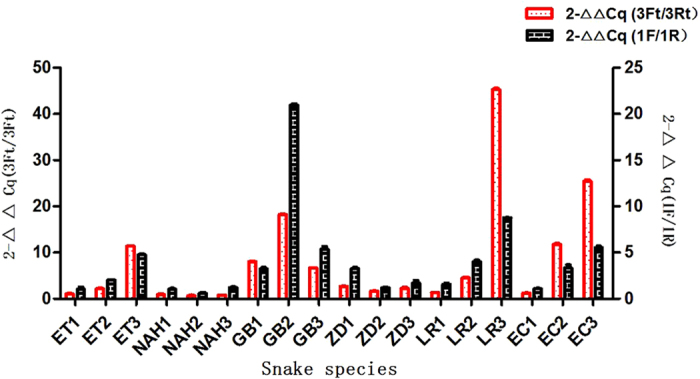
Q-PCR results of liver mRNA from different snake species. Duplicates from two sets of primers are shown in two columns. Elaphe carinata (EC), Elaphe taeniura (ET), Zaocys dhumnades (ZD), Natrix annularis Hallowell (NAH), Lycodon rufozonatus (LR), Gloydius brevicaudus (GB) were used here.

QPCR was used to verify whether the sequences could be PCR amplified in 5 other different snake species. The primer information is listed in Table 1. The relative amount of each fragment is given in Fig. 3. These results indicate that all the snakes contained the defined sequence information.

### Proteomic analysis

Protein extracts from *Elaphe carinata* liver were analyzed with a triple-TOF shotgun proteomic system. The data was searched against a different snake species (*Ophiophagus Hannah*) database that is known to contain BSEP protein. Proteins are listed in Supplementary Information.

There were 3186 proteins identified (FDR ≤ 1%), in which BSEP was included. Selected protein information is listed in Table 2, the extra proteins (fatty acid synthase and glycogen debranching enzyme) were selected to verify the reliability of this method. Additional BSEP peptides information is listed in Table 3. For BSEP, there were in total 11 peptides with a confidence greater than 95% being identified. Its unused score is 22.56, which is significantly greater than 1.410, which is the critical value of proteins with an FDR smaller than 1%.

Selected proteins are listed in Table 2 to validate the effectiveness of using a different snake species for the protein database to search against the data obtained in this experiment.

## Discussion

In order to validate the existence of BSEP and its encoding DNA, *ABCB/11*, PCR analysis was initially conducted. In this analysis, the first sequence serves both as evidence of BSEP’s existence in snakes and also as a starting point for later sequencing. In the snake (*Elaphe carinat*) liver sample, a known primer and a random primer were designed across the up-stream (or down-stream) and inside of the first sequence, so that the sequence could extend to both ends. In total, four fragments were successfully obtained through the standard PCR technique (detailed information is listed in Supplementary Information). However, the sequence did not reach the poly A end (3′ end), and the spliced sequence was only 2068 base pairs long, indicating that more of the sequence exists beyond the obtained one.

Because the total, larger fragment could not be acquired using a standard PCR technique, another technique, RACE (rapid amplification of cDNA ends) was applied. In total, there were 2666 base pairs identified in the final sequences, including the poly-A end which indicated the finish of a whole sequence at the 3′ end. However, we were unable to determine whether more sequence existed beyond the identified 5′ end. Even though we are not certain whether the characterized cDNA sequence is complete, this does not affect the conclusion that BSEP can be characterized from the genetic level.

All known sequences were aligned to determine their homology and similarity. The results clearly demonstrate that our resulting sequence has a reasonable similarity with other known *ABCB/11*, especially in the snake conserved region. A dendrogram using known *ABCB/11* sequences (Fig. 2) from other species indicates a homology pattern that relates the *ABCB/11* sequences to their species specificity. All this indicates that the sequencing of the DNA was reliable.

As shown in Fig. 3, more liver cDNA extracts from 6 different snake species were detected using Q-PCR based on primers specific for known BSEP sequences. The relative expression of RNA could be identified across all of the snake liver samples. The fact that all of these liver specimens substantially expressed BSEP mRNA indicates that the gene was expressed in all of the 6 different snake species studied here.

No empirical research has previously been reported on the existence of BSEP/Bsep in any snake species, except for 3 predicted cases reported in NCBI regarding the sequences of this particular protein. Our study is the first that provides concrete evidence of BSEP/Bsep existence at the genetic level.

From the protein level, the existence of BSEP was also identified. An advanced proteomic technique (Triple TOF based MS analysis) was applied on crude liver tissue. As the protein BSEP has not been characterized in the snakes studied here, an alternative searching method was applied, using the database of a snake species predicted to contain BSEP: *Ophiophagus Hannah*.

Two test proteins were analyzed first in order to assess the reliability of using different databases. The first was fatty acid synthase (tr|V8P8N6|V8P8N6_OPHHA), a highly expressed protein in fat, liver and mammary glands in mammals^9^,^10^,^11^. The second protein analyzed was glycogen debranching enzyme (tr|V8PGB5|V8PGB5_OPHHA), which is a key enzyme in glycogen degradation in the liver^12^. These two enzymes are common proteins in the liver. In the proteomic results, these two proteins were easily identified (see Table 2). Both of them showed a concrete and reliable identification. This fact suggest that our methodology using different databases was valid. As for BSEP, 11 peptides with confidence greater than 95% were identified. These data strongly suggests that the protein BSEP also exists in the studied snake liver.

In summary, here we show that BSEP does exist in the snake species researched in this study, *Elaphe carinat.* The mRNA could also be identified from 5 other snake species: *Elaphe taeniura, Zaocys dhumnades, Natrix annularis Hallowell, Lycodon rufozonatus* and *Gloydius brevicaudus*.

This study provides the first solid and reliable evidence of the existence of BSEP in snake species, including information on both genetic and proteomic levels. Furthermore, genetic manipulation of *ABCB11*, the gene expressing BSEP, is vital for the research of snake bile quality control, especially regarding the aspect of artificial breeding, production capacity and the sustainable utilization of this precious traditional drug.

Future research on the effect of BSEP on bile acid secretion would be important for snake bile research. A knock out of BSEP could be used to study the relationship of this protein with the content of bile acid. The above findings provide a solid foundation for the future study on BSEP manipulation from protein and genetic levels.

## Material and Methods

### Animal and tissue acquisition

Six different snake species were used in this study: *Elaphe carinata, Elaphe taeniura, Zaocys dhumnades, Natrix annularis Hallowell, Lycodon rufozonatus, and Gloydius brevicaudus*. Snakes were kept in a standard animal room with a 12 hour dark/light cycle (lights on at 7 am); the temperature was set at 22 ± 1 °C.Livers were collected when snakes were euthanized, the specimens were snap frozen with liquid nitrogen and then stored at −80 °C. All procedures were approved by Huazhong University of Science and Technology Tongji Medical College Animal Care and Ethics Committee. All methods described below were performed in accordance with the relevant guidelines and regulations.

### RNA isolation and cDNA analysis

Snake liver samples (50 mg) were homogenized with 1 mL of trizol (thermofisher, USA) solution. 200 μL of chloroform was added and samples were mixed thoroughly. Samples were then centrifuged at 12,000 g for 15 min, with the supernatant transferred into 1.5 mL tubes (Axygen MCT-150-C, USA) and mixed with 600 μL of isopropanol (Sigma-Aldrich, USA). The solutions were centrifuged at 12,000 g for 10 min and the pellet was washed with 1 mL of 75% ethanol. The resulting solutions were centrifuged again at 12,000 g for 5 min, the pellets were dissolved with DEPC liquor and stored at 4 °C.The cDNA were synthesized following the protocol of the cDNA Synthesis Kit (TOYOBO, Japan).

### Sequence analysis and Quantitative real-time PCR

**Figure 4:**
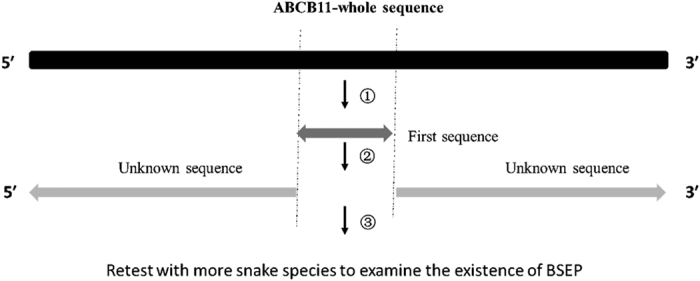
Sequence analysis design. The bar stands for gene sequences. The first sequence was obtained through PCR amplification from a conserved region of predicted sequences from different snakes (①), extended sequences were subsequently obtained with primers based the first sequences and the other parts of predicted sequences (②), finally, more snakes liver samples was used to re-test the existence of BSEP.

As summarized in Fig. 4, the sequences of cDNA from the liver of six different snake species were analyzed using primers based on highly conserved regions between the predicted sequence of BSEP/Bsep from *Ophiophagus Hannah, Thamnophis sirtalis and Python bivittatus*. The mRNA sequence of ABCB11 in *Ophiophagus Hannah* was obtained from a whole genome shotgun sequence (GenBank: AZIM01000723.1) provided in NCBI (The National Centre for Biotechnology Information).mRNA of *Thamnophis sirtalis* and *Python bivittatus* were obtained from NCBI directly (Reference number are XM_014063741.1 and XM_007421852.1, respectively). The primer sequences are listed in Table 1. PCR was performed using a Utaq mix kit, as per instructions. First the sequences were obtained using primers based on conserved regions. Following sequences were obtained using primers designed using the first sequence and the predicted sequence from NCBI (primers are listed in Table 1).

The rapid amplification of cDNA ends (RACE) technique was used to obtain the whole sequence of a specimen from *Elaphe carinata* liver, similar with a method described elsewhere^13^. After an initial denaturation temperature of 95 °C for 10 min, PCR was performed at 95 °C for 40 s, at 50–60 °C for 30 s, and at 72 °C for 1 min + 30 s for a total of 35 cycles, followed by a final extension of 10 min at 72 °C. Primer information is given in Table 1 in the Supplementary Information.

Quantitative PCR was conducted using FAST SYBR Green Master Mix on a CFX Connect Real-Time PCR System (Bio-Rad, Hercules, CA, USA). Relative expression of the mRNA was calculated using the 2−ΔΔCt method.

### Verification of BSEP using 6 different snake species

Six different snake species (3 individuals for each): *Elaphe carinata, Elaphe taeniura, Zaocys dhumnades, Natrix annularis Hallowell, Lycodon rufozonatus and Gloydius brevicaudus* were analyzed using Q-PCR using the BIO-RAD system Syb Green protocol to verify the feasibility of BSEP’s presence.

### Proteomics on Elaphe carinat liver protein

As described elsewhere^14^, a proteomic technique using Triple-TOF was used to scan existed proteins in the liver sample of *Elaphe carinata.* Protein was extracted from crude liver tissue using acetone precipitation after homogenizing the liver in a cell lysis buffer. Trypsin (Sigma-Aldrich, USA) was used to digest proteins into peptides with a ratio of 1:50 (trypsin: protein). The following solution was desalted using Sep-Pak C18 columns as instructed. Reversed phase HPLC was applied to fractionate peptides into 15 fractions according to their affinity to the column. Resulting fractions were analyzed using RPLC-ESI-MS/MS, and MS spectrum information was searched against the *Ophiophagus Hannah* database.

## Additional Information

**How to cite this article**: Tan, X. *et al*. Genetic and Proteomic characterization of Bile Salt Export Pump (BSEP) in Snake Liver. *Sci. Rep.* **7**, 43556; doi: 10.1038/srep43556 (2017).

**Publisher's note:** Springer Nature remains neutral with regard to jurisdictional claims in published maps and institutional affiliations.

## Supplementary Material

Supplementary Information

## Figures and Tables

**Table 1:** Designed primers used for PCR and DNA sequencing. 1 F/1 R and 3Ft/3Rt were used in QPCR as well.

primers	Sequence (5′ → 3′)
1 F	AGCAACTGACGCCTCTCAAG
1 R	GCCTGTCAGCATTCGAGACT
2 F	GAGCATCTCTTCGACAGCGT
2 R	ACTTGAGAGGCGTCAGTTGC
3 F	TGAGGCTACCGTGCAAGAGA
3 R	TCCTGGTCACGTTAGCAGGT
3Ft	TGGCTTCCAGGCGATGTTAG
3Rt	GTCGCCCCTTGAACTTGAGA
4 F	GGACATAGGCTGGTTTGATTGTAC
4 R	TCAAACCCAACGATAATGTCAGCAG

**Table 2:** Proteomic information of the selected proteins.

N	Unused	Accession	Name	Species	Peptides (95%)
8	101.77	tr|V8P8N6|V8P8N6_OPHHA	Fatty acid synthase (Fragment) OS = Ophiophagus hannah GN = FASN PE = 4 SV = 1	OPHHA	60
14	89.44	tr|V8PGB5|V8PGB5_OPHHA	Glycogen debranching enzyme (Fragment) OS = Ophiophagus hannah GN = AGL PE = 4 SV = 1	OPHHA	54
336	22.56	tr|V8P6M9|V8P6M9_OPHHA	Bile salt export pump (Fragment) OS = Ophiophagus hannah GN = ABCB11 PE = 4 SV = 1	OPHHA	11

**Table 3:** Peptide information of identified BSEP.

Contrib	Conf	Sequence	Modifications	Cleavages	dMass	Prec MW	Prec m/z	Theor MW	Theor m/z	Theor z
2	99.00000095	AGAIADEVLSSIR			0.000592131	1300.699219	651.3569	1300.69873	651.3566284	2
2	99.00000095	GAATNIFETIDEKPR			−0.00584046	1660.836304	831.4254	1660.842041	831.4282837	2
2	99.00000095	GVYFTLVTLQSQGDK			0.000708538	1654.857422	828.436	1654.856689	828.4356079	2
2	99.00000095	ISNEALANIR			−0.000604897	1099.598022	550.8063	1099.598511	550.8065796	2
2	99.00000095	LLLLDMATSALDNESEATVQK			−0.0018081	2261.143311	1131.579	2261.145996	1131.580322	2
2	99.00000095	NLVFAQNWGIR			0.00141097	1316.700439	659.3575	1316.698975	659.3567505	2
1.698970437	98.89000058	SLNIQWLR			−0.0014581	1028.575317	515.2949	1028.57666	515.2956543	2
1.619789004	98.6800015	EKTFINAYEK		missed K-T@2	0.000771041	1241.630127	621.8223	1241.62915	621.8218994	2
1.619789004	98.71000051	LTGLELK			0.000923233	772.470459	387.2425	772.4694824	387.2420044	2
1.585026264	98.55999947	IDCMSEDGYK	Carbamidomethyl(C)@3		−0.00127288	1216.4729	609.2437	1216.473999	609.2442627	2
1.468521357	98.11999798	ASSYTPNYAK			0.00137648	1100.515259	551.2649	1100.513794	551.2642212	2
