# Supplementary material for: Genetic and Proteomic characterization of Bile Salt Export Pump (BSEP) in Snake Liver
Source: Sci Rep. 2017 Apr 3;7:43556. doi: 10.1038/srep43556 (PMC5377248; doi:10.1038/srep43556)
Supplement: Supplementary Information [file srep43556-s1.pdf]

Supplementary information

Genetic and Proteomic characterization of Bile Salt Export Pump (BSEP) in Snake Liver

Xinle Tan[1, 2, ‡], Fei Gao[1,‡] , Hexiu Su[3], Yajun Gong[1], Jie Zhang[1], Mitchell A. Sullivan[4], Jiachun Chen[1,※]

[1]Tongji School of Pharmacy, Huazhong University of Science and Technology, Wuhan, Hubei 430030, China

[2]The University of Queensland, School of Chemistry & Molecular Bioscience, Brisbane, QLD 4072, Australia

[3]Department of Hematology, Affiliated Union Hospital, Tongji Medical College, Huazhong University of Science and Technology

[4] Program in Genetics and Genome Biology, The Hospital for Sick Children, Toronto, ON, M5G 1X8, Canada.

‡ These authors contributed equally to this work. Correspondence and requests for materials should be addressed to Jiachun Chen (email: homespringchen@126.com; homespringchen@mail.hust.edu.cn)

1. Predicted ABCB/11 sequences from 3 known snakes

There are already predicted sequences of ABCB/11 from 3 snakes: *Ophiophagus Hannah, Thamnophis sirtalis and Python bivittatus.* Based on the shared sequences (conserved region), primers were designed and PCR amplified on the sample, which is exampled as *Elaphe carinata-P.*

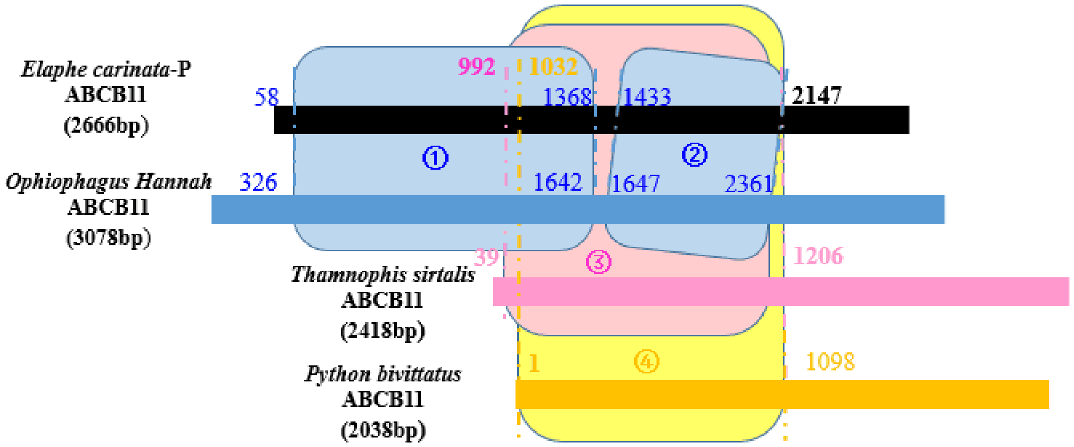

Fig. 1 Conserved region in predicted sequences between three different snakes. The colored square stands for shared sequences in different species. There were 2666bp identified from Elaphe carinata-P, which is detailed in the main text.

2. First sequences information:

Based on the conserved region of the three predicted snake ABCB/11 sequences, primers were designed and PCR amplification gives the sequences listed as below (red text is the primer sequences):

AGCAACTGACGCCTCTCAAGTTCAAGGGGCGACTGGCACACAGATAGGCATGATTGTGAATTCCTTCACCAATATCGGAGTCTC
CATTATTATCGCTTTCTACTTCAGCTGGAAACTCAGCTTAGTCATAACCTGCTTCTTGCCTTTCTTGGCTTTGTCAGGAATGGTTC**A**
GTCTCGAATGCTGACAGGC

3. Sequence information of later fragments:

Later fragment information were obtained based on the first sequences, detailed information were listed as below (red text is the primers):

Fragment 2

GAGCATCTTCTTCGACAGCGTTCCAGATCTCAGTTGTCAAACATAGTCCCTGACCATCCAGCGTCAATTGTAGGATCCTATACAG
AGCGCAACTGTAGCATATCTCACTATGAAGAAGATGGAAGACCATTTATGAAGGAACGGATTCATGAAAAGGATGAGGAAGAA
GTCGTACCTGCTAACGTGACCAGGATTTTGAAGTACAACGCTCCCGAATGGCCCTATATGGTGCTTGGATCCCTTGGGGCAGGG

GTCAATGGGGCAGTCAACCCTCTCTATGCCCTGCTCTTCAGTCAAATTCTTGGAACCTTTTCGGTGCCTGATGAAGAAGAGCAA
AGAGCTCAGATCAACGGTGTCTGCTTGCTCTTTGTGGTGGTTGGAATCGTATCATTCTTCACTCAGTTCTTACAGGGCTATACGTT
TGCAAAGGCCGGAGAATTGCTTACACGACGCTTAAGGAAAATTGGCTTCCAGGCGATGTTAGGCCAAGAAATTGGCTGGTTTG
ATGACCATAGAAACAGCCCAGGGGCCTTGACTACGAGACTA**GCAACTGACGCCTTCCTCAAGT**

Fragment 3

TGAGGCTACCGTGCAAGAGACACTTTCCAAGGCTCGTTTTGGGCGCACCGCAATCTCCATAGCTCACCGTTTGTCCACAGTGA
AATCTGCTGACATTATCGTTGGGTTTGAGCATGGAAGAGCTGTGGAAAGAGGGAAACATGAAGAACTGATAGAACGGAAAGG
AGTGTATTTCACACTGGTAACGTTGCAAAGCCAAGGCGACAAGGCACTCATGGAAAGCTCAATGCAAATATCTGACCCAAAACC
TGAAAAAATGCAGTCTTTCAGCAGGGGAAGCTATCAAGCAAGTTTACGAGCATCTCTTCGACAGCGTTCCAGATCTCAGTTGTC
AAACATAGTCCCTGACCATCCAGCGTCAATTGTAGGATCCTATACAGAGCGCAACTGTAGCATATCTCACTATGAAGAAGATGGA
AGACCATTTATGAAGGAACGGATTCATGAAAAGGATGAGGAAGAAGTCGT**ACCTGCTAACGTGACCAGGA**

Fragment 4

GGACATTGGCTGGTTTGATTGTACATCAGTAGGGGAACTCAATACGCGAATTTCTGAGATTTGTCAGAGGCTGGAAGTTAACC
TTGGTTATTATCGCAGTCAGTCCTCTAATTGGGATCGGAGCTGGCATAATTGGCCTGTCTGTAGCAAAGTTGACGGGTCTAGAGT
TAAAAGCTTATGCAAAAGCTGGGGCCATCGCTGATGAAGTGCTCTCATCGATCCGAACCGTGGCTGCCTTTGGAGGAGAAAAA
AAAGAAGTTGAAAGATATGACAAAAACCTGGTGTTTGCTCAAACTGGGGGATCAGAAAGGGAATCATTATGGGATTTTTCACA
GGCTACATTTGGTGCATCATTTTCCTGTGCTATGCACTGGCCTTCTGGTATGGCTCAAAACTTGTCCTTGAAGAAGAGGAATACT
CTGCTGGCACCCTTTTGCAGGTCTTTTCTCGGCGTCCTAGTAGGTGCCTTAAATCTTGGTCAAGCAGCCCCTTGCTTGGAAGCTT
TTGCAACAGGTCGAGGGGCTGCCACAAATATATTTGAGACAATAGATGAAAAACCTAGAATTGACTGTATGTCAGAAGATGGCT
ACAAACTGGACAAAGTCCGAGGTGACATTGAATTCCATAATGTAACCTTCCATTACCCATCCAGGCCCGATGTTAAGATTTTAGA
TCAACTCAGCATGATTATCAAGCCAGGTGAAACCACAGCCTTTGTTGGCCCAAGCGGAGCAGGGAAAAGTACCACAATTCAGC
TGATCCAGCGCTTCTATGACCCTACAGAAGGCATGGTTACCCTAGATGGACACGACATTCGTTCCCTTAACATTCAGTGGCTGCG
TTCTTTGATGGGGGTGGTGGAGCAAGAACCTGTCCTGTTTGCCACCACTATAGCAGAAAACATTTCCTACGGTCGGGAAGATG
CCACTATGGAAGACATCGTCAGAGCTTCCAAAGAAGCCAATGCCTACAGCTTTATCATGGATCTGCCTCTGCAATTCGACACCCT
TGTTGGAGAAGGTGGTGGCCAAATGAGCGGAGGCCAGAAGCAGCGAATTGCCATTGCTCGGGCTTTAGTTCGAAACCCCAAA
ATCCTGTTGCTGGACATGGCTACATCAGCACTGGACAATGAAAGTGAAGCTACTGCAATAAAACTCGACCT

3’RACE results

When RACE was used to obtain the extra sequence information, different primers were used (see table. 1). The resulting sequences were listed below:

CGCTTTCTACTTCAGCTGGAAACTCAGCTTAGTCATAACCTGCTTCTTGCCTTTCTTGGCTTTGTCAGGAATGGTTCAGCATTCA
ATGCAAATGGTGGCATTGAACTGTATTTCCTACTGCTCCATCATCATCATCATCTCGCTCTCAATGATTCTGGGTGGTTCACAACAA
TCAAAGAAATGACAATGAAATCAATAAAAATGAAGAAAATGAAGATAAAGGATGGCAAGAGCGATGAAGTGAGGGATCTCTCT
CTCCCTTACCAAAGACCTTCCTTACCAAAGAGCCTGGTCCTCCTAGCTTCTAAACAACAATAGAGTGGGGGACCACCCACATCTT
GGGAGACCCTTGCTCCAGAGGGCTGCAAGAGAGAAGGACGTATTCACCATTATGATGGGGAAATACCAGACGCTGATTCTTAC
ATCCATAAAAGAGCAATATTGAAACCTGACTCTGCGAATCTCTAGGGATTCTCTTATATATACAATGTCTTCTGTGTGTAATGGTTC
TCTACGTAGTGTTCAAAGCACTTTGGCCAGATTACAAAATCCAATTATTCTATTCACTGCCA*TGA*AAAAAAAAAAAAAAAAAAA
A

Table 1. Information of primers in 3' RACE

procedure	primers	Sequence (5'→3')
cDNA synthesis	3RACEP1	gcagtggtatcaacgcagagtac-TTTTTTTTTTTTTTTTTTTTTTTTTTVN
Nest PCR	Forward 1 (F1)	CCTTCACCAATATCGGAGTCTC
	Forward 2 (F2)	CGCTTTCTACTTCAGCTGGAAAC
	R1=R2 =3RACEP2	gcagtggtatcaacgcagagtac

4. Spliced whole sequences of BSEP from *Elaphe carinata*

Together, all the sequences were spliced in a single sequence, listed as below:

GGACATTGGCTGGTTTGATTGTACATCAGTAGGGGAACTCAATACGCGAATTTCTGAGATTTGTCAGAGGCTGGAAGTTAACCT
TGGTTATTATCGCAGTCAGTCCTCTAATTGGGATCGGAGCTGGCATAATTGGCCTGTCTGTAGCAAAGTTGACGGGTCTAGAGTT
AAAAGCTTATGCAAAAGCTGGGGCCATCGCTGATGAAGTGCTCTCATCGATCCGAACCGTGGCTGCCTTTGGAGGAGAAAAAA
AAGAAGTTGAAAGATATGACAAAAACCTGGTGTTTGCTCAAACTGGGGGATCAGAAAGGGAATCATTATGGGATTTTTCACAG
GCTACATTTGGTGCATCATTTTCCTGTGCTATGCACTGGCCTTCTGGTATGGCTCAAAACTTGTCCTTGAAGAAGAGGAATACTCT
GCTGGCACCCTTTTGCAGGTCTTTTCTCGGCGTCCTAGTAGGTGCCTTAAATCTTGGTCAAGCAGCCCCTTGCTTGGAAGCTTTT
GCAACAGGTCGAGGGGCTGCCACAAATATATTTGAGACAATAGATGAAAAACCTAGAATTGACTGTATGTCAGAAGATGGCTAC
AAACTGGACAAAGTCCGAGGTGACATTGAATTCCATAATGTAACCTTCCATTACCCATCCAGGCCCGATGTTAAGATTTTAGATC
AACTCAGCATGATTATCAAGCCAGGTGAAACCACAGCCTTTGTTGGCCCAAGCGGAGCAGGGAAAAGTACCACAATTCAGCTG
ATCCAGCGCTTCTATGACCCTACAGAAGGCATGGTTACCCTAGATGGACACGACATTCGTTCCCTTAACATTCAGTGGCTGCGTT
CTTTGATGGGGGTGGTGGAGCAAGAACCTGTCCTGTTTGCCACCACTATAGCAGAAAACATTTCCTACGGTCGGGAAGATGCC
ACTATGGAAGACATCGTCAGAGCTTCCAAAGAAGCCAATGCCTACAGCTTTATCATGGATCTGCCTCTGCAATTCGACACCCTTG
TTGGAGAAGGTGGTGGCCAAATGAGCGGAGGCCAGAAGCAGCGAATTGCCATTGCTCGGGCTTTAGTTCGAAACCCCAAAAT
CCTGTTGCTGGACATGGCTACATCAGCACTGGACAATGAAAGTGAGGCTACCGTGCAAGAGACACTTTCCAAGGCTCGTTTTG
GGCGCACCGCAATCTCCATAGCTCACCGTTTGTCCACAGTGAAATCTGCTGACATTATCGTTGGGTTTGAGCATGGAAGAGCTG
TGGAAAGAGGGAAACATGAAGAACTGATAGAACGGAAAGGAGTGTATTTCACACTGGTAACGTTGCAAAGCCAAGGCGACA
AGGCACTCATGGAAAGCTCAATGCAAATATCTGACCCAAAACCTGAAAAAATGCAGTCTTTCAGCAGGGGAAGCTATCAAGCA
AGTTTACGAGCATCTCTTCGACAGCGTTCCAGATCTCAGTTGTCAAACATAGTCCCTGACCATCCAGCGTCAATTGTAGGATCCT
ATACAGAGCGCAACTGTAGCATATCTCACTATGAAGAAGATGGAAGACCATTTATGAAGGAACGGATTCATGAAAAGGATGAG
GAAGAAGTCGTACCTGCTAACGTGACCAGGATTTTGAAGTACAACGCTCCCGAATGGCCCTATATGGTGCTTGGATCCCTTGGG
GCAGGGGTCAATGGGGCAGTCAACCCTCTCTATGCCCTGCTCTTCAGTCAAATTCTTGGAACCTTTTCGGTGCCTGATGAAGAA
GAGCAAAGAGCTCAGATCAACGGTGTCTGCTTGCTCTTTGTGGTGGTTGGAATCGTATCATTCTTCACTCAGTTCTTACAGGGCT
ATACGTTTGCAAAGGCCGGAGAATTGCTTACACGACGCTTAAGGAAAATTGGCTTCCAGGCGATGTTAGGCCAAGAAATTGGC
TGGTTTGATGACCATAGAAACAGCCCAGGGGCCTTGACTACGAGACTAGCAACTGACGCCTCTCAAGTTCAAGGGGCGACTG
GCACACAGATAGGCATGATTGTGAATTCCTTCACCAATATCGGAGTCTCCATTATTATCGCTTTCTACTTCAGCTGGAAACTCAGC

TTAGTCATAACCTGCTTCTTGCCTTTCTTGGCTTTGTCAGGAATGGTTCAGCATTCAATGCAAATGGTGGCATTGAACTGTATTTC
CTACTGCTCCATCATCATCATCATCTCGCTCTCAATGATTCTGGGTGGTTCACAACAATCAAAGAAATGACAATGAAATCAATAAA
AATGAAGAAAATGAAGATAAAGGATGGCAAGAGCGATGAAGTGAGGGATCTCTCTCCCTTACCAAAGACCTTCCTTACCAA
AGAGCCTGGTCCTCCTAGCTTCTAAACAACAATAGAGTGGGGGACCACCCACATCTTGGGAGACCCTTGCTCCAGAGGGCTGC
AAGAGAGAAGGACGTATTCACCATTATGATGGGGAAATACCAGACGCTGATTCTTACATCCATAAAAGAGCAATATTGAAACCT
GACTCTGCGAATCTCTAGGGATTCTCTTATATATACAATGTCTTCTGTGTGTAATGGTTCTCTACGTAGTGTTCAAAGCACTTTGGC
CAGATTACAAAATCCAATTATTCTATTCACTGCCATGAAAAAAAAAAAAAAAAAAAAAAAA

5. Blast result between resulting sequences and other species

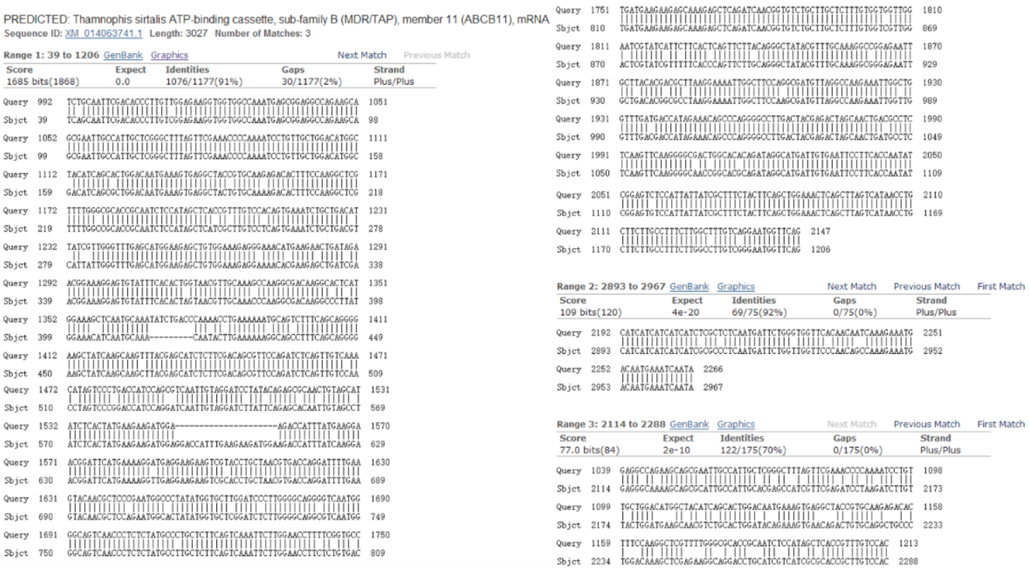

Fig. 2 blast result of resulting sequences and *Thamnophis sirtalis*

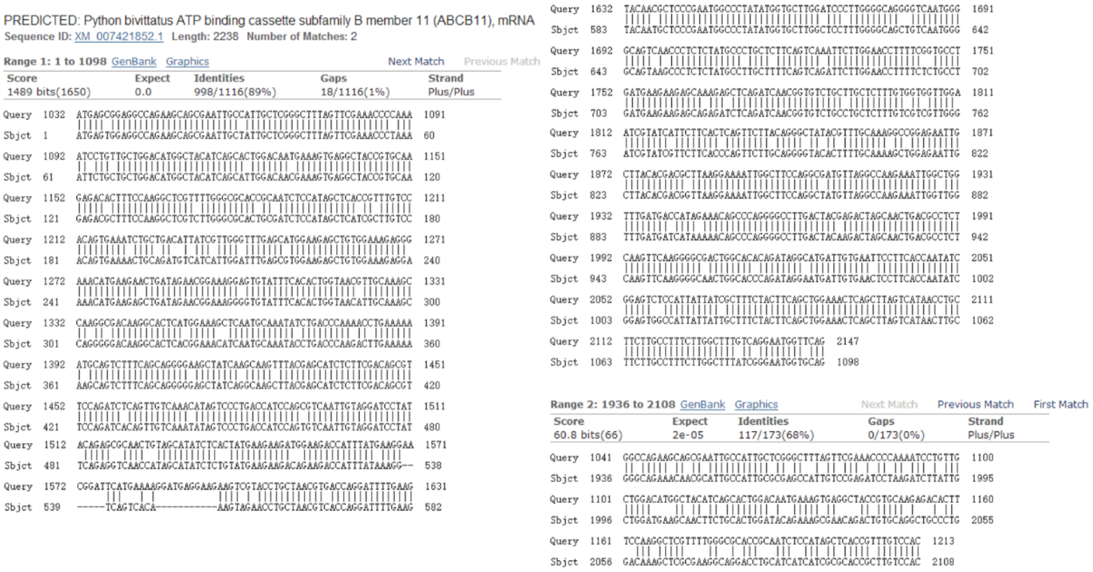

Fig. 3 blast result of resulting sequences and *Python bivittatus*

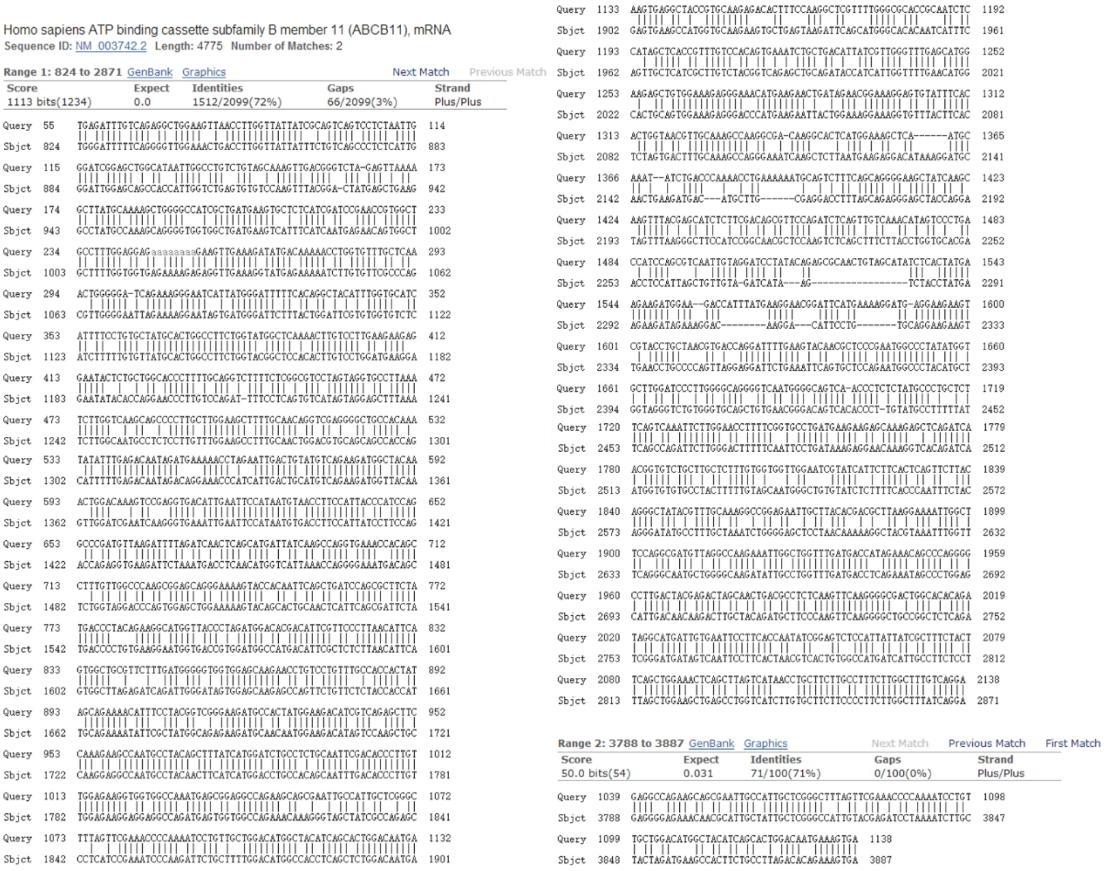

Fig. 4 blast result of resulting sequences and *Homo sapiens*

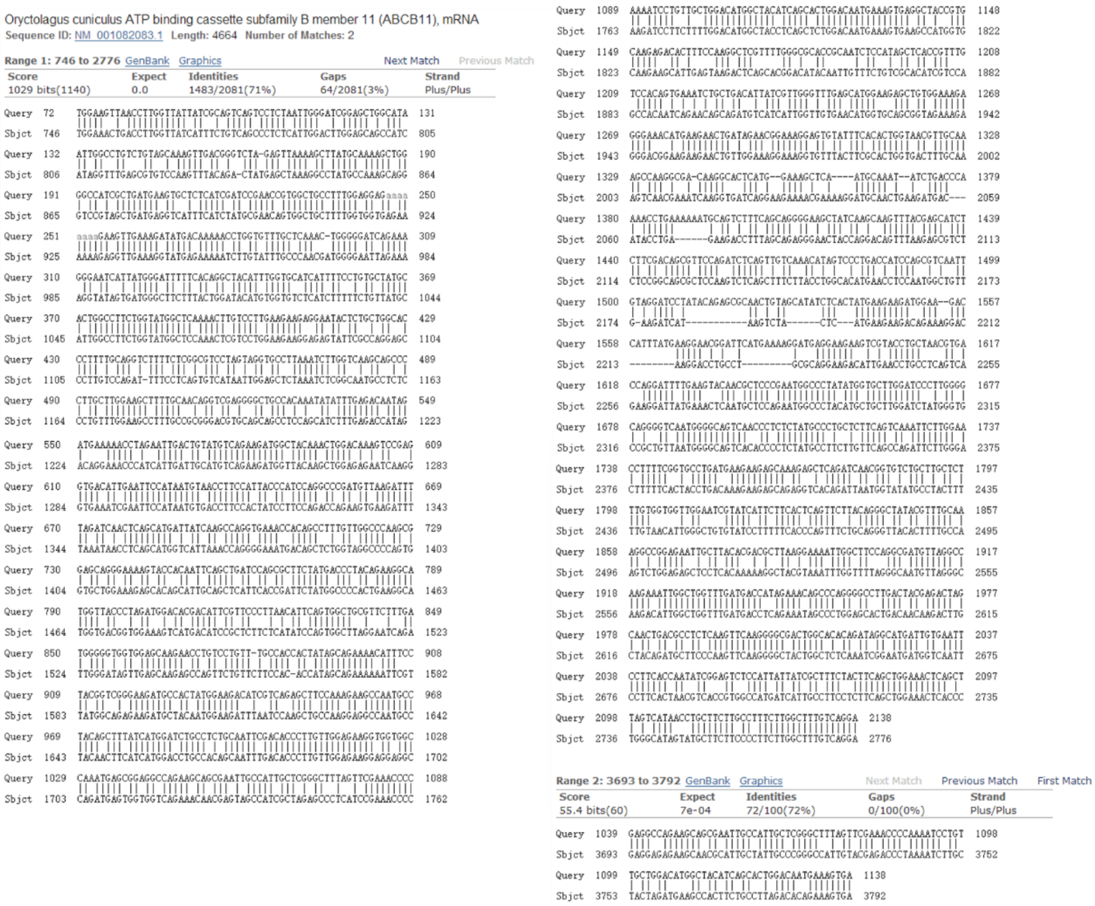

Fig. 5 blast result of resulting sequences and *Oryctolagus cuniculus*

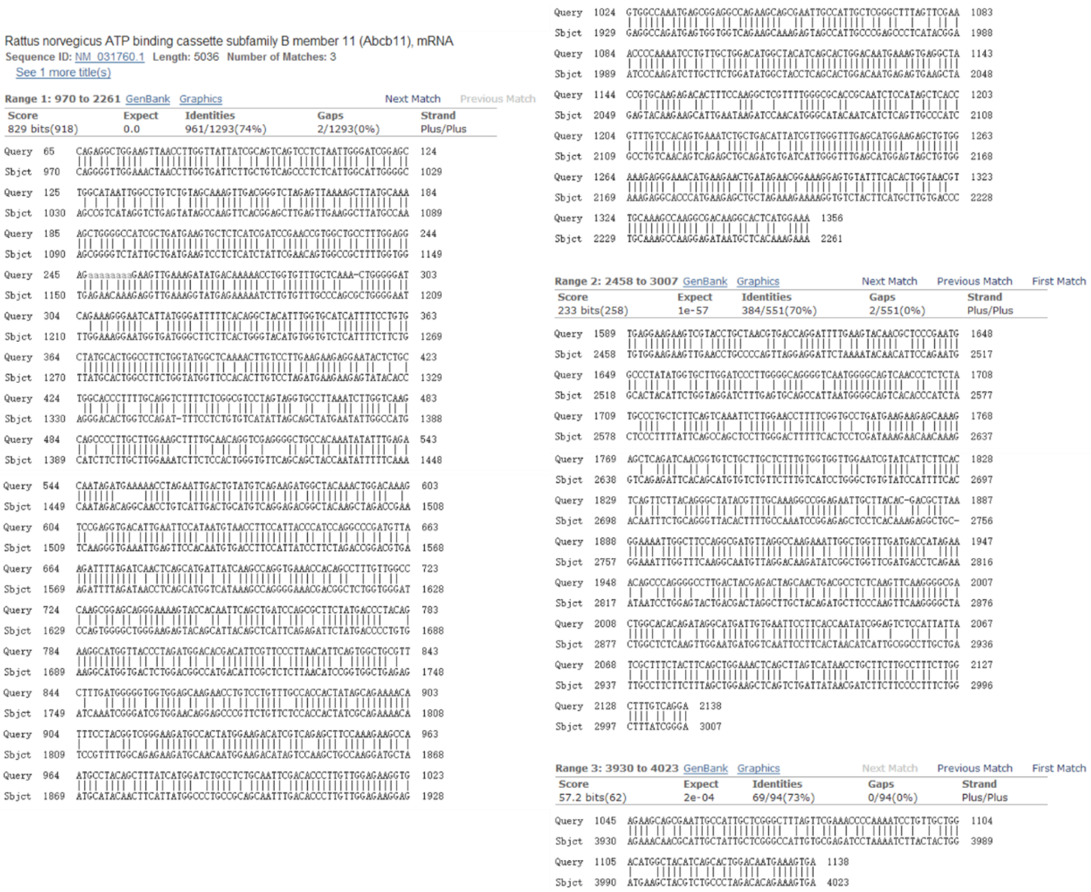

Fig. 6 blast result of resulting sequences and *Rattus norvegicus*

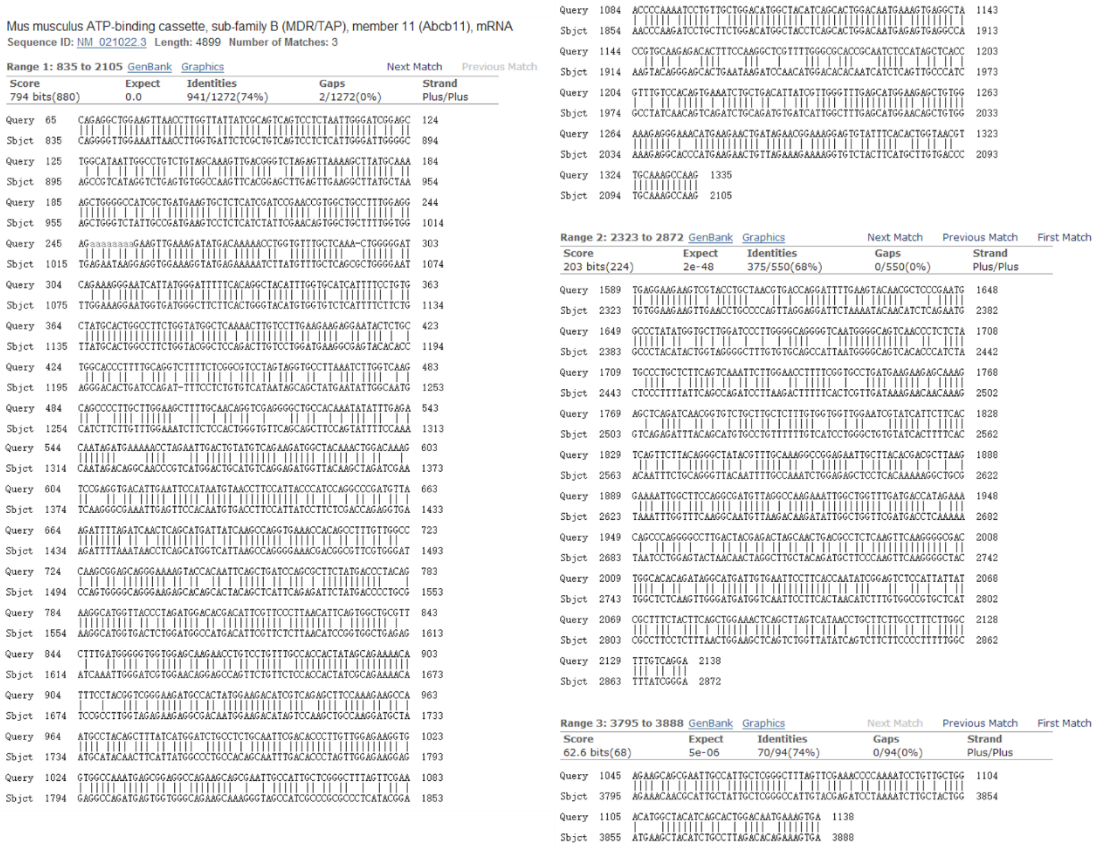

Fig. 7 blast result of resulting sequences and *Mus musculus*

6. Proteomic result of *Ophiophagus Hannah* liver proteins.

Shotgun proteomic was applied to protein extract of *Ophiophagus Hannah* liver, the resulting proteins were listed as below (start from next page):

N	Unused	Total	%Cov	%Cov(50)	%Cov(95)	Accession	Name	Species	Peptides(95%)
1	230.29	230.29	83.87	53.78	49.19	tr|V8PJW7|V8PJW7_OPHHA	Myosin-10 (Fragment) OS=Ophiophagus hannah GN=Myh10 PE=	OPHHA	157
2	191.36	191.36	83.1	57.67	52.15	tr|V8PJJ1|V8PJJ1_OPHHA	Spectrin beta chain, brain 1 (Fragment) OS=Ophiophagus	OPHHA	110
3	172.61	172.61	65.48	48	44.99	tr|V8P9W0|V8P9W0_OPHHA	Filamin-A OS=Ophiophagus hannah GN=FLNA PE=4 SV=1	OPHHA	104
4	171.18	204.26	80.03	50.84	47.64	tr|V8PFF2|V8PFF2_OPHHA	Myosin-9 (Fragment) OS=Ophiophagus hannah GN=MYH9 PE=4	OPHHA	140
5	149.77	154.12	64.13	43.59	39.04	tr|V8P4P2|V8P4P2_OPHHA	Filamin-B (Fragment) OS=Ophiophagus hannah GN=FLNB PE=4	OPHHA	87
6	116.36	116.36	62.53	47.73	44.06	tr|V8P2J0|V8P2J0_OPHHA	Clathrin heavy chain 1 (Fragment) OS=Ophiophagus hannah	OPHHA	71
7	108.91	108.91	71.28	35.77	31.62	tr|V8NH62|V8NH62_OPHHA	Neuroblast differentiation-associated protein AHNAK (Fr	OPHHA	94
8	101.77	101.77	46.72	30.28	28.12	tr|V8P8N6|V8P8N6_OPHHA	Fatty acid synthase (Fragment) OS=Ophiophagus hannah GN	OPHHA	60
9	101.56	101.56	61.38	29.17	22.64	tr|V8NJK8|V8NJK8_OPHHA	Cytoplasmic dynein 1 heavy chain 1 (Fragment) OS=Ophiop	OPHHA	72
10	100.03	100.03	44.67	15.63	13.03	tr|V8NQB4|V8NQB4_OPHHA	Apolipoprotein (Fragment) OS=Ophiophagus hannah GN=Apob	OPHHA	63
11	94.85	94.85	72.87	37.27	31.52	tr|V8PI16|V8PI16_OPHHA	Kinectin OS=Ophiophagus hannah GN=KTN1 PE=4 SV=1	OPHHA	62
12	92.85	92.85	58.1	26.84	22.12	tr|V8NS95|V8NS95_OPHHA	Utrophin (Fragment) OS=Ophiophagus hannah GN=UTRN PE=4	OPHHA	50
13	89.46	89.46	47.78	24.47	19.87	tr|V8PCU6|V8PCU6_OPHHA	Low-density lipoprotein receptor-related protein 1 (Fra	OPHHA	59
14	89.44	89.44	57.07	34.01	31.98	tr|V8PGB5|V8PGB5_OPHHA	Glycogen debranching enzyme (Fragment) OS=Ophiophagus h	OPHHA	54
15	89.18	89.18	47.69	22.37	20.63	tr|V8P3W1|V8P3W1_OPHHA	Collagen alpha-1(XII) chain (Fragment) OS=Ophiophagus h	OPHHA	47
16	88.47	88.47	54.36	33.98	28.93	tr|V8P133|V8P133_OPHHA	Ras GTPase-activating-like protein IQGAP2 OS=Ophiophagu	OPHHA	48
17	84.5	84.5	83.97	61.78	56.84	tr|V8P2D8|V8P2D8_OPHHA	Alpha-actinin, sarcomeric (Fragment) OS=Ophiophagus han	OPHHA	57
18	81.1	81.1	74.29	31.68	24.27	tr|V8NTQ8|V8NTQ8_OPHHA	Desmoplakin OS=Ophiophagus hannah GN=Dsp PE=4 SV=1	OPHHA	41
19	80.59	80.59	61.68	48.47	42.69	tr|V8P5F2|V8P5F2_OPHHA	Endoplasmin (Fragment) OS=Ophiophagus hannah GN=HSP90B1	OPHHA	54
20	79.47	79.47	89.69	63.91	62.97	tr|V8P294|V8P294_OPHHA	Phosphoenolpyruvate carboxykinase [GTP], mitochondrial	GTP	58
21	74.48	74.48	77.77	46.42	38.29	tr|V8NVB7|V8NVB7_OPHHA	Vinculin (Fragment) OS=Ophiophagus hannah GN=VCL PE=4 S	OPHHA	42
22	74.34	74.34	72.66	49.45	46.7	tr|V8N9M0|V8N9M0_OPHHA	Heat shock protein HSP 90-alpha (Fragment) OS=Ophiophag	OPHHA	46
23	73	73	52.34	43.09	37.4	tr|V8P0K4|V8P0K4_OPHHA	AP-2 complex subunit beta (Fragment) OS=Ophiophagus han	OPHHA	37
24	71.47	71.47	72.82	53.48	46.85	tr|V8NX72|V8NX72_OPHHA	Catenin alpha-1 OS=Ophiophagus hannah GN=CTNNA1 PE=4 SV	OPHHA	37
25	70.8	70.8	72.03	42.73	40.95	tr|V8NWL5|V8NWL5_OPHHA	C-1-tetrahydrofolate synthase, cytoplasmic (Fragment) O	OPHHA	44
26	69.72	69.72	61.37	35.14	29.98	tr|V8NJ00|V8NJ00_OPHHA	Vigilin (Fragment) OS=Ophiophagus hannah GN=HDLBP PE=4	OPHHA	41
27	69.68	69.68	57.21	19.45	17.08	tr|V8NVT7|V8NVT7_OPHHA	Laminin subunit gamma-1 (Fragment) OS=Ophiophagus hanna	OPHHA	41
28	66.37	66.37	66.25	26.91	20.37	tr|V8P9J0|V8P9J0_OPHHA	Eukaryotic translation initiation factor 3 subunit A OS	OPHHA	31
29	66.28	66.28	45.26	21.28	18.6	tr|V8NJM6|V8NJM6_OPHHA	S-(hydroxymethyl)glutathione dehydrogenase (Fragment) O	OPHHA	44
30	64.59	64.59	63.79	40.08	35.12	tr|V8PFP1|V8PFP1_OPHHA	Trifunctional purine biosynthetic protein adenosine-3 O	OPHHA	46
31	64.22	64.22	72.01	44.18	41.51	tr|V8NJH5|V8NJH5_OPHHA	Protein disulfide-isomerase A4 OS=Ophiophagus hannah GN	OPHHA	36
32	64.2	64.2	34.83	21.79	19.74	tr|V8P062|V8P062_OPHHA	Fibronectin (Fragment) OS=Ophiophagus hannah GN=FN1 PE=	OPHHA	37
33	64.05	64.05	76.25	49.85	42.96	tr|V8NXQ5|V8NXQ5_OPHHA	Annexin (Fragment) OS=Ophiophagus hannah GN=ANXA6 PE=3	OPHHA	36
34	62.1	62.1	73.4	44.83	41.54	tr|V8NEC1|V8NEC1_OPHHA	78 kDa glucose-regulated protein OS=Ophiophagus hannah	OPHHA	48
35	59.51	59.51	55.03	30.09	23.73	tr|V8NYF2|V8NYF2_OPHHA	UDP-glucose:glycoprotein glucosyltransferase 1 (Fragmen	OPHHA	30
36	58.69	58.69	56.67	40.04	33.79	tr|V8NUK3|V8NUK3_OPHHA	AP-2 complex subunit alpha-2 (Fragment) OS=Ophiophagus	OPHHA	30
37	58.69	58.69	76.15	50.71	50.71	tr|V8NM23|V8NM23_OPHHA	60 kDa heat shock protein, mitochondrial OS=Ophiophagus	OPHHA	46
38	58.4	58.4	79.66	50.75	50.75	tr|V8NZT7|V8NZT7_OPHHA	Bifunctional purine biosynthesis protein PURH OS=Ophiop	OPHHA	41
39	57.79	57.79	85.07	76.8	76	tr|V8PI67|V8PI67_OPHHA	Actin, cytoplasmic 2 OS=Ophiophagus hannah GN=ACTG1 PE=	OPHHA	47
39	0	57.79	83.61	80	79.17	tr|V8N8G6|V8N8G6_OPHHA	Uncharacterized protein (Fragment) OS=Ophiophagus hanna	OPHHA	47
40	57.72	57.72	50.79	14.09	12.97	tr|V8P7Y8|V8P7Y8_OPHHA	Collagen alpha-2(VI) chain (Fragment) OS=Ophiophagus ha	OPHHA	39
41	56.54	56.54	58.95	43.35	34.16	tr|V8NGV0|V8NGV0_OPHHA	Regulator of nonsense transcripts 1 (Fragment) OS=Ophio	OPHHA	27
42	55.46	55.46	56.26	37.87	29.55	tr|V8NZD8|V8NZD8_OPHHA	Sodium/potassium-transporting ATPase subunit alpha OS=O	OPHHA	29
43	55.15	55.15	68.75	52.13	50.27	tr|V8P2P2|V8P2P2_OPHHA	Junction plakoglobin OS=Ophiophagus hannah GN=Jup PE=4	OPHHA	33
44	55.03	82.62	52.11	25.33	20.18	tr|V8PCQ7|V8PCQ7_OPHHA	Myosin-11 (Fragment) OS=Ophiophagus hannah GN=MYH11 PE=	OPHHA	51
45	54.96	54.97	69.26	43.77	40.66	tr|V8P8K8|V8P8K8_OPHHA	Protein disulfide-isomerase OS=Ophiophagus hannah GN=P4	OPHHA	37
46	54.73	54.73	56.68	41.02	34.84	tr|V8PAZ8|V8PAZ8_OPHHA	Tensin (Fragment) OS=Ophiophagus hannah GN=TNS PE=4 SV=	OPHHA	29
47	54.58	54.58	68.91	41.45	36.79	tr|V8NA37|V8NA37_OPHHA	Moesin (Fragment) OS=Ophiophagus hannah GN=Msn PE=4 SV=	OPHHA	27
48	53.03	53.05	51.71	25.58	21.14	tr|V8NFW1|V8NFW1_OPHHA	Myosin-Ic (Fragment) OS=Ophiophagus hannah GN=MYO1C PE=	OPHHA	25
49	52.59	52.59	57.95	27.19	21.2	tr|V8NPZ9|V8NPZ9_OPHHA	Plexin-B2 (Fragment) OS=Ophiophagus hannah GN=PLXNB2 PE	OPHHA	25
50	52.13	52.13	50.64	20.12	15.88	tr|V8NA23|V8NA23_OPHHA	Translational activator GCN1 (Fragment) OS=Ophiophagus	OPHHA	26
51	52.07	52.07	76.94	41.48	36.62	tr|V8P9V0|V8P9V0_OPHHA	Heat shock 70 kDa protein 4 (Fragment) OS=Ophiophagus h	OPHHA	25
52	51.95	51.95	66.35	35.21	32.5	tr|V8PEE5|V8PEE5_OPHHA	Kinesin-like protein OS=Ophiophagus hannah GN=KIF5B PE=	OPHHA	28
53	51.35	51.35	51.55	23.32	17.97	tr|V8NYB3|V8NYB3_OPHHA	Bifunctional aminoacyl-tRNA synthetase OS=Ophiophagus h	OPHHA	27
54	51.27	51.27	71.28	41.29	36.1	tr|V8NV43|V8NV43_OPHHA	Transitional endoplasmic reticulum ATPase (Fragment) OS	OPHHA	27
55	50.57	50.57	62.76	35.74	27.03	tr|V8PCN4|V8PCN4_OPHHA	2-oxoglutarate dehydrogenase, mitochondrial OS=Ophiopha	OPHHA	21
56	49.99	49.99	48.65	21.04	16.96	tr|V8NJS6|V8NJS6_OPHHA	Putative ubiquitin carboxyl-terminal hydrolase FAF-X (F	OPHHA	25
57	49.93	49.93	45.69	36.42	34.77	tr|V8P3P1|V8P3P1_OPHHA	Aconitate hydratase, mitochondrial (Fragment) OS=Ophiop	OPHHA	34
58	49.88	49.88	51.02	32.85	23.72	tr|V8NW73|V8NW73_OPHHA	Cullin-associated NEDD8-dissociated protein 1 (Fragment	OPHHA	22
59	48.26	48.38	43.53	17.52	14.96	tr|V8P209|V8P209_OPHHA	Collagen alpha-3(VI) chain (Fragment) OS=Ophiophagus ha	OPHHA	30
60	48.11	48.11	54.51	34.07	31.76	tr|V8P0Q4|V8P0Q4_OPHHA	Coatomer subunit beta (Fragment) OS=Ophiophagus hannah	OPHHA	27
61	47.84	47.84	71.3	22.93	19.97	tr|V8NRT5|V8NRT5_OPHHA	Early endosome antigen 1 (Fragment) OS=Ophiophagus hann	OPHHA	25
62	47.62	47.62	55.02	21.8	17.06	tr|V8NPB1|V8NPB1_OPHHA	Serine/threonine-protein kinase MRCK beta (Fragment) OS	OPHHA	24
63	47.57	57.36	65.96	21.25	16.8	tr|V8PFR6|V8PFR6_OPHHA	Spectrin beta chain, erythrocyte (Fragment) OS=Ophiopha	OPHHA	30
64	47.24	47.24	54.87	21.48	14.47	tr|V8NRQ6|V8NRQ6_OPHHA	Kinesin-like protein (Fragment) OS=Ophiophagus hannah G	OPHHA	21
65	46.82	46.82	58.38	23.4	18.58	tr|V8NNH1|V8NNH1_OPHHA	Brefeldin A-inhibited guanine nucleotide-exchange prote	OPHHA	23
66	46.69	46.69	77.11	37.19	30.79	tr|V8N8J0|V8N8J0_OPHHA	Elongation factor 2 (Fragment) OS=Ophiophagus hannah GN	OPHHA	25
67	45.69	45.69	73.75	43.87	39.38	tr|V8NWK2|V8NWK2_OPHHA	T-complex protein 1 subunit epsilon (Fragment) OS=Ophio	OPHHA	21
68	44.87	44.87	78.98	68.18	62.5	tr|V8NY29|V8NY29_OPHHA	Fructose-bisphosphate aldolase OS=Ophiophagus hannah GN	OPHHA	36
69	44.83	44.83	70.46	49.32	44.99	tr|V8PAD8|V8PAD8_OPHHA	Phosphoserine aminotransferase OS=Ophiophagus hannah GN	OPHHA	33
70	44.11	44.11	53.52	29.05	27.05	tr|V8P459|V8P459_OPHHA	Constitutive coactivator of PPAR-gamma-like protein 1-l	OPHHA	25
71	43.68	43.68	70.6	51.81	43.86	tr|V8NM39|V8NM39_OPHHA	Isocitrate dehydrogenase [NADP] OS=Ophiophagus hannah G	NADP	26
72	43.4	43.4	70.41	55.81	55.81	tr|V8PIT3|V8PIT3_OPHHA	ATP synthase subunit beta (Fragment) OS=Ophiophagus han	OPHHA	37
73	42.93	42.93	43.78	22.27	19.11	tr|V8NT99|V8NT99_OPHHA	Eukaryotic translation initiation factor 4 gamma 1 (Fra	OPHHA	22
74	42.67	42.67	47.26	17.47	13.15	tr|V8P0S2|V8P0S2_OPHHA	DnaJ-like subfamily C member 13 (Fragment) OS=Ophiophag	OPHHA	21
75	42.56	42.56	74.47	48.36	45.07	tr|V8PBR6|V8PBR6_OPHHA	Serine hydroxymethyltransferase OS=Ophiophagus hannah G	OPHHA	23
76	42.55	66.84	64.14	42.05	38.89	tr|V8NTA5|V8NTA5_OPHHA	Heat shock protein HSP 90-beta (Fragment) OS=Ophiophagu	OPHHA	41
77	42.36	42.36	66.89	21.57	16.78	tr|V8NZ34|V8NZ34_OPHHA	Rho-associated protein kinase 2 (Fragment) OS=Ophiophag	OPHHA	21
78	42.32	42.32	69.31	45.73	37.8	tr|V8PEI7|V8PEI7_OPHHA	Arginyl-tRNA synthetase, cytoplasmic OS=Ophiophagus han	OPHHA	19
79	42.15	42.15	49.7	29.58	28.74	tr|V8P4E7|V8P4E7_OPHHA	Aconitate hydratase (Fragment) OS=Ophiophagus hannah GN	OPHHA	24
80	42.14	42.14	64.19	31.88	23.69	tr|V8NSK8|V8NSK8_OPHHA	Golgi apparatus protein 1 (Fragment) OS=Ophiophagus han	OPHHA	20
81	42.09	42.09	66.94	37.57	29.64	tr|V8PBT8|V8PBT8_OPHHA	ATP-dependent RNA helicase DDX1 OS=Ophiophagus hannah G	OPHHA	21
82	42.09	42.09	71.8	52.63	45.11	tr|V8PGM9|V8PGM9_OPHHA	Aspartyl-tRNA synthetase, cytoplasmic (Fragment) OS=Oph	OPHHA	22
83	42.02	42.02	49.79	23.11	18.27	tr|V8NM58|V8NM58_OPHHA	Xanthine dehydrogenase/oxidase (Fragment) OS=Ophiophagu	OPHHA	23
84	41.74	41.74	58.22	42.43	37.83	tr|V8P027|V8P027_OPHHA	Dihydropyrimidinase-related protein 3 OS=Ophiophagus ha	OPHHA	26
85	41.36	41.36	45.99	16.94	16.94	tr|V8PF84|V8PF84_OPHHA	Eukaryotic translation initiation factor 3 subunit C OS	OPHHA	22
86	41.35	41.35	57.93	52.18	50.11	tr|V8PB80|V8PB80_OPHHA	Tubulin beta-4B chain (Fragment) OS=Ophiophagus hannah	OPHHA	33
87	41.27	41.27	59.71	24.59	22.17	tr|V8PCJ0|V8PCJ0_OPHHA	Aldehyde dehydrogenase, mitochondrial OS=Ophiophagus ha	OPHHA	32
88	41.09	41.09	64.29	26.85	22.33	tr|V8P063|V8P063_OPHHA	Structural maintenance of chromosomes protein 3 (Fragme	OPHHA	19
89	41.01	41.01	49.02	27.81	24.3	tr|V8NQ95|V8NQ95_OPHHA	Exportin-2 OS=Ophiophagus hannah GN=CSE1L PE=4 SV=1	OPHHA	20
90	41.01	41.01	71.21	62.12	60.61	tr|V8PB36|V8PB36_OPHHA	Elongation factor 1-alpha OS=Ophiophagus hannah GN=EEF1	OPHHA	26
91	40.99	40.99	58.92	26.28	24.19	tr|V8P6I6|V8P6I6_OPHHA	Phosphoribosylformylglycinamidine synthase (Fragment) O	OPHHA	26
92	40.97	42.7	67.63	42.19	42.19	tr|V8NVN2|V8NVN2_OPHHA	Isocitrate dehydrogenase [NADP], mitochondrial (Fragmen	NADP	25
93	40.31	40.31	80.15	42.99	39.71	tr|V8PJ35|V8PJ35_OPHHA	Polyadenylate-binding protein (Fragment) OS=Ophiophagus	OPHHA	20
94	40.29	40.29	68.73	44.57	44.57	tr|V8NL59|V8NL59_OPHHA	T-complex protein 1 subunit beta (Fragment) OS=Ophiopha	OPHHA	20

95	40.12	40.12	66.42	38.02	36.54	tr|V8NVZ0|V8NVZ0_OPHHA	Multifunctional protein ADE2 (Fragment) OS=Ophiophagus	OPHHA	24
96	40.06	40.06	76.44	24.73	16.54	tr|V8PGR2|V8PGR2_OPHHA	Structural maintenance of chromosomes protein 1A (Fragm	OPHHA	17
97	39.95	39.95	54.95	39.59	34.13	tr|V8NUY6|V8NUY6_OPHHA	Histidine ammonia-lyase OS=Ophiophagus hannah GN=HAL PE	OPHHA	23
98	39.88	41.48	54.02	17.28	17.18	tr|V8N7V6|V8N7V6_OPHHA	Cytochrome protein (Fragment) OS=Ophiophagus hannah GN=	OPHHA	28
99	39.66	39.66	59.02	38.2	34.59	tr|V8PEL6|V8PEL6_OPHHA	Methylmalonate-semialdehyde dehydrogenase [acylating],	OPHHA	25
100	39.55	39.55	71.61	45.34	45.34	tr|V8PG19|V8PG19_OPHHA	T-complex protein 1 subunit theta OS=Ophiophagus hannah	OPHHA	22
101	39.35	39.35	57.53	27.19	21.66	tr|V8NN65|V8NN65_OPHHA	Splicing factor 3B subunit 1 OS=Ophiophagus hannah GN=S	OPHHA	19
102	39.29	39.29	49.53	25.99	25.06	tr|V8P6J4|V8P6J4_OPHHA	Microsomal triglyceride transfer protein large subunit	OPHHA	21
103	38.9	38.9	54.14	30.81	29.09	tr|V8NFY9|V8NFY9_OPHHA	Ankyrin repeat and FYVE domain-containing protein 1 (Fr	OPHHA	21
104	38.85	38.85	66.48	33.52	33.52	tr|V8NCG3|V8NCG3_OPHHA	Heterogeneous nuclear ribonucleoprotein U-like protein	OPHHA	22
105	38.73	38.73	56.79	33.77	30.94	tr|V8P1G2|V8P1G2_OPHHA	Catalase (Fragment) OS=Ophiophagus hannah GN=CAT PE=3 S	OPHHA	26
106	38.69	38.69	51	26.62	24.85	tr|V8NZS0|V8NZS0_OPHHA	Phenylalanine-4-hydroxylase (Fragment) OS=Ophiophagus h	OPHHA	21
107	38.66	38.66	68.91	47.72	38.61	tr|V8PBL7|V8PBL7_OPHHA	tRNA-splicing ligase RtcB homolog OS=Ophiophagus hannah	OPHHA	20
108	38.19	38.19	74.39	31.76	31.35	tr|V8NZ73|V8NZ73_OPHHA	Vimentin (Fragment) OS=Ophiophagus hannah GN=VIM PE=3 S	OPHHA	25
109	38.14	38.14	68.18	31.17	28.05	tr|V8PBQ3|V8PBQ3_OPHHA	Propionyl-CoA carboxylase alpha chain, mitochondrial (F	OPHHA	19
110	38.04	38.04	67.43	41.59	33.98	tr|V8NQ25|V8NQ25_OPHHA	T-complex protein 1 subunit alpha (Fragment) OS=Ophioph	OPHHA	18
111	37.52	37.52	59.07	45.36	43.75	tr|V8NJB3|V8NJB3_OPHHA	Calcium-binding mitochondrial carrier protein Aralar2 (	OPHHA	23
112	37.3	37.3	60.53	38.28	35.16	tr|V8NM20|V8NM20_OPHHA	GMP synthase [glutamine-hydrolyzing] (Fragment) OS=Ophi	OPHHA	18
113	37.27	37.27	38.13	22.74	18.57	tr|V8PG50|V8PG50_OPHHA	NAD(P) transhydrogenase, mitochondrial (Fragment) OS=Op	OPHHA	23
114	37.24	37.24	80.19	55.56	50.97	tr|V8P7W6|V8P7W6_OPHHA	26S protease regulatory subunit 8 (Fragment) OS=Ophioph	OPHHA	20
115	36.96	36.96	69.29	59.76	54.52	tr|V8PAY9|V8PAY9_OPHHA	2-amino-3-ketobutyrate coenzyme A ligase, mitochondrial	OPHHA	20
116	36.94	36.94	72.93	47.6	40.39	tr|V8P2N0|V8P2N0_OPHHA	Testin (Fragment) OS=Ophiophagus hannah GN=TES PE=4 SV=	OPHHA	17
117	36.91	36.91	58.15	24.73	22.8	tr|V8PJB1|V8PJB1_OPHHA	Pleckstrin-likey domain-containing family A member 6 (F	OPHHA	19
118	36.88	36.88	56.47	30.01	23.96	tr|V8P8G9|V8P8G9_OPHHA	Alpha-1,4 glucan phosphorylase (Fragment) OS=Ophiophagu	OPHHA	18
119	36.78	36.78	56.7	37.98	33.58	tr|V8PA16|V8PA16_OPHHA	Stress-70 protein, mitochondrial OS=Ophiophagus hannah	OPHHA	20
120	36.73	36.73	60.14	45.98	39.69	tr|V8NN23|V8NN23_OPHHA	Serine/threonine-protein phosphatase 2A 65 kDa regulato	OPHHA	18
121	36.67	36.67	53.06	32.6	24.74	tr|V8NJ70|V8NJ70_OPHHA	Dynamin-1 OS=Ophiophagus hannah GN=Dnm1 PE=3 SV=1	OPHHA	18
122	36.48	36.48	57.06	19.35	17.14	tr|V8P6A0|V8P6A0_OPHHA	Cytoplasmic FMR1-interacting protein 1 OS=Ophiophagus h	OPHHA	19
123	36.24	36.24	65.77	51.21	46.63	tr|V8NVK0|V8NVK0_OPHHA	Amidophosphoribosyltransferase (Fragment) OS=Ophiophagu	OPHHA	21
124	36.04	36.04	42.81	18.69	18.69	tr|V8PEY3|V8PEY3_OPHHA	Serine hydroxymethyltransferase (Fragment) OS=Ophiophag	OPHHA	27
125	35.9	35.9	61.22	25.94	25.06	tr|V8PIR9|V8PIR9_OPHHA	26S protease regulatory subunit 6A (Fragment) OS=Ophiop	OPHHA	21
126	35.9	35.9	54.71	33	25.72	tr|V8NRN1|V8NRN1_OPHHA	NADH-ubiquinone oxidoreductase 75 kDa subunit, mitochon	OPHHA	17
127	35.89	35.9	68.62	40.27	38.09	tr|V8PDE6|V8PDE6_OPHHA	Plastin-3 OS=Ophiophagus hannah GN=PLS3 PE=4 SV=1	OPHHA	21
128	35.87	35.94	56.25	36.86	34.78	tr|V8NIS2|V8NIS2_OPHHA	Dolichyl-diphosphooligosaccharide--protein glycosyltran	OPHHA	21
129	35.85	35.85	75.24	36.29	33.08	tr|V8N601|V8N601_OPHHA	Tyrosine--tRNA ligase (Fragment) OS=Ophiophagus hannah	OPHHA	18
130	35.73	35.73	68.02	24.87	22.17	tr|V8P0I5|V8P0I5_OPHHA	Lamin-B1 OS=Ophiophagus hannah GN=Lmnb1 PE=3 SV=1	OPHHA	20
131	35.51	35.51	53.65	35.21	33.43	tr|V8P7D2|V8P7D2_OPHHA	Signal transducer and activator of transcription (Fragm	OPHHA	19
132	35.47	35.47	63.7	42.79	38.7	tr|V8PGR3|V8PGR3_OPHHA	Calreticulin OS=Ophiophagus hannah GN=CALR PE=3 SV=1	OPHHA	26
133	35.09	35.09	85.01	53.75	53.75	tr|V8PFW0|V8PFW0_OPHHA	Actin-related protein 3 OS=Ophiophagus hannah GN=ACTR3	OPHHA	21
134	35.03	35.03	62.2	19.35	13.79	tr|V8PCT1|V8PCT1_OPHHA	Afadin (Fragment) OS=Ophiophagus hannah GN=Mllt4 PE=4 S	OPHHA	17
135	35.01	35.03	82.11	63.16	62.11	tr|V8NI54|V8NI54_OPHHA	Glutamate dehydrogenase 1, mitochondrial (Fragment) OS=	OPHHA	27
136	34.93	34.93	79.09	62.36	56.27	tr|V8P745|V8P745_OPHHA	40S ribosomal protein S4 OS=Ophiophagus hannah GN=RPS4	OPHHA	19
137	34.79	34.79	62.17	40.78	36.9	tr|V8P708|V8P708_OPHHA	Far upstream element-binding protein 1 OS=Ophiophagus h	OPHHA	19
138	34.66	34.66	48.7	29.42	26.38	tr|V8NSB9|V8NSB9_OPHHA	NADPH-cytochrome reductase (Fragment) OS=Ophiophagus ha	OPHHA	19
139	34.66	34.66	45.84	33.5	28.46	tr|V8NUS4|V8NUS4_OPHHA	Coatomer subunit gamma OS=Ophiophagus hannah GN=COPG PE	OPHHA	17
140	34.65	34.65	41.44	21.55	18.88	tr|V8P5Z1|V8P5Z1_OPHHA	Nidogen-1 (Fragment) OS=Ophiophagus hannah GN=Nid1 PE=4	OPHHA	19
141	34.5	34.5	56.03	45.39	43.74	tr|V8NG04|V8NG04_OPHHA	ATP synthase subunit alpha OS=Ophiophagus hannah GN=Atp	OPHHA	24
142	34.23	34.23	44.77	19.09	17.33	tr|V8NB12|V8NB12_OPHHA	Collagen alpha-6(VI) chain (Fragment) OS=Ophiophagus ha	OPHHA	20
143	34.18	34.18	67.71	52.12	52.12	tr|V8NFX8|V8NFX8_OPHHA	Septin-7 (Fragment) OS=Ophiophagus hannah GN=sept7 PE=3	OPHHA	21
144	34.13	34.13	58.55	28.25	22.29	tr|V8PEC5|V8PEC5_OPHHA	Tight junction protein ZO-1 (Fragment) OS=Ophiophagus h	OPHHA	17
145	34	34	62.18	31.49	28.48	tr|V8NZ67|V8NZ67_OPHHA	Vesicle-fusing ATPase (Fragment) OS=Ophiophagus hannah	OPHHA	17
146	33.75	33.98	69.92	35.48	26.35	tr|V8NZL6|V8NZL6_OPHHA	Keratin, type II cytoskeletal 8 (Fragment) OS=Ophiophag	OPHHA	26
147	33.54	33.54	56.04	23.29	21.26	tr|V8P238|V8P238_OPHHA	Sorbin and SH3 domain-containing protein 1 (Fragment) O	OPHHA	17
148	33.42	33.71	55.31	29.66	29.66	tr|V8NNT3|V8NNT3_OPHHA	Hydroxymethylglutaryl-CoA synthase 1 (Fragment) OS=Ophi	OPHHA	22
149	33.36	33.36	49.04	22.74	16.67	tr|V8NXJ3|V8NXJ3_OPHHA	Isoleucyl-tRNA synthetase, mitochondrial (Fragment) OS=	OPHHA	14
150	33.25	33.25	79.35	54.84	47.53	tr|V8P794|V8P794_OPHHA	NADH dehydrogenase [ubiquinone] flavoprotein 1, mitocho	OPHHA	17
151	33.25	33.25	92.55	68.24	63.53	tr|V8P1F0|V8P1F0_OPHHA	Phosphoglycerate mutase 1 OS=Ophiophagus hannah GN=Pgam	OPHHA	25
152	33.24	33.24	40.75	12.05	10.76	tr|V8NW16|V8NW16_OPHHA	Long-chain specific acyl-CoA dehydrogenase, mitochondri	OPHHA	22
153	33.2	33.2	62.84	33.65	30.94	tr|V8NX18|V8NX18_OPHHA	Mitochondrial import receptor subunit TOM70 (Fragment)	OPHHA	18
154	33.12	33.27	53.11	30.49	26.35	tr|V8PG08|V8PG08_OPHHA	Putative ATP-dependent RNA helicase DDX17 OS=Ophiophagu	OPHHA	16
155	32.92	32.92	42.36	21.89	19.45	tr|V8P6U5|V8P6U5_OPHHA	Echinoderm microtubule-associated protein-like 4 (Fragm	OPHHA	17
156	32.87	32.87	58.81	23.12	13.58	tr|V8PJ74|V8PJ74_OPHHA	Pleckstrin-likey domain-containing family A member 7 (F	OPHHA	17
157	32.87	32.87	59.08	21.8	18.69	tr|V8NZ52|V8NZ52_OPHHA	Scavenger receptor cysteine-rich type 1 protein (Fragme	OPHHA	20
158	32.85	32.85	66.46	31.99	30.91	tr|V8P1J3|V8P1J3_OPHHA	Chaperone activity of bc1 complex-like, mitochondrial O	OPHHA	19
159	32.78	32.78	56.3	26.54	20.74	tr|V8NEA9|V8NEA9_OPHHA	Programmed cell death 6-interacting protein OS=Ophiopha	OPHHA	15
160	32.68	32.68	41.27	32.45	30.86	tr|V8N861|V8N861_OPHHA	Aminopeptidase B (Fragment) OS=Ophiophagus hannah GN=Rn	OPHHA	19
161	32.64	34.8	60.54	34.95	28.65	tr|V8NEW1|V8NEW1_OPHHA	Plastin-1 (Fragment) OS=Ophiophagus hannah GN=PLS1 PE=4	OPHHA	18
162	32.51	40.6	76.55	40.28	38.48	tr|V8PDF5|V8PDF5_OPHHA	Retinal dehydrogenase 1 OS=Ophiophagus hannah GN=ALDH1A	OPHHA	26
163	32.49	32.49	55.74	32.91	29.59	tr|V8NJM9|V8NJM9_OPHHA	Long-chain-fatty-acid--CoA ligase 3 (Fragment) OS=Ophio	OPHHA	17
164	32.36	32.36	48.05	27.62	26.9	tr|V8NXA4|V8NXA4_OPHHA	Importin-5 OS=Ophiophagus hannah GN=IPO5 PE=4 SV=1	OPHHA	17
165	32.33	32.33	58.01	32.21	29.89	tr|V8NM08|V8NM08_OPHHA	D-3-phosphoglycerate dehydrogenase OS=Ophiophagus hanna	OPHHA	27
166	32.18	32.18	60.98	37.76	30.81	tr|V8P6Z5|V8P6Z5_OPHHA	Heterogeneous nuclear ribonucleoprotein R OS=Ophiophagu	OPHHA	15
167	32.12	32.12	58.86	42.23	40.05	tr|V8P4Q6|V8P4Q6_OPHHA	Proliferation-associated protein 2G4 OS=Ophiophagus han	OPHHA	17
168	32.09	32.09	42.23	17.25	9.865	tr|V8NHS8|V8NHS8_OPHHA	RNA-binding protein 12 OS=Ophiophagus hannah GN=RBM12 P	OPHHA	14
169	31.89	31.89	84.84	51.62	51.62	tr|V8NHD0|V8NHD0_OPHHA	Enolase (Fragment) OS=Ophiophagus hannah GN=L345_12783	OPHHA	20
170	31.88	31.88	74.71	54.86	54.86	tr|V8NXN9|V8NXN9_OPHHA	Electron transfer flavoprotein subunit beta OS=Ophiopha	OPHHA	19
171	31.78	31.78	50.12	26.39	18.4	tr|V8ND82|V8ND82_OPHHA	Interleukin enhancer-binding factor 3 OS=Ophiophagus ha	OPHHA	17
172	31.75	32.15	72.49	37.4	24.53	tr|V8PI27|V8PI27_OPHHA	Ribosomal protein S6 kinase 2 alpha (Fragment) OS=Ophio	OPHHA	14
173	31.71	33.15	61.47	31.29	25.73	tr|V8PB84|V8PB84_OPHHA	Far upstream element-binding protein 2 (Fragment) OS=Op	OPHHA	18
174	31.71	31.71	60.91	30.39	26.39	tr|V8PE42|V8PE42_OPHHA	Alpha-aminoadipic semialdehyde synthase, mitochondrial	OPHHA	18
175	31.5	38.78	72.05	55.89	45.75	tr|V8NZI8|V8NZI8_OPHHA	Fructose-bisphosphate aldolase (Fragment) OS=Ophiophagu	OPHHA	23
176	31.45	31.45	51.45	32	28.97	tr|V8NQZ2|V8NQZ2_OPHHA	Protein transport protein Sec23A OS=Ophiophagus hannah	OPHHA	16
177	31.38	31.38	64.89	24.71	18.47	tr|V8NHN0|V8NHN0_OPHHA	Amine sulfotransferase (Fragment) OS=Ophiophagus hannah	OPHHA	22
178	31.26	31.26	71.13	44.57	41.8	tr|V8ND58|V8ND58_OPHHA	AP-2 complex subunit mu-1 OS=Ophiophagus hannah GN=AP2M	OPHHA	19
179	31.2	31.2	48.79	37.74	31.13	tr|V8PA47|V8PA47_OPHHA	Coatomer subunit beta OS=Ophiophagus hannah GN=Copb2 PE	OPHHA	18
180	31.13	31.13	59.7	29.04	27.03	tr|V8NEY3|V8NEY3_OPHHA	Alanyl-tRNA synthetase, cytoplasmic (Fragment) OS=Ophio	OPHHA	16
181	31.03	31.03	56.07	52.18	49.51	tr|V8P9N2|V8P9N2_OPHHA	Tubulin alpha-1A chain (Fragment) OS=Ophiophagus hannah	OPHHA	22
182	30.94	30.94	60.35	23.57	23.57	tr|V8NIM4|V8NIM4_OPHHA	Oxysterol-binding protein (Fragment) OS=Ophiophagus han	OPHHA	18
183	30.93	30.93	78.28	44.12	42.53	tr|V8NJF1|V8NJF1_OPHHA	Phosphoglycerate kinase (Fragment) OS=Ophiophagus hanna	OPHHA	21
184	30.88	30.88	79.95	51.05	44.06	tr|V8NRT1|V8NRT1_OPHHA	Splicing factor, proline-and glutamine-rich OS=Ophiopha	OPHHA	14
185	30.85	31.18	56.98	31.01	25	tr|V8NIH1|V8NIH1_OPHHA	WD repeat-containing protein 11 (Fragment) OS=Ophiophag	OPHHA	16
186	30.85	30.85	46.65	26.67	22.49	tr|V8NY92|V8NY92_OPHHA	Oxidation resistance protein 1 (Fragment) OS=Ophiophagu	OPHHA	14
187	30.82	30.82	62.79	40.39	34.22	tr|V8NVQ8|V8NVQ8_OPHHA	Phosphoglucomutase-like protein 5 (Fragment) OS=Ophioph	OPHHA	15
188	30.8	30.8	56.01	29.25	24.49	tr|V8NYU2|V8NYU2_OPHHA	Keratin, type I cytoskeletal 18 (Fragment) OS=Ophiophag	OPHHA	19
189	30.71	30.71	47.59	35.31	33.77	tr|V8P3W3|V8P3W3_OPHHA	Elongation factor Tu OS=Ophiophagus hannah GN=TUFM PE=3	OPHHA	18
190	30.7	30.7	74.19	50	50	tr|V8NTY1|V8NTY1_OPHHA	Malate dehydrogenase (Fragment) OS=Ophiophagus hannah G	OPHHA	19

191	30.66	39.17	56.67	33.51	25.65	tr|V8NBZ7|V8NBZ7_OPHHA	Catenin beta-1 (Fragment) OS=Ophiophagus hannah GN=CTNN	OPHHA	21
192	30.66	30.66	52.74	22.03	19.23	tr|V8NXH8|V8NXH8_OPHHA	Matrin-3 (Fragment) OS=Ophiophagus hannah GN=MATR3 PE=4	OPHHA	15
193	30.57	30.57	58.56	22.01	16.85	tr|V8NYV7|V8NYV7_OPHHA	Leucyl-tRNA synthetase, cytoplasmic OS=Ophiophagus hann	OPHHA	15
194	30.54	30.54	41.74	14.21	12.6	tr|V8NF26|V8NF26_OPHHA	Cytochrome protein (Fragment) OS=Ophiophagus hannah GN=	OPHHA	20
195	30.53	30.53	56.44	39.33	34	tr|V8NQK3|V8NQK3_OPHHA	Sulfide:quinone oxidoreductase, mitochondrial OS=Ophiop	OPHHA	18
196	30.51	30.51	43.05	16.65	12.95	tr|V8NN56|V8NN56_OPHHA	Transmembrane protein 2 (Fragment) OS=Ophiophagus hanna	OPHHA	14
197	30.32	30.32	49.45	24.24	24.24	tr|V8NI87|V8NI87_OPHHA	Peroxisomal multifunctional enzyme type 2 OS=Ophiophagu	OPHHA	22
198	30.28	36.43	56.77	27.71	20.32	tr|V8NE95|V8NE95_OPHHA	Ras GTPase-activating-like protein IQGAP1 (Fragment) OS	OPHHA	16
199	30.22	30.22	75.3	47.89	43.67	tr|V8P6K5|V8P6K5_OPHHA	L-lactate dehydrogenase OS=Ophiophagus hannah GN=LDHA P	OPHHA	19
200	30.16	30.16	54.87	17.37	16.5	tr|V8NPK2|V8NPK2_OPHHA	Caldesmon (Fragment) OS=Ophiophagus hannah GN=CALD1 PE=	OPHHA	17
201	30.12	30.12	68.12	43.32	43.32	tr|V8NIX2|V8NIX2_OPHHA	Flotillin-2 (Fragment) OS=Ophiophagus hannah GN=FLOT2 P	OPHHA	15
202	30.1	30.1	50.09	32.07	27.89	tr|V8P699|V8P699_OPHHA	Calnexin (Fragment) OS=Ophiophagus hannah GN=CANX PE=3	OPHHA	17
203	30.09	30.1	62.58	39.35	36.34	tr|V8NKU8|V8NKU8_OPHHA	Sorting nexin-2 OS=Ophiophagus hannah GN=SNX2 PE=4 SV=1	OPHHA	17
204	29.97	29.97	44.37	15.42	12.72	tr|V8PH64|V8PH64_OPHHA	FAD-dependent oxidoreductase domain-containing protein	OPHHA	15
205	29.96	29.96	75.36	46.13	46.13	tr|V8NCG0|V8NCG0_OPHHA	Peptidyl-prolyl cis-trans isomerase FKBP4 OS=Ophiophagu	OPHHA	17
206	29.78	29.78	83.87	61.29	56.85	tr|V8PDX2|V8PDX2_OPHHA	Triosephosphate isomerase OS=Ophiophagus hannah GN=TPI1	OPHHA	18
207	29.7	29.7	45.64	20.75	16.98	tr|V8NFK2|V8NFK2_OPHHA	DNA damage-binding protein 1 (Fragment) OS=Ophiophagus	OPHHA	14
208	29.6	29.6	50.43	35.58	29.88	tr|V8P0Z6|V8P0Z6_OPHHA	Hydroxyacid oxidase 1 (Fragment) OS=Ophiophagus hannah	OPHHA	16
209	29.52	29.52	63.78	42.6	34.69	tr|V8PF03|V8PF03_OPHHA	Succinate-semialdehyde dehydrogenase, mitochondrial (Fr	OPHHA	16
210	29.51	29.51	50.82	32.08	24.71	tr|V8PCN6|V8PCN6_OPHHA	Ubiquitin carboxyl-terminal hydrolase 5 OS=Ophiophagus	OPHHA	15
211	29.46	29.46	49.61	24.61	18.95	tr|V8NL24|V8NL24_OPHHA	Ubiquitin carboxyl-terminal hydrolase 7 (Fragment) OS=O	OPHHA	14
212	29.13	29.13	48.07	28.04	27.89	tr|V8P4I6|V8P4I6_OPHHA	Prolyl endopeptidase (Fragment) OS=Ophiophagus hannah G	OPHHA	14
213	29.08	29.08	65.67	29.85	25.37	tr|V8NVZ1|V8NVZ1_OPHHA	Amine oxidase [flavin-containing] A (Fragment) OS=Ophio	OPHHA	16
214	28.91	28.91	48.56	28.6	27.24	tr|V8P4J6|V8P4J6_OPHHA	Acyl-CoA synthetase short-chain family member 3, mitoch	OPHHA	15
215	28.84	28.84	48.95	18.96	14.57	tr|V8NV23|V8NV23_OPHHA	Eukaryotic translation initiation factor 5B (Fragment)	OPHHA	13
216	28.82	28.82	60.06	34.91	29.72	tr|V8NZJ3|V8NZJ3_OPHHA	BetA (Fragment) OS=Ophiophagus hannah GN=betA PE=3 SV=1	OPHHA	15
217	28.62	28.62	73.19	28.4	24.87	tr|V8P7H8|V8P7H8_OPHHA	Cytoskeleton-associated protein 4 (Fragment) OS=Ophioph	OPHHA	23
218	28.49	28.49	45.22	30.82	26.24	tr|V8P7P9|V8P7P9_OPHHA	Signal transducer and activator of transcription (Fragm	OPHHA	14
219	28.31	28.31	69.87	29.99	28.94	tr|V8NU45|V8NU45_OPHHA	Succinate dehydrogenase (quinone) (Fragment) OS=Ophioph	OPHHA	15
220	28.18	28.18	52.77	25.44	21.41	tr|V8PC38|V8PC38_OPHHA	Extended synaptotagmin-1 (Fragment) OS=Ophiophagus hann	OPHHA	14
221	28.17	28.17	41.21	20.21	18.85	tr|V8P0N6|V8P0N6_OPHHA	Leukocyte elastase inhibitor (Fragment) OS=Ophiophagus	OPHHA	18
222	28.06	28.06	46.46	25.63	22.83	tr|V8PE44|V8PE44_OPHHA	Methylmalonyl-CoA mutase, mitochondrial OS=Ophiophagus	OPHHA	14
223	28.03	28.03	53.78	21.11	18.97	tr|V8NL23|V8NL23_OPHHA	MICOS complex subunit MIC60 OS=Ophiophagus hannah GN=Im	OPHHA	15
224	28	28.37	54.06	21.24	15.6	tr|V8PEZ1|V8PEZ1_OPHHA	Lon protease homolog (Fragment) OS=Ophiophagus hannah G	OPHHA	13
225	27.92	27.92	47.43	35.98	35.75	tr|V8NWT5|V8NWT5_OPHHA	Saccharopine dehydrogenase-like oxidoreductase OS=Ophio	OPHHA	15
226	27.91	27.91	68.25	49.5	41.5	tr|V8NFD5|V8NFD5_OPHHA	Guanine nucleotide-binding protein subunit beta-2-like	OPHHA	17
227	27.84	27.84	45.34	7.808	6.655	tr|V8N8U7|V8N8U7_OPHHA	Uncharacterized protein (Fragment) OS=Ophiophagus hanna	OPHHA	17
228	27.79	27.79	48.5	33.88	30.74	tr|V8NB65|V8NB65_OPHHA	26S proteasome non-ATPase regulatory subunit 2 OS=Ophio	OPHHA	15
229	27.74	27.74	54.32	23.13	23.13	tr|V8NYG0|V8NYG0_OPHHA	Major vault protein alpha (Fragment) OS=Ophiophagus han	OPHHA	15
230	27.71	29.79	62.21	33.4	31.3	tr|V8N7M5|V8N7M5_OPHHA	T-complex protein 1 subunit delta OS=Ophiophagus hannah	OPHHA	16
231	27.67	27.67	57.43	53.72	53.72	tr|V8NDK0|V8NDK0_OPHHA	40S ribosomal protein SA OS=Ophiophagus hannah GN=RPSA	OPHHA	14
232	27.64	27.64	51.77	29.79	23.12	tr|V8NJX7|V8NJX7_OPHHA	Gamma-adducin OS=Ophiophagus hannah GN=ADD3 PE=4 SV=1	OPHHA	13
233	27.63	27.63	60.51	42.96	34.18	tr|V8PG75|V8PG75_OPHHA	ATP-dependent RNA helicase DDX39A OS=Ophiophagus hannah	OPHHA	13
234	27.58	27.58	71.25	49.52	43.13	tr|V8N6Y4|V8N6Y4_OPHHA	Glycerol-3-phosphate dehydrogenase [NAD(+)] OS=Ophiopha	NAD(+)	16
235	27.56	27.56	61.4	35.59	34.09	tr|V8P718|V8P718_OPHHA	Formimidoyltransferase-cyclodeaminase (Fragment) OS=Oph	OPHHA	20
236	27.32	27.32	86.7	62.23	58.8	tr|V8NDW0|V8NDW0_OPHHA	14-3-3 protein epsilon OS=Ophiophagus hannah GN=YWHAE P	OPHHA	21
237	27.16	27.16	45.51	18.56	14.87	tr|V8NMD0|V8NMD0_OPHHA	SEC23-interacting protein (Fragment) OS=Ophiophagus han	OPHHA	14
238	26.95	26.95	77.21	56.46	53.74	tr|V8NXK1|V8NXK1_OPHHA	Voltage-dependent anion-selective channel protein 3 OS=	OPHHA	23
239	26.91	26.91	26.2	11.44	11.44	tr|V8PFH1|V8PFH1_OPHHA	NADH-cytochrome b5 reductase 2 (Fragment) OS=Ophiophagu	OPHHA	18
240	26.89	48.97	53.66	34.45	27.85	tr|V8PAD6|V8PAD6_OPHHA	AP-2 complex subunit alpha-2 (Fragment) OS=Ophiophagus	OPHHA	23
241	26.68	26.68	57.65	21.42	21.42	tr|V8NCP4|V8NCP4_OPHHA	Complement C3 (Fragment) OS=Ophiophagus hannah GN=C3 PE	OPHHA	16
242	26.67	26.67	63.27	43.13	32.94	tr|V8P0I6|V8P0I6_OPHHA	26S proteasome non-ATPase regulatory subunit 11 (Fragme	OPHHA	14
243	26.67	26.67	88.89	75.36	66.67	tr|V8NJ44|V8NJ44_OPHHA	Ras-related protein Rab-7a OS=Ophiophagus hannah GN=RAB	OPHHA	14
244	26.62	26.62	83.53	53.41	53.01	tr|V8P5H2|V8P5H2_OPHHA	Proteasome subunit alpha type OS=Ophiophagus hannah GN=	OPHHA	14
245	26.55	26.55	49.58	34.35	34.35	tr|V8PG34|V8PG34_OPHHA	Dehydrogenase/reductase SDR family member 7 (Fragment)	OPHHA	18
246	26.54	26.54	56.42	26.43	21.47	tr|V8PFY6|V8PFY6_OPHHA	Glycine dehydrogenase [decarboxylating], mitochondrial	OPHHA	12
247	26.42	26.42	59.95	44.03	32.89	tr|V8PJ99|V8PJ99_OPHHA	Uncharacterized protein (Fragment) OS=Ophiophagus hanna	OPHHA	12
248	26.4	26.4	44.66	18.65	16.39	tr|V8NHT3|V8NHT3_OPHHA	Aspartyl/asparaginyl beta-hydroxylase (Fragment) OS=Oph	OPHHA	13
249	26.4	26.4	68.35	36.5	33.54	tr|V8P2C8|V8P2C8_OPHHA	Pyruvate kinase OS=Ophiophagus hannah GN=PKM2 PE=3 SV=1	OPHHA	13
250	26.38	26.38	53.56	26.67	23.45	tr|V8NQK7|V8NQK7_OPHHA	Aspartate aminotransferase (Fragment) OS=Ophiophagus ha	OPHHA	16
251	26.36	26.36	27.36	10.71	9.695	tr|V8NZA6|V8NZA6_OPHHA	Host cell factor 1 OS=Ophiophagus hannah GN=HCFC1 PE=4	OPHHA	13
252	26.3	26.3	38.73	10.96	7.416	tr|V8NSQ9|V8NSQ9_OPHHA	CCR4-NOT transcription complex subunit 1 OS=Ophiophagus	OPHHA	14
253	26.27	26.27	48.89	26	19.61	tr|V8PGH4|V8PGH4_OPHHA	Vacuolar protein sorting-associated protein 35 OS=Ophio	OPHHA	14
254	26.27	26.27	63.95	38.84	36.91	tr|V8PBY1|V8PBY1_OPHHA	Protein phosphatase 1B OS=Ophiophagus hannah GN=PPM1B P	OPHHA	17
255	26.27	26.27	78.45	53.13	43.86	tr|V8P3G8|V8P3G8_OPHHA	NAD kinase domain-containing protein 1 (Fragment) OS=Op	OPHHA	15
256	26.23	26.23	60.62	27.23	21.38	tr|V8PGZ0|V8PGZ0_OPHHA	Signal recognition particle 68 kDa protein (Fragment) O	OPHHA	14
257	26.17	26.17	53.53	24.59	16.12	tr|V8NNJ1|V8NNJ1_OPHHA	Transcription intermediary factor 1-beta (Fragment) OS=	OPHHA	11
258	26.07	26.07	34.43	18.94	15.39	tr|V8P7S7|V8P7S7_OPHHA	Protein transport protein Sec24A (Fragment) OS=Ophiopha	OPHHA	13
259	25.96	25.96	66.2	13.35	11.12	tr|V8NQD9|V8NQD9_OPHHA	LIM domain only protein 7 (Fragment) OS=Ophiophagus han	OPHHA	14
260	25.93	25.93	43.9	28.27	23.34	tr|V8NDU4|V8NDU4_OPHHA	Annexin (Fragment) OS=Ophiophagus hannah GN=Anxa11 PE=3	OPHHA	13
261	25.9	25.9	81.55	38.35	38.35	tr|V8PGH6|V8PGH6_OPHHA	Tropomyosin alpha-1 chain (Fragment) OS=Ophiophagus han	OPHHA	15
262	25.85	25.85	41.52	13.29	11.26	tr|V8NK36|V8NK36_OPHHA	Murinoglobulin-2 OS=Ophiophagus hannah GN=Mug2 PE=4 SV=	OPHHA	14
263	25.77	25.77	50.64	33.21	29.58	tr|V8NK80|V8NK80_OPHHA	Apoptosis-inducing factor 1, mitochondrial (Fragment) O	OPHHA	19
264	25.7	25.7	46.36	12.63	11.89	tr|V8PD84|V8PD84_OPHHA	Leucine-rich PPR motif-containing protein, mitochondria	OPHHA	14
265	25.69	25.69	48.59	36.01	27.55	tr|V8P2T3|V8P2T3_OPHHA	Asparaginyl-tRNA synthetase, cytoplasmic OS=Ophiophagus	OPHHA	12
266	25.67	25.67	67.82	50.99	47.03	tr|V8PCC9|V8PCC9_OPHHA	Acetyl-CoA acetyltransferase, mitochondrial OS=Ophiopha	OPHHA	14
267	25.66	25.66	48.64	23.19	23.19	tr|V8NZD0|V8NZD0_OPHHA	Transketolase (Fragment) OS=Ophiophagus hannah GN=Tkt P	OPHHA	16
268	25.59	27.3	58.76	31.96	31.96	tr|V8NWG5|V8NWG5_OPHHA	Aldehyde dehydrogenase family 8 member A1 (Fragment) OS	OPHHA	15
269	25.55	25.96	53.15	23.94	14.24	tr|V8PGP9|V8PGP9_OPHHA	Myosin-Ib (Fragment) OS=Ophiophagus hannah GN=MYO1B PE=	OPHHA	12
270	25.42	25.42	56.07	28.12	24.96	tr|V8NWF0|V8NWF0_OPHHA	Bifunctional 3'-phosphoadenosine 5'-phosphosulfate synt	OPHHA	13
271	25.2	30.48	60.89	44.28	36.9	tr|V8P6L3|V8P6L3_OPHHA	Calcium-binding mitochondrial carrier protein Aralar1 O	OPHHA	14
272	25.2	27.28	74.04	47.3	33.93	tr|V8P8Y6|V8P8Y6_OPHHA	26S protease regulatory subunit 4 OS=Ophiophagus hannah	OPHHA	13
273	25.18	25.18	56.01	19.64	10.68	tr|V8P7Q5|V8P7Q5_OPHHA	GRIP1-associated protein 1 (Fragment) OS=Ophiophagus ha	OPHHA	11
274	25.18	25.18	61.36	31.96	23.3	tr|V8PCY8|V8PCY8_OPHHA	DCC-interacting protein 13-alpha OS=Ophiophagus hannah	OPHHA	11
275	25.11	25.11	72.95	35.7	35.7	tr|V8NJV8|V8NJV8_OPHHA	2-hydroxyacyl-CoA lyase 1 (Fragment) OS=Ophiophagus han	OPHHA	15
276	25.1	25.1	46.68	33.2	31.84	tr|V8NNS8|V8NNS8_OPHHA	Ubiquitin carboxyl-terminal hydrolase 14 OS=Ophiophagus	OPHHA	12
277	25.09	25.09	43.53	8.424	7.765	tr|V8NSH6|V8NSH6_OPHHA	Laminin subunit alpha-2 (Fragment) OS=Ophiophagus hanna	OPHHA	13
278	25.01	25.01	63.27	37.79	36.09	tr|V8P9F7|V8P9F7_OPHHA	Putative aminopeptidase NPEPL1 (Fragment) OS=Ophiophagu	OPHHA	13
279	24.9	24.9	49.39	30.85	25.13	tr|V8NML9|V8NML9_OPHHA	Phenylalanyl-tRNA synthetase beta chain (Fragment) OS=O	OPHHA	11
280	24.86	24.86	58.26	30.02	25.58	tr|V8PAY8|V8PAY8_OPHHA	Eukaryotic translation initiation factor 3 subunit L OS	OPHHA	14
281	24.81	24.81	66.34	29.77	25.49	tr|V8PE54|V8PE54_OPHHA	Seryl-tRNA synthetase, cytoplasmic OS=Ophiophagus hanna	OPHHA	13
282	24.79	24.79	51	20.07	18.56	tr|V8NLN2|V8NLN2_OPHHA	Fructose-1,6-bisphosphatase isozyme 2 (Fragment) OS=Oph	OPHHA	25
283	24.77	24.77	72.28	41.57	41.57	tr|V8NUJ3|V8NUJ3_OPHHA	Inorganic pyrophosphatase (Fragment) OS=Ophiophagus han	OPHHA	14
284	24.73	24.73	53.1	13.56	11.08	tr|V8NNK8|V8NNK8_OPHHA	Nuclear pore complex protein OS=Ophiophagus hannah GN=N	OPHHA	11
285	24.7	24.7	69.4	36.57	34.08	tr|V8NHZ5|V8NHZ5_OPHHA	Sorting nexin-4 OS=Ophiophagus hannah GN=SNX4 PE=4 SV=1	OPHHA	13
286	24.67	24.67	52.4	14.73	12.76	tr|V8P4M7|V8P4M7_OPHHA	Cordon-bleu protein-like 1 (Fragment) OS=Ophiophagus ha	OPHHA	12

287	24.67	24.67	61.17	25.96	24.14	tr|V8P046|V8P046_OPHHA	Fibrinogen beta chain OS=Ophiophagus hannah GN=FGB PE=4	OPHHA	20
288	24.61	24.61	46.93	26.61	21.93	tr|V8P826|V8P826_OPHHA	Acetyl-coenzyme A synthetase 2-like, mitochondrial (Fra	OPHHA	11
289	24.56	24.56	59.91	39.66	29.42	tr|V8P198|V8P198_OPHHA	Programmed cell death protein 4 OS=Ophiophagus hannah G	OPHHA	12
290	24.55	24.55	42.77	24.96	18.69	tr|V8PA91|V8PA91_OPHHA	Acetyl-coenzyme A synthetase OS=Ophiophagus hannah GN=A	OPHHA	10
291	24.54	24.54	74.16	40.55	29.41	tr|V8PFA7|V8PFA7_OPHHA	V-type proton ATPase subunit H OS=Ophiophagus hannah GN	OPHHA	13
292	24.48	24.48	54.98	36.43	35.75	tr|V8NDL7|V8NDL7_OPHHA	V-type proton ATPase subunit B (Fragment) OS=Ophiophagu	OPHHA	13
293	24.27	24.27	67.19	36.75	30.71	tr|V8NNL1|V8NNL1_OPHHA	60S ribosomal protein L4 (Fragment) OS=Ophiophagus hann	OPHHA	14
294	24.25	24.25	75.1	50.59	49.8	tr|V8NXD2|V8NXD2_OPHHA	Uncharacterized protein OS=Ophiophagus hannah GN=L345_0	OPHHA	15
295	24.23	26.51	46.95	22.89	17.22	tr|V8PHH0|V8PHH0_OPHHA	U5 small nuclear ribonucleoprotein component (Fragment)	OPHHA	12
296	24.23	24.23	50.4	29.12	24.7	tr|V8P9H1|V8P9H1_OPHHA	UTP--glucose-1-phosphate uridylyltransferase OS=Ophioph	OPHHA	14
297	24.21	52.72	69.16	20.88	17.94	tr|V8PFG8|V8PFG8_OPHHA	Myosin-10 OS=Ophiophagus hannah GN=MYH10 PE=4 SV=1	OPHHA	29
298	24.07	31.06	73.29	36.06	33.22	tr|V8P075|V8P075_OPHHA	Heterogeneous nuclear ribonucleoprotein Q (Fragment) OS	OPHHA	16
299	24.06	24.06	68	17.27	15.57	tr|V8NPI7|V8NPI7_OPHHA	Collagen alpha-2(I) chain (Fragment) OS=Ophiophagus han	OPHHA	15
300	24.05	24.05	64.73	52.71	52.71	tr|V8NWC0|V8NWC0_OPHHA	Ribose-phosphate pyrophosphokinase 2 (Fragment) OS=Ophi	OPHHA	22
301	23.98	23.98	48.45	24	19.63	tr|V8NG27|V8NG27_OPHHA	Nodal modulator 1 OS=Ophiophagus hannah GN=NOMO1 PE=4 S	OPHHA	11
302	23.94	23.94	62.94	56.35	46.7	tr|V8NWQ9|V8NWQ9_OPHHA	Peroxiredoxin-6 (Fragment) OS=Ophiophagus hannah GN=PRD	OPHHA	11
303	23.85	26.14	82.86	56.33	53.06	tr|V8P7V9|V8P7V9_OPHHA	14-3-3 protein theta OS=Ophiophagus hannah GN=YWHAQ PE=	OPHHA	17
304	23.83	23.83	63.51	11.34	9.026	tr|V8PCL4|V8PCL4_OPHHA	Band 4.1-like protein 1 (Fragment) OS=Ophiophagus hanna	OPHHA	13
305	23.8	23.8	65.4	27.68	23.72	tr|V8P3Y7|V8P3Y7_OPHHA	V-type proton ATPase catalytic subunit A OS=Ophiophagus	OPHHA	15
306	23.73	23.73	55.02	13.08	12.09	tr|V8PE26|V8PE26_OPHHA	Sickle tail protein OS=Ophiophagus hannah GN=Skt PE=4 S	OPHHA	15
307	23.69	23.69	52.8	21.51	17.02	tr|V8NGE4|V8NGE4_OPHHA	Ubiquitin-like modifier-activating enzyme 6 (Fragment)	OPHHA	12
308	23.68	23.68	64.44	40.63	40.63	tr|V8PB09|V8PB09_OPHHA	Peroxiredoxin-4 (Fragment) OS=Ophiophagus hannah GN=Prd	OPHHA	17
309	23.59	23.59	36.59	10.84	8.13	tr|V8NI08|V8NI08_OPHHA	DENN domain-containing protein 4C (Fragment) OS=Ophioph	OPHHA	11
310	23.56	23.56	64.94	59.74	51.3	tr|V8P7S0|V8P7S0_OPHHA	Eukaryotic translation initiation factor 5A OS=Ophiopha	OPHHA	17
311	23.47	23.47	59.72	30.49	27.17	tr|V8PGK1|V8PGK1_OPHHA	Coiled-coil domain-containing protein 22 OS=Ophiophagus	OPHHA	15
312	23.45	23.45	51.08	24.46	22.03	tr|V8NVE6|V8NVE6_OPHHA	6-phosphofructokinase, liver type OS=Ophiophagus hannah	OPHHA	11
313	23.42	23.42	42.39	7.954	6.698	tr|V8P0X6|V8P0X6_OPHHA	Lysine--tRNA ligase OS=Ophiophagus hannah GN=KARS PE=3	OPHHA	15
314	23.4	23.4	59.42	44.81	42.21	tr|V8NX80|V8NX80_OPHHA	3'(2'),5'-bisphosphate nucleotidase 1 OS=Ophiophagus ha	OPHHA	15
315	23.35	26.39	61.9	30.09	22.51	tr|V8NRX1|V8NRX1_OPHHA	Uncharacterized protein (Fragment) OS=Ophiophagus hanna	OPHHA	14
316	23.29	23.29	47.81	29.33	25.71	tr|V8NQJ7|V8NQJ7_OPHHA	Methionyl-tRNA synthetase, cytoplasmic OS=Ophiophagus h	OPHHA	11
317	23.24	23.24	46.32	16.73	13.79	tr|V8P6S8|V8P6S8_OPHHA	Protein transport protein Sec24D (Fragment) OS=Ophiopha	OPHHA	11
318	23.16	26.53	62.82	26.99	25.64	tr|V8NKM4|V8NKM4_OPHHA	ATP-dependent RNA helicase DDX3X OS=Ophiophagus hannah	OPHHA	14
319	23.12	23.12	42.58	16.08	12.72	tr|V8NZF2|V8NZF2_OPHHA	Splicing factor 3B subunit 3 OS=Ophiophagus hannah GN=S	OPHHA	11
320	23.12	23.12	55.82	18.32	15.36	tr|V8NGJ6|V8NGJ6_OPHHA	Hephaestin-like protein 1 OS=Ophiophagus hannah GN=HEPH	OPHHA	15
321	23.07	23.07	34.72	10.78	8.492	tr|V8P7E4|V8P7E4_OPHHA	Protein furry-like-like protein (Fragment) OS=Ophiophag	OPHHA	15
322	23.06	23.06	45.99	20.11	17.97	tr|V8P0U3|V8P0U3_OPHHA	Serine/threonine-protein phosphatase 4 regulatory subun	OPHHA	12
323	23.04	23.04	69.38	38.52	36.54	tr|V8P8K0|V8P8K0_OPHHA	Aminomethyltransferase OS=Ophiophagus hannah GN=AMT PE=	OPHHA	15
324	23.03	23.03	57.43	23.14	21.62	tr|V8NCQ7|V8NCQ7_OPHHA	Methylcrotonoyl-CoA carboxylase subunit alpha, mitochon	OPHHA	13
325	23.02	23.02	59.65	24.56	22.06	tr|V8PBY8|V8PBY8_OPHHA	Vacuolar protein sorting-associated protein 45 OS=Ophio	OPHHA	11
326	23.01	23.01	54.32	12.42	10.72	tr|V8PHD2|V8PHD2_OPHHA	Sulfurtransferase OS=Ophiophagus hannah GN=Tst PE=4 SV=	OPHHA	16
327	22.98	22.98	49.75	19.34	16.79	tr|V8PF80|V8PF80_OPHHA	Exocyst complex component 7 OS=Ophiophagus hannah GN=EX	OPHHA	11
328	22.9	22.9	57.25	15.15	14.31	tr|V8P8H5|V8P8H5_OPHHA	Putative helicase MOV-10 (Fragment) OS=Ophiophagus hann	OPHHA	13
329	22.9	22.9	54.7	16	12.56	tr|V8NY20|V8NY20_OPHHA	ATP-dependent RNA helicase SUPV3L1, mitochondrial (Frag	OPHHA	10
330	22.84	22.84	67.69	21.25	19.21	tr|V8NXB4|V8NXB4_OPHHA	Kinesin light chain 4 (Fragment) OS=Ophiophagus hannah	OPHHA	11
331	22.78	22.78	60.94	36.71	36.71	tr|V8P364|V8P364_OPHHA	Perilipin OS=Ophiophagus hannah GN=PLIN2 PE=3 SV=1	OPHHA	11
332	22.72	22.72	35.23	6.917	6.472	tr|V8P7J4|V8P7J4_OPHHA	Uncharacterized protein (Fragment) OS=Ophiophagus hanna	OPHHA	12
333	22.69	22.69	43.6	23.51	17.26	tr|V8NIE2|V8NIE2_OPHHA	Heterogeneous nuclear ribonucleoprotein U-like protein	OPHHA	11
334	22.59	22.59	62.36	32.1	24.71	tr|V8P5J0|V8P5J0_OPHHA	Eukaryotic translation initiation factor 5 OS=Ophiophag	OPHHA	12
335	22.57	22.57	68.16	30.13	27.78	tr|V8N4L4|V8N4L4_OPHHA	Acyl-CoA dehydrogenase family member 9, mitochondrial (	OPHHA	10
336	22.56	22.56	38.24	16.59	12.68	tr|V8P6M9|V8P6M9_OPHHA	Bile salt export pump (Fragment) OS=Ophiophagus hannah	OPHHA	11
337	22.52	22.52	73.48	53.03	46.97	tr|V8PA97|V8PA97_OPHHA	40S ribosomal protein S3a OS=Ophiophagus hannah GN=RPS3	OPHHA	13
338	22.51	22.51	69.49	29.66	27.4	tr|V8P9B0|V8P9B0_OPHHA	Reticulocalbin-1 (Fragment) OS=Ophiophagus hannah GN=RC	OPHHA	13
339	22.46	22.46	58.1	29.18	26.43	tr|V8P8H9|V8P8H9_OPHHA	Heterogeneous nuclear ribonucleoprotein A1 (Fragment) O	OPHHA	12
340	22.46	22.46	56.16	27.03	27.03	tr|V8NE43|V8NE43_OPHHA	Eukaryotic translation initiation factor 2 subunit 2 OS	OPHHA	14
341	22.45	22.45	58.52	26.51	18.41	tr|V8NMH5|V8NMH5_OPHHA	Cysteinyl-tRNA synthetase, cytoplasmic (Fragment) OS=Op	OPHHA	10
342	22.44	22.44	48.56	33.41	31.01	tr|V8P9Q5|V8P9Q5_OPHHA	Eukaryotic peptide chain release factor subunit 1 (Frag	OPHHA	12
343	22.42	22.42	44.9	18.01	15.26	tr|V8NTN6|V8NTN6_OPHHA	Plasma membrane calcium-transporting ATPase 4 (Fragment	OPHHA	11
344	22.39	22.39	44.68	5.573	3.791	tr|V8P1E2|V8P1E2_OPHHA	Ankyrin-3 (Fragment) OS=Ophiophagus hannah GN=ANK3 PE=4	OPHHA	15
345	22.36	22.36	80.97	39.53	32.98	tr|V8P0E7|V8P0E7_OPHHA	Methyl-CpG-binding protein 2 (Fragment) OS=Ophiophagus	OPHHA	12
346	22.36	22.36	55	19.73	17.57	tr|V8NIG3|V8NIG3_OPHHA	Beta,beta-carotene 9',10'-oxygenase (Fragment) OS=Ophio	OPHHA	15
347	22.28	22.57	45.02	23	14.45	tr|V8NZI7|V8NZI7_OPHHA	Urocanate hydratase (Fragment) OS=Ophiophagus hannah GN	OPHHA	17
348	22.26	22.26	47.14	19.03	15.8	tr|V8NCP1|V8NCP1_OPHHA	Integrin beta OS=Ophiophagus hannah GN=ITGB1 PE=3 SV=1	OPHHA	13
349	22.26	22.26	62.79	41.86	41.86	tr|V8NNK5|V8NNK5_OPHHA	Eukaryotic initiation factor 4A-II OS=Ophiophagus hanna	OPHHA	12
350	22.24	22.24	48.56	26.95	25.51	tr|V8NW58|V8NW58_OPHHA	Leukotriene A-4 hydrolase (Fragment) OS=Ophiophagus han	OPHHA	13
351	22.22	22.22	53.71	14.01	8.204	tr|V8NV83|V8NV83_OPHHA	GRIP and coiled-coil domain-containing protein 2 (Fragm	OPHHA	11
352	22.21	22.21	55.72	14.79	10.71	tr|V8NH83|V8NH83_OPHHA	Dynamin-like protein (Fragment) OS=Ophiophagus hannah G	OPHHA	9
353	22.2	22.2	54.51	30.02	28.73	tr|V8NTJ5|V8NTJ5_OPHHA	Fumarate hydratase, mitochondrial (Fragment) OS=Ophioph	OPHHA	19
354	22.14	22.14	52.29	34.7	26.27	tr|V8PDB7|V8PDB7_OPHHA	Citrate synthase OS=Ophiophagus hannah GN=CS PE=3 SV=1	OPHHA	11
355	22.13	22.13	80.26	66.95	57.08	tr|V8NZZ6|V8NZZ6_OPHHA	40S ribosomal protein S3 (Fragment) OS=Ophiophagus hann	OPHHA	12
356	22	22	67.64	44	44	tr|V8NTR1|V8NTR1_OPHHA	40S ribosomal protein S2 OS=Ophiophagus hannah GN=RPS2	OPHHA	13
357	21.99	21.99	62.37	49.13	47.04	tr|V8PAT0|V8PAT0_OPHHA	Glucosamine-6-phosphate isomerase OS=Ophiophagus hannah	OPHHA	11
358	21.97	21.97	49.1	39.43	39.43	tr|V8PGE9|V8PGE9_OPHHA	Proteasome subunit alpha type (Fragment) OS=Ophiophagus	OPHHA	15
359	21.95	21.95	58.95	21.33	15.19	tr|V8P0G9|V8P0G9_OPHHA	Protein phosphatase 1 regulatory subunit 12A (Fragment)	OPHHA	12
360	21.94	21.94	40.08	24.05	16.03	tr|V8NVU5|V8NVU5_OPHHA	Ubiquitin carboxyl-terminal hydrolase 10 (Fragment) OS=	OPHHA	10
361	21.92	21.92	58.2	25.08	20.16	tr|V8P2R9|V8P2R9_OPHHA	Threonyl-tRNA synthetase, cytoplasmic (Fragment) OS=Oph	OPHHA	11
362	21.91	21.91	47.13	7.583	5.589	tr|V8PC53|V8PC53_OPHHA	RANBP2-like and GRIP domain-containing protein 5/6 (Fra	OPHHA	12
363	21.91	21.91	62.65	32.83	29.52	tr|V8PG26|V8PG26_OPHHA	Glutaredoxin-3 OS=Ophiophagus hannah GN=GLRX3 PE=4 SV=1	OPHHA	11
364	21.87	21.87	57.62	22.98	15	tr|V8PJ82|V8PJ82_OPHHA	Putative ATP-dependent RNA helicase DDX23 (Fragment) OS	OPHHA	11
365	21.83	21.83	63.48	19.5	15.03	tr|V8PBX6|V8PBX6_OPHHA	E3 ubiquitin-protein ligase HECTD3 (Fragment) OS=Ophiop	OPHHA	10
366	21.83	21.83	69.95	24.35	21.93	tr|V8P4U2|V8P4U2_OPHHA	Heterogeneous nuclear ribonucleoprotein U (Fragment) OS	OPHHA	13
367	21.8	21.85	66.23	34.43	24.34	tr|V8NWQ4|V8NWQ4_OPHHA	26S proteasome non-ATPase regulatory subunit 12 (Fragme	OPHHA	11
368	21.76	21.76	45.8	21.09	20.1	tr|V8P9C3|V8P9C3_OPHHA	Carnitine O-palmitoyltransferase 2, mitochondrial (Frag	OPHHA	13
369	21.71	21.71	69.02	34.63	30.73	tr|V8P437|V8P437_OPHHA	Lissencephaly-1 homolog OS=Ophiophagus hannah GN=PAFAH1	OPHHA	11
370	21.7	21.7	48.79	31.35	30.68	tr|V8NLC9|V8NLC9_OPHHA	Actin-related protein 2 (Fragment) OS=Ophiophagus hanna	OPHHA	17
371	21.7	21.7	83.5	54.05	41.1	tr|V8NKD1|V8NKD1_OPHHA	Serine/threonine-protein phosphatase OS=Ophiophagus han	OPHHA	11
371	0	19.61	82.61	55.8	41.3	tr|V8P7S2|V8P7S2_OPHHA	Serine/threonine-protein phosphatase (Fragment) OS=Ophi	OPHHA	10
372	21.68	21.68	45.6	12.36	10.14	tr|V8PB91|V8PB91_OPHHA	Ubiquitin carboxyl-terminal hydrolase 47 (Fragment) OS=	OPHHA	12
373	21.67	21.67	58.03	26.83	20.9	tr|V8NDQ0|V8NDQ0_OPHHA	Actin-related protein 2/3 complex subunit 1B (Fragment)	OPHHA	12
374	21.55	21.6	49.84	11.07	10.42	tr|V8NQG3|V8NQG3_OPHHA	Sorbin and SH3 domain-containing protein 2 (Fragment) O	OPHHA	11
375	21.55	21.55	46.12	17.17	16	tr|V8PG17|V8PG17_OPHHA	Fibronectin type III domain-containing protein 3B OS=Op	OPHHA	12
376	21.5	21.5	70.94	57.81	49.38	tr|V8PBR7|V8PBR7_OPHHA	Protein SEC13-like protein OS=Ophiophagus hannah GN=Sec	OPHHA	12
377	21.49	21.5	57.8	47.98	40.17	tr|V8N6N2|V8N6N2_OPHHA	26S proteasome non-ATPase regulatory subunit 13 (Fragme	OPHHA	10
378	21.45	21.52	73.66	43.89	38.17	tr|V8P8W0|V8P8W0_OPHHA	Annexin (Fragment) OS=Ophiophagus hannah GN=ANXA5 PE=3	OPHHA	11
379	21.43	21.43	43.5	12.84	8.784	tr|V8P298|V8P298_OPHHA	Tripeptidyl-peptidase 2 (Fragment) OS=Ophiophagus hanna	OPHHA	10
380	21.43	21.43	50.11	20.59	18.76	tr|V8NMW3|V8NMW3_OPHHA	Non-specific lipid-transfer protein OS=Ophiophagus hann	OPHHA	17
381	21.4	21.4	51.13	39.8	30.73	tr|V8NPL9|V8NPL9_OPHHA	S-adenosylmethionine synthase (Fragment) OS=Ophiophagus	OPHHA	12

382	21.4	21.4	58.75	43.89	40.92	tr|V8P0L1|V8P0L1_OPHHA	Guanine nucleotide-binding protein G(I) subunit alpha-2	OPHHA	14
383	21.31	21.31	55.28	17.61	13.45	tr|V8P931|V8P931_OPHHA	Presequence protease, mitochondrial (Fragment) OS=Ophio	OPHHA	9
384	21.25	21.25	54.73	33.85	29.89	tr|V8NU21|V8NU21_OPHHA	Glucose-6-phosphate isomerase (Fragment) OS=Ophiophagus	OPHHA	17
385	21.23	21.23	41.49	11.26	7.638	tr|V8P091|V8P091_OPHHA	Ubiquitin carboxyl-terminal hydrolase 24 OS=Ophiophagus	OPHHA	9
386	21.18	21.18	39.8	16.71	16.71	tr|V8PH33|V8PH33_OPHHA	Integrin beta (Fragment) OS=Ophiophagus hannah GN=ITGB3	OPHHA	12
387	21.16	21.69	43.07	22.91	17.18	tr|V8NZC8|V8NZC8_OPHHA	Protein transport protein Sec24C (Fragment) OS=Ophiopha	OPHHA	10
388	21.16	21.16	54.86	24.76	24.76	tr|V8P2W6|V8P2W6_OPHHA	Protein LYRIC (Fragment) OS=Ophiophagus hannah GN=MTDH	OPHHA	15
389	21.06	21.06	52.58	21.67	18.83	tr|V8P7C3|V8P7C3_OPHHA	Beta-ureidopropionase (Fragment) OS=Ophiophagus hannah	OPHHA	14
390	21.03	21.03	38.57	28.48	28.48	tr|V8PBJ8|V8PBJ8_OPHHA	EGF-containing fibulin-like extracellular matrix protei	OPHHA	11
391	21	21	60.1	22.54	19.3	tr|V8PC88|V8PC88_OPHHA	Cell division cycle 5-like protein (Fragment) OS=Ophiop	OPHHA	10
392	20.99	20.99	55.46	27.84	26.06	tr|V8PA64|V8PA64_OPHHA	26S proteasome non-ATPase regulatory subunit 6 (Fragmen	OPHHA	12
393	20.94	20.94	27.76	8.1	6.611	tr|V8PAX2|V8PAX2_OPHHA	Fibrillin-1 (Fragment) OS=Ophiophagus hannah GN=FBN1 PE	OPHHA	16
394	20.9	20.9	63.17	26.18	14.46	tr|V8P0H5|V8P0H5_OPHHA	Cullin-3 (Fragment) OS=Ophiophagus hannah GN=CUL3 PE=3	OPHHA	8
395	20.89	20.89	60.33	20.93	19.97	tr|V8PFC9|V8PFC9_OPHHA	Disintegrin and metalloproteinase domain-containing pro	OPHHA	12
396	20.89	20.89	51.5	33.5	32.75	tr|V8NQT0|V8NQT0_OPHHA	PDZ and LIM domain protein 3 OS=Ophiophagus hannah GN=P	OPHHA	15
397	20.84	20.84	54.02	26.32	22.22	tr|V8NN80|V8NN80_OPHHA	Sorting nexin OS=Ophiophagus hannah GN=Snx9 PE=3 SV=1	OPHHA	11
398	20.8	20.8	56.76	28.38	24.87	tr|V8PEA8|V8PEA8_OPHHA	Transforming growth factor-beta-induced protein ig-h3 (	OPHHA	12
399	20.79	20.8	67.33	64.85	64.85	tr|V8P8Q7|V8P8Q7_OPHHA	Ras-related protein R-Ras2 OS=Ophiophagus hannah GN=RRA	OPHHA	12
400	20.77	20.77	63.69	45.85	40.92	tr|V8P7Q0|V8P7Q0_OPHHA	Purine nucleoside phosphorylase (Fragment) OS=Ophiophag	OPHHA	11
401	20.76	20.76	67.41	31.6	27.41	tr|V8PGK3|V8PGK3_OPHHA	Transducin beta-like protein 2 (Fragment) OS=Ophiophagu	OPHHA	11
402	20.71	20.71	67.93	11.44	8.14	tr|V8PA14|V8PA14_OPHHA	TRIO and F-actin-binding protein (Fragment) OS=Ophiopha	OPHHA	10
403	20.65	20.65	50.99	19.34	17.35	tr|V8NX23|V8NX23_OPHHA	Zyxin (Fragment) OS=Ophiophagus hannah GN=ZYX PE=4 SV=1	OPHHA	11
404	20.6	20.6	59.82	32.59	27.9	tr|V8PC74|V8PC74_OPHHA	Septin-10 (Fragment) OS=Ophiophagus hannah GN=SEPT10 PE	OPHHA	10
405	20.54	20.54	51.87	31.17	28.18	tr|V8NN25|V8NN25_OPHHA	AidB (Fragment) OS=Ophiophagus hannah GN=aidB PE=3 SV=1	OPHHA	13
406	20.49	30.88	49.1	14.93	13.25	tr|V8NIG4|V8NIG4_OPHHA	Filamin-C (Fragment) OS=Ophiophagus hannah GN=Flnc PE=4	OPHHA	17
407	20.49	20.49	54.32	16.69	12.37	tr|V8P862|V8P862_OPHHA	Deubiquitinating protein (Fragment) OS=Ophiophagus hann	OPHHA	10
408	20.48	20.48	50.06	18.96	13.53	tr|V8NGK5|V8NGK5_OPHHA	RNA-binding protein 4 (Fragment) OS=Ophiophagus hannah	OPHHA	10
409	20.48	20.48	59.58	31.37	26.95	tr|V8PG00|V8PG00_OPHHA	Succinyl-CoA ligase subunit beta OS=Ophiophagus hannah	OPHHA	10
410	20.47	20.47	63.76	31.65	29.59	tr|V8N5K0|V8N5K0_OPHHA	Uncharacterized protein (Fragment) OS=Ophiophagus hanna	OPHHA	12
411	20.44	20.44	50.63	23.22	23.22	tr|V8NI20|V8NI20_OPHHA	Acetyl-CoA carboxylase 1 (Fragment) OS=Ophiophagus hann	OPHHA	12
412	20.4	20.4	70.75	59.91	56.6	tr|V8P4D1|V8P4D1_OPHHA	Ras-related protein Rab-2A OS=Ophiophagus hannah GN=RAB	OPHHA	13
413	20.39	20.39	76.67	48.79	39.39	tr|V8PG15|V8PG15_OPHHA	Serine/threonine-protein phosphatase OS=Ophiophagus han	OPHHA	10
414	20.36	20.36	86.57	62.19	61.69	tr|V8NDS9|V8NDS9_OPHHA	Ras-related protein Rab-14 OS=Ophiophagus hannah GN=RAB	OPHHA	11
415	20.35	20.35	44.29	15.6	10.58	tr|V8PEN0|V8PEN0_OPHHA	Protein flightless-1-like protein OS=Ophiophagus hannah	OPHHA	10
416	20.32	20.32	45.36	19.1	13.93	tr|V8N949|V8N949_OPHHA	GDH/6PGL endoplasmic bifunctional protein (Fragment) OS	OPHHA	11
417	20.26	20.26	56.7	21.58	15.66	tr|V8PJG1|V8PJG1_OPHHA	Nuclear migration protein nudC (Fragment) OS=Ophiophagu	OPHHA	10
418	20.25	20.25	42.2	14.51	11.93	tr|V8P5K5|V8P5K5_OPHHA	AP-3 complex subunit delta-1 OS=Ophiophagus hannah GN=A	OPHHA	10
419	20.24	20.24	67.93	34.3	31.4	tr|V8P3C8|V8P3C8_OPHHA	Dihydropyrimidine dehydrogenase [NADP+] (Fragment) OS=O	NADP+	11
420	20.2	20.2	62.94	26.03	21.64	tr|V8PC41|V8PC41_OPHHA	LETM1 and EF-hand domain-containing protein 1, mitochon	OPHHA	10
421	20.11	20.11	55.47	25.59	25.59	tr|V8P0Z0|V8P0Z0_OPHHA	Fibrinogen gamma chain (Fragment) OS=Ophiophagus hannah	OPHHA	11
422	20.1	20.1	66.15	32.3	23.21	tr|V8PCE3|V8PCE3_OPHHA	MAGUK p55 subfamily member 6 (Fragment) OS=Ophiophagus	OPHHA	10
423	20.08	20.08	57.28	32.18	27.59	tr|V8PG98|V8PG98_OPHHA	T-complex protein 1 subunit zeta OS=Ophiophagus hannah	OPHHA	10
424	20.07	20.07	50.71	17.17	10.87	tr|V8PIQ3|V8PIQ3_OPHHA	Insulin-degrading enzyme (Fragment) OS=Ophiophagus hann	OPHHA	9
425	20.04	20.18	84.53	55.8	55.8	tr|V8NNE3|V8NNE3_OPHHA	Nucleoside diphosphate kinase (Fragment) OS=Ophiophagus	OPHHA	14
426	20.04	20.04	52.78	44.44	30.56	tr|V8PGJ1|V8PGJ1_OPHHA	Histone H4 (Fragment) OS=Ophiophagus hannah GN=H4-VIII	OPHHA	11
426	0	18.32	58.25	54.37	53.4	tr|V8N8S2|V8N8S2_OPHHA	Histone H4 (Fragment) OS=Ophiophagus hannah GN=L345_010	OPHHA	11
427	19.99	22.04	69.9	41.1	38.51	tr|V8PHF4|V8PHF4_OPHHA	Glycerol-3-phosphate dehydrogenase [NAD(+)] (Fragment)	NAD(+)	19
428	19.96	22.23	80.69	51.74	48.65	tr|V8PHQ7|V8PHQ7_OPHHA	14-3-3 protein zeta/delta (Fragment) OS=Ophiophagus han	OPHHA	14
429	19.92	19.92	85.95	44.12	35.95	tr|V8P625|V8P625_OPHHA	Mitochondrial import receptor subunit TOM34 OS=Ophiopha	OPHHA	9
430	19.91	31.3	57.33	49.33	41.33	tr|V8NQI9|V8NQI9_OPHHA	Uncharacterized protein OS=Ophiophagus hannah GN=L345_1	OPHHA	22
431	19.91	19.91	70.77	49.3	44.01	tr|V8PFV1|V8PFV1_OPHHA	Uncharacterized protein (Fragment) OS=Ophiophagus hanna	OPHHA	10
432	19.85	19.85	37.75	10.58	9.75	tr|V8NQP0|V8NQP0_OPHHA	Tankyrase-1-binding protein (Fragment) OS=Ophiophagus h	OPHHA	10
433	19.85	19.85	37.58	23.23	21.62	tr|V8NNB1|V8NNB1_OPHHA	Peroxisomal acyl-coenzyme A oxidase 2 OS=Ophiophagus ha	OPHHA	11
434	19.8	19.8	75.14	61.88	61.88	tr|V8PEH4|V8PEH4_OPHHA	ADP-ribosylation factor 1 OS=Ophiophagus hannah GN=ARF1	OPHHA	13
434	0	19.77	69.61	61.88	61.88	tr|V8PHQ3|V8PHQ3_OPHHA	ADP-ribosylation factor 3 OS=Ophiophagus hannah GN=ARF3	OPHHA	13
435	19.76	20.01	51.93	20.63	15.47	tr|V8NQA6|V8NQA6_OPHHA	Band 4.1-like protein 1 (Fragment) OS=Ophiophagus hanna	OPHHA	11
436	19.76	19.76	55.8	21.91	16.49	tr|V8NQX6|V8NQX6_OPHHA	Cullin-1 OS=Ophiophagus hannah GN=Cul1 PE=3 SV=1	OPHHA	10
437	19.75	19.75	35.29	8.551	6.812	tr|V8NCC0|V8NCC0_OPHHA	von Willebrand factor (Fragment) OS=Ophiophagus hannah	OPHHA	10
438	19.71	19.71	53.5	27.81	26.54	tr|V8NGN8|V8NGN8_OPHHA	APOBEC1 complementation factor (Fragment) OS=Ophiophagu	OPHHA	10
439	19.69	19.69	61.36	28.21	24.18	tr|V8P7I2|V8P7I2_OPHHA	Methylcrotonoyl-CoA carboxylase beta chain, mitochondri	OPHHA	10
440	19.63	19.63	52.35	36.05	33.83	tr|V8NBS9|V8NBS9_OPHHA	Endoplasmic reticulum resident protein 44 OS=Ophiophagu	OPHHA	12
441	19.6	19.6	51.63	20.55	16.64	tr|V8NR64|V8NR64_OPHHA	E3 UFM1-protein ligase 1 (Fragment) OS=Ophiophagus hann	OPHHA	11
442	19.54	19.54	57.85	25.59	16.77	tr|V8NVI9|V8NVI9_OPHHA	Stress-induced-phosphoprotein 1 (Fragment) OS=Ophiophag	OPHHA	11
443	19.54	19.54	74.48	51.38	44.14	tr|V8NI35|V8NI35_OPHHA	Septin-2 (Fragment) OS=Ophiophagus hannah GN=Sept2 PE=4	OPHHA	9
444	19.53	19.53	51.58	32.11	26.58	tr|V8P9S5|V8P9S5_OPHHA	Aminoacylase-1 OS=Ophiophagus hannah GN=ACY1 PE=4 SV=1	OPHHA	10
445	19.52	19.52	59.77	18.63	15.01	tr|V8NM62|V8NM62_OPHHA	Cytosol aminopeptidase (Fragment) OS=Ophiophagus hannah	OPHHA	9
446	19.5	19.5	50.16	27.06	19.94	tr|V8P5D0|V8P5D0_OPHHA	Sec1 family domain-containing protein 1 OS=Ophiophagus	OPHHA	9
447	19.49	19.49	58.78	25	25	tr|V8PBJ1|V8PBJ1_OPHHA	Myosin light polypeptide 6 (Fragment) OS=Ophiophagus ha	OPHHA	19
448	19.47	19.47	46.31	19.72	16.03	tr|V8P997|V8P997_OPHHA	Glucocorticoid receptor (Fragment) OS=Ophiophagus hanna	OPHHA	9
449	19.47	19.47	42.62	15.04	15.04	tr|V8PFM2|V8PFM2_OPHHA	Delta-1-pyrroline-5-carboxylate synthase (Fragment) OS=	OPHHA	11
450	19.44	19.44	57.01	38.51	38.51	tr|V8P8G5|V8P8G5_OPHHA	Malate dehydrogenase OS=Ophiophagus hannah GN=MDH1 PE=3	OPHHA	17
451	19.43	19.43	64.74	33.61	23.42	tr|V8NAS7|V8NAS7_OPHHA	Sorting nexin-6 (Fragment) OS=Ophiophagus hannah GN=SNX	OPHHA	10
452	19.42	19.42	45.31	16.49	16.49	tr|V8NYL2|V8NYL2_OPHHA	Serine/threonine-protein phosphatase 6 regulatory subun	OPHHA	10
453	19.4	19.4	52.12	24.6	24.45	tr|V8NKS8|V8NKS8_OPHHA	Calpain-2 catalytic subunit (Fragment) OS=Ophiophagus h	OPHHA	12
454	19.38	19.38	48.87	25.5	25.5	tr|V8NYY1|V8NYY1_OPHHA	Gelsolin OS=Ophiophagus hannah GN=GSN PE=4 SV=1	OPHHA	12
455	19.38	19.38	58.66	36.74	23.38	tr|V8PH14|V8PH14_OPHHA	Rho GTPase-activating protein 1 (Fragment) OS=Ophiophag	OPHHA	8
456	19.37	19.38	45.92	22.96	19.79	tr|V8NLC1|V8NLC1_OPHHA	Acyl-coenzyme A oxidase OS=Ophiophagus hannah GN=ACOX1	OPHHA	11
457	19.33	20.66	71.29	28.55	18.18	tr|V8PDF3|V8PDF3_OPHHA	Coiled-coil domain-containing protein 93 (Fragment) OS=	OPHHA	10
458	19.32	19.44	68.19	21.54	17.97	tr|V8P4M9|V8P4M9_OPHHA	RUN and FYVE domain-containing protein 1 OS=Ophiophagus	OPHHA	10
459	19.3	19.3	56.99	26.1	19.21	tr|V8NUX0|V8NUX0_OPHHA	Putative adenosylhomocysteinase 2 (Fragment) OS=Ophioph	OPHHA	8
460	19.3	19.3	65.88	32.94	32.94	tr|V8P9T9|V8P9T9_OPHHA	Citrate lyase subunit beta-like protein, mitochondrial	OPHHA	11
461	19.19	19.19	54.74	16.34	8.357	tr|V8NTV6|V8NTV6_OPHHA	Thrombospondin-1 (Fragment) OS=Ophiophagus hannah GN=TH	OPHHA	8
462	19.18	19.18	51.2	29.19	27.67	tr|V8P8L3|V8P8L3_OPHHA	THO complex subunit 4 OS=Ophiophagus hannah GN=THOC4 PE	OPHHA	12
463	19.15	19.15	47.93	23.65	21.58	tr|V8NTB5|V8NTB5_OPHHA	Protein kinase C and casein kinase substrate in neurons	OPHHA	10
464	19.03	19.08	52.7	22.37	18.02	tr|V8NLV9|V8NLV9_OPHHA	Band 4.1-like protein 2 (Fragment) OS=Ophiophagus hanna	OPHHA	9
465	19.02	21.07	84.44	35.24	35.24	tr|V8PAL4|V8PAL4_OPHHA	Glycerol-3-phosphate dehydrogenase [NAD(+)] (Fragment)	NAD(+)	12
466	18.99	19.07	55.26	19.19	9.942	tr|V8NZ22|V8NZ22_OPHHA	N-alpha-acetyltransferase 15, NatA auxiliary subunit OS	OPHHA	8
467	18.94	18.94	79.94	33.53	31.14	tr|V8NHE8|V8NHE8_OPHHA	Coatomer subunit epsilon (Fragment) OS=Ophiophagus hann	OPHHA	11
468	18.93	18.93	52.82	28.01	22.93	tr|V8P1U0|V8P1U0_OPHHA	Zinc finger protein OS=Ophiophagus hannah GN=Znf326 PE=	OPHHA	9
469	18.9	18.9	51.28	9.946	7.925	tr|V8NQD7|V8NQD7_OPHHA	Acyl-coenzyme A synthetase ACSM4, mitochondrial (Fragme	OPHHA	10
470	18.9	18.9	46.7	26.2	22.99	tr|V8P0U1|V8P0U1_OPHHA	ATP-binding cassette sub-family B member 7, mitochondri	OPHHA	12
471	18.9	18.9	62.07	33.05	30.17	tr|V8PFS2|V8PFS2_OPHHA	Protein phosphatase 1 regulatory subunit 7 OS=Ophiophag	OPHHA	10
472	18.89	18.89	51.42	39.62	39.62	tr|V8PGL2|V8PGL2_OPHHA	ATP synthase subunit O, mitochondrial OS=Ophiophagus ha	OPHHA	15
473	18.86	18.86	46.45	9.747	8.205	tr|V8PF25|V8PF25_OPHHA	Intersectin-1 (Fragment) OS=Ophiophagus hannah GN=ITSN1	OPHHA	10
474	18.86	18.86	48.79	36.39	29.65	tr|V8NS48|V8NS48_OPHHA	Dimethylaniline monooxygenase [N-oxide-forming] 3 (Frag	N-oxide-[illegible]	10
475	18.84	18.84	57.43	25.08	19.2	tr|V8PAI2|V8PAI2_OPHHA	Long-chain fatty acid transport protein 1 OS=Ophiophagu	OPHHA	10

476	18.8	18.8	56.65	29.23	20.16	tr	V8NVN6	V8NVN6_OPHHA	E3 ubiquitin-protein ligase ARIH2 OS=Ophiophagus hannah	OPHHA	8
477	18.76	18.76	49.17	11.93	9.873	tr	V8NYV1	V8NYV1_OPHHA	Transcription elongation regulator 1 (Fragment) OS=Ophi	OPHHA	9
478	18.75	18.75	67.55	30.41	22.24	tr	V8NAJ8	V8NAJ8_OPHHA	Cytoplasmic dynein 1 heavy chain 1 OS=Ophiophagus hanna	OPHHA	8
479	18.61	18.61	77.34	34.64	24.4	tr	V8PDR5	V8PDR5_OPHHA	Tyrosine-protein phosphatase non-receptor type 6 (Fragm	OPHHA	9
480	18.61	18.61	67.53	45.36	34.02	tr	V8PFB9	V8PFB9_OPHHA	40S ribosomal protein S9 OS=Ophiophagus hannah GN=RPS9	OPHHA	11
481	18.6	18.6	57.84	30.35	26.88	tr	V8NLC5	V8NLC5_OPHHA	Methionine aminopeptidase 2 OS=Ophiophagus hannah GN=ME	OPHHA	10
482	18.58	18.58	56.05	21.63	20.23	tr	V8P1F7	V8P1F7_OPHHA	Synapse-associated protein 1 (Fragment) OS=Ophiophagus	OPHHA	10
483	18.56	18.56	77.13	46.12	42.64	tr	V8PFL3	V8PFL3_OPHHA	Carbonic anhydrase 2 OS=Ophiophagus hannah GN=CA2 PE=4	OPHHA	11
484	18.56	18.56	48.16	29.48	29.48	tr	V8P4D7	V8P4D7_OPHHA	Zinc-binding alcohol dehydrogenase domain-containing pr	OPHHA	10
485	18.51	18.51	60	28.53	26.4	tr	V8PGG6	V8PGG6_OPHHA	cAMP-dependent protein kinase type I-alpha regulatory s	OPHHA	9
486	18.47	18.47	48.04	15.12	13.44	tr	V8N9M1	V8N9M1_OPHHA	Disks large-like 1 (Fragment) OS=Ophiophagus hannah GN=	OPHHA	9
487	18.47	18.47	63.03	21.41	21.41	tr	V8NT83	V8NT83_OPHHA	Heterogeneous nuclear ribonucleoprotein D0 OS=Ophiophag	OPHHA	10
488	18.47	18.47	51.23	32.6	30.68	tr	V8NQ34	V8NQ34_OPHHA	Pyruvate dehydrogenase E1 component subunit beta, mitoc	OPHHA	9
489	18.46	18.46	55.41	20.23	12.63	tr	V8NXE2	V8NXE2_OPHHA	Plakophilin-2 OS=Ophiophagus hannah GN=PKP2 PE=4 SV=1	OPHHA	9
490	18.42	18.43	51.19	14.08	11.77	tr	V8PE43	V8PE43_OPHHA	Myosin-VI (Fragment) OS=Ophiophagus hannah GN=MYO6 PE=4	OPHHA	9
491	18.38	18.38	48.52	27.75	25.64	tr	V8PAO8	V8PAO8_OPHHA	Aspartyl aminopeptidase OS=Ophiophagus hannah GN=DNPEP	OPHHA	9
492	18.33	18.33	68.59	26.04	20.48	tr	V8PDY1	V8PDY1_OPHHA	Protein kinase C and casein kinase substrate in neurons	OPHHA	9
493	18.32	18.32	45.17	22.15	15.54	tr	V8NPY3	V8NPY3_OPHHA	Sarcoplasmic/endoplasmic reticulum calcium ATPase 1 (Fr	OPHHA	9
494	18.28	18.28	70.69	32.89	24.61	tr	V8PGL9	V8PGL9_OPHHA	Signal recognition particle 54 kDa protein OS=Ophiophag	OPHHA	10
495	18.25	18.25	76.1	48.9	36.4	tr	V8PIK7	V8PIK7_OPHHA	Dephospho-CoA kinase domain-containing protein (Fragmen	OPHHA	9
496	18.24	18.24	55.66	27.26	20.15	tr	V8P215	V8P215_OPHHA	Double-strand-break repair protein rad21-like protein O	OPHHA	8
497	18.22	18.75	57.08	9.67	7.642	tr	V8NLY0	V8NLY0_OPHHA	Myosin phosphatase Rho-interacting protein (Fragment) O	OPHHA	12
498	18.21	18.21	60.8	34.13	32.27	tr	V8NM87	V8NM87_OPHHA	Uncharacterized protein (Fragment) OS=Ophiophagus hanna	OPHHA	10
499	18.19	18.19	58.41	29.52	27.3	tr	V8P0E5	V8P0E5_OPHHA	Alcohol dehydrogenase [NADP+] (Fragment) OS=Ophiophagus	NADP+	9
500	18.14	18.14	49.26	28.33	26.6	tr	V8NYR3	V8NYR3_OPHHA	N-acylglucosamine 2-epimerase OS=Ophiophagus hannah GN=	OPHHA	11
501	18.07	18.07	36.12	16.72	9.179	tr	V8P3H3	V8P3H3_OPHHA	Nuclear pore complex protein OS=Ophiophagus hannah GN=N	OPHHA	9
502	18.05	18.05	57.84	19.78	15.92	tr	V8P0Y3	V8P0Y3_OPHHA	Cold shock domain-containing protein E1 (Fragment) OS=O	OPHHA	10
503	17.98	17.98	60.5	27.9	25.71	tr	V8NVB9	V8NVB9_OPHHA	Glyceraldehyde-3-phosphate dehydrogenase OS=Ophiophagus	OPHHA	14
504	17.95	17.95	33.52	12.71	7.555	tr	V8P6B4	V8P6B4_OPHHA	Stabilin-2 OS=Ophiophagus hannah GN=Stab2 PE=4 SV=1	OPHHA	10
505	17.85	17.85	54.58	15.44	10.76	tr	V8PIB5	V8PIB5_OPHHA	Rab GTPase-activating protein 1 (Fragment) OS=Ophiophag	OPHHA	8
506	17.83	17.83	52.75	28.64	22.49	tr	V8P866	V8P866_OPHHA	1,4-alpha-glucan-branching enzyme (Fragment) OS=Ophioph	OPHHA	9
507	17.83	17.83	70.49	39.93	34.72	tr	V8N9K6	V8N9K6_OPHHA	EH domain-containing protein 1 (Fragment) OS=Ophiophagu	OPHHA	10
508	17.81	17.81	42.5	10.75	7.906	tr	V8P7L1	V8P7L1_OPHHA	Putative E3 ubiquitin-protein ligase TRIP12 OS=Ophiopha	OPHHA	9
509	17.81	17.81	65.43	51.44	47.74	tr	V8P9M3	V8P9M3_OPHHA	N-alpha-acetyltransferase 10 OS=Ophiophagus hannah GN=N	OPHHA	9
510	17.79	17.92	32.67	19.57	13.56	tr	V8PBX9	V8PBX9_OPHHA	Clathrin coat assembly protein OS=Ophiophagus hannah GN	OPHHA	11
511	17.76	17.76	73.76	59.28	58.82	tr	V8PB57	V8PB57_OPHHA	Inorganic pyrophosphatase 2, mitochondrial (Fragment) O	OPHHA	10
512	17.71	17.71	56.19	44.76	44.76	tr	V8P9V2	V8P9V2_OPHHA	Abhydrolase domain-containing protein 14B OS=Ophiophagu	OPHHA	9
513	17.69	17.69	45.43	16.52	11.89	tr	V8P2K1	V8P2K1_OPHHA	Endoplasmic reticulum aminopeptidase 1 (Fragment) OS=Op	OPHHA	8
514	17.65	17.65	45.57	16.05	13.74	tr	V8P9C4	V8P9C4_OPHHA	ATP-binding cassette sub-family F member 1 OS=Ophiophag	OPHHA	10
515	17.65	17.65	45.29	30.53	27.99	tr	V8NC48	V8NC48_OPHHA	Nucleosome assembly protein 1-like 1 OS=Ophiophagus han	OPHHA	10
516	17.63	17.63	50.44	33.72	31.09	tr	V8PC58	V8PC58_OPHHA	Serine/threonine-protein kinase MST4 (Fragment) OS=Ophi	OPHHA	9
517	17.63	17.63	60.33	34.1	32.79	tr	V8PEF3	V8PEF3_OPHHA	Argininosuccinate lyase (Fragment) OS=Ophiophagus hanna	OPHHA	11
518	17.58	17.58	52.61	31.7	31.7	tr	V8PGZ7	V8PGZ7_OPHHA	60S ribosomal protein L5 (Fragment) OS=Ophiophagus hann	OPHHA	16
519	17.56	17.56	35.99	10.47	7.9	tr	V8P4N3	V8P4N3_OPHHA	TBC1 domain family member 9B (Fragment) OS=Ophiophagus	OPHHA	8
520	17.56	17.56	67.37	33.95	29.44	tr	V8NVF6	V8NVF6_OPHHA	Diphosphoinositol polyphosphate phosphohydrolase 1 OS=O	OPHHA	10
521	17.53	17.66	62.83	31.09	27.61	tr	V8NNU0	V8NNU0_OPHHA	Eukaryotic translation initiation factor 2 subunit 3 OS	OPHHA	10
522	17.53	17.53	59.9	10.54	6.299	tr	V8NRH5	V8NRH5_OPHHA	Pinin OS=Ophiophagus hannah GN=PNN PE=4 SV=1	OPHHA	10
523	17.51	17.84	44.84	14.17	10.69	tr	V8PBI7	V8PBI7_OPHHA	AP-3 complex subunit beta-1 (Fragment) OS=Ophiophagus h	OPHHA	8
524	17.5	17.53	51.04	28.13	28.13	tr	V8N986	V8N986_OPHHA	Chloride intracellular channel protein 1 OS=Ophiophagus	OPHHA	11
525	17.48	17.48	40.4	20.03	18.53	tr	V8NNI6	V8NNI6_OPHHA	Very long-chain acyl-CoA synthetase (Fragment) OS=Ophio	OPHHA	11
526	17.45	17.45	50.09	29.23	17.78	tr	V8NKF4	V8NKF4_OPHHA	Eukaryotic translation initiation factor 2A OS=Ophiopha	OPHHA	8
527	17.42	17.42	62.09	36.9	28.24	tr	V8NVR5	V8NVR5_OPHHA	Eukaryotic translation initiation factor 3 subunit E OS	OPHHA	9
528	17.4	17.4	54.86	31.42	31.42	tr	V8NKC0	V8NKC0_OPHHA	Serine-threonine kinase receptor-associated protein (Fr	OPHHA	9
529	17.38	17.38	35.44	8.053	6.598	tr	V8NWU3	V8NWU3_OPHHA	Zinc finger ZZ-type and EF-hand domain-containing prote	OPHHA	14
530	17.24	17.24	42.83	6.921	3.938	tr	V8PEE2	V8PEE2_OPHHA	Microtubule-actin cross-linking factor 1, isoforms 1/2/	OPHHA	12
531	17.23	17.23	51	11.67	10.91	tr	V8NXV0	V8NXV0_OPHHA	WASH complex subunit strumpellin OS=Ophiophagus hannah	OPHHA	9
532	17.23	17.23	48.67	20.25	17.27	tr	V8P1P7	V8P1P7_OPHHA	CD2-associated protein OS=Ophiophagus hannah GN=CD2AP P	OPHHA	8
533	17.22	17.22	58.99	32.46	30.26	tr	V8P2Y9	V8P2Y9_OPHHA	DNA helicase (Fragment) OS=Ophiophagus hannah GN=RUVBL1	OPHHA	9
534	17.2	18.37	61.81	20.69	11.63	tr	V8NTV0	V8NTV0_OPHHA	Protein 4.1 (Fragment) OS=Ophiophagus hannah GN=EPB41 P	OPHHA	7
535	17.18	36.65	61.32	27.7	21.96	tr	V8PI56	V8PI56_OPHHA	Radixin (Fragment) OS=Ophiophagus hannah GN=RDX PE=4 SV	OPHHA	17
536	17.14	17.14	73.77	15.63	14.3	tr	V8NDX8	V8NDX8_OPHHA	ELKS/Rab6-interacting/CAST family member 1 (Fragment) O	OPHHA	10
537	17.12	17.12	57.46	16.67	11.44	tr	V8P8A3	V8P8A3_OPHHA	FACT complex subunit SPT16 (Fragment) OS=Ophiophagus ha	OPHHA	8
538	17.12	17.12	87.63	36.56	36.56	tr	V8PBW2	V8PBW2_OPHHA	Tumor protein D52 OS=Ophiophagus hannah GN=TPD52 PE=4 S	OPHHA	10
539	17.11	17.11	33.56	7.458	6.621	tr	V8PCM0	V8PCM0_OPHHA	N-alpha-acetyltransferase 25, NatB auxiliary subunit (F	OPHHA	12
540	17.1	17.1	59.33	13.43	11.57	tr	V8P9X4	V8P9X4_OPHHA	Exocyst complex component 2 (Fragment) OS=Ophiophagus h	OPHHA	8
541	17.09	17.1	52.6	16.31	14.89	tr	V8N6I2	V8N6I2_OPHHA	Myosin-Ie (Fragment) OS=Ophiophagus hannah GN=Myole PE=	OPHHA	9
542	17.06	17.06	75.29	38.4	34.98	tr	V8P2P8	V8P2P8_OPHHA	Serine/arginine-rich splicing factor 5 OS=Ophiophagus h	OPHHA	11
543	17.03	17.03	65.93	33.19	33.19	tr	V8NQN0	V8NQN0_OPHHA	Superoxide dismutase (Fragment) OS=Ophiophagus hannah G	OPHHA	13
544	17.01	17.01	100	53.33	48.48	tr	V8PBL8	V8PBL8_OPHHA	Peptidyl-prolyl cis-trans isomerase A OS=Ophiophagus ha	OPHHA	17
545	16.98	17.77	41.32	23.74	13.01	tr	V8N8G4	V8N8G4_OPHHA	Cytochrome protein (Fragment) OS=Ophiophagus hannah GN=	OPHHA	8
546	16.98	16.98	45.65	14.35	14.24	tr	V8P6N9	V8P6N9_OPHHA	cGMP-specific 3',5'-cyclic phosphodiesterase (Fragment)	OPHHA	9
547	16.97	16.97	62.31	16.88	14.33	tr	V8NMR0	V8NMR0_OPHHA	Eukaryotic translation initiation factor 4 gamma 2 (Fra	OPHHA	8
548	16.95	16.95	57.87	37.07	22.93	tr	V8P1T5	V8P1T5_OPHHA	Eukaryotic translation initiation factor 3 subunit M OS	OPHHA	8
549	16.85	16.85	42.51	17.68	12.3	tr	V8NWD0	V8NWD0_OPHHA	Valyl-tRNA synthetase OS=Ophiophagus hannah GN=Vars PE=	OPHHA	7
550	16.85	16.85	32.44	27.06	21.69	tr	V8PFW4	V8PFW4_OPHHA	Importin subunit alpha OS=Ophiophagus hannah GN=KPNA4 P	OPHHA	9
551	16.82	16.85	57.42	5.488	4.25	tr	V8P6K4	V8P6K4_OPHHA	Dystonin (Fragment) OS=Ophiophagus hannah GN=DST PE=4 S	OPHHA	11
552	16.82	16.82	66.48	13.91	11.28	tr	V8P909	V8P909_OPHHA	Tyrosine-protein kinase OS=Ophiophagus hannah GN=JAK1 P	OPHHA	9
553	16.82	16.82	40	23.8	19.71	tr	V8NAV0	V8NAV0_OPHHA	Heterogeneous nuclear ribonucleoprotein L (Fragment) OS	OPHHA	9
554	16.82	16.82	59.08	32.62	32.62	tr	V8NF69	V8NF69_OPHHA	Enoyl-CoA hydratase, mitochondrial (Fragment) OS=Ophiop	OPHHA	11
555	16.82	16.82	50.12	27.74	21.68	tr	V8NAW1	V8NAW1_OPHHA	T-complex protein 1 subunit eta (Fragment) OS=Ophiophag	OPHHA	9
556	16.79	16.79	50.71	27.4	17.95	tr	V8P244	V8P244_OPHHA	Bifunctional UDP-N-acetylglucosamine 2-epimerase/N-acet	OPHHA	9
557	16.78	16.78	49.01	41.06	41.06	tr	V8PBD2	V8PBD2_OPHHA	40S ribosomal protein S14 OS=Ophiophagus hannah GN=RPS1	OPHHA	11
558	16.76	16.86	47.7	19.37	15.38	tr	V8NCB6	V8NCB6_OPHHA	GTPase-activating protein and VPS9 domain-containing pr	OPHHA	9
559	16.75	18.34	50.62	6.234	4.541	tr	V8P8L1	V8P8L1_OPHHA	Keratin, type I cytoskeletal 19 (Fragment) OS=Ophiophag	OPHHA	13
560	16.75	16.75	66.36	29.44	26.17	tr	V8P0B9	V8P0B9_OPHHA	Uncharacterized protein (Fragment) OS=Ophiophagus hanna	OPHHA	8
561	16.73	16.73	56.85	46.89	43.57	tr	V8NWH3	V8NWH3_OPHHA	GTP-binding protein SAR1a (Fragment) OS=Ophiophagus han	OPHHA	9
562	16.72	16.78	47.79	12	12	tr	V8P3B6	V8P3B6_OPHHA	Vacuolar protein sorting-associated protein 18-like pro	OPHHA	9
563	16.7	16.7	56.82	28.41	21.73	tr	V8NEW4	V8NEW4_OPHHA	Gamma-soluble NSF attachment protein (Fragment) OS=Ophi	OPHHA	9
564	16.69	16.69	61.64	14.47	12.26	tr	V8NM38	V8NM38_OPHHA	La-related protein 1 (Fragment) OS=Ophiophagus hannah G	OPHHA	9
565	16.65	16.65	50.96	27.6	23.99	tr	V8P1Q2	V8P1Q2_OPHHA	Coiled-coil domain-containing protein 6 (Fragment) OS=O	OPHHA	10
566	16.62	16.73	76.03	19.28	14.88	tr	V8NZ46	V8NZ46_OPHHA	Golgin subfamily A member 5 (Fragment) OS=Ophiophagus h	OPHHA	8
567	16.54	16.54	57.14	6.785	4.712	tr	V8PIJ1	V8PIJ1_OPHHA	Dystonin (Fragment) OS=Ophiophagus hannah GN=DST PE=4 S	OPHHA	10
568	16.53	16.53	28.8	11.7	10.67	tr	V8N855	V8N855_OPHHA	Cytochrome protein OS=Ophiophagus hannah GN=CYP3A29 PE=	OPHHA	12
569	16.52	16.52	42.78	19.51	14.82	tr	V8PCA2	V8PCA2_OPHHA	Coronin (Fragment) OS=Ophiophagus hannah GN=CORO1C PE=3	OPHHA	9
570	16.51	16.51	60.06	37.42	24.84	tr	V8NLS7	V8NLS7_OPHHA	Aminoacyl tRNA synthase complex-interacting multifuncti	OPHHA	11
571	16.49	16.49	69.17	45	40.83	tr	V8NUD5	V8NUD5_OPHHA	Catechol O-methyltransferase OS=Ophiophagus hannah GN=C	OPHHA	10

572	16.47	16.47	37.98	17.08	15.42	tr|V8NP25|V8NP25_OPHHA Protein numb-like protein OS=Ophiophagus hannah GN=NUMB	OPHHA	9
573	16.42	16.51	43.74	14.29	13.29	tr|V8PIK1|V8PIK1_OPHHA Cytosolic non-specific dipeptidase (Fragment) OS=Ophiop	OPHHA	8
574	16.39	16.39	46.35	16.73	11.21	tr|V8NC92|V8NC92_OPHHA IQ motif and SEC7 domain-containing protein 1 (Fragment	OPHHA	9
575	16.37	16.37	74.95	23.75	22.44	tr|V8NRG3|V8NRG3_OPHHA Protein POF1B (Fragment) OS=Ophiophagus hannah GN=Pof1b	OPHHA	8
576	16.28	16.28	50.38	12.06	10.55	tr|V8P1R5|V8P1R5_OPHHA Multimerin-1 (Fragment) OS=Ophiophagus hannah GN=MMRN1	OPHHA	9
577	16.23	16.55	56.49	12.08	7.361	tr|V8NZF4|V8NZF4_OPHHA Tight junction protein ZO-2 (Fragment) OS=Ophiophagus h	OPHHA	8
578	16.23	16.23	69.57	27.11	21.99	tr|V8NP16|V8NP16_OPHHA Sorting nexin-7 OS=Ophiophagus hannah GN=SNX7 PE=4 SV=1	OPHHA	8
579	16.21	18.22	58.6	34.71	32.48	tr|V8N7V7|V8N7V7_OPHHA 4-trimethylaminobutyraldehyde dehydrogenase (Fragment)	OPHHA	15
580	16.18	16.18	51.42	10.08	6.616	tr|V8PAH4|V8PAH4_OPHHA TATA element modulatory factor (Fragment) OS=Ophiophagu	OPHHA	8
581	16.18	16.18	73.6	41.2	32	tr|V8NV58|V8NV58_OPHHA Carbonyl reductase [NADPH] 1 (Fragment) OS=Ophiophagus	NADPH	8
582	16.17	16.17	33.08	22.82	20.26	tr|V8NAR9|V8NAR9_OPHHA Interleukin enhancer-binding factor 2 OS=Ophiophagus ha	OPHHA	8
583	16.13	16.13	65.82	33.76	33.76	tr|V8PF77|V8PF77_OPHHA Methylglutaconyl-CoA hydratase, mitochondrial (Fragment	OPHHA	9
584	16.12	16.12	36.11	10.97	7.89	tr|V8NNV7|V8NNV7_OPHHA Inositol 1,4,5-trisphosphate receptor type 1 (Fragment)	OPHHA	10
585	16.12	16.12	62.94	27.27	22.38	tr|V8NZV7|V8NZV7_OPHHA Actin-related protein 2/3 complex subunit 2 (Fragment)	OPHHA	9
586	16.11	16.23	38.7	19.48	8.312	tr|V8P1U6|V8P1U6_OPHHA AP-1 complex subunit gamma-1 OS=Ophiophagus hannah GN=A	OPHHA	6
587	16.11	16.11	73.64	34.96	28.08	tr|V8NLY8|V8NLY8_OPHHA Phosphotriesterase-related protein OS=Ophiophagus hanna	OPHHA	9
588	16.1	16.11	40.25	23.54	23.54	tr|V8NA47|V8NA47_OPHHA Cytochrome b-c1 complex subunit 2, mitochondrial (Fragm	OPHHA	10
589	16.08	16.08	40.9	12.7	11.72	tr|V8NU38|V8NU38_OPHHA Protein FAM91A1 (Fragment) OS=Ophiophagus hannah GN=FAM	OPHHA	9
590	16.08	16.08	37.75	14.48	10.55	tr|V8NKX1|V8NKX1_OPHHA Cleavage and polyadenylation specificity factor subunit	OPHHA	9
591	16.08	16.08	77.07	29.6	29.6	tr|V8NDE2|V8NDE2_OPHHA L-asparaginase (Fragment) OS=Ophiophagus hannah GN=asrg	OPHHA	9
592	16.04	16.08	39.73	7.797	6.773	tr|V8PFW1|V8PFW1_OPHHA Laminin subunit alpha-4 (Fragment) OS=Ophiophagus hanna	OPHHA	9
593	16.04	16.04	59.57	51.49	51.06	tr|V8PFX9|V8PFX9_OPHHA Protein phosphatase 1 regulatory subunit 1B OS=Ophiopha	OPHHA	9
594	16.01	17.89	64.26	17.95	13.59	tr|V8NJD4|V8NJD4_OPHHA Dynamin-1 (Fragment) OS=Ophiophagus hannah GN=Dnm1 PE=4	OPHHA	9
595	16	16.01	43.68	22.2	22.2	tr|V8NZM8|V8NZM8_OPHHA Protein disulfide-isomerase A6 (Fragment) OS=Ophiophagu	OPHHA	10
596	15.97	15.97	41.06	18.25	16.61	tr|V8NGC4|V8NGC4_OPHHA Poly(U)-binding-splicing factor PUF60 (Fragment) OS=Oph	OPHHA	8
597	15.93	15.93	67.41	37.7	31.31	tr|V8NVA0|V8NVA0_OPHHA Glutaminyl-tRNA synthetase (Fragment) OS=Ophiophagus ha	OPHHA	8
598	15.92	15.92	52.31	16.37	16.19	tr|V8P2S3|V8P2S3_OPHHA Apoptosis inhibitor 5 (Fragment) OS=Ophiophagus hannah	OPHHA	10
599	15.92	15.92	72.85	20.81	20.81	tr|V8PI87|V8PI87_OPHHA Glutathione S-transferase 2 OS=Ophiophagus hannah GN=GS	OPHHA	8
600	15.9	15.9	55.42	25.78	23.61	tr|V8P4S2|V8P4S2_OPHHA TAR DNA-binding protein 43 OS=Ophiophagus hannah GN=TAR	OPHHA	8
601	15.86	15.86	51.87	22.86	19.12	tr|V8PHM3|V8PHM3_OPHHA Ribonuclease inhibitor OS=Ophiophagus hannah GN=Rnh1 PE	OPHHA	9
602	15.84	15.84	58.3	29.79	20.21	tr|V8P4B2|V8P4B2_OPHHA ERO1-like protein beta OS=Ophiophagus hannah GN=ERO1LB	OPHHA	7
603	15.83	15.83	55.5	29.36	27.98	tr|V8P678|V8P678_OPHHA Heterogeneous nuclear ribonucleoprotein H OS=Ophiophagu	OPHHA	11
604	15.82	15.82	69.64	51.79	47.77	tr|V8NQR3|V8NQR3_OPHHA Elongation factor 1-beta OS=Ophiophagus hannah GN=EEF1B	OPHHA	9
605	15.79	15.83	66.35	9.563	8.737	tr|V8PGH1|V8PGH1_OPHHA SAFB-like transcription modulator OS=Ophiophagus hannah	OPHHA	8
606	15.78	15.78	50.1	22.74	21.13	tr|V8PBN0|V8PBN0_OPHHA Carbohydrate kinase domain-containing protein (Fragment	OPHHA	8
607	15.73	15.73	74.93	33.9	29.06	tr|V8P425|V8P425_OPHHA LIM and cysteine-rich domains protein 1 (Fragment) OS=O	OPHHA	8
608	15.72	15.72	83.61	44.26	40.98	tr|V8NJ95|V8NJ95_OPHHA Glutathione S-transferase Mu 7 (Fragment) OS=Ophiophagu	OPHHA	12
609	15.69	15.69	41.52	10.68	6.59	tr|V8NQD2|V8NQD2_OPHHA Laminin subunit beta-1 (Fragment) OS=Ophiophagus hannah	OPHHA	7
610	15.68	15.68	54.23	23.91	23.91	tr|V8NXC3|V8NXC3_OPHHA DnaJ-like subfamily B member 11 OS=Ophiophagus hannah G	OPHHA	8
611	15.66	15.66	43.58	15.14	10.78	tr|V8NTU0|V8NTU0_OPHHA Transportin-1 (Fragment) OS=Ophiophagus hannah GN=TNPO1	OPHHA	8
612	15.64	15.64	40.55	19.53	16.56	tr|V8NJI3|V8NJI3_OPHHA Protein transport protein Sec31A (Fragment) OS=Ophiopha	OPHHA	10
613	15.59	15.59	77.81	28.94	21.22	tr|V8PAB0|V8PAB0_OPHHA LIM and SH3 domain protein 1 (Fragment) OS=Ophiophagus	OPHHA	12
614	15.59	15.59	68.86	33.33	33.33	tr|V8NRV2|V8NRV2_OPHHA Acetyl-CoA carboxylase (Fragment) OS=Ophiophagus hannah	OPHHA	9
615	15.56	15.56	35.27	11.37	9.984	tr|V8PHH1|V8PHH1_OPHHA Acylamino-acid-releasing enzyme (Fragment) OS=Ophiophag	OPHHA	8
616	15.55	15.55	54.99	25.06	19.95	tr|V8NGU6|V8NGU6_OPHHA Signal recognition particle receptor subunit beta OS=Op	OPHHA	8
617	15.55	15.55	62.75	40.39	32.94	tr|V8NIG9|V8NIG9_OPHHA NADH-cytochrome b5 reductase 3 (Fragment) OS=Ophiophagu	OPHHA	8
618	15.53	15.53	54.21	28.22	28.22	tr|V8NJU8|V8NJU8_OPHHA Alpha-aminoadipic semialdehyde dehydrogenase OS=Ophioph	OPHHA	10
619	15.53	15.53	70.83	40.83	37.08	tr|V8P224|V8P224_OPHHA Prohibitin OS=Ophiophagus hannah GN=PHB PE=4 SV=1	OPHHA	8
620	15.5	15.72	68.82	41.76	38.24	tr|V8P2E0|V8P2E0_OPHHA Guanine nucleotide-binding protein subunit beta-4 OS=Op	OPHHA	9
621	15.48	15.48	51.19	42.46	41.67	tr|V8PAU7|V8PAU7_OPHHA 6-phosphogluconolactonase (Fragment) OS=Ophiophagus han	OPHHA	9
622	15.45	15.45	61.64	18.51	13.43	tr|V8NKM7|V8NKM7_OPHHA Cullin-4B (Fragment) OS=Ophiophagus hannah GN=CUL4B PE=	OPHHA	8
623	15.44	15.44	64.33	17.68	13.87	tr|V8N8X8|V8N8X8_OPHHA Nucleolin (Fragment) OS=Ophiophagus hannah GN=NCL PE=4	OPHHA	9
624	15.42	15.42	50.84	15.23	9.979	tr|V8PHR6|V8PHR6_OPHHA Ras-interacting protein 1 (Fragment) OS=Ophiophagus han	OPHHA	7
625	15.4	15.4	61.54	47.01	43.59	tr|V8NJ09|V8NJ09_OPHHA Cysteine desulfurase, mitochondrial OS=Ophiophagus hann	OPHHA	8
626	15.39	15.39	62.3	25.2	25.2	tr|V8NDX6|V8NDX6_OPHHA Translocation protein SEC63-like protein (Fragment) OS=	OPHHA	9
627	15.38	15.38	42.64	6.498	5.322	tr|V8NYN0|V8NYN0_OPHHA Branched-chain-amino-acid aminotransferase, mitochondri	OPHHA	11
628	15.35	15.35	67.92	21.97	21.97	tr|V8NP09|V8NP09_OPHHA ATP synthase mitochondrial F1 complex assembly factor 1	OPHHA	9
629	15.35	15.35	67.78	56.67	38.89	tr|V8P961|V8P961_OPHHA Calmodulin (Fragment) OS=Ophiophagus hannah GN=CALM PE=	OPHHA	10
629	0	15.14	70	48.89	38.89	tr|V8NW64|V8NW64_OPHHA Calmodulin (Fragment) OS=Ophiophagus hannah GN=CALM PE=	OPHHA	10
630	15.32	15.32	55.43	18.99	18.99	tr|V8NC60|V8NC60_OPHHA Glutathione S-transferase (Fragment) OS=Ophiophagus han	OPHHA	8
631	15.3	15.3	65.47	33.98	24.31	tr|V8NP28|V8NP28_OPHHA Serine/threonine-protein kinase OSR1 (Fragment) OS=Ophi	OPHHA	8
632	15.26	43.79	60.73	56.5	56.5	tr|V8NKE3|V8NKE3_OPHHA Actin, aortic smooth muscle OS=Ophiophagus hannah GN=AC	OPHHA	35
632	0	43.79	65.52	57.47	57.47	tr|V8NYT7|V8NYT7_OPHHA Uncharacterized protein OS=Ophiophagus hannah GN=L345_0	OPHHA	36
633	15.23	15.23	47.08	7.985	6.871	tr|V8P6V6|V8P6V6_OPHHA Aldehyde dehydrogenase family 16 member A1 (Fragment) O	OPHHA	8
634	15.21	18.12	59.6	26.27	20.29	tr|V8PEM1|V8PEM1_OPHHA Acetyl-coenzyme A synthetase 2-like, mitochondrial (Fra	OPHHA	8
635	15.17	15.17	50.87	16.76	15.46	tr|V8NBV6|V8NBV6_OPHHA Exocyst complex component 8 (Fragment) OS=Ophiophagus h	OPHHA	8
636	15.17	15.17	73.49	62.65	57.83	tr|V8NZX4|V8NZX4_OPHHA Succinyl-CoA ligase GDP-forming subunit beta (Fragment)	OPHHA	9
637	15.16	15.16	54.06	13.14	11.89	tr|V8PBI1|V8PBI1_OPHHA Ubiquitin-protein ligase E3A OS=Ophiophagus hannah GN=U	OPHHA	7
638	15.16	15.16	44.96	30.58	26.98	tr|V8NEC7|V8NEC7_OPHHA Uncharacterized protein (Fragment) OS=Ophiophagus hanna	OPHHA	7
639	15.15	15.15	60.79	25.75	21.81	tr|V8PAV2|V8PAV2_OPHHA Hydroxysteroid dehydrogenase-like protein 2 OS=Ophiopha	OPHHA	9
640	15.13	15.13	39.14	6.549	5.592	tr|V8NTJ9|V8NTJ9_OPHHA 5'-3' exoribonuclease 1 (Fragment) OS=Ophiophagus hanna	OPHHA	10
641	15.13	15.13	31.24	24.72	24.72	tr|V8NPZ8|V8NPZ8_OPHHA Ubiquinone biosynthesis monooxygenase COQ6 (Fragment) O	OPHHA	9
642	15.11	15.11	60.17	42.62	28.41	tr|V8PDG5|V8PDG5_OPHHA Guanine deaminase (Fragment) OS=Ophiophagus hannah GN=G	OPHHA	8
643	15.06	15.06	72.57	23	17.3	tr|V8PB76|V8PB76_OPHHA Glutathione synthetase OS=Ophiophagus hannah GN=GSS PE=	OPHHA	7
644	15.06	15.06	47.88	20.44	17.66	tr|V8NNX0|V8NNX0_OPHHA Glycyl-tRNA synthetase (Fragment) OS=Ophiophagus hannah	OPHHA	12
645	15.06	15.06	72.86	28.02	23.3	tr|V8P1E1|V8P1E1_OPHHA Hsp90 co-chaperone Cdc37 OS=Ophiophagus hannah GN=CDC37	OPHHA	7
646	15.03	19.05	47.6	6.286	5.831	tr|V8PBC0|V8PBC0_OPHHA Acetyl-CoA carboxylase 2 (Fragment) OS=Ophiophagus hann	OPHHA	12
647	14.99	14.99	67.92	23.08	20.08	tr|V8NVJ4|V8NVJ4_OPHHA Nucleolar protein 58 (Fragment) OS=Ophiophagus hannah G	OPHHA	8
648	14.98	14.98	67.3	18.56	12.81	tr|V8PA01|V8PA01_OPHHA Polyribonucleotide nucleotidyltransferase 1, mitochondr	OPHHA	7
649	14.97	14.97	45.44	16.49	14.74	tr|V8NYP3|V8NYP3_OPHHA Complement component C8 beta chain (Fragment) OS=Ophiop	OPHHA	8
650	14.95	14.95	27.27	13.7	11.46	tr|V8NRX3|V8NRX3_OPHHA Anion exchange protein (Fragment) OS=Ophiophagus hannah	OPHHA	8
651	14.95	14.95	68	31.56	20.89	tr|V8NB73|V8NB73_OPHHA Putative signal peptidase complex subunit 2 (Fragment)	OPHHA	8
652	14.94	14.94	77.36	36.49	32.43	tr|V8NMA9|V8NMA9_OPHHA Sulfotransferase (Fragment) OS=Ophiophagus hannah GN=SU	OPHHA	11
653	14.91	23.38	42.17	17.22	14.96	tr|V8NT90|V8NT90_OPHHA Cytoplasmic FMR1-interacting protein 2 (Fragment) OS=Op	OPHHA	13
654	14.91	14.91	32.96	7.431	6.22	tr|V8NKA6|V8NKA6_OPHHA Angiotensin-converting enzyme OS=Ophiophagus hannah GN=	OPHHA	7
655	14.9	14.91	63.45	16.24	16.24	tr|V8NP00|V8NP00_OPHHA Thyroid hormone receptor-associated protein 3 OS=Ophiop	OPHHA	8
656	14.89	14.89	63.81	33.98	22.65	tr|V8PH70|V8PH70_OPHHA V-type proton ATPase subunit C 1 OS=Ophiophagus hannah	OPHHA	7
657	14.82	14.82	48.36	12.72	9.351	tr|V8PF53|V8PF53_OPHHA Partitioning defective 3-like protein (Fragment) OS=Oph	OPHHA	7
658	14.82	14.82	62.16	53.28	49.81	tr|V8NEA4|V8NEA4_OPHHA WD repeat-containing protein 1 OS=Ophiophagus hannah GN	OPHHA	8
659	14.8	14.8	82.53	30.14	21.92	tr|V8NN02|V8NN02_OPHHA Pyruvate dehydrogenase E1 component subunit alpha, soma	OPHHA	7
660	14.79	15.68	40.06	11.62	11.09	tr|V8PFQ6|V8PFQ6_OPHHA Nuclear pore complex protein (Fragment) OS=Ophiophagus	OPHHA	8
661	14.79	14.79	73.98	36.73	32.14	tr|V8P6H0|V8P6H0_OPHHA V-type proton ATPase subunit E 1 OS=Ophiophagus hannah	OPHHA	8
662	14.78	14.79	53.61	21.29	17.87	tr|V8P710|V8P710_OPHHA COP9 signalosome complex subunit 1 (Fragment) OS=Ophiop	OPHHA	7
663	14.75	14.77	41.41	5.482	3.346	tr|V8PET1|V8PET1_OPHHA Vacuolar protein sorting-associated protein 13C (Fragme	OPHHA	8
664	14.73	14.73	56.67	18.74	16.16	tr|V8NTJ2|V8NTJ2_OPHHA Isovaleryl-CoA dehydrogenase, mitochondrial OS=Ophiopha	OPHHA	10
665	14.69	14.72	73.71	36.31	21.68	tr|V8PBF5|V8PBF5_OPHHA Obg-like ATPase 1 (Fragment) OS=Ophiophagus hannah GN=O	OPHHA	7

666	14.68	14.68	61.3	38.31	34.87	tr|V8NYI2|V8NYI2_OPHHA Proteasome subunit alpha type (Fragment) OS=Ophiophagus	OPHHA	9
667	14.67	23.11	39.14	23.03	21.54	tr|V8N9E0|V8N9E0_OPHHA AP-1 complex subunit beta-1 OS=Ophiophagus hannah GN=AP	OPHHA	16
668	14.67	14.67	65.28	25.23	19.44	tr|V8NVT0|V8NVT0_OPHHA Putative imidazolonepropionase (Fragment) OS=Ophiophagu	OPHHA	8
669	14.63	14.63	45.27	19.67	12.86	tr|V8NCH8|V8NCH8_OPHHA E3 ubiquitin-protein ligase UBR4 (Fragment) OS=Ophiopha	OPHHA	7
670	14.63	14.63	52.3	25.38	16.52	tr|V8NHH5|V8NHH5_OPHHA Xaa-Pro aminopeptidase 1 OS=Ophiophagus hannah GN=XPNPE	OPHHA	7
671	14.62	14.62	55.89	21.37	21.37	tr|V8NZ64|V8NZ64_OPHHA Serpin H1 (Fragment) OS=Ophiophagus hannah GN=SERPINH1	OPHHA	8
672	14.61	14.61	81.93	52.1	52.1	tr|V8P3M5|V8P3M5_OPHHA Brain acid soluble protein 1-like protein (Fragment) OS	OPHHA	11
673	14.59	14.59	43.66	30.28	21.48	tr|V8NVV2|V8NVV2_OPHHA Polypyrimidine tract-binding protein 2 (Fragment) OS=Op	OPHHA	9
674	14.57	14.58	66.45	43.87	34.19	tr|V8N6R3|V8N6R3_OPHHA Uncharacterized protein OS=Ophiophagus hannah GN=L345_1	OPHHA	8
675	14.56	14.57	35.86	11.81	8.793	tr|V8NFU9|V8NFU9_OPHHA Sortilin-related receptor (Fragment) OS=Ophiophagus han	OPHHA	7
676	14.54	14.54	58.02	50.62	40.74	tr|V8NP41|V8NP41_OPHHA Ras suppressor protein 1 (Fragment) OS=Ophiophagus hann	OPHHA	7
677	14.53	14.53	69.96	42.15	31.39	tr|V8NHZ3|V8NHZ3_OPHHA GrpE protein homolog OS=Ophiophagus hannah GN=GRPEL1 PE	OPHHA	7
678	14.52	16.74	44.13	23.32	16.61	tr|V8NPT8|V8NPT8_OPHHA Ankyrin-1 (Fragment) OS=Ophiophagus hannah GN=Ank1 PE=4	OPHHA	8
679	14.51	29.57	61.05	20.27	17.53	tr|V8P5W8|V8P5W8_OPHHA Alpha-actinin-2 OS=Ophiophagus hannah GN=ACTN2 PE=4 SV=	OPHHA	14
680	14.48	14.48	41.4	28.34	22.29	tr|V8P3J3|V8P3J3_OPHHA Autophagy-related protein 3 OS=Ophiophagus hannah GN=AT	OPHHA	7
681	14.47	14.47	42.86	6.707	5.834	tr|V8NZ98|V8NZ98_OPHHA DNA-directed RNA polymerase (Fragment) OS=Ophiophagus h	OPHHA	7
682	14.45	14.45	56.18	49.44	49.44	tr|V8NSY1|V8NSY1_OPHHA Uncharacterized protein (Fragment) OS=Ophiophagus hanna	OPHHA	12
683	14.44	15.33	57.1	31.88	27.54	tr|V8NRH4|V8NRH4_OPHHA Beta-1-syntrophin OS=Ophiophagus hannah GN=SNTB1 PE=4 S	OPHHA	8
684	14.4	14.42	37.5	4.923	3	tr|V8P932|V8P932_OPHHA Kalirin (Fragment) OS=Ophiophagus hannah GN=Kalrn PE=4	OPHHA	8
685	14.4	14.4	57.34	20.94	14.68	tr|V8PEP3|V8PEP3_OPHHA PDZ and LIM domain protein 7 (Fragment) OS=Ophiophagus	OPHHA	7
686	14.37	14.37	37.86	13.69	6.667	tr|V8PCV3|V8PCV3_OPHHA PDZ and LIM domain protein 5 (Fragment) OS=Ophiophagus	OPHHA	6
687	14.34	14.34	70.9	26.72	22.75	tr|V8NQW5|V8NQW5_OPHHA Heterogeneous nuclear ribonucleoprotein G OS=Ophiophagu	OPHHA	8
688	14.31	14.38	67.26	25	22.92	tr|V8P4Y1|V8P4Y1_OPHHA Annexin OS=Ophiophagus hannah GN=ANXA1 PE=3 SV=1	OPHHA	9
689	14.3	14.4	66.79	32.86	27.14	tr|V8N7X5|V8N7X5_OPHHA Uncharacterized protein (Fragment) OS=Ophiophagus hanna	OPHHA	8
690	14.27	14.27	51.94	42.23	38.35	tr|V8P4Z8|V8P4Z8_OPHHA Membrane-associated progesterone receptor component 2 O	OPHHA	8
691	14.27	14.27	45.99	26.64	26.64	tr|V8PID6|V8PID6_OPHHA NADH dehydrogenase [ubiquinone] iron-sulfur protein 3,	OPHHA	8
692	14.24	18.64	71.63	35.46	29.55	tr|V8PHS9|V8PHS9_OPHHA Protein phosphatase 1A (Fragment) OS=Ophiophagus hannah	OPHHA	11
693	14.24	14.24	81.37	28.6	27.27	tr|V8PGX0|V8PGX0_OPHHA Glutaryl-CoA dehydrogenase, mitochondrial (Fragment) OS	OPHHA	9
694	14.23	14.23	51.79	44.62	41.04	tr|V8NLH8|V8NLH8_OPHHA Calponin OS=Ophiophagus hannah GN=CNN3 PE=3 SV=1	OPHHA	11
695	14.22	14.22	60.95	12.88	8.194	tr|V8P1T7|V8P1T7_OPHHA Liprin-alpha-1 (Fragment) OS=Ophiophagus hannah GN=PPFI	OPHHA	7
696	14.22	14.22	43.3	15.82	12.73	tr|V8PAJ0|V8PAJ0_OPHHA Conserved oligomeric Golgi complex subunit 3 (Fragment)	OPHHA	7
697	14.21	14.21	49.11	18.83	18.83	tr|V8P2F3|V8P2F3_OPHHA Coagulation factor XIII A chain OS=Ophiophagus hannah G	OPHHA	9
698	14.2	14.2	64.24	10.66	8.197	tr|V8PJ09|V8PJ09_OPHHA ATP-dependent RNA helicase DDX42 (Fragment) OS=Ophiopha	OPHHA	7
699	14.2	14.2	54.55	32.21	27.01	tr|V8NAG5|V8NAG5_OPHHA Dihydrolipoyl dehydrogenase, mitochondrial (Fragment) O	OPHHA	8
700	14.17	14.76	49.84	16.25	12.66	tr|V8NDP3|V8NDP3_OPHHA C-factor (Fragment) OS=Ophiophagus hannah GN=csgA PE=4	OPHHA	9
701	14.17	14.17	76.21	24.48	20.34	tr|V8NZU9|V8NZU9_OPHHA Eukaryotic translation initiation factor 4B (Fragment)	OPHHA	7
702	14.17	14.17	38.41	24.68	22.75	tr|V8PC91|V8PC91_OPHHA Malic enzyme OS=Ophiophagus hannah GN=ME1 PE=3 SV=1	OPHHA	7
703	14.15	14.15	71.38	23.1	23.1	tr|V8NSZ3|V8NSZ3_OPHHA Proteasome subunit beta type (Fragment) OS=Ophiophagus	OPHHA	7
704	14.13	14.13	64.78	20.53	18.83	tr|V8NGY3|V8NGY3_OPHHA UDP-N-acetylhexosamine pyrophosphorylase (Fragment) OS=	OPHHA	8
705	14.13	14.13	49.51	20.91	17.16	tr|V8NWA8|V8NWA8_OPHHA NEDD8-activating enzyme E1 regulatory subunit (Fragment	OPHHA	7
706	14.13	14.13	53.15	21.4	21.4	tr|V8NXE6|V8NXE6_OPHHA Hydroxyacylglutathione hydrolase, mitochondrial (Fragme	OPHHA	8
707	14.13	14.13	59.51	38.65	38.65	tr|V8PF33|V8PF33_OPHHA Uncharacterized protein (Fragment) OS=Ophiophagus hanna	OPHHA	12
708	14.08	14.14	57.89	33.87	27.69	tr|V8P9H4|V8P9H4_OPHHA FAS-associated factor 2 OS=Ophiophagus hannah GN=FAF2 P	OPHHA	8
709	14.04	14.04	47.28	18.56	18.56	tr|V8NN61|V8NN61_OPHHA Lamina-associated polypeptide 2, isoforms alpha/zeta (F	OPHHA	9
710	14.03	14.03	56.54	34.28	34.28	tr|V8NXE1|V8NXE1_OPHHA Pyridoxal kinase (Fragment) OS=Ophiophagus hannah GN=Pd	OPHHA	11
711	14.03	14.03	48.77	43.83	43.83	tr|V8N5V3|V8N5V3_OPHHA Peptidyl-prolyl cis-trans isomerase OS=Ophiophagus hann	OPHHA	9
712	14	14	63.21	39.38	35.23	tr|V8P9H5|V8P9H5_OPHHA Rho-related GTP-binding protein RhoC OS=Ophiophagus han	OPHHA	10
713	13.97	13.97	41.67	10.82	6.817	tr|V8NXS9|V8NXS9_OPHHA Myoferlin (Fragment) OS=Ophiophagus hannah GN=Myof PE=4	OPHHA	7
714	13.96	13.96	43.5	7.357	5.709	tr|V8NBL5|V8NBL5_OPHHA Collagen alpha-4(VI) chain (Fragment) OS=Ophiophagus ha	OPHHA	7
715	13.95	14.18	58.09	9.243	6.135	tr|V8NLN1|V8NLN1_OPHHA C-Jun-amino-terminal kinase-interacting protein 4 (Frag	OPHHA	6
716	13.94	13.94	46.75	18.04	12.84	tr|V8P1G3|V8P1G3_OPHHA FACT complex subunit SSRP1 (Fragment) OS=Ophiophagus ha	OPHHA	7
717	13.91	13.91	69.37	10.62	6.288	tr|V8NPY9|V8NPY9_OPHHA Thyroid receptor-interacting protein 11 (Fragment) OS=O	OPHHA	6
718	13.89	13.89	50.49	28.68	24.75	tr|V8P7L6|V8P7L6_OPHHA Serine/threonine-protein kinase 4 (Fragment) OS=Ophioph	OPHHA	7
719	13.89	13.89	92.06	60.32	54.76	tr|V8P013|V8P013_OPHHA Histidine triad nucleotide-binding protein 1 OS=Ophioph	OPHHA	7
720	13.88	13.88	35.42	14.58	12.88	tr|V8NDA3|V8NDA3_OPHHA Eukaryotic peptide chain release factor GTP-binding sub	OPHHA	7
721	13.88	13.88	44.02	16.79	16.79	tr|V8P9Y0|V8P9Y0_OPHHA Heterogeneous nuclear ribonucleoproteins C1/C2 (Fragmen	OPHHA	8
722	13.86	17.1	39.01	15.1	13	tr|V8N6B9|V8N6B9_OPHHA Cytochrome protein (Fragment) OS=Ophiophagus hannah GN=	OPHHA	10
723	13.86	13.86	58.91	31.72	31.72	tr|V8PI51|V8PI51_OPHHA Twinfilin-2 (Fragment) OS=Ophiophagus hannah GN=TWF2 PE	OPHHA	8
724	13.86	13.86	55.14	29.91	23.05	tr|V8NTD6|V8NTD6_OPHHA Epidermal retinol dehydrogenase 2 OS=Ophiophagus hannah	OPHHA	6
725	13.85	13.88	64.34	39.34	35.25	tr|V8P9B2|V8P9B2_OPHHA Thioredoxin-dependent peroxide reductase, mitochondrial	OPHHA	10
726	13.84	13.84	54.49	25.75	22.16	tr|V8PEA3|V8PEA3_OPHHA Histone H2A (Fragment) OS=Ophiophagus hannah GN=H2AFY P	OPHHA	8
727	13.83	13.83	55.26	42.11	39.47	tr|V8P8N1|V8P8N1_OPHHA Actin-related protein 2/3 complex subunit 5 (Fragment)	OPHHA	8
728	13.82	13.82	73.29	55.48	43.15	tr|V8NVJ6|V8NVJ6_OPHHA 40S ribosomal protein S16 OS=Ophiophagus hannah GN=RPS1	OPHHA	8
729	13.82	13.82	43.77	35.46	29.07	tr|V8PDC3|V8PDC3_OPHHA 5'-AMP-activated protein kinase subunit gamma-1 OS=Ophi	OPHHA	8
730	13.8	13.8	60.37	33.64	27.65	tr|V8NMV9|V8NMV9_OPHHA Ribosomal protein OS=Ophiophagus hannah GN=RPL10A PE=3	OPHHA	7
731	13.79	13.79	49.36	37.77	31.76	tr|V8NS71|V8NS71_OPHHA Alpha-soluble NSF attachment protein (Fragment) OS=Ophi	OPHHA	8
732	13.78	13.78	39.51	14.81	14.81	tr|V8NR60|V8NR60_OPHHA Clathrin interactor 1 (Fragment) OS=Ophiophagus hannah	OPHHA	7
733	13.76	16.01	57.53	29.04	24.66	tr|V8P6I7|V8P6I7_OPHHA Eukaryotic initiation factor 4A-III OS=Ophiophagus hann	OPHHA	9
734	13.76	13.76	45.66	18.28	9.091	tr|V8NII9|V8NII9_OPHHA Trafficking protein particle complex subunit 10 (Fragme	OPHHA	6
735	13.7	13.7	48.94	30.85	20	tr|V8PFK4|V8PFK4_OPHHA Ubiquitin-like modifier-activating enzyme ATG7 (Fragmen	OPHHA	7
736	13.66	13.66	46.28	28.67	28.67	tr|V8P934|V8P934_OPHHA Hydroxyacid-oxoacid transhydrogenase, mitochondrial OS=	OPHHA	9
737	13.63	13.63	52.67	18.85	10.29	tr|V8PCG2|V8PCG2_OPHHA Cullin-5 (Fragment) OS=Ophiophagus hannah GN=CUL5 PE=3	OPHHA	7
738	13.62	13.62	63.36	23.14	15.7	tr|V8P8Y4|V8P8Y4_OPHHA Ethanolamine-phosphate cytidylyltransferase OS=Ophiopha	OPHHA	6
739	13.59	13.59	49.14	25	25	tr|V8N4Z5|V8N4Z5_OPHHA Short-chain dehydrogenase/reductase family 9C member 7	OPHHA	9
740	13.56	13.56	57.54	39.11	35.2	tr|V8P180|V8P180_OPHHA Sorcin OS=Ophiophagus hannah GN=SRI PE=4 SV=1	OPHHA	10
741	13.55	13.55	44.75	13.78	11.55	tr|V8PG97|V8PG97_OPHHA AMP deaminase 2 (Fragment) OS=Ophiophagus hannah GN=AMP	OPHHA	7
742	13.53	13.55	23.32	15.55	15.55	tr|V8P3I9|V8P3I9_OPHHA Protein transport protein Sec61 subunit alpha isoform 1	OPHHA	7
743	13.53	13.53	43.79	16.5	12.91	tr|V8NHH4|V8NHH4_OPHHA Peroxisomal carnitine O-octanoyltransferase OS=Ophiopha	OPHHA	7
744	13.53	13.53	62.26	36.78	29.09	tr|V8NBL1|V8NBL1_OPHHA Delta-1-pyrroline-5-carboxylate dehydrogenase, mitochon	OPHHA	10
745	13.53	13.53	66.8	34.43	34.43	tr|V8NWH1|V8NWH1_OPHHA Vesicle-associated membrane protein-associated protein	OPHHA	8
746	13.52	13.55	59.47	36.32	26.58	tr|V8PGY1|V8PGY1_OPHHA Sorting and assembly machinery component 50-like protei	OPHHA	6
747	13.52	13.52	41.76	20.59	20.59	tr|V8NXN6|V8NXN6_OPHHA Fumarylacetoacetase (Fragment) OS=Ophiophagus hannah GN	OPHHA	10
748	13.46	13.46	52.73	19.21	14.31	tr|V8PHP9|V8PHP9_OPHHA Bridging integrator 2 (Fragment) OS=Ophiophagus hannah	OPHHA	7
749	13.42	13.42	83.27	39.78	36.06	tr|V8NKP2|V8NKP2_OPHHA COP9 signalosome complex subunit 4 (Fragment) OS=Ophiop	OPHHA	7
750	13.4	13.55	41.49	7.635	7.23	tr|V8NZ18|V8NZ18_OPHHA Canalicular multispecific organic anion transporter 1 (	OPHHA	7
751	13.38	13.43	50.9	21.1	16.37	tr|V8NYY8|V8NYY8_OPHHA Dystrobrevin alpha (Fragment) OS=Ophiophagus hannah GN=	OPHHA	7
752	13.37	13.37	64.02	19	15.31	tr|V8NKS7|V8NKS7_OPHHA Prolyl 4-hydroxylase subunit alpha-1 (Fragment) OS=Ophi	OPHHA	7
753	13.36	13.36	40.22	12.62	12.62	tr|V8NRG5|V8NRG5_OPHHA von Willebrand factor A domain-containing protein 5A (F	OPHHA	8
754	13.35	13.35	35.03	9.189	5.599	tr|V8P808|V8P808_OPHHA Telomerase protein component 1 (Fragment) OS=Ophiophagu	OPHHA	6
755	13.35	13.35	89.84	50	50	tr|V8NR18|V8NR18_OPHHA Uncharacterized protein (Fragment) OS=Ophiophagus hanna	OPHHA	8
756	13.34	13.34	87.58	46.58	46.58	tr|V8NCG9|V8NCG9_OPHHA Serine/arginine-rich splicing factor 9 (Fragment) OS=Op	OPHHA	9
757	13.33	13.33	46.6	11.22	8.927	tr|V8NLW9|V8NLW9_OPHHA Rho GTPase-activating protein 35 (Fragment) OS=Ophiopha	OPHHA	8
758	13.33	13.33	50.66	18.09	18.09	tr|V8NKY2|V8NKY2_OPHHA Serine/threonine-protein phosphatase (Fragment) OS=Ophi	OPHHA	7
759	13.3	13.3	57.85	14.19	10.74	tr|V8P629|V8P629_OPHHA Beta-adrenergic receptor kinase 1 (Fragment) OS=Ophioph	OPHHA	7
760	13.28	13.28	74.39	21.68	17.57	tr|V8P229|V8P229_OPHHA Src substrate cortactin (Fragment) OS=Ophiophagus hanna	OPHHA	7
761	13.28	13.28	47.74	30.97	28.71	tr|V8PB68|V8PB68_OPHHA 26S proteasome non-ATPase regulatory subunit 14 OS=Ophi	OPHHA	7

762	13.25	18.43	42.72	34.95	29.77	tr	V8NIF8	V8NIF8_OPHHA	L-lactate dehydrogenase OS=Ophiophagus hannah GN=LDHB P	OPHHA	13
763	13.23	13.23	54.71	46.47	46.47	tr	V8P3X6	V8P3X6_OPHHA	N-alpha-acetyltransferase 50 OS=Ophiophagus hannah GN=N	OPHHA	7
764	13.21	13.21	39.93	21.32	16.07	tr	V8NVS4	V8NVS4_OPHHA	CTP synthase OS=Ophiophagus hannah GN=CTPS PE=3 SV=1	OPHHA	7
765	13.2	13.2	53.16	32.28	29.11	tr	V8P7L4	V8P7L4_OPHHA	Pyrroline-5-carboxylate reductase OS=Ophiophagus hannah	OPHHA	8
766	13.19	13.39	40.44	9.737	7.178	tr	V8P8T3	V8P8T3_OPHHA	Protein VPRBP OS=Ophiophagus hannah GN=VPRBP PE=4 SV=1	OPHHA	6
767	13.18	13.18	43.12	14.34	7.415	tr	V8N9N8	V8N9N8_OPHHA	Small nuclear ribonucleoprotein helicase (Fragment) OS=	OPHHA	6
768	13.17	13.17	52.62	9.927	5.165	tr	V8NZ25	V8NZ25_OPHHA	Nuclear mitotic apparatus protein 1 (Fragment) OS=Ophio	OPHHA	6
769	13.16	13.16	57.05	6.686	4.759	tr	V8P082	V8P082_OPHHA	Melanoma inhibitory activity protein 3 OS=Ophiophagus h	OPHHA	8
770	13.16	13.16	76.28	44.65	32.56	tr	V8NTQ2	V8NTQ2_OPHHA	Vesicle-trafficking protein SEC22b OS=Ophiophagus hanna	OPHHA	9
771	13.15	13.15	42.18	10.14	9.467	tr	V8NML1	V8NML1_OPHHA	Phosphorylase b kinase regulatory subunit alpha, liver	OPHHA	7
772	13.11	13.11	49.82	18.79	14.54	tr	V8PCX9	V8PCX9_OPHHA	Drebrin-like protein (Fragment) OS=Ophiophagus hannah G	OPHHA	9
773	13.05	13.05	41.88	11.37	6.961	tr	V8NTS6	V8NTS6_OPHHA	Glutamyl aminopeptidase (Fragment) OS=Ophiophagus hanna	OPHHA	6
774	13.04	25.61	52.85	15.72	12.52	tr	V8NFJ6	V8NFJ6_OPHHA	Brefeldin A-inhibited guanine nucleotide-exchange prote	OPHHA	12
775	13.03	13.03	74.05	25.14	24.86	tr	V8NND4	V8NND4_OPHHA	Serine/threonine-protein phosphatase 2A 55 kDa regulato	OPHHA	7
776	13.01	13.01	55.01	15.99	13.86	tr	V8PH40	V8PH40_OPHHA	Paraspeckle component 1 OS=Ophiophagus hannah GN=PSPC1	OPHHA	6
777	13	13	24.62	7.859	6.09	tr	V8NAV5	V8NAV5_OPHHA	ATP-binding cassette sub-family A member 8-A (Fragment)	OPHHA	7
778	12.99	12.99	62.56	38.86	38.86	tr	V8P6Y7	V8P6Y7_OPHHA	Uncharacterized protein OS=Ophiophagus hannah GN=L345_0	OPHHA	8
779	12.97	12.97	40.38	7.258	6.203	tr	V8P819	V8P819_OPHHA	N-acetyltransferase 10 (Fragment) OS=Ophiophagus hannah	OPHHA	7
780	12.97	12.97	34.49	11.07	8.794	tr	V8NFG3	V8NFG3_OPHHA	Agrin (Fragment) OS=Ophiophagus hannah GN=AGRN PE=4 SV=	OPHHA	8
781	12.96	13.39	67.63	13.89	8.855	tr	V8NDC6	V8NDC6_OPHHA	N-alpha-acetyltransferase 16, NatA auxiliary subunit (F	OPHHA	6
782	12.96	12.96	57.05	19.96	14.97	tr	V8P3S0	V8P3S0_OPHHA	Protein kinase C alpha type (Fragment) OS=Ophiophagus h	OPHHA	6
783	12.95	12.95	25.94	14.09	12.29	tr	V8PEG3	V8PEG3_OPHHA	Dolichyl-diphosphooligosaccharide--protein glycosyltran	OPHHA	7
784	12.88	12.88	41.89	22.76	22.52	tr	V8PDK4	V8PDK4_OPHHA	Mannose-1-phosphate guanyltransferase alpha-A OS=Ophiop	OPHHA	7
785	12.87	12.87	51.04	19.73	13.66	tr	V8P0K3	V8P0K3_OPHHA	Asparagine synthetase [glutamine-hydrolyzing] OS=Ophiop	OPHHA	6
786	12.83	12.83	24.65	7.567	5.94	tr	V8NM36	V8NM36_OPHHA	Protein MON2-like protein OS=Ophiophagus hannah GN=mon2	OPHHA	6
787	12.82	12.82	31.66	12.57	10.01	tr	V8NK75	V8NK75_OPHHA	Nuclear pore complex protein OS=Ophiophagus hannah GN=N	OPHHA	7
788	12.8	12.8	50.51	34.81	22.53	tr	V8P8Z3	V8P8Z3_OPHHA	Mitochondrial dicarboxylate carrier (Fragment) OS=Ophio	OPHHA	7
789	12.78	12.78	37.56	19.38	17.46	tr	V8NL87	V8NL87_OPHHA	Nucleosome assembly protein 1-like 4 (Fragment) OS=Ophi	OPHHA	7
790	12.74	12.74	62.23	18.25	15.33	tr	V8NAE4	V8NAE4_OPHHA	Sarcolemmal membrane-associated protein OS=Ophiophagus	OPHHA	6
791	12.7	12.7	53.17	27.07	25.37	tr	V8NVT6	V8NVT6_OPHHA	Biglycan (Fragment) OS=Ophiophagus hannah GN=BGN PE=4 S	OPHHA	6
792	12.68	12.68	59.45	37.4	32.28	tr	V8NLD4	V8NLD4_OPHHA	NADH dehydrogenase [ubiquinone] iron-sulfur protein 2,	OPHHA	8
793	12.67	12.67	79.26	24.07	24.07	tr	V8N9W8	V8N9W8_OPHHA	Alpha-2-macroglobulin receptor-associated protein (Frag	OPHHA	8
794	12.66	12.66	66.46	16.67	16.67	tr	V8NQ28	V8NQ28_OPHHA	6-phosphofructo-2-kinase/fructose-2, 6-biphosphatase 4	OPHHA	8
795	12.65	12.65	52.27	11.25	9.619	tr	V8NBW6	V8NBW6_OPHHA	Glutamate--cysteine ligase catalytic subunit OS=Ophioph	OPHHA	6
796	12.63	12.63	69.97	34.98	26.07	tr	V8NZA4	V8NZA4_OPHHA	LIM and senescent cell antigen-like-containing domain p	OPHHA	9
797	12.6	12.6	58.69	44.13	40.38	tr	V8P9G7	V8P9G7_OPHHA	Dolichol-phosphate mannosyltransferase (Fragment) OS=Op	OPHHA	8
798	12.59	12.59	51.8	8.546	6.522	tr	V8P5I0	V8P5I0_OPHHA	Poly [ADP-ribose] polymerase OS=Ophiophagus hannah GN=P	ADP-ribo	6
799	12.59	12.59	35.7	9.974	8.924	tr	V8P7C1	V8P7C1_OPHHA	Nuclear pore complex protein OS=Ophiophagus hannah GN=N	OPHHA	7
800	12.52	12.52	62.27	37.27	34.55	tr	V8P4E3	V8P4E3_OPHHA	Maleylacetoacetate isomerase OS=Ophiophagus hannah GN=G	OPHHA	11
801	12.52	12.52	78.26	56.52	51.3	tr	V8P297	V8P297_OPHHA	60S ribosomal protein L30 OS=Ophiophagus hannah GN=RPL3	OPHHA	6
802	12.5	12.5	36.27	19.57	14.18	tr	V8NAG9	V8NAG9_OPHHA	Bifunctional coenzyme A synthase OS=Ophiophagus hannah	OPHHA	6
803	12.49	12.49	54.78	40.44	23.9	tr	V8PC26	V8PC26_OPHHA	Synaptic vesicle membrane protein VAT-1-like protein (F	OPHHA	7
804	12.45	12.45	55.52	16.93	11.76	tr	V8PCK1	V8PCK1_OPHHA	Protein OS-9 (Fragment) OS=Ophiophagus hannah GN=OS9 PE	OPHHA	6
805	12.44	12.44	41.35	7.275	6.509	tr	V8NYR5	V8NYR5_OPHHA	alpha-1,2-Mannosidase OS=Ophiophagus hannah GN=EDEM3 PE	OPHHA	7
806	12.44	12.44	45.93	12.22	10.41	tr	V8PBD9	V8PBD9_OPHHA	A-kinase anchor protein 2 (Fragment) OS=Ophiophagus han	OPHHA	7
807	12.42	12.42	32.53	5.459	3.817	tr	V8NUB4	V8NUB4_OPHHA	Protein dopey-2 (Fragment) OS=Ophiophagus hannah GN=DOP	OPHHA	6
808	12.41	12.41	53.59	13.11	6.557	tr	V8P312	V8P312_OPHHA	Putative ATP-dependent RNA helicase DDX46 (Fragment) OS	OPHHA	5
809	12.41	12.41	50.34	40.69	36.55	tr	V8N7G7	V8N7G7_OPHHA	Cytochrome c oxidase subunit 5A, mitochondrial (Fragmen	OPHHA	8
810	12.4	12.4	45.04	15.63	15.63	tr	V8PBM4	V8PBM4_OPHHA	WD repeat-containing protein 44 (Fragment) OS=Ophiophag	OPHHA	9
811	12.4	12.4	42.59	17.64	17.07	tr	V8P2R3	V8P2R3_OPHHA	Dynactin subunit 4 (Fragment) OS=Ophiophagus hannah GN=	OPHHA	7
812	12.35	14.51	76.42	34.93	25.97	tr	V8NRN4	V8NRN4_OPHHA	Septin-9 OS=Ophiophagus hannah GN=SEPT9 PE=3 SV=1	OPHHA	9
813	12.35	12.35	48.24	10.47	8.588	tr	V8P4D8	V8P4D8_OPHHA	Glucosamine--fructose-6-phosphate aminotransferase [iso	OPHHA	7
814	12.34	12.34	79.17	41.67	41.67	tr	V8P478	V8P478_OPHHA	14 kDa phosphohistidine phosphatase (Fragment) OS=Ophio	OPHHA	6
815	12.33	12.33	46.85	9.321	6.623	tr	V8PC22	V8PC22_OPHHA	SWI/SNF complex subunit SMARCC2 (Fragment) OS=Ophiophag	OPHHA	6
816	12.32	12.32	67.78	27.22	27.22	tr	V8NU77	V8NU77_OPHHA	[Pyruvate dehydrogenase [lipoamide]] kinase isozyme 1,	Pyruvate	6
817	12.32	12.32	32.34	28.19	24.04	tr	V8P2Q0	V8P2Q0_OPHHA	Glycogenin-1 OS=Ophiophagus hannah GN=Gyg1 PE=4 SV=1	OPHHA	6
818	12.3	12.3	31.32	13.01	9.228	tr	V8P1N3	V8P1N3_OPHHA	Plasminogen (Fragment) OS=Ophiophagus hannah GN=PLG PE=	OPHHA	6
819	12.29	12.29	38.56	9.804	8.235	tr	V8P8L8	V8P8L8_OPHHA	Hepatocyte growth factor-regulated tyrosine kinase subs	OPHHA	6
820	12.28	12.28	47.53	9.801	8.326	tr	V8PIC9	V8PIC9_OPHHA	Diacylglycerol kinase (Fragment) OS=Ophiophagus hannah	OPHHA	6
821	12.26	12.33	51.41	12.1	7.56	tr	V8NW59	V8NW59_OPHHA	PERQ amino acid-rich with GYF domain-containing protein	OPHHA	6
822	12.26	12.26	65	25.25	22	tr	V8NZ75	V8NZ75_OPHHA	Putative RNA-binding protein Luc7-like 2 OS=Ophiophagus	OPHHA	6
823	12.23	12.23	36.92	34.05	31.18	tr	V8NAI3	V8NAI3_OPHHA	N(G),N(G)-dimethylarginine dimethylaminohydrolase 1 OS=	OPHHA	7
824	12.21	12.21	82.3	29.19	29.19	tr	V8P4E1	V8P4E1_OPHHA	Ferrochelatase, mitochondrial (Fragment) OS=Ophiophagus	OPHHA	7
825	12.19	12.46	61.81	19.23	14.84	tr	V8PFN4	V8PFN4_OPHHA	HCLS1-binding protein 3 (Fragment) OS=Ophiophagus hanna	OPHHA	6
826	12.19	12.19	38.3	5.133	4.393	tr	V8PIE8	V8PIE8_OPHHA	M-phase phosphoprotein 8 (Fragment) OS=Ophiophagus hann	OPHHA	9
827	12.18	12.18	72.44	48.03	40.94	tr	V8NNR3	V8NNR3_OPHHA	Serine/arginine-rich splicing factor 3 OS=Ophiophagus h	OPHHA	8
828	12.16	12.16	42.57	9.979	8.599	tr	V8NTO0	V8NTO0_OPHHA	Uncharacterized protein (Fragment) OS=Ophiophagus hanna	OPHHA	8
829	12.14	12.14	93.07	66.34	59.41	tr	V8P1H5	V8P1H5_OPHHA	Pterin-4-alpha-carbinolamine dehydratase 2 (Fragment) O	OPHHA	9
830	12.12	12.12	70.45	39.2	39.2	tr	V8N8Y4	V8N8Y4_OPHHA	Proteasome subunit alpha type-2 (Fragment) OS=Ophiophag	OPHHA	8
831	12.11	12.11	47.39	9.13	8.261	tr	V8NGB9	V8NGB9_OPHHA	Uncharacterized protein (Fragment) OS=Ophiophagus hanna	OPHHA	8
832	12.09	13.66	48.93	10.81	6.518	tr	V8P5N6	V8P5N6_OPHHA	Clustered mitochondria protein homolog OS=Ophiophagus h	OPHHA	6
833	12.09	12.09	58.22	25.91	25.91	tr	V8P110	V8P110_OPHHA	Sialic acid synthase OS=Ophiophagus hannah GN=NANS PE=4	OPHHA	8
834	12.08	12.08	42.86	20.48	17.86	tr	V8NZM4	V8NZM4_OPHHA	Angio-associated migratory cell protein OS=Ophiophagus	OPHHA	6
835	12.08	12.08	53.99	29.58	25.35	tr	V8PIE5	V8PIE5_OPHHA	40S ribosomal protein S7 OS=Ophiophagus hannah GN=rps7	OPHHA	7
836	12.06	12.06	43.27	19.93	18.85	tr	V8N4T2	V8N4T2_OPHHA	Inter-alpha-trypsin inhibitor heavy chain H3 (Fragment)	OPHHA	7
837	12.06	12.06	82	70	70	tr	V8NQ30	V8NQ30_OPHHA	Uncharacterized protein (Fragment) OS=Ophiophagus hanna	OPHHA	9
838	12.05	12.05	52.15	11.37	9.349	tr	V8NNT9	V8NNT9_OPHHA	Alpha-mannosidase OS=Ophiophagus hannah GN=MAN2A1 PE=3	OPHHA	7
839	12.05	12.05	40	16.31	12.87	tr	V8PBL4	V8PBL4_OPHHA	Protein sel-1-like 1 (Fragment) OS=Ophiophagus hannah G	OPHHA	6
840	12.05	12.05	53.43	28.36	22.69	tr	V8NV38	V8NV38_OPHHA	DnaJ-like subfamily B member 1 OS=Ophiophagus hannah GN	OPHHA	7
841	12.05	12.05	51.85	22.9	20.88	tr	V8N9J7	V8N9J7_OPHHA	60S acidic ribosomal protein P0 OS=Ophiophagus hannah G	OPHHA	6
842	12.03	12.03	64.18	25.75	25.75	tr	V8NGI7	V8NGI7_OPHHA	Putative methyltransferase (Fragment) OS=Ophiophagus ha	OPHHA	6
843	12.02	14.34	52.24	22.08	22.08	tr	V8P534	V8P534_OPHHA	Polypyrimidine tract-binding protein 1 OS=Ophiophagus h	OPHHA	10
844	12.02	14.15	62.96	29.01	24.38	tr	V8NSI2	V8NSI2_OPHHA	Uncharacterized protein (Fragment) OS=Ophiophagus hanna	OPHHA	7
845	12.02	12.02	58.31	25.76	23.39	tr	V8NIU2	V8NIU2_OPHHA	Flotillin-1 (Fragment) OS=Ophiophagus hannah GN=flot1 P	OPHHA	7
846	12.01	12.24	51.79	15.54	9.96	tr	V8NPI0	V8NPI0_OPHHA	Monofunctional C1-tetrahydrofolate synthase, mitochondr	OPHHA	6
847	12.01	12.01	52.04	23.43	20.16	tr	V8PAE4	V8PAE4_OPHHA	Mannose-1-phosphate guanyltransferase beta-A (Fragment)	OPHHA	7
848	12	12	48.9	15.76	13.11	tr	V8P6C7	V8P6C7_OPHHA	Trafficking protein particle complex subunit 9 (Fragmen	OPHHA	7
849	12	12	68.52	33.8	33.8	tr	V8NTB0	V8NTB0_OPHHA	Ras-related protein Rab-5A OS=Ophiophagus hannah GN=RAB	OPHHA	6
850	11.99	11.99	55.39	16.82	14.56	tr	V8N7X3	V8N7X3_OPHHA	Mitofusin-2 (Fragment) OS=Ophiophagus hannah GN=Mfn2 PE	OPHHA	6
851	11.98	11.98	66.23	53.25	28.57	tr	V8NY31	V8NY31_OPHHA	Protein-L-isoaspartate O-methyltransferase OS=Ophiophag	OPHHA	7
852	11.96	11.96	52.93	13.41	6.039	tr	V8PAM2	V8PAM2_OPHHA	Acyl-CoA dehydrogenase family member 11 OS=Ophiophagus	OPHHA	6
853	11.96	11.96	75.4	38.1	38.1	tr	V8N341	V8N341_OPHHA	Histone H2B (Fragment) OS=Ophiophagus hannah GN=H2B-I P	OPHHA	11
854	11.95	11.95	44.86	24.29	17.07	tr	V8P4K3	V8P4K3_OPHHA	Selenocysteine-specific elongation factor (Fragment) OS	OPHHA	6
855	11.94	11.94	53.36	8.734	7.948	tr	V8NZ20	V8NZ20_OPHHA	DENN domain-containing protein 2C (Fragment) OS=Ophioph	OPHHA	6
856	11.92	11.92	51.03	41.03	35.86	tr	V8N717	V8N717_OPHHA	Nicotinamide phosphoribosyltransferase (Fragment) OS=Op	OPHHA	6
857	11.91	11.91	53.89	24.44	24.44	tr	V8P7P5	V8P7P5_OPHHA	Mesencephalic astrocyte-derived neurotrophic factor (Fr	OPHHA	7

858	11.89	11.89	53.56	17.85	12.21	tr|V8P9F3|V8P9F3_OPHHA	6-phosphofructokinase type C OS=Ophiophagus hannah GN=P	OPHHA	5
859	11.89	11.89	69.17	37.5	30.42	tr|V8P212|V8P212_OPHHA	Proteasome activator complex subunit 2 OS=Ophiophagus h	OPHHA	8
860	11.89	11.89	68.34	27.41	13.51	tr|V8N9Y2|V8N9Y2_OPHHA	Tropomodulin-3 (Fragment) OS=Ophiophagus hannah GN=TMOD	OPHHA	5
861	11.86	11.86	44.13	22.54	20.66	tr|V8NYN6|V8NYN6_OPHHA	Microtubule-associated protein RP/EB family member 2 (F	OPHHA	6
862	11.84	12.06	50.26	13.44	8.308	tr|V8NLM7|V8NLM7_OPHHA	U2 snRNP-associated SURP motif-containing protein OS=Op	OPHHA	6
863	11.84	11.84	28.5	18.65	14.34	tr|V8P9S8|V8P9S8_OPHHA	Beta-galactosidase-1-like protein (Fragment) OS=Ophioph	OPHHA	5
864	11.83	20.29	72.85	47.96	47.96	tr|V8N5X4|V8N5X4_OPHHA	Eukaryotic initiation factor 4A-I (Fragment) OS=Ophioph	OPHHA	12
865	11.82	12.27	52.33	24.57	14.99	tr|V8N5T1|V8N5T1_OPHHA	Amine oxidase [flavin-containing] A OS=Ophiophagus hann	OPHHA	6
866	11.82	11.82	52.77	19.5	12.7	tr|V8P1U1|V8P1U1_OPHHA	ATP-binding cassette sub-family E member 1 OS=Ophiophag	OPHHA	5
867	11.81	11.81	48.48	16.45	11.98	tr|V8P1J5|V8P1J5_OPHHA	Vacuolar protein sorting-associated protein 4A (Fragmen	OPHHA	6
868	11.8	11.8	42.71	36.68	36.68	tr|V8NNY5|V8NNY5_OPHHA	Chromobox protein-like 3 (Fragment) OS=Ophiophagus hann	OPHHA	7
869	11.78	11.78	59.35	28.05	24.8	tr|V8NDP5|V8NDP5_OPHHA	Annexin OS=Ophiophagus hannah GN=ANXA2 PE=3 SV=1	OPHHA	6
870	11.77	14	75.41	43.03	39.75	tr|V8P7Z6|V8P7Z6_OPHHA	14-3-3 protein beta/alpha OS=Ophiophagus hannah GN=YWHA	OPHHA	8
871	11.77	11.77	38.35	9.519	7.706	tr|V8NVM0|V8NVM0_OPHHA	LisH domain and HEAT repeat-containing protein (Fragmen	OPHHA	7
872	11.75	11.86	48.55	16.91	11.99	tr|V8NQL5|V8NQL5_OPHHA	Double-stranded RNA-binding protein Staufen-like 1 OS=O	OPHHA	6
873	11.75	11.75	64.07	41.36	30.17	tr|V8NZF0|V8NZF0_OPHHA	Syntenin-1 OS=Ophiophagus hannah GN=SDCBP PE=4 SV=1	OPHHA	6
874	11.74	11.74	79.6	23.68	22.17	tr|V8NZM6|V8NZM6_OPHHA	Heterogeneous nuclear ribonucleoprotein L-like protein	OPHHA	6
875	11.73	11.73	38.3	9.265	9.265	tr|V8PCQ6|V8PCQ6_OPHHA	Adipocyte enhancer-binding protein 1 (Fragment) OS=Ophi	OPHHA	7
876	11.72	11.73	43.86	6.941	6.178	tr|V8NPZ4|V8NPZ4_OPHHA	Guanylate cyclase soluble subunit beta-1 (Fragment) OS=	OPHHA	6
877	11.72	11.72	70.59	39.22	33.33	tr|V8PD47|V8PD47_OPHHA	Sorting nexin-3 OS=Ophiophagus hannah GN=SNX3 PE=4 SV=1	OPHHA	6
878	11.72	11.72	45.76	20.34	20.34	tr|V8P0C2|V8P0C2_OPHHA	Transmembrane emp24 domain-containing protein 10 (Fragm	OPHHA	6
879	11.7	11.7	55.17	20	16.9	tr|V8NNB0|V8NNB0_OPHHA	Small nuclear ribonucleoprotein Sm D2 (Fragment) OS=Oph	OPHHA	6
880	11.68	11.68	53.58	8.734	8.636	tr|V8PDA7|V8PDA7_OPHHA	DNA helicase OS=Ophiophagus hannah GN=mcm5 PE=3 SV=1	OPHHA	6
881	11.67	11.68	62.97	23.68	17.38	tr|V8P8E3|V8P8E3_OPHHA	Nucleobindin-1 OS=Ophiophagus hannah GN=Nucb1 PE=4 SV=1	OPHHA	6
882	11.66	11.67	41.44	17.5	17.5	tr|V8NH23|V8NH23_OPHHA	ATP-dependent Clp protease ATP-binding subunit clpX-lik	OPHHA	7
883	11.66	11.66	60.49	38.54	38.54	tr|V8NWQ1|V8NWQ1_OPHHA	Ras-related protein Rab-18 OS=Ophiophagus hannah GN=RAB	OPHHA	7
884	11.63	12.9	58.25	31.65	29.29	tr|V8PFE6|V8PFE6_OPHHA	Sulfurtransferase OS=Ophiophagus hannah GN=MPST PE=4 SV	OPHHA	9
885	11.63	11.63	56.48	12.96	7.673	tr|V8P004|V8P004_OPHHA	Leucine-rich repeat-containing protein 39 (Fragment) OS	OPHHA	5
886	11.61	11.61	45.35	30.23	29.94	tr|V8P4H8|V8P4H8_OPHHA	Mitotic checkpoint protein BUB3 (Fragment) OS=Ophiophag	OPHHA	7
887	11.6	11.6	57.76	11.45	11.45	tr|V8NRC0|V8NRC0_OPHHA	Nucleolar transcription factor 1 OS=Ophiophagus hannah	OPHHA	6
888	11.6	11.6	54.95	13.7	10.72	tr|V8PGV7|V8PGV7_OPHHA	Exocyst complex component 5 (Fragment) OS=Ophiophagus h	OPHHA	6
889	11.59	13.97	48.35	7.302	4.381	tr|V8NBU4|V8NBU4_OPHHA	Cytochrome protein (Fragment) OS=Ophiophagus hannah GN=	OPHHA	8
890	11.59	11.59	61.18	10.93	10.93	tr|V8P9S7|V8P9S7_OPHHA	Synaptopodin (Fragment) OS=Ophiophagus hannah GN=Synpo	OPHHA	6
891	11.59	11.59	50.32	25.95	25.95	tr|V8PFV3|V8PFV3_OPHHA	Phosphoribosyl pyrophosphate synthase-associated protei	OPHHA	7
892	11.58	11.58	59.2	37.81	37.81	tr|V8P641|V8P641_OPHHA	39S ribosomal protein L46, mitochondrial (Fragment) OS=	OPHHA	6
893	11.57	11.57	81.61	35.25	18.77	tr|V8NDR8|V8NDR8_OPHHA	Phosphatidylinositol transfer protein beta isoform (Fra	OPHHA	5
894	11.54	11.54	35.13	17.56	17.56	tr|V8P5W4|V8P5W4_OPHHA	Putative serine carboxypeptidase CPVL OS=Ophiophagus ha	OPHHA	6
895	11.53	12.12	52.35	21.63	18.18	tr|V8NVH7|V8NVH7_OPHHA	FCH domain only protein 2 (Fragment) OS=Ophiophagus han	OPHHA	6
896	11.52	11.52	44.75	15.45	8.283	tr|V8P8D1|V8P8D1_OPHHA	KN motif and ankyrin repeat domain-containing protein 4	OPHHA	6
897	11.52	11.52	53.05	16.16	16.16	tr|V8NWU9|V8NWU9_OPHHA	High mobility group protein B1 (Fragment) OS=Ophiophagu	OPHHA	7
898	11.49	11.49	41.79	21.07	21.07	tr|V8NE81|V8NE81_OPHHA	60S ribosomal protein L7a OS=Ophiophagus hannah GN=RPL7	OPHHA	6
899	11.48	11.48	46.01	16.36	12.27	tr|V8P8F7|V8P8F7_OPHHA	Myc box-dependent-interacting protein 1 (Fragment) OS=O	OPHHA	6
900	11.48	11.48	49.03	24.71	24.71	tr|V8NPW0|V8NPW0_OPHHA	15-hydroxyprostaglandin dehydrogenase [NAD+] OS=Ophioph	NAD+	8
901	11.47	11.47	47.04	7.527	6.631	tr|V8NRT2|V8NRT2_OPHHA	Apoptotic chromatin condensation inducer in the nucleus	OPHHA	6
902	11.47	11.47	37.12	14.71	7.981	tr|V8NFI7|V8NFI7_OPHHA	Serine/threonine-protein phosphatase 6 regulatory ankyr	OPHHA	5
903	11.47	11.47	30.77	7.958	7.029	tr|V8P9G9|V8P9G9_OPHHA	Dipeptidyl peptidase 4 OS=Ophiophagus hannah GN=DPP4 PE	OPHHA	6
904	11.46	11.59	48.41	18.69	12.34	tr|V8NCM0|V8NCM0_OPHHA	Importin subunit alpha OS=Ophiophagus hannah GN=KPNA6 P	OPHHA	6
905	11.46	11.46	48.88	17.41	15.63	tr|V8NXX6|V8NXX6_OPHHA	Cytoplasmic dynein 1 light intermediate chain 2 OS=Ophi	OPHHA	6
906	11.46	11.46	53.28	32.79	32.79	tr|V8NRH7|V8NRH7_OPHHA	F-actin-capping protein subunit alpha-2 OS=Ophiophagus	OPHHA	9
907	11.44	11.44	47.95	8.709	7.45	tr|V8NNP3|V8NNP3_OPHHA	Formin-like protein 3 (Fragment) OS=Ophiophagus hannah	OPHHA	6
908	11.44	11.44	36.54	11.04	8.528	tr|V8NH27|V8NH27_OPHHA	Enhancer of mRNA-decapping protein 4 (Fragment) OS=Ophi	OPHHA	6
909	11.42	11.42	38.59	6.222	4.249	tr|V8P0M7|V8P0M7_OPHHA	Dedicator of cytokinesis protein 8 (Fragment) OS=Ophiop	OPHHA	7
910	11.42	11.42	52.61	42.57	35.34	tr|V8PBB4|V8PBB4_OPHHA	Pleckstrin-likey domain-containing family F member 2 OS	OPHHA	7
911	11.41	11.41	78.41	35.23	30.11	tr|V8NMW4|V8NMW4_OPHHA	60S ribosomal protein L18a OS=Ophiophagus hannah GN=RPL	OPHHA	6
912	11.4	11.4	49.37	41.14	21.84	tr|V8PHR8|V8PHR8_OPHHA	Hexaprenyldihydroxybenzoate methyltransferase, mitochon	OPHHA	5
913	11.39	11.39	41.15	16.53	14.5	tr|V8PEM5|V8PEM5_OPHHA	2',5'-phosphodiesterase 12 (Fragment) OS=Ophiophagus ha	OPHHA	6
914	11.38	11.38	36.73	14.66	12.19	tr|V8P0W6|V8P0W6_OPHHA	Integrin alpha-1 (Fragment) OS=Ophiophagus hannah GN=It	OPHHA	6
915	11.37	11.37	60.06	23.84	16.41	tr|V8NJF8|V8NJF8_OPHHA	Protein arginine N-methyltransferase 1 (Fragment) OS=Op	OPHHA	6
916	11.37	11.37	38.53	14.48	14.48	tr|V8N972|V8N972_OPHHA	Acetolactate synthase-like protein (Fragment) OS=Ophiop	OPHHA	7
917	11.37	11.37	30.6	15.3	15.3	tr|V8NQG6|V8NQG6_OPHHA	High mobility group protein B3 (Fragment) OS=Ophiophagu	OPHHA	6
918	11.35	11.35	46.67	14.23	14.23	tr|V8NTW1|V8NTW1_OPHHA	Epidermal growth factor receptor kinase substrate 8 OS=	OPHHA	6
919	11.33	12.26	58.31	29.78	22.33	tr|V8PB23|V8PB23_OPHHA	DnaJ-like subfamily A member 1 OS=Ophiophagus hannah GN	OPHHA	7
920	11.31	11.31	47.85	24.09	24.09	tr|V8PCV2|V8PCV2_OPHHA	Epimerase family protein SDR39U1 (Fragment) OS=Ophiopha	OPHHA	6
921	11.27	13.6	50.25	34.07	17.16	tr|V8NVI6|V8NVI6_OPHHA	Septin-6 (Fragment) OS=Ophiophagus hannah GN=SEPT6 PE=3	OPHHA	7
922	11.26	11.26	53.22	13.14	10.99	tr|V8NZ44|V8NZ44_OPHHA	Prospero homeobox protein 1 (Fragment) OS=Ophiophagus h	OPHHA	6
923	11.23	11.23	50.7	10.73	4.996	tr|V8NVR9|V8NVR9_OPHHA	Laminin subunit alpha-5 OS=Ophiophagus hannah GN=LAMA5	OPHHA	6
924	11.2	11.2	45.59	12.62	10.12	tr|V8PCC6|V8PCC6_OPHHA	Exostosin-like 3 (Fragment) OS=Ophiophagus hannah GN=EX	OPHHA	6
925	11.18	11.18	46.99	16.64	10.77	tr|V8NHT5|V8NHT5_OPHHA	Putative pre-mRNA-splicing factor ATP-dependent RNA hel	OPHHA	6
926	11.18	11.18	34.23	15.34	11.65	tr|V8NU41|V8NU41_OPHHA	Threonine synthase-like 1 (Fragment) OS=Ophiophagus han	OPHHA	7
927	11.18	11.18	52.27	30.45	24.55	tr|V8NWX7|V8NWX7_OPHHA	Peptidyl-prolyl cis-trans isomerase OS=Ophiophagus hann	OPHHA	9
928	11.16	11.16	35.68	5.704	3.58	tr|V8P633|V8P633_OPHHA	Serine/threonine-protein kinase mTOR OS=Ophiophagus han	OPHHA	7
929	11.15	11.15	31.63	1.498	1.198	tr|V8NT23|V8NT23_OPHHA	Cadherin EGF LAG seven-pass G-type receptor 1 (Fragment	OPHHA	7
930	11.15	11.15	39.61	8.425	7.659	tr|V8P2Q3|V8P2Q3_OPHHA	Ataxin-2-like protein (Fragment) OS=Ophiophagus hannah	OPHHA	6
931	11.14	11.14	56.7	12.41	7.349	tr|V8N8N0|V8N8N0_OPHHA	Rho guanine nucleotide exchange factor 12 OS=Ophiophagu	OPHHA	6
932	11.14	11.14	31.91	8.547	7.407	tr|V8NZE9|V8NZE9_OPHHA	Microsomal triglyceride transfer protein large subunit	OPHHA	8
933	11.12	11.12	54.92	26.94	22.28	tr|V8NP40|V8NP40_OPHHA	Delta-aminolevulinic acid dehydratase (Fragment) OS=Oph	OPHHA	6
934	11.11	11.11	59.27	29.03	20.97	tr|V8PI20|V8PI20_OPHHA	Proteasome subunit alpha type OS=Ophiophagus hannah GN=	OPHHA	5
935	11.1	18.74	39.63	15.71	7.496	tr|V8PBG8|V8PBG8_OPHHA	Cullin-associated NEDD8-dissociated protein 1 (Fragment	OPHHA	7
936	11.1	11.3	57.64	9.807	6.029	tr|V8NLM4|V8NLM4_OPHHA	Golgin subfamily A member 3 OS=Ophiophagus hannah GN=GO	OPHHA	6
937	11.08	11.08	60.45	34.33	34.33	tr|V8NJY4|V8NJY4_OPHHA	60S ribosomal protein L9 OS=Ophiophagus hannah GN=RPL9	OPHHA	7
938	11.05	13.33	57.03	33.13	15.06	tr|V8NG26|V8NG26_OPHHA	EH domain-containing protein 4 OS=Ophiophagus hannah GN	OPHHA	6
939	11.05	11.05	37.16	8.063	5.468	tr|V8NDX1|V8NDX1_OPHHA	Protein diaphanous-like 1 (Fragment) OS=Ophiophagus han	OPHHA	5
940	11.05	11.05	79.79	34.2	30.57	tr|V8PH65|V8PH65_OPHHA	Neurocalcin-delta (Fragment) OS=Ophiophagus hannah GN=N	OPHHA	7
941	11.05	11.05	88.68	47.17	47.17	tr|V8NWV6|V8NWV6_OPHHA	Cysteine and glycine-rich protein 1 (Fragment) OS=Ophio	OPHHA	7
942	11.03	11.03	40.33	14.83	14.83	tr|V8PJT7|V8PJT7_OPHHA	Sulfite oxidase (Fragment) OS=Ophiophagus hannah GN=SUO	OPHHA	7
943	11.01	11.01	62.56	21.5	15.7	tr|V8N8N3|V8N8N3_OPHHA	Glutaminyl-tRNA synthetase (Fragment) OS=Ophiophagus ha	OPHHA	5
944	11.01	11.01	24.74	9.754	7.803	tr|V8P260|V8P260_OPHHA	MMS19 nucleotide excision repair protein-like protein (	OPHHA	6
945	10.97	11.06	34.4	16	16	tr|V8NQ39|V8NQ39_OPHHA	Transmembrane 9 superfamily member 2 (Fragment) OS=Ophi	OPHHA	7
946	10.97	10.97	37.54	11.5	11.5	tr|V8NJ76|V8NJ76_OPHHA	Disabled-like 2 (Fragment) OS=Ophiophagus hannah GN=Dab	OPHHA	6
947	10.95	11.78	33.23	16.46	8.54	tr|V8P2R2|V8P2R2_OPHHA	TBC1 domain family member 17 OS=Ophiophagus hannah GN=T	OPHHA	5
948	10.95	10.95	47.65	20.47	15.44	tr|V8P4W7|V8P4W7_OPHHA	ADP/ATP translocase 1 OS=Ophiophagus hannah GN=SLC25A4	OPHHA	5
949	10.95	10.95	52.36	30.55	24.36	tr|V8PEB5|V8PEB5_OPHHA	Acylglycerol kinase, mitochondrial (Fragment) OS=Ophiop	OPHHA	5
950	10.95	10.95	68.57	55.24	55.24	tr|V8P1H8|V8P1H8_OPHHA	Dynein light chain 2, cytoplasmic (Fragment) OS=Ophioph	OPHHA	8
951	10.92	12.17	51.42	13.21	9.375	tr|V8NWY8|V8NWY8_OPHHA	Echinoderm microtubule-associated protein-like 1 (Fragm	OPHHA	5
952	10.92	11.03	64.63	26.19	16.67	tr|V8NW74|V8NW74_OPHHA	Uncharacterized protein (Fragment) OS=Ophiophagus hanna	OPHHA	5
953	10.92	10.92	56.42	22.57	14.6	tr|V8NFN2|V8NFN2_OPHHA	Xylulose kinase (Fragment) OS=Ophiophagus hannah GN=Xyl	OPHHA	5

954	10.91	10.91	64.09	22.27	20	tr	V8PB62	V8PB62_OPHHA Peptidyl-prolyl cis-trans isomerase FKBP5 OS=Ophiophagu	OPHHA	6
955	10.91	10.91	29.71	20.42	15.92	tr	V8NEY1	V8NEY1_OPHHA Dolichyl-diphosphooligosaccharide--protein glycosyltran	OPHHA	5
956	10.9	10.9	61	12.02	8.504	tr	V8NVQ4	V8NVQ4_OPHHA Ladinin-1 OS=Ophiophagus hannah GN=Lad1 PE=4 SV=1	OPHHA	5
957	10.89	10.89	49.19	25.81	18.55	tr	V8PCP5	V8PCP5_OPHHA Macrophage-capping protein OS=Ophiophagus hannah GN=Cap	OPHHA	5
958	10.88	10.88	48.25	17.68	12.89	tr	V8PBJ9	V8PBJ9_OPHHA Methylcrotonoyl-CoA carboxylase beta chain, mitochondri	OPHHA	5
959	10.87	10.87	49.26	28.78	28.78	tr	V8P6T1	V8P6T1_OPHHA Nuclear pore glycoprotein p62 (Fragment) OS=Ophiophagus	OPHHA	6
960	10.84	18.85	67.78	67.78	63.33	tr	V8PEI6	V8PEI6_OPHHA ADP-ribosylation factor 4 OS=Ophiophagus hannah GN=ARF4	OPHHA	12
961	10.83	10.83	36.8	11.77	10.66	tr	V8NNM2	V8NNM2_OPHHA Protein enabled-like protein (Fragment) OS=Ophiophagus	OPHHA	7
962	10.82	10.84	41.37	5.325	4.207	tr	V8NL45	V8NL45_OPHHA Myotubularin-related protein 5 (Fragment) OS=Ophiophagu	OPHHA	6
963	10.82	10.83	62.58	12.05	7.928	tr	V8NNU9	V8NNU9_OPHHA Myosin-Id (Fragment) OS=Ophiophagus hannah GN=MYO1D PE=	OPHHA	6
964	10.82	10.82	42.78	10.53	6.732	tr	V8NWW4	V8NWW4_OPHHA Toll-like receptor 3 (Fragment) OS=Ophiophagus hannah G	OPHHA	5
965	10.81	10.81	41.47	13.76	11.82	tr	V8PHX6	V8PHX6_OPHHA Coiled-coil domain-containing protein 47 (Fragment) OS=	OPHHA	5
966	10.79	16.9	67.01	26.53	26.53	tr	V8PH67	V8PH67_OPHHA Heterogeneous nuclear ribonucleoprotein A3 (Fragment) O	OPHHA	9
967	10.77	15.68	61.22	37.95	26.87	tr	V8NYM0	V8NYM0_OPHHA S-adenosylmethionine synthase isoform type-2 (Fragment)	OPHHA	8
968	10.77	12.04	31.99	13.35	11.18	tr	V8N9W1	V8N9W1_OPHHA Alpha-2-macroglobulin (Fragment) OS=Ophiophagus hannah	OPHHA	6
969	10.77	10.77	50.78	24.45	21	tr	V8P1I1	V8P1I1_OPHHA Dual specificity mitogen-activated protein kinase kinas	OPHHA	5
970	10.77	10.77	37.72	35.33	30.54	tr	V8NZJ6	V8NZJ6_OPHHA Proteasome subunit beta type-2 (Fragment) OS=Ophiophagu	OPHHA	7
971	10.76	10.76	29.54	8.475	6.295	tr	V8P8D7	V8P8D7_OPHHA 1-aminocyclopropane-1-carboxylate synthase-like protein	OPHHA	5
972	10.76	10.76	34.41	16.1	15.9	tr	V8NIA0	V8NIA0_OPHHA Neutral alpha-glucosidase AB (Fragment) OS=Ophiophagus	OPHHA	6
973	10.76	10.76	81.25	62.5	51.25	tr	V8N1C7	V8N1C7_OPHHA Tropomyosin beta chain (Fragment) OS=Ophiophagus hannah	OPHHA	8
974	10.72	10.72	43.23	13.06	11.04	tr	V8PID4	V8PID4_OPHHA 5-aminolevulinate synthase (Fragment) OS=Ophiophagus ha	OPHHA	5
975	10.71	10.71	56.15	7.282	6.634	tr	V8PGN6	V8PGN6_OPHHA Cingulin-like protein 1 (Fragment) OS=Ophiophagus hanna	OPHHA	6
976	10.7	10.7	64.09	17.41	13.29	tr	V8PGX6	V8PGX6_OPHHA Protein phosphatase 1 regulatory subunit 21 (Fragment)	OPHHA	6
977	10.69	10.69	38.94	26.84	22.42	tr	V8NIJ6	V8NIJ6_OPHHA Cytochrome protein (Fragment) OS=Ophiophagus hannah GN=	OPHHA	6
978	10.66	12.53	78.89	30.15	24.62	tr	V8NYS7	V8NYS7_OPHHA GTPase KRas (Fragment) OS=Ophiophagus hannah GN=KRAS PE	OPHHA	6
979	10.66	10.66	40.32	7.111	3.576	tr	V8NXB9	V8NXB9_OPHHA Cullin-9 OS=Ophiophagus hannah GN=CUL9 PE=3 SV=1	OPHHA	7
980	10.66	10.66	71.27	32.73	28.36	tr	V8NE97	V8NE97_OPHHA Crk-like protein (Fragment) OS=Ophiophagus hannah GN=Cr	OPHHA	5
981	10.65	10.65	67.1	27.69	18.24	tr	V8PI80	V8PI80_OPHHA ELKS/Rab6-interacting/CAST family member 1 OS=Ophiophag	OPHHA	5
982	10.65	10.65	48.6	25.86	25.55	tr	V8PC36	V8PC36_OPHHA Heme oxygenase 2 (Fragment) OS=Ophiophagus hannah GN=Hm	OPHHA	5
983	10.64	10.64	69.44	20.3	15.17	tr	V8PDA4	V8PDA4_OPHHA Uncharacterized protein (Fragment) OS=Ophiophagus hanna	OPHHA	5
984	10.64	10.64	63.9	41.95	37.56	tr	V8NGZ7	V8NGZ7_OPHHA Ran-specific GTPase-activating protein (Fragment) OS=Op	OPHHA	5
985	10.64	10.64	65.14	28.57	28.57	tr	V8NYF0	V8NYF0_OPHHA Low molecular weight phosphotyrosine protein phosphatas	OPHHA	6
986	10.62	10.62	80.15	31.99	31.99	tr	V8NVP2	V8NVP2_OPHHA cAMP-dependent protein kinase type II-alpha regulatory	OPHHA	6
987	10.61	10.61	40.72	18.57	7.84	tr	V8NTM4	V8NTM4_OPHHA Tubulin--tyrosine ligase-like protein 12 (Fragment) OS=	OPHHA	4
988	10.59	10.71	58.67	22.38	14.92	tr	V8PB17	V8PB17_OPHHA WD40 repeat-containing protein SMU1 OS=Ophiophagus hann	OPHHA	6
989	10.57	13.29	39.14	16.44	14.87	tr	V8PG83	V8PG83_OPHHA Coronin OS=Ophiophagus hannah GN=Coro1b PE=3 SV=1	OPHHA	6
990	10.57	10.57	33.47	8.566	8.566	tr	V8NIS7	V8NIS7_OPHHA Glutathione reductase, mitochondrial (Fragment) OS=Ophi	OPHHA	6
991	10.56	10.56	43.41	12.52	9.683	tr	V8PG77	V8PG77_OPHHA cAMP-dependent protein kinase catalytic subunit alpha (	OPHHA	6
992	10.53	10.53	42.13	3	2.562	tr	V8PHV8	V8PHV8_OPHHA Laminin subunit alpha-3 (Fragment) OS=Ophiophagus hanna	OPHHA	6
993	10.52	10.52	60.3	24.12	15.33	tr	V8NFU6	V8NFU6_OPHHA Ornithine aminotransferase, mitochondrial OS=Ophiophagu	OPHHA	5
994	10.51	10.51	45.78	11.41	10.31	tr	V8ND09	V8ND09_OPHHA Alpha-fetoprotein (Fragment) OS=Ophiophagus hannah GN=A	OPHHA	6
995	10.49	10.49	68.35	22.78	21.01	tr	V8NQ06	V8NQ06_OPHHA [Pyruvate dehydrogenase [lipoamide]] kinase isozyme 3,	Pyruvate	5
996	10.46	10.46	57.03	19.63	15.15	tr	V8P6A7	V8P6A7_OPHHA Peptidylprolyl isomerase domain and WD repeat-containin	OPHHA	5
997	10.46	10.46	62.5	31.25	22.5	tr	V8P7L5	V8P7L5_OPHHA Clathrin light chain B (Fragment) OS=Ophiophagus hannah	OPHHA	5
998	10.45	10.45	43.93	10.47	6.769	tr	V8NSD0	V8NSD0_OPHHA Uncharacterized protein OS=Ophiophagus hannah GN=L345_0	OPHHA	4
999	10.41	10.41	58.31	13.52	8.632	tr	V8P6E1	V8P6E1_OPHHA Nexilin OS=Ophiophagus hannah GN=NEXN PE=4 SV=1	OPHHA	6
1000	10.4	10.4	69.49	57.63	57.63	tr	V8P2J2	V8P2J2_OPHHA Myotrophin OS=Ophiophagus hannah GN=MTPN PE=4 SV=1	OPHHA	7
1001	10.39	10.39	54.6	17.77	16.48	tr	V8NHK5	V8NHK5_OPHHA Fragile X mental retardation syndrome-related protein 1	OPHHA	6
1002	10.38	10.38	44.85	20.39	15.88	tr	V8PFF6	V8PFF6_OPHHA Splicing factor U2AF 65 kDa subunit OS=Ophiophagus hann	OPHHA	5
1003	10.37	10.37	45.85	12.1	6.567	tr	V8P5Q9	V8P5Q9_OPHHA Epidermal growth factor receptor substrate 15-like 1 OS	OPHHA	5
1004	10.36	10.36	54.38	22.96	19.03	tr	V8NA46	V8NA46_OPHHA Long-chain-fatty-acid--CoA ligase 1 (Fragment) OS=Ophio	OPHHA	5
1005	10.35	10.35	41.24	12.21	10.06	tr	V8POK6	V8POK6_OPHHA GRB2-associated-binding protein 1 (Fragment) OS=Ophioph	OPHHA	5
1006	10.32	10.32	28.48	28.48	23.42	tr	V8PH92	V8PH92_OPHHA Transcription factor BTF3 OS=Ophiophagus hannah GN=BTF3	OPHHA	6
1007	10.31	12.27	47.09	6.757	4.938	tr	V8P243	V8P243_OPHHA Coagulation factor V OS=Ophiophagus hannah GN=F5 PE=4 S	OPHHA	7
1008	10.3	10.3	29.73	14.23	10.63	tr	V8NHS0	V8NHS0_OPHHA WW domain-binding protein 11 (Fragment) OS=Ophiophagus	OPHHA	5
1009	10.3	10.3	49.83	19.47	16.17	tr	V8NZX6	V8NZX6_OPHHA Methionine adenosyltransferase 2 subunit beta (Fragment	OPHHA	5
1010	10.29	10.29	57.62	31.1	17.68	tr	V8NTY6	V8NTY6_OPHHA Calcium/calmodulin-dependent protein kinase type 1 OS=O	OPHHA	5
1011	10.28	14.71	37.27	14	11.58	tr	V8PD88	V8PD88_OPHHA Disks large-like 1 (Fragment) OS=Ophiophagus hannah GN=	OPHHA	7
1012	10.27	38.56	63.49	27.85	23.14	tr	V8NH89	V8NH89_OPHHA Elongation factor 2 OS=Ophiophagus hannah GN=EEF2 PE=4	OPHHA	19
1013	10.27	10.27	52.15	28.65	14.33	tr	V8NW96	V8NW96_OPHHA 1-acyl-sn-glycerol-3-phosphate acyltransferase gamma (F	OPHHA	5
1014	10.27	10.27	45.17	27.1	14.08	tr	V8NBF0	V8NBF0_OPHHA Basement membrane-specific heparan sulfate proteoglycan	OPHHA	4
1015	10.26	10.26	44.93	17.6	12.63	tr	V8P4S1	V8P4S1_OPHHA Ufm1-specific protease 2 OS=Ophiophagus hannah GN=UFSP2	OPHHA	5
1016	10.26	10.26	43.01	22.43	13.19	tr	V8NED4	V8NED4_OPHHA COP9 signalosome complex subunit 5 (Fragment) OS=Ophiop	OPHHA	6
1017	10.26	10.26	46.25	19.22	19.22	tr	V8NJ04	V8NJ04_OPHHA Toll-interacting protein (Fragment) OS=Ophiophagus hann	OPHHA	6
1018	10.25	10.25	42.01	18.26	17.35	tr	V8PGD5	V8PGD5_OPHHA Uncharacterized protein (Fragment) OS=Ophiophagus hanna	OPHHA	6
1019	10.24	10.24	60.38	29.06	18.11	tr	V8NDS4	V8NDS4_OPHHA Transmembrane emp24 domain-containing protein 7 (Fragme	OPHHA	5
1020	10.21	10.21	44.8	19.89	13.8	tr	V8NXB2	V8NXB2_OPHHA WD repeat-containing protein 26 (Fragment) OS=Ophiophag	OPHHA	5
1021	10.21	10.21	53.2	23.23	19.53	tr	V8NUX4	V8NUX4_OPHHA Tetratricopeptide repeat protein 35 OS=Ophiophagus hann	OPHHA	5
1022	10.2	10.21	48.24	32.94	32.94	tr	V8PFF0	V8PFF0_OPHHA Glutamate--cysteine ligase regulatory subunit OS=Ophiop	OPHHA	5
1023	10.2	10.2	60	30.37	30.37	tr	V8P1A5	V8P1A5_OPHHA Alpha-aminoadipic semialdehyde dehydrogenase (Fragment)	OPHHA	9
1024	10.19	10.19	62.84	10.14	7.239	tr	V8P6N4	V8P6N4_OPHHA Sister chromatid cohesion protein PDS5-like B (Fragment	OPHHA	5
1025	10.19	10.19	47.64	13.21	7.547	tr	V8NPB4	V8NPB4_OPHHA Nucleoporin p54 (Fragment) OS=Ophiophagus hannah GN=NUP	OPHHA	5
1026	10.18	10.18	44.79	17.08	15	tr	V8NTB8	V8NTB8_OPHHA Cleavage stimulation factor subunit 1 (Fragment) OS=Oph	OPHHA	5
1027	10.17	10.17	35.98	9.382	7.395	tr	V8NZI0	V8NZI0_OPHHA Serine/threonine-protein kinase Nek9 (Fragment) OS=Ophi	OPHHA	5
1028	10.17	10.17	52.16	19.85	17.3	tr	V8P043	V8P043_OPHHA Transmembrane protein 43 OS=Ophiophagus hannah GN=Tmem4	OPHHA	6
1029	10.16	10.16	52.35	19.71	17.06	tr	V8NAX3	V8NAX3_OPHHA Beta-1,4-galactosyltransferase 1 OS=Ophiophagus hannah	OPHHA	5
1030	10.14	10.14	60.91	28.81	28.81	tr	V8NJA6	V8NJA6_OPHHA rRNA 2'-O-methyltransferase fibrillarin OS=Ophiophagus	OPHHA	8
1031	10.14	10.14	45.28	26.95	20.49	tr	V8PGR8	V8PGR8_OPHHA [Pyruvate dehydrogenase [lipoamide]] kinase isozyme 2,	Pyruvate	5
1032	10.13	12.44	62.35	25.67	17.85	tr	V8NEK3	V8NEK3_OPHHA Ras GTPase-activating-like protein IQGAP3 (Fragment) OS	OPHHA	8
1033	10.13	10.13	43.09	23.58	17.07	tr	V8NZW7	V8NZW7_OPHHA Prolargin OS=Ophiophagus hannah GN=Prelp PE=4 SV=1	OPHHA	8
1034	10.13	10.13	38.98	12.47	10.24	tr	V8P672	V8P672_OPHHA Sequestosome-1 (Fragment) OS=Ophiophagus hannah GN=SQST	OPHHA	5
1035	10.12	10.28	49.59	35.66	31.97	tr	V8NFP1	V8NFP1_OPHHA Adapter molecule crk (Fragment) OS=Ophiophagus hannah G	OPHHA	6
1036	10.12	10.17	34.99	17.97	17.97	tr	V8P1A7	V8P1A7_OPHHA Kynurenine/alpha-aminoadipate aminotransferase, mitocho	OPHHA	5
1037	10.09	10.09	40.58	9.682	5.504	tr	V8NXX4	V8NXX4_OPHHA Tensin-like C1 domain-containing phosphatase (Fragment)	OPHHA	5
1038	10.08	10.09	43.65	6.781	3.375	tr	V8N9B6	V8N9B6_OPHHA Epiplakin (Fragment) OS=Ophiophagus hannah GN=Eppk1 PE=	OPHHA	7
1039	10.07	10.07	31.9	9.348	4.803	tr	V8PBP3	V8PBP3_OPHHA Elongator complex protein 1 (Fragment) OS=Ophiophagus h	OPHHA	4
1040	10.06	10.06	46.86	20.42	17.28	tr	V8NZW4	V8NZW4_OPHHA E3 ubiquitin-protein ligase ARIH1 (Fragment) OS=Ophioph	OPHHA	6
1041	10.05	10.05	66.18	44.85	44.85	tr	V8PAR8	V8PAR8_OPHHA 60S ribosomal protein L27 OS=Ophiophagus hannah GN=RPL2	OPHHA	6
1042	10.04	16.72	69.26	28.79	28.79	tr	V8NL10	V8NL10_OPHHA Ras-related protein Rap-1A OS=Ophiophagus hannah GN=RAP	OPHHA	8
1043	10.04	10.04	51.92	36.86	23.4	tr	V8NIR0	V8NIR0_OPHHA V-type proton ATPase subunit d 1 (Fragment) OS=Ophiopha	OPHHA	4
1044	10.03	12.4	46.05	13.66	8.878	tr	V8NT65	V8NT65_OPHHA Putative G-protein coupled receptor 21 OS=Ophiophagus h	OPHHA	7
1045	10.02	10.02	59.07	24.89	24.89	tr	V8NQN9	V8NQN9_OPHHA Peptidyl-prolyl cis-trans isomerase FKBP8 (Fragment) OS	OPHHA	5
1046	10.02	10.02	67.35	23.67	23.67	tr	V8NYX1	V8NYX1_OPHHA NADH dehydrogenase [ubiquinone] flavoprotein 2, mitocho	OPHHA	6
1047	10.01	10.08	31.06	15.32	15.32	tr	V8NQU9	V8NQU9_OPHHA Putative ubiquitin carboxyl-terminal hydrolase FAF-X (F	OPHHA	6
1048	10.01	10.01	58.8	21.72	21.72	tr	V8NY86	V8NY86_OPHHA Electron transfer flavoprotein subunit alpha, mitochond	OPHHA	10
1049	10	10.17	52.77	20.84	19.26	tr	V8P0R6	V8P0R6_OPHHA Heterogeneous nuclear ribonucleoprotein H3 OS=Ophiophag	OPHHA	5

1050	10	10.05	34.3	19.83	19.83	tr	V8NDL5	V8NDL5_OPHHA Uncharacterized protein (Fragment) OS=Ophiophagus hanna	OPHHA	8
1051	10	10.04	42.83	6.528	6.528	tr	V8PB93	V8PB93_OPHHA Coiled-coil and C2 domain-containing protein 1A OS=Ophi	OPHHA	5
1052	10	10	62.45	45.57	36.71	tr	V8P3W5	V8P3W5_OPHHA Transcriptional activator protein Pur-alpha (Fragment)	OPHHA	5
1053	10	10	46.15	21.28	21.28	tr	V8NC88	V8NC88_OPHHA Protein NDRG1 (Fragment) OS=Ophiophagus hannah GN=Ndrg1	OPHHA	10
1054	10	10	12.91	4.719	4.719	tr	V8NRB7	V8NRB7_OPHHA Nascent polypeptide-associated complex subunit alpha, m	OPHHA	6
1055	10	10	77.71	41.57	41.57	tr	V8PD17	V8PD17_OPHHA Protein canopy-like 2 (Fragment) OS=Ophiophagus hannah	OPHHA	5
1056	10	10	20.39	11.78	11.78	tr	V8NIS5	V8NIS5_OPHHA Ubiquilin-4 (Fragment) OS=Ophiophagus hannah GN=UBQLN4	OPHHA	5
1057	10	10	83.33	71.93	71.93	tr	V8NA13	V8NA13_OPHHA Uncharacterized protein (Fragment) OS=Ophiophagus hanna	OPHHA	6
1058	10	10	35.09	35.09	35.09	tr	V8NE16	V8NE16_OPHHA Cell division control protein 42-like protein OS=Ophiop	OPHHA	5
1059	9.98	10	47.98	16.83	11.68	tr	V8PGL4	V8PGL4_OPHHA Glycerol-3-phosphate dehydrogenase (Fragment) OS=Ophiop	OPHHA	5
1060	9.98	10	57.19	30.48	20.55	tr	V8N9B0	V8N9B0_OPHHA Glycerol kinase OS=Ophiophagus hannah GN=Gk PE=4 SV=1	OPHHA	5
1061	9.98	9.98	37.05	3.929	3.047	tr	V8PGR1	V8PGR1_OPHHA Talin-2 (Fragment) OS=Ophiophagus hannah GN=TLN2 PE=4 S	OPHHA	5
1062	9.96	9.96	64.31	6.403	6.403	tr	V8P7W2	V8P7W2_OPHHA Leucine zipper protein 1 (Fragment) OS=Ophiophagus hann	OPHHA	6
1063	9.96	9.96	37.36	16.76	14.84	tr	V8NGJ3	V8NGJ3_OPHHA 3-hydroxybutyrate dehydrogenase type 2 (Fragment) OS=Op	OPHHA	5
1064	9.95	9.95	62.82	19.55	16.03	tr	V8P8X3	V8P8X3_OPHHA Oxidoreductase HTATIP2 (Fragment) OS=Ophiophagus hannah	OPHHA	5
1065	9.92	9.92	54.8	13.18	9.975	tr	V8NFD1	V8NFD1_OPHHA Rab3 GTPase-activating protein catalytic subunit (Fragm	OPHHA	6
1066	9.91	9.91	43.54	10.31	7.818	tr	V8NSV1	V8NSV1_OPHHA SLIT-ROBO Rho GTPase-activating protein 2 OS=Ophiophagu	OPHHA	5
1067	9.9	9.9	47.04	7.928	6.66	tr	V8NAC4	V8NAC4_OPHHA Myosin-If OS=Ophiophagus hannah GN=MYO1F PE=4 SV=1	OPHHA	5
1068	9.9	9.9	52.12	12.22	9.726	tr	V8NVH9	V8NVH9_OPHHA Basic leucine zipper and W2 domain-containing protein 1	OPHHA	5
1069	9.9	9.9	66.17	35.34	17.67	tr	V8P5J2	V8P5J2_OPHHA ADP-ribosylation factor-like protein 8A (Fragment) OS=O	OPHHA	5
1070	9.89	9.89	58.24	24.18	20.51	tr	V8P9B8	V8P9B8_OPHHA Arf-GAP with dual PH domain-containing protein 1 (Fragm	OPHHA	5
1071	9.88	9.88	53.68	8.655	7.836	tr	V8P7D0	V8P7D0_OPHHA Ras GTPase-activating protein 1 (Fragment) OS=Ophiophag	OPHHA	5
1072	9.88	9.88	53.34	13.5	8.422	tr	V8PG40	V8PG40_OPHHA Lon protease homolog (Fragment) OS=Ophiophagus hannah G	OPHHA	5
1073	9.88	9.88	33	11.4	7.8	tr	V8NLA0	V8NLA0_OPHHA Heparin cofactor 2 (Fragment) OS=Ophiophagus hannah GN=	OPHHA	6
1074	9.88	9.88	49.45	22.18	16.36	tr	V8P0G1	V8P0G1_OPHHA COP9 signalosome complex subunit 7a (Fragment) OS=Ophio	OPHHA	5
1075	9.87	9.87	43.33	11.45	11.3	tr	V8PCW5	V8PCW5_OPHHA Calcium/calmodulin-dependent protein kinase type II sub	OPHHA	5
1076	9.87	9.87	47.12	19.11	14.66	tr	V8P6R7	V8P6R7_OPHHA Golgi resident protein GCP60 (Fragment) OS=Ophiophagus	OPHHA	6
1077	9.86	9.86	65.85	37.98	29.97	tr	V8P1U9	V8P1U9_OPHHA Axin interactor, dorsalization-associated protein OS=Op	OPHHA	6
1078	9.85	9.85	50.38	20.46	20.46	tr	V8P7A6	V8P7A6_OPHHA SWI/SNF-related matrix-associated actin-dependent regul	OPHHA	6
1079	9.85	9.85	69.15	23.4	22.87	tr	V8N7G8	V8N7G8_OPHHA ATP synthase subunit gamma, mitochondrial (Fragment) OS	OPHHA	7
1080	9.81	9.81	38.81	14.93	13.22	tr	V8NHC7	V8NHC7_OPHHA Puromycin-sensitive aminopeptidase (Fragment) OS=Ophiop	OPHHA	8
1081	9.8	9.8	40.52	5.043	3.668	tr	V8NBP2	V8NBP2_OPHHA Transcriptional repressor CTCF (Fragment) OS=Ophiophagu	OPHHA	5
1082	9.79	9.79	47.84	24.46	15.29	tr	V8N597	V8N597_OPHHA Glutamate carboxypeptidase 2 (Fragment) OS=Ophiophagus	OPHHA	5
1083	9.79	9.79	31.94	17.37	17.17	tr	V8P6L6	V8P6L6_OPHHA Golgi reassembly-stacking protein 2 (Fragment) OS=Ophio	OPHHA	5
1084	9.77	9.77	62.07	35.47	21.18	tr	V8P8S4	V8P8S4_OPHHA 60S ribosomal protein L13a OS=Ophiophagus hannah GN=RPL	OPHHA	5
1085	9.77	9.77	69.12	55.15	48.53	tr	V8NG47	V8NG47_OPHHA Ubiquitin-conjugating enzyme E2 L3 OS=Ophiophagus hanna	OPHHA	5
1086	9.76	9.76	63.18	17.81	10.93	tr	V8P470	V8P470_OPHHA Pre-mRNA-splicing factor RBM22 (Fragment) OS=Ophiophagu	OPHHA	5
1087	9.76	9.76	43.24	17.3	17.3	tr	V8NUM4	V8NUM4_OPHHA 3-hydroxyanthranilate 3,4-dioxygenase OS=Ophiophagus ha	OPHHA	7
1088	9.74	9.74	30.14	19.39	13.55	tr	V8N830	V8N830_OPHHA Tetratricopeptide repeat protein 39B (Fragment) OS=Ophi	OPHHA	5
1089	9.74	9.74	41.4	32.36	27.41	tr	V8NST7	V8NST7_OPHHA Geranylgeranyl transferase type-2 subunit alpha (Fragme	OPHHA	6
1090	9.73	9.73	73.64	59.09	50.91	tr	V8NJ67	V8NJ67_OPHHA Neutral alpha-glucosidase AB (Fragment) OS=Ophiophagus	OPHHA	7
1091	9.72	9.72	48.43	17.66	17.66	tr	V8P772	V8P772_OPHHA Hsc70-interacting protein OS=Ophiophagus hannah GN=ST13	OPHHA	5
1092	9.71	9.71	47.1	18.55	9.355	tr	V8P4E6	V8P4E6_OPHHA Neurolysin, mitochondrial OS=Ophiophagus hannah GN=NLN	OPHHA	4
1093	9.7	9.7	45.54	20.06	11.78	tr	V8P1I4	V8P1I4_OPHHA Proteasome subunit alpha type-1 (Fragment) OS=Ophiophag	OPHHA	7
1094	9.69	9.76	43.17	8.314	8.314	tr	V8NLZ2	V8NLZ2_OPHHA Canalicular multispecific organic anion transporter 2 (	OPHHA	5
1095	9.68	10.46	40.02	10.78	7.559	tr	V8P546	V8P546_OPHHA Conserved oligomeric Golgi complex subunit 1 (Fragment)	OPHHA	5
1096	9.68	9.68	53.02	31.03	27.16	tr	V8NNI0	V8NNI0_OPHHA Coiled-coil domain-containing protein OS=Ophiophagus ha	OPHHA	9
1097	9.67	9.67	49.82	18.6	18.6	tr	V8NNA3	V8NNA3_OPHHA 2,4-dienoyl-CoA reductase, mitochondrial OS=Ophiophagus	OPHHA	7
1098	9.66	9.66	37.87	7.534	5.479	tr	V8PF71	V8PF71_OPHHA Integrin alpha-3 (Fragment) OS=Ophiophagus hannah GN=It	OPHHA	5
1099	9.66	9.66	60.85	20.11	18.25	tr	V8P6I2	V8P6I2_OPHHA Adipocyte plasma membrane-associated protein (Fragment)	OPHHA	8
1100	9.65	9.65	39.34	16.39	14.21	tr	V8P2J9	V8P2J9_OPHHA Mitogen-activated protein kinase 9 (Fragment) OS=Ophiop	OPHHA	5
1101	9.65	9.65	58.96	39.15	33.96	tr	V8P2U4	V8P2U4_OPHHA Dihydropyrimidine dehydrogenase [NADP+] (Fragment) OS=O	NADP+	5
1102	9.62	9.96	64.43	5.023	2.91	tr	V8NRZ7	V8NRZ7_OPHHA Golgin subfamily B member 1 OS=Ophiophagus hannah GN=GO	OPHHA	6
1103	9.62	9.62	37.68	13.37	8.535	tr	V8NP36	V8NP36_OPHHA NEDD8 ultimate buster 1 (Fragment) OS=Ophiophagus hanna	OPHHA	4
1104	9.62	9.62	37.87	25.6	25.6	tr	V8NBA3	V8NBA3_OPHHA Ubiquitin-like modifier-activating enzyme 5 OS=Ophiopha	OPHHA	5
1105	9.6	9.6	40.8	19.4	15.67	tr	V8PAV4	V8PAV4_OPHHA Arfaptin-1 OS=Ophiophagus hannah GN=ARFIP1 PE=4 SV=1	OPHHA	5
1106	9.58	11.97	61.36	13.45	11.59	tr	V8NLF1	V8NLF1_OPHHA Rho-associated protein kinase 1 (Fragment) OS=Ophiophag	OPHHA	7
1107	9.58	9.58	49.55	18.75	12.28	tr	V8PI44	V8PI44_OPHHA Gamma-butyrobetaine dioxygenase (Fragment) OS=Ophiophag	OPHHA	5
1108	9.57	9.57	53.88	18.93	18.93	tr	V8PAF7	V8PAF7_OPHHA Ras-related protein Ral-B OS=Ophiophagus hannah GN=RALB	OPHHA	5
1109	9.55	9.55	49.79	31.22	31.22	tr	V8PF10	V8PF10_OPHHA 3-hydroxyacyl-CoA dehydrogenase type-2 OS=Ophiophagus h	OPHHA	6
1110	9.51	9.51	86.07	55.74	55.74	tr	V8P885	V8P885_OPHHA Mitochondrial peptide methionine sulfoxide reductase (F	OPHHA	5
1111	9.5	9.5	88.18	25	25	tr	V8NDE5	V8NDE5_OPHHA Protein CDV3-like protein (Fragment) OS=Ophiophagus han	OPHHA	5
1112	9.49	9.49	43.97	17.59	11.45	tr	V8NNU3	V8NNU3_OPHHA 60 kDa SS-A/Ro ribonucleoprotein (Fragment) OS=Ophiopha	OPHHA	4
1113	9.48	9.48	33.62	7.866	6.789	tr	V8NIV0	V8NIV0_OPHHA Periodic tryptophan protein 2-like protein (Fragment) O	OPHHA	4
1114	9.47	9.47	51.14	11.14	11.14	tr	V8NXA3	V8NXA3_OPHHA Ephexin-1 (Fragment) OS=Ophiophagus hannah GN=NGEF PE=4	OPHHA	5
1115	9.47	9.47	42.16	18.38	11.8	tr	V8NJR5	V8NJR5_OPHHA SUMO-activating enzyme subunit 2 (Fragment) OS=Ophiopha	OPHHA	5
1116	9.47	9.47	61.26	20.95	20.95	tr	V8PGA4	V8PGA4_OPHHA DnaJ-like subfamily A member 2 (Fragment) OS=Ophiophagu	OPHHA	5
1117	9.46	9.46	48.42	19.72	10.04	tr	V8NUT2	V8NUT2_OPHHA Beta-galactosidase (Fragment) OS=Ophiophagus hannah GN=	OPHHA	4
1118	9.45	9.45	34.67	17.11	15.11	tr	V8NU48	V8NU48_OPHHA Tubulin gamma chain (Fragment) OS=Ophiophagus hannah GN	OPHHA	5
1119	9.44	9.44	49.59	4.508	3.864	tr	V8PF55	V8PF55_OPHHA Nicotinamide N-methyltransferase (Fragment) OS=Ophiopha	OPHHA	7
1120	9.43	9.43	82.57	51.38	47.71	tr	V8P9P7	V8P9P7_OPHHA Uncharacterized protein (Fragment) OS=Ophiophagus hanna	OPHHA	10
1121	9.4	9.4	44.65	33.49	21.86	tr	V8NA53	V8NA53_OPHHA Tricarboxylate transport protein, mitochondrial (Fragme	OPHHA	4
1122	9.4	9.4	38.76	25.73	23.45	tr	V8NL08	V8NL08_OPHHA [Protein ADP-ribosylarginine] hydrolase (Fragment) OS=O	Protein /	5
1123	9.36	9.36	42	8.243	7.556	tr	V8NS56	V8NS56_OPHHA Complement component C6 (Fragment) OS=Ophiophagus hanna	OPHHA	5
1124	9.36	9.36	29.71	11.21	11.21	tr	V8PC05	V8PC05_OPHHA Carbonic anhydrase 14 (Fragment) OS=Ophiophagus hannah	OPHHA	5
1125	9.36	9.36	39.88	24.86	21.39	tr	V8NJ50	V8NJ50_OPHHA Enoyl-CoA delta isomerase 2, mitochondrial (Fragment) O	OPHHA	6
1126	9.35	9.35	69.85	22.3	19.61	tr	V8NUE1	V8NUE1_OPHHA ADP-ribosylation factor GTPase-activating protein 3 OS=	OPHHA	6
1127	9.35	9.35	36.59	12.04	7.165	tr	V8NKJ4	V8NKJ4_OPHHA Palladin (Fragment) OS=Ophiophagus hannah GN=PALLD PE=4	OPHHA	4
1128	9.33	9.33	50.96	18.26	12.74	tr	V8N966	V8N966_OPHHA FAD synthase OS=Ophiophagus hannah GN=flad1 PE=4 SV=1	OPHHA	4
1129	9.32	9.32	34.61	6.435	5.565	tr	V8NVE0	V8NVE0_OPHHA Integrin alpha-6 (Fragment) OS=Ophiophagus hannah GN=IT	OPHHA	5
1130	9.31	9.31	35.75	14.25	11.25	tr	V8NMK4	V8NMK4_OPHHA Cytochrome protein (Fragment) OS=Ophiophagus hannah GN=	OPHHA	5
1131	9.31	9.31	85.85	62.26	62.26	tr	V8P334	V8P334_OPHHA Peptidyl-prolyl cis-trans isomerase NIMA-interacting 1	OPHHA	5
1132	9.29	9.29	56.67	18.15	15.56	tr	V8NJT5	V8NJT5_OPHHA Regulator of microtubule dynamics protein 1 (Fragment)	OPHHA	5
1133	9.28	9.28	67.47	45.78	45.78	tr	V8N9Z2	V8N9Z2_OPHHA 40S ribosomal protein S21 OS=Ophiophagus hannah GN=RPS2	OPHHA	6
1134	9.26	9.26	42.15	12.65	7.728	tr	V8NEI2	V8NEI2_OPHHA Elongation factor 1-gamma OS=Ophiophagus hannah GN=EEF1	OPHHA	5
1135	9.26	9.26	23.78	14.23	14.23	tr	V8P1B3	V8P1B3_OPHHA Transmembrane 9 superfamily member 3 OS=Ophiophagus han	OPHHA	6
1136	9.23	10.14	40.42	10.53	8.626	tr	V8PFG1	V8PFG1_OPHHA Ubiquitin-protein ligase E3C (Fragment) OS=Ophiophagus	OPHHA	6
1137	9.23	9.23	44.94	7.23	7.23	tr	V8NCV9	V8NCV9_OPHHA Cytochrome protein OS=Ophiophagus hannah GN=CYP1A5 PE=4	OPHHA	6
1138	9.23	9.23	63.66	20.94	13.55	tr	V8P6T4	V8P6T4_OPHHA G patch domain and KOW motifs-containing protein OS=Oph	OPHHA	4
1139	9.22	9.23	46.74	9.309	6.046	tr	V8NR95	V8NR95_OPHHA Exocyst complex component 1 (Fragment) OS=Ophiophagus h	OPHHA	4
1140	9.22	9.22	41.74	11.41	9.933	tr	V8P4G1	V8P4G1_OPHHA Oxysterol-binding protein OS=Ophiophagus hannah GN=OSBP	OPHHA	5
1141	9.22	9.22	48.98	17.01	12.5	tr	V8PCU4	V8PCU4_OPHHA ADP-ribosylation factor GTPase-activating protein 2 (Fr	OPHHA	5
1142	9.21	9.21	55.74	23.83	17.87	tr	V8P358	V8P358_OPHHA 60S ribosomal protein L17 (Fragment) OS=Ophiophagus han	OPHHA	5
1143	9.19	9.19	43.97	8.906	5.975	tr	V8P5G7	V8P5G7_OPHHA Lymphocyte antigen 75 (Fragment) OS=Ophiophagus hannah	OPHHA	4
1144	9.19	9.19	64.45	32.7	29.38	tr	V8NI36	V8NI36_OPHHA Eukaryotic translation initiation factor 4E (Fragment)	OPHHA	6
1145	9.18	9.18	59.37	25.94	14.12	tr	V8P218	V8P218_OPHHA Eukaryotic translation initiation factor 3 subunit H OS	OPHHA	4

1146	9.17	9.17	40.71	4.667	4.667	tr	V8P5V6	V8P5V6_OPHHA	Elongation factor G, mitochondrial OS=Ophiophagus hanna	OPHHA	5
1147	9.15	10.13	41.48	5.89	4.574	tr	V8NX99	V8NX99_OPHHA	Neutral alpha-glucosidase C (Fragment) OS=Ophiophagus h	OPHHA	6
1148	9.14	9.14	47.48	18.83	18.83	tr	V8P4X0	V8P4X0_OPHHA	Ubiquitin-conjugating enzyme E2 variant 3 (Fragment) OS	OPHHA	5
1149	9.12	9.12	78.01	23.56	23.56	tr	V8P2T8	V8P2T8_OPHHA	Adenylate kinase 2, mitochondrial OS=Ophiophagus hannah	OPHHA	6
1150	9.1	9.1	39.16	18.53	18.18	tr	V8P9X8	V8P9X8_OPHHA	S-formylglutathione hydrolase (Fragment) OS=Ophiophagus	OPHHA	5
1151	9.09	9.12	35.32	8.66	6.36	tr	V8NHA5	V8NHA5_OPHHA	Glycogen [starch] synthase, muscle OS=Ophiophagus hanna	OPHHA	4
1152	9.09	9.09	50.16	18.47	10.19	tr	V8NF47	V8NF47_OPHHA	Ankyrin-1 OS=Ophiophagus hannah GN=ANK1 PE=4 SV=1	OPHHA	4
1153	9.07	9.07	50.97	6.395	3.391	tr	V8P992	V8P992_OPHHA	Interferon-induced helicase C domain-containing protein	OPHHA	4
1154	9.07	9.07	39.55	21.64	21.64	tr	V8NRD3	V8NRD3_OPHHA	5'-AMP-activated protein kinase catalytic subunit alpha	OPHHA	5
1155	9.06	9.06	39.9	15.15	15.15	tr	V8NZA8	V8NZA8_OPHHA	Prostaglandin reductase 2 (Fragment) OS=Ophiophagus han	OPHHA	5
1156	9.05	9.05	45.68	13.53	11.47	tr	V8NFR6	V8NFR6_OPHHA	Epidermal growth factor receptor OS=Ophiophagus hannah	OPHHA	5
1157	9.04	15.07	67.45	30.54	27.85	tr	V8P7Q2	V8P7Q2_OPHHA	GTP-binding protein SAR1b (Fragment) OS=Ophiophagus han	OPHHA	9
1158	9.03	9.03	55.26	19.12	11.4	tr	V8NQ87	V8NQ87_OPHHA	BAG family molecular chaperone regulator 3 OS=Ophiophag	OPHHA	4
1159	9.03	9.03	46.77	12.69	12.69	tr	V8P8S1	V8P8S1_OPHHA	U1 small nuclear ribonucleoprotein 70 kDa (Fragment) OS	OPHHA	5
1160	9.02	9.02	56.18	14.54	11.16	tr	V8NFC4	V8NFC4_OPHHA	Rho guanine nucleotide exchange factor 1 OS=Ophiophagus	OPHHA	5
1161	9.02	9.02	66.93	19.79	12.76	tr	V8P5T0	V8P5T0_OPHHA	Short/branched chain specific acyl-CoA dehydrogenase, m	OPHHA	5
1162	9	9	52.46	10.15	8.769	tr	V8PFI8	V8PFI8_OPHHA	Cullin-2 (Fragment) OS=Ophiophagus hannah GN=CUL2 PE=3	OPHHA	5
1163	8.99	9.05	57.08	11.92	9.677	tr	V8PJC2	V8PJC2_OPHHA	Transcription initiation factor IIB OS=Ophiophagus hann	OPHHA	6
1164	8.99	8.99	55.86	24.32	24.32	tr	V8PE61	V8PE61_OPHHA	Serine/arginine-rich splicing factor 6 OS=Ophiophagus h	OPHHA	8
1165	8.98	8.98	45.91	19.74	10.43	tr	V8P6I8	V8P6I8_OPHHA	Cytoplasmic dynein 1 intermediate chain 2 (Fragment) OS	OPHHA	4
1166	8.98	8.98	58.45	50	32.39	tr	V8N6Y1	V8N6Y1_OPHHA	Phosphatidylethanolamine-binding protein 1 (Fragment) O	OPHHA	4
1167	8.97	8.97	58.35	6.357	6.357	tr	V8NIQ8	V8NIQ8_OPHHA	Replication factor C subunit 1 (Fragment) OS=Ophiophagu	OPHHA	5
1168	8.96	8.96	42.52	9.979	5.509	tr	V8NQ84	V8NQ84_OPHHA	Papilin (Fragment) OS=Ophiophagus hannah GN=PAPLN PE=4	OPHHA	5
1169	8.95	8.95	59.7	16.67	16.67	tr	V8NJF6	V8NJF6_OPHHA	DnaJ-like subfamily C member 11 (Fragment) OS=Ophiophag	OPHHA	5
1170	8.95	8.95	43.5	23.16	17.51	tr	V8P7N3	V8P7N3_OPHHA	Protein FAM45A OS=Ophiophagus hannah GN=FAM45A PE=4 SV=	OPHHA	5
1171	8.94	8.98	53.52	12.74	6.897	tr	V8PJL3	V8PJL3_OPHHA	Serine/threonine-protein kinase N2 (Fragment) OS=Ophiop	OPHHA	4
1172	8.94	8.94	35.84	8.117	5.786	tr	V8NMT5	V8NMT5_OPHHA	Apoptotic protease-activating factor 1 OS=Ophiophagus h	OPHHA	5
1173	8.94	8.94	44.61	22.68	20.45	tr	V8N4R8	V8N4R8_OPHHA	Uncharacterized protein OS=Ophiophagus hannah GN=L345_1	OPHHA	6
1174	8.93	8.93	36.91	18.06	15.45	tr	V8NS44	V8NS44_OPHHA	LanC-like protein 1 OS=Ophiophagus hannah GN=LANCL1 PE=	OPHHA	5
1175	8.91	8.91	43.78	10.04	6.747	tr	V8NJK7	V8NJK7_OPHHA	Golgi integral membrane protein 4 (Fragment) OS=Ophioph	OPHHA	4
1176	8.91	8.91	56.3	40.74	29.63	tr	V8PBR9	V8PBR9_OPHHA	60S ribosomal protein L32 OS=Ophiophagus hannah GN=RPL3	OPHHA	4
1177	8.9	8.9	69.44	20.83	13.66	tr	V8NSR1	V8NSR1_OPHHA	60S ribosomal protein L3 (Fragment) OS=Ophiophagus hann	OPHHA	4
1178	8.88	11.12	82.4	37.77	25.75	tr	V8PF48	V8PF48_OPHHA	14-3-3 protein gamma (Fragment) OS=Ophiophagus hannah G	OPHHA	6
1179	8.87	8.98	42.77	6.423	5.766	tr	V8NWE5	V8NWE5_OPHHA	COP9 signalosome complex subunit 2 (Fragment) OS=Ophiop	OPHHA	6
1180	8.87	8.89	54.78	13.04	12.61	tr	V8NFT6	V8NFT6_OPHHA	Glutathione S-transferase OS=Ophiophagus hannah GN=L345	OPHHA	4
1181	8.86	10.97	57.76	18.22	13.25	tr	V8PH87	V8PH87_OPHHA	Dihydropyrimidinase (Fragment) OS=Ophiophagus hannah GN	OPHHA	6
1182	8.86	8.87	36.76	13.38	9.296	tr	V8PBA8	V8PBA8_OPHHA	RalBP1-associated Eps domain-containing protein 1 OS=Op	OPHHA	6
1183	8.86	8.86	42.75	13.53	13.53	tr	V8NLU3	V8NLU3_OPHHA	BRISC complex subunit Abro1 OS=Ophiophagus hannah GN=FA	OPHHA	5
1184	8.85	8.86	49.87	4.068	4.068	tr	V8NN36	V8NN36_OPHHA	Hydroxyindole O-methyltransferase (Fragment) OS=Ophioph	OPHHA	6
1185	8.85	8.85	48.82	7.649	6.637	tr	V8NRD2	V8NRD2_OPHHA	Phosphoinositide phospholipase C (Fragment) OS=Ophiopha	OPHHA	5
1186	8.85	8.85	42.25	23.47	23.47	tr	V8NPQ0	V8NPQ0_OPHHA	Enoyl-CoA delta isomerase 2, mitochondrial OS=Ophiophag	OPHHA	6
1187	8.84	8.85	47.69	14.97	11.46	tr	V8NMM0	V8NMM0_OPHHA	Mitochondrial Rho GTPase 2 OS=Ophiophagus hannah GN=RHO	OPHHA	6
1188	8.84	8.85	47.19	12.19	12.19	tr	V8PFW5	V8PFW5_OPHHA	Collagen alpha-1(IV) chain OS=Ophiophagus hannah GN=COL	OPHHA	5
1189	8.84	8.85	43.5	10.77	10.77	tr	V8NJP6	V8NJP6_OPHHA	KIF1-binding protein-like protein (Fragment) OS=Ophioph	OPHHA	5
1190	8.83	8.83	19.33	7.692	6.781	tr	V8N8H0	V8N8H0_OPHHA	Nuclear pore complex protein Nup98-Nup96 OS=Ophiophagus	OPHHA	4
1191	8.83	8.83	34.53	16.21	10.53	tr	V8P0J2	V8P0J2_OPHHA	Dihydrolipoyllysine-residue succinyltransferase compone	OPHHA	6
1192	8.82	8.82	58.52	26.92	20.05	tr	V8NH71	V8NH71_OPHHA	Guanine nucleotide-binding protein subunit alpha-11 OS=	OPHHA	5
1193	8.81	8.81	38.47	12.52	9.223	tr	V8P1F8	V8P1F8_OPHHA	HBS1-like protein (Fragment) OS=Ophiophagus hannah GN=H	OPHHA	5
1194	8.81	8.81	46.21	15.88	11.55	tr	V8NKQ1	V8NKQ1_OPHHA	CWF19-like protein 1 (Fragment) OS=Ophiophagus hannah G	OPHHA	5
1195	8.79	8.8	42.88	5.742	4.773	tr	V8NWL8	V8NWL8_OPHHA	Chromodomain-helicase-DNA-binding protein 2 (Fragment)	OPHHA	4
1196	8.79	8.79	64.83	11.31	8.966	tr	V8NMY2	V8NMY2_OPHHA	Scaffold attachment factor B1 (Fragment) OS=Ophiophagus	OPHHA	4
1197	8.78	8.78	49.8	20.48	14.46	tr	V8NHM5	V8NHM5_OPHHA	Uncharacterized protein (Fragment) OS=Ophiophagus hanna	OPHHA	4
1198	8.78	8.78	41.26	15.38	15.38	tr	V8P788	V8P788_OPHHA	Peroxisomal 2,4-dienoyl-CoA reductase OS=Ophiophagus ha	OPHHA	7
1199	8.75	8.75	45.44	8.87	7.898	tr	V8NK59	V8NK59_OPHHA	Protein argonaute-1 (Fragment) OS=Ophiophagus hannah GN	OPHHA	5
1200	8.75	8.75	70.11	21.54	12.53	tr	V8NI92	V8NI92_OPHHA	SAM domain and HD domain-containing protein 1 (Fragment	OPHHA	4
1201	8.75	8.75	36.19	21.27	18.66	tr	V8NHH1	V8NHH1_OPHHA	Nucleophosmin (Fragment) OS=Ophiophagus hannah GN=NPM1	OPHHA	5
1202	8.74	8.74	54.79	13.79	8.429	tr	V8NEN0	V8NEN0_OPHHA	Transferrin receptor protein 1 (Fragment) OS=Ophiophagu	OPHHA	5
1203	8.72	8.73	78.43	48.04	42.16	tr	V8NPI8	V8NPI8_OPHHA	High mobility group protein B1 (Fragment) OS=Ophiophagu	OPHHA	5
1204	8.71	8.71	48.39	10.75	9.677	tr	V8NQI7	V8NQI7_OPHHA	Rab GTPase-binding effector protein 1 (Fragment) OS=Oph	OPHHA	6
1205	8.71	8.71	38.5	18.25	15.25	tr	V8P760	V8P760_OPHHA	Monoacylglycerol lipase ABHD12 (Fragment) OS=Ophiophagu	OPHHA	4
1206	8.71	8.71	63.2	39.6	35.2	tr	V8N7B1	V8N7B1_OPHHA	Guanine nucleotide-binding protein G(I)/G(S)/G(T) subun	OPHHA	5
1207	8.71	8.71	57.14	27.62	27.62	tr	V8N8M0	V8N8M0_OPHHA	60S ribosomal protein L23a (Fragment) OS=Ophiophagus ha	OPHHA	5
1208	8.7	8.86	56.1	15.02	7.981	tr	V8NZF6	V8NZF6_OPHHA	WD repeat-containing protein mio (Fragment) OS=Ophiopha	OPHHA	5
1209	8.7	8.7	68.23	19.86	17.33	tr	V8PF79	V8PF79_OPHHA	Sulfotransferase OS=Ophiophagus hannah GN=SULT1B1 PE=3	OPHHA	7
1210	8.7	8.7	80.77	33.17	28.37	tr	V8NP90	V8NP90_OPHHA	EF-hand domain-containing family member A1 OS=Ophiophag	OPHHA	5
1211	8.67	8.67	42.57	24.57	15.43	tr	V8P147	V8P147_OPHHA	Putative 4-hydroxy-2-oxoglutarate aldolase, mitochondri	OPHHA	6
1212	8.66	8.66	51.93	16.57	16.57	tr	V8P6U9	V8P6U9_OPHHA	Selenide, water dikinase 1 (Fragment) OS=Ophiophagus ha	OPHHA	5
1213	8.63	8.63	18.69	8.923	5.556	tr	V8PGS0	V8PGS0_OPHHA	Epsin-1 (Fragment) OS=Ophiophagus hannah GN=Epn1 PE=4 S	OPHHA	7
1214	8.62	8.62	38.49	11.29	6.022	tr	V8PFM7	V8PFM7_OPHHA	Superkiller viralicidic activity 2-like 2 (Fragment) OS	OPHHA	4
1215	8.61	8.61	22.48	19.88	19.88	tr	V8PB34	V8PB34_OPHHA	Translocating chain-associated membrane protein 1 (Frag	OPHHA	8
1216	8.59	8.6	58.42	12.63	11.4	tr	V8PIV4	V8PIV4_OPHHA	Glycylpeptide N-tetradecanoyltransferase (Fragment) OS=	OPHHA	5
1217	8.58	8.58	54.39	8.667	6.501	tr	V8NL95	V8NL95_OPHHA	Phosphatidylinositol 4-kinase alpha (Fragment) OS=Ophio	OPHHA	5
1218	8.58	8.58	46.07	16.57	11.8	tr	V8P6H2	V8P6H2_OPHHA	Cat eye syndrome critical region protein 5 (Fragment) O	OPHHA	4
1219	8.57	8.73	41.62	9.714	5.524	tr	V8PBU7	V8PBU7_OPHHA	Cystathionine beta-synthase (Fragment) OS=Ophiophagus h	OPHHA	5
1220	8.57	8.57	60.74	38.43	23.14	tr	V8NBZ0	V8NBZ0_OPHHA	Ubiquitin thioesterase OTUB1 (Fragment) OS=Ophiophagus	OPHHA	4
1221	8.57	8.57	52.94	23.53	23.04	tr	V8P869	V8P869_OPHHA	Serine/arginine-rich splicing factor 12 (Fragment) OS=O	OPHHA	5
1222	8.56	8.56	63.16	24.01	24.01	tr	V8N8W5	V8N8W5_OPHHA	ELAV-like protein 1 (Fragment) OS=Ophiophagus hannah GN	OPHHA	6
1223	8.56	8.56	53.48	14.34	9.426	tr	V8NEM7	V8NEM7_OPHHA	Target of Myb protein 1 OS=Ophiophagus hannah GN=TOM1 P	OPHHA	5
1224	8.55	8.58	45.03	6.277	3.656	tr	V8PA85	V8PA85_OPHHA	InaD-like protein (Fragment) OS=Ophiophagus hannah GN=I	OPHHA	4
1225	8.54	8.57	63.79	5.212	4.587	tr	V8NPP9	V8NPP9_OPHHA	FYVE and coiled-coil domain-containing protein 1 OS=Oph	OPHHA	6
1226	8.53	8.54	52.03	8.102	6.077	tr	V8NY13	V8NY13_OPHHA	E3 ubiquitin-protein ligase BRE1A OS=Ophiophagus hannah	OPHHA	4
1227	8.53	8.53	45.76	19.56	17.34	tr	V8NJF7	V8NJF7_OPHHA	Sulfotransferase (Fragment) OS=Ophiophagus hannah GN=Su	OPHHA	5
1228	8.52	8.52	55.9	28.13	19.79	tr	V8NZN1	V8NZN1_OPHHA	Aldose 1-epimerase (Fragment) OS=Ophiophagus hannah GN=	OPHHA	4
1229	8.5	8.5	55.88	21.76	15.59	tr	V8NDL2	V8NDL2_OPHHA	Vinexin (Fragment) OS=Ophiophagus hannah GN=SORBS3 PE=4	OPHHA	4
1230	8.5	8.5	50.66	17.11	17.11	tr	V8NIL4	V8NIL4_OPHHA	Putative deoxyribose-phosphate aldolase OS=Ophiophagus	OPHHA	5
1231	8.48	11.77	62.44	36.1	36.1	tr	V8NN41	V8NN41_OPHHA	Ras-related protein Rab-1A OS=Ophiophagus hannah GN=RAB	OPHHA	9
1232	8.48	9.64	38.76	6.435	4.921	tr	V8NEG2	V8NEG2_OPHHA	KN motif and ankyrin repeat domain-containing protein 1	OPHHA	5
1233	8.47	8.47	78.73	35.29	25.34	tr	V8P071	V8P071_OPHHA	Proteasome subunit beta type OS=Ophiophagus hannah GN=P	OPHHA	4
1234	8.46	8.57	29.53	6.345	5.076	tr	V8NXI6	V8NXI6_OPHHA	Tyrosine-protein phosphatase non-receptor type 23 (Frag	OPHHA	4
1235	8.46	8.46	51.15	4.287	4.287	tr	V8NVC6	V8NVC6_OPHHA	Collagen alpha-2(IV) chain (Fragment) OS=Ophiophagus ha	OPHHA	6
1236	8.45	8.45	71.12	22.09	12.38	tr	V8NAS8	V8NAS8_OPHHA	Leucine-rich repeat flightless-interacting protein 2 (F	OPHHA	5
1237	8.45	8.45	31.98	19.77	15.99	tr	V8P5D6	V8P5D6_OPHHA	Beta-1,3-glucosyltransferase (Fragment) OS=Ophiophagus	OPHHA	5
1238	8.44	8.44	52.31	18.46	16.15	tr	V8NKR4	V8NKR4_OPHHA	Pyruvate dehydrogenase protein X component, mitochondri	OPHHA	4
1239	8.43	8.43	63.39	45.36	37.16	tr	V8NR61	V8NR61_OPHHA	Lactoylglutathione lyase OS=Ophiophagus hannah GN=Glo1	OPHHA	5
1240	8.43	8.43	46.52	24.6	19.79	tr	V8P4F1	V8P4F1_OPHHA	Eukaryotic translation initiation factor 3 subunit J (F	OPHHA	4
1241	8.42	8.42	62.68	6.856	2.781	tr	V8NQT9	V8NQT9_OPHHA	A-kinase anchor protein 9 (Fragment) OS=Ophiophagus han	OPHHA	4

1242	8.42	8.42	55.94	23.27	18.81	tr|V8NYM1|V8NYM1_OPHHA Trans-2,3-enoyl-CoA reductase (Fragment) OS=Ophiophagus	OPHHA	5
1243	8.41	9.29	37.14	13.93	6.786	tr|V8P3P7|V8P3P7_OPHHA TBC1 domain family member 15 (Fragment) OS=Ophiophagus	OPHHA	3
1244	8.41	8.41	39.03	4.865	3.106	tr|V8P902|V8P902_OPHHA Lipopolysaccharide-responsive and beige-like anchor pro	OPHHA	4
1245	8.41	8.41	39.55	7.872	4.568	tr|V8NUS7|V8NUS7_OPHHA Nuclear pore complex protein (Fragment) OS=Ophiophagus	OPHHA	4
1246	8.4	8.4	51.16	18.82	8.879	tr|V8NRZ9|V8NRZ9_OPHHA Tryptophanyl-tRNA synthetase, cytoplasmic OS=Ophiophagu	OPHHA	3
1247	8.4	8.4	43.09	19.29	14.79	tr|V8NI81|V8NI81_OPHHA Ferritin OS=Ophiophagus hannah GN=L345_12574 PE=3 SV=1	OPHHA	4
1248	8.39	8.48	59.47	27.24	14.95	tr|V8NLN0|V8NLN0_OPHHA Uncharacterized protein (Fragment) OS=Ophiophagus hanna	OPHHA	5
1249	8.39	8.39	46.77	12.9	11.29	tr|V8PAV1|V8PAV1_OPHHA Putative carboxypeptidase PM20D1 (Fragment) OS=Ophiopha	OPHHA	4
1250	8.39	8.39	35.69	29.58	26.37	tr|V8NQM7|V8NQM7_OPHHA Acetyl-CoA acetyltransferase, cytosolic (Fragment) OS=O	OPHHA	6
1251	8.38	8.38	48.31	11.59	6.039	tr|V8PBC4|V8PBC4_OPHHA Rho guanine nucleotide exchange factor 7 (Fragment) OS=	OPHHA	4
1252	8.37	8.37	41.48	3.564	3.049	tr|V8PHT2|V8PHT2_OPHHA A-kinase anchor protein 13 (Fragment) OS=Ophiophagus ha	OPHHA	7
1253	8.37	8.37	55.56	13.07	12.75	tr|V8NNV1|V8NNV1_OPHHA Mitochondrial import inner membrane translocase subunit	OPHHA	4
1254	8.37	8.37	65.1	52.35	46.31	tr|V8N8J8|V8N8J8_OPHHA Dehydrogenase/reductase SDR family member 11 OS=Ophioph	OPHHA	4
1255	8.36	8.36	35.98	8.821	6.046	tr|V8POZ2|V8POZ2_OPHHA Acyl-coenzyme A thioesterase 1 (Fragment) OS=Ophiophagu	OPHHA	4
1256	8.35	13.25	49.23	6.12	3.989	tr|V8N7A2|V8N7A2_OPHHA Uncharacterized protein (Fragment) OS=Ophiophagus hanna	OPHHA	7
1257	8.35	8.68	28.76	6.352	5.15	tr|V8P8E6|V8P8E6_OPHHA GRB2-associated-binding protein 3 (Fragment) OS=Ophioph	OPHHA	5
1258	8.34	13.21	76.04	22.92	19.27	tr|V8NE74|V8NE74_OPHHA Uncharacterized protein (Fragment) OS=Ophiophagus hanna	OPHHA	6
1259	8.33	8.33	51.19	9.002	6.941	tr|V8NQ00|V8NQ00_OPHHA Transcription elongation factor SPT5 (Fragment) OS=Ophi	OPHHA	5
1260	8.33	8.33	58.86	21.43	14	tr|V8PGQ0|V8PGQ0_OPHHA Serine beta-lactamase-like protein LACTB, mitochondrial	OPHHA	4
1261	8.33	8.33	26.82	9.683	6.331	tr|V8N6C2|V8N6C2_OPHHA Acyl-coenzyme A amino acid N-acyltransferase 1 (Fragmen	OPHHA	4
1262	8.33	8.33	54.3	47.85	36.02	tr|V8PCM1|V8PCM1_OPHHA Erlin-2 OS=Ophiophagus hannah GN=ERLIN2 PE=4 SV=1	OPHHA	4
1263	8.32	8.46	33.01	20	13.73	tr|V8NNV0|V8NNV0_OPHHA Glutamate-rich WD repeat-containing protein 1 OS=Ophiop	OPHHA	4
1264	8.31	8.31	33.22	5.217	5.217	tr|V8NBS0|V8NBS0_OPHHA Macrophage mannose receptor 1 (Fragment) OS=Ophiophagus	OPHHA	6
1265	8.3	9.87	64.89	37.02	29.01	tr|V8P9E1|V8P9E1_OPHHA F-actin-capping protein subunit alpha-1 (Fragment) OS=O	OPHHA	6
1266	8.3	8.3	54.89	11.65	8.459	tr|V8PDS2|V8PDS2_OPHHA Translation initiation factor eIF-2B subunit gamma OS=O	OPHHA	4
1267	8.29	10.6	61.38	13.23	9.846	tr|V8PAY7|V8PAY7_OPHHA Heat shock protein 75 kDa, mitochondrial OS=Ophiophagus	OPHHA	5
1268	8.28	8.29	17.84	10.8	8.291	tr|V8NH26|V8NH26_OPHHA Cleft lip and palate transmembrane protein 1-like prote	OPHHA	4
1269	8.27	8.27	52.64	13.53	6.642	tr|V8NPR0|V8NPR0_OPHHA Plakophilin-3 (Fragment) OS=Ophiophagus hannah GN=Pkp3	OPHHA	4
1270	8.27	8.27	60.63	28.51	16.74	tr|V8NA36|V8NA36_OPHHA Uncharacterized protein (Fragment) OS=Ophiophagus hanna	OPHHA	4
1271	8.27	8.27	50	21.22	18.31	tr|V8P857|V8P857_OPHHA Methylosome protein 50 OS=Ophiophagus hannah GN=Wdr77 P	OPHHA	4
1272	8.26	8.26	38.26	9.266	6.606	tr|V8P0Y8|V8P0Y8_OPHHA Pre-mRNA-splicing factor ATP-dependent RNA helicase PRP	OPHHA	5
1273	8.25	8.26	55.19	27.04	27.04	tr|V8NZS4|V8NZS4_OPHHA S-methyl-5'-thioadenosine phosphorylase (Fragment) OS=O	OPHHA	5
1274	8.24	8.24	41.84	14.54	11.87	tr|V8PBE6|V8PBE6_OPHHA Sulfotransferase OS=Ophiophagus hannah GN=Sult1a1 PE=3	OPHHA	5
1275	8.23	27.16	52.72	49.75	45.05	tr|V8P5I4|V8P5I4_OPHHA Uncharacterized protein OS=Ophiophagus hannah GN=L345_0	OPHHA	16
1276	8.23	8.23	53.98	24.22	19.38	tr|V8NT89|V8NT89_OPHHA Isochorismatase domain-containing protein 1 (Fragment)	OPHHA	4
1277	8.23	8.23	51.5	23.18	14.59	tr|V8P7N2|V8P7N2_OPHHA UMP-CMP kinase OS=Ophiophagus hannah GN=CMPK1 PE=3 SV=1	OPHHA	4
1278	8.22	8.22	34.97	24.04	20.49	tr|V8P3U9|V8P3U9_OPHHA Creatine kinase B-type (Fragment) OS=Ophiophagus hannah	OPHHA	4
1279	8.21	8.94	34.14	7.484	4.771	tr|V8P9K2|V8P9K2_OPHHA Cation-transporting ATPase (Fragment) OS=Ophiophagus ha	OPHHA	4
1280	8.21	8.21	51.08	12.77	11.04	tr|V8NKT0|V8NKT0_OPHHA RNA-binding protein 47 (Fragment) OS=Ophiophagus hannah	OPHHA	5
1281	8.2	8.2	42.95	16.99	14.1	tr|V8P5I3|V8P5I3_OPHHA D-aspartate oxidase (Fragment) OS=Ophiophagus hannah GN	OPHHA	5
1282	8.19	8.19	51.03	46.9	46.9	tr|V8NHR8|V8NHR8_OPHHA Ubiquitin-conjugating enzyme E2 K (Fragment) OS=Ophioph	OPHHA	5
1283	8.18	8.33	28.83	19.68	11.21	tr|V8NKN0|V8NKN0_OPHHA Histone-binding protein RBBP4 (Fragment) OS=Ophiophagus	OPHHA	3
1284	8.17	8.18	62.95	29.79	21.5	tr|V8NHF9|V8NHF9_OPHHA Methionine aminopeptidase 1 OS=Ophiophagus hannah GN=ME	OPHHA	6
1285	8.17	8.17	47.3	12.45	10.17	tr|V8NH29|V8NH29_OPHHA Proactivator polypeptide OS=Ophiophagus hannah GN=PSAP	OPHHA	5
1286	8.16	8.16	27.43	27.43	27.43	tr|V8NAC9|V8NAC9_OPHHA NADH dehydrogenase [ubiquinone] 1 subunit C2 OS=Ophioph	OPHHA	4
1287	8.15	8.15	62.98	27.88	25	tr|V8PEW2|V8PEW2_OPHHA NADH dehydrogenase [ubiquinone] iron-sulfur protein 8,	OPHHA	4
1288	8.13	8.22	56.8	17.24	11.76	tr|V8P8U4|V8P8U4_OPHHA DnaJ-like subfamily C member 3 (Fragment) OS=Ophiophagu	OPHHA	6
1289	8.13	8.13	47.77	4.738	2.428	tr|V8NU88|V8NU88_OPHHA Chromodomain-helicase-DNA-binding protein 4 OS=Ophiopha	OPHHA	5
1290	8.13	8.13	40.47	13.14	8.898	tr|V8P2C1|V8P2C1_OPHHA RNA-binding protein FUS OS=Ophiophagus hannah GN=FUS PE	OPHHA	5
1291	8.13	8.13	44.53	21.89	21.89	tr|V8NHN6|V8NHN6_OPHHA LanC-like protein 2 OS=Ophiophagus hannah GN=LANCL2 PE=	OPHHA	4
1292	8.12	8.12	44.99	17.72	15.85	tr|V8N6Z1|V8N6Z1_OPHHA Mannose-6-phosphate isomerase (Fragment) OS=Ophiophagus	OPHHA	4
1293	8.11	8.11	49.26	9.664	8.188	tr|V8NZE8|V8NZE8_OPHHA Conserved oligomeric Golgi complex subunit 4 (Fragment)	OPHHA	4
1294	8.11	8.11	41.31	11.96	11.96	tr|V8PDD3|V8PDD3_OPHHA Ran-binding protein 3 (Fragment) OS=Ophiophagus hannah	OPHHA	4
1295	8.1	8.1	42.38	18.29	15.85	tr|V8P0J5|V8P0J5_OPHHA Eukaryotic translation initiation factor 3 subunit F OS	OPHHA	4
1296	8.09	8.09	28.37	7.151	7.151	tr|V8NLL4|V8NLL4_OPHHA Furin OS=Ophiophagus hannah GN=FURIN PE=3 SV=1	OPHHA	4
1297	8.09	8.09	55.71	21.92	21.92	tr|V8P0X8|V8P0X8_OPHHA Translin (Fragment) OS=Ophiophagus hannah GN=TSN PE=4 S	OPHHA	5
1298	8.09	8.09	65.12	32.56	32.56	tr|V8NLG0|V8NLG0_OPHHA 10 kDa heat shock protein, mitochondrial (Fragment) OS=	OPHHA	7
1299	8.08	8.15	54.5	8.882	5.702	tr|V8NXS7|V8NXS7_OPHHA DNA ligase (Fragment) OS=Ophiophagus hannah GN=LIG3 PE=	OPHHA	4
1300	8.07	8.09	34.61	12.93	10.93	tr|V8NEF3|V8NEF3_OPHHA Peptidyl-prolyl cis-trans isomerase FKBP10 OS=Ophiophag	OPHHA	5
1301	8.06	8.06	43.42	10.44	10.44	tr|V8NG15|V8NG15_OPHHA Zinc transporter 9 (Fragment) OS=Ophiophagus hannah GN=	OPHHA	4
1302	8.05	8.05	51.74	10.99	10.99	tr|V8P400|V8P400_OPHHA X-ray repair cross-complementing protein 6 (Fragment) O	OPHHA	4
1303	8.04	8.04	41.28	7.11	7.11	tr|V8NNW5|V8NNW5_OPHHA Complement C5 (Fragment) OS=Ophiophagus hannah GN=C5 PE	OPHHA	4
1304	8.04	8.04	60.22	14.36	14.36	tr|V8N665|V8N665_OPHHA Protein-glutamine gamma-glutamyltransferase E (Fragment	OPHHA	4
1305	8.04	8.04	72.22	32.64	32.64	tr|V8P783|V8P783_OPHHA Nucleoside diphosphate kinase, mitochondrial (Fragment)	OPHHA	5
1306	8.04	8.04	39.87	23.59	21.26	tr|V8PED8|V8PED8_OPHHA Mitochondrial carrier-like 2 OS=Ophiophagus hannah GN=M	OPHHA	5
1307	8.02	14.94	29.27	11.28	10.28	tr|V8P3H7|V8P3H7_OPHHA Sarcoplasmic/endoplasmic reticulum calcium ATPase 1 OS=	OPHHA	7
1308	8.02	10.31	59.46	34.05	34.05	tr|V8PFI9|V8PFI9_OPHHA Rho-related GTP-binding protein RhoB (Fragment) OS=Ophi	OPHHA	7
1309	8.02	8.54	38.38	14.39	14.39	tr|V8P6E9|V8P6E9_OPHHA Kynureninase (Fragment) OS=Ophiophagus hannah GN=KYNU P	OPHHA	5
1310	8.01	8.02	57.19	11.87	7.554	tr|V8P8Y0|V8P8Y0_OPHHA Procollagen galactosyltransferase 1 (Fragment) OS=Ophio	OPHHA	4
1311	8	13.35	85.71	45.58	45.58	tr|V8N2E2|V8N2E2_OPHHA Uncharacterized protein (Fragment) OS=Ophiophagus hanna	OPHHA	8
1312	8	8	37.1	4.708	4.708	tr|V8P6C4|V8P6C4_OPHHA Lymphocyte antigen 75 (Fragment) OS=Ophiophagus hannah	OPHHA	5
1313	8	8	43.49	12.82	12.82	tr|V8PGK0|V8PGK0_OPHHA D site-binding protein (Fragment) OS=Ophiophagus hannah	OPHHA	5
1314	8	8	62.03	31.01	31.01	tr|V8NY64|V8NY64_OPHHA Synaptosomal-associated protein 23 OS=Ophiophagus hanna	OPHHA	4
1315	8	8	64.29	44.05	44.05	tr|V8PBL9|V8PBL9_OPHHA Sorting nexin-12 (Fragment) OS=Ophiophagus hannah GN=SN	OPHHA	4
1316	8	8	75.19	20.93	20.93	tr|V8NWM5|V8NWM5_OPHHA 17-beta-hydroxysteroid dehydrogenase 13 (Fragment) OS=O	OPHHA	4
1317	7.99	8	73.19	21.18	19.03	tr|V8NUW7|V8NUW7_OPHHA Isocitrate dehydrogenase [NAD] subunit alpha, mitochond	NAD	5
1318	7.99	7.99	63.51	7.74	7.74	tr|V8NK84|V8NK84_OPHHA Liprin-beta-1 OS=Ophiophagus hannah GN=PPFIBP1 PE=4 SV=	OPHHA	5
1319	7.99	7.99	73.33	40	34.81	tr|V8NQT7|V8NQT7_OPHHA Short-chain specific acyl-CoA dehydrogenase, mitochondr	OPHHA	5
1320	7.98	7.98	57.7	13.17	13.17	tr|V8NSQ0|V8NSQ0_OPHHA Torsin-1A-interacting protein 1 OS=Ophiophagus hannah G	OPHHA	4
1321	7.97	7.97	37.03	2.61	2.61	tr|V8P8R8|V8P8R8_OPHHA Autophagy-related protein 9A (Fragment) OS=Ophiophagus	OPHHA	4
1322	7.97	7.97	36.28	7.419	5.386	tr|V8NEY4|V8NEY4_OPHHA SCY1-like protein 2 (Fragment) OS=Ophiophagus hannah GN	OPHHA	4
1323	7.97	7.97	69.23	39.23	30	tr|V8NAA6|V8NAA6_OPHHA 40S ribosomal protein S15a OS=Ophiophagus hannah GN=RPS	OPHHA	4
1324	7.96	7.96	53.45	40.52	39.66	tr|V8P4X6|V8P4X6_OPHHA Eukaryotic translation initiation factor 1A, Y-chromoso	OPHHA	5
1325	7.96	7.96	39.61	12.96	12.96	tr|V8NDI3|V8NDI3_OPHHA Carboxylic ester hydrolase OS=Ophiophagus hannah GN=L34	OPHHA	5
1326	7.96	7.96	39.59	20.41	16.33	tr|V8PBD4|V8PBD4_OPHHA Inositol monophosphatase 1 (Fragment) OS=Ophiophagus ha	OPHHA	4
1327	7.94	7.94	39.18	16.42	14.74	tr|V8NX44|V8NX44_OPHHA [Pyruvate dehydrogenase [acetyl-transferring]]-phosphat	Pyruvate	5
1328	7.94	7.94	34.53	21.62	17.42	tr|V8PDI5|V8PDI5_OPHHA Uncharacterized protein OS=Ophiophagus hannah GN=L345_0	OPHHA	5
1329	7.93	8.45	71.81	32.21	22.15	tr|V8N6H9|V8N6H9_OPHHA Glycogen [starch] synthase, liver (Fragment) OS=Ophioph	OPHHA	5
1330	7.93	7.94	63.31	44.97	34.91	tr|V8NHP6|V8NHP6_OPHHA Regulation of nuclear pre-mRNA domain-containing protei	OPHHA	5
1331	7.93	7.93	85.43	32.39	28.34	tr|V8P0T1|V8P0T1_OPHHA PRKC apoptosis WT1 regulator protein (Fragment) OS=Ophi	OPHHA	4
1332	7.92	7.92	33.12	16.03	9.916	tr|V8NEJ7|V8NEJ7_OPHHA Uncharacterized protein (Fragment) OS=Ophiophagus hanna	OPHHA	4
1333	7.92	7.92	39.36	17.02	17.02	tr|V8P531|V8P531_OPHHA Cytochrome b-c1 complex subunit 7 (Fragment) OS=Ophioph	OPHHA	5
1334	7.91	7.91	35.01	6.162	5.509	tr|V8P2D7|V8P2D7_OPHHA Zinc finger protein (Fragment) OS=Ophiophagus hannah GN	OPHHA	5
1335	7.91	7.91	56.15	14.12	9.302	tr|V8PDL0|V8PDL0_OPHHA WD repeat-containing protein 48 (Fragment) OS=Ophiophag	OPHHA	4
1336	7.91	7.91	18.03	3.72	3.72	tr|V8P7P1|V8P7P1_OPHHA Carboxylic ester hydrolase (Fragment) OS=Ophiophagus ha	OPHHA	6
1337	7.91	7.91	45	19.67	16.33	tr|V8N8G8|V8N8G8_OPHHA Very long-chain specific acyl-CoA dehydrogenase, mitoch	OPHHA	7

1338	7.9	17.81	59.17	40.83	33.14	tr	V8P7D3	V8P7D3_OPHHA	Eukaryotic translation initiation factor 5A OS=Ophiopha	OPHHA	12
1339	7.9	7.9	36.22	22.76	18.59	tr	V8P7N4	V8P7N4_OPHHA	Lipid phosphate phosphohydrolase 3 (Fragment) OS=Ophiop	OPHHA	4
1340	7.89	7.89	50.13	19.34	14.5	tr	V8PCH4	V8PCH4_OPHHA	Golgi-associated PDZ and coiled-coil motif-containing p	OPHHA	6
1341	7.89	7.89	64.65	21.34	18.47	tr	V8P6Q2	V8P6Q2_OPHHA	DNA-(apurinic or apyrimidinic site) lyase OS=Ophiophagu	OPHHA	5
1342	7.88	7.91	45.07	2.9	1.801	tr	V8NWK3	V8NWK3_OPHHA	Nesprin-1 (Fragment) OS=Ophiophagus hannah GN=SYNE1 PE=	OPHHA	8
1343	7.88	7.88	53.29	12.65	9.444	tr	V8P6G2	V8P6G2_OPHHA	Procollagen-lysine,2-oxoglutarate 5-dioxygenase 3 OS=Op	OPHHA	4
1344	7.87	10.23	51.22	11.89	8.566	tr	V8PFS0	V8PFS0_OPHHA	Tyrosine-protein kinase (Fragment) OS=Ophiophagus hanna	OPHHA	5
1345	7.87	7.87	88.34	38.65	32.52	tr	V8PGN0	V8PGN0_OPHHA	Superoxide dismutase [Cu-Zn] OS=Ophiophagus hannah GN=L	Cu-Zn	6
1346	7.86	7.88	28.47	12.73	10.42	tr	V8NQJ4	V8NQJ4_OPHHA	Cathepsin Z (Fragment) OS=Ophiophagus hannah GN=CTSZ PE	OPHHA	4
1347	7.86	7.87	45.13	3.859	1.963	tr	V8PGL6	V8PGL6_OPHHA	Protein SON (Fragment) OS=Ophiophagus hannah GN=SON PE=	OPHHA	5
1348	7.85	7.85	32.83	11.72	8.276	tr	V8NT05	V8NT05_OPHHA	Nuclear cap-binding protein subunit 1 (Fragment) OS=Oph	OPHHA	5
1349	7.85	7.85	40.41	21.51	17.15	tr	V8P2W1	V8P2W1_OPHHA	IST1-like protein OS=Ophiophagus hannah GN=IST1 PE=4 SV	OPHHA	5
1350	7.85	7.85	54.48	26.12	22.76	tr	V8NS03	V8NS03_OPHHA	TIP41-like protein (Fragment) OS=Ophiophagus hannah GN=	OPHHA	4
1351	7.84	7.84	46.1	7.797	3.22	tr	V8P4C3	V8P4C3_OPHHA	Methionine synthase (Fragment) OS=Ophiophagus hannah GN	OPHHA	3
1352	7.83	7.83	55.88	15.59	12.95	tr	V8NRD0	V8NRD0_OPHHA	Myoferlin (Fragment) OS=Ophiophagus hannah GN=Myof PE=4	OPHHA	4
1353	7.81	7.81	72.87	32.45	27.13	tr	V8PGJ5	V8PGJ5_OPHHA	60S ribosomal protein L18 OS=Ophiophagus hannah GN=rpl1	OPHHA	4
1354	7.81	7.81	47.6	22.75	22.75	tr	V8P771	V8P771_OPHHA	Protein FAM98B (Fragment) OS=Ophiophagus hannah GN=Fam9	OPHHA	5
1355	7.8	8.49	56.86	8.007	7.026	tr	V8PC11	V8PC11_OPHHA	Peroxidase mlt-7 (Fragment) OS=Ophiophagus hannah GN=ml	OPHHA	5
1356	7.8	7.8	43.87	35.48	35.48	tr	V8PHT5	V8PHT5_OPHHA	Proteasome subunit alpha type-5 (Fragment) OS=Ophiophag	OPHHA	5
1357	7.77	7.81	62.22	49.63	42.96	tr	V8NLX1	V8NLX1_OPHHA	ADP-ribosylation factor-like protein 1 OS=Ophiophagus h	OPHHA	4
1358	7.77	7.79	44.05	5.712	4.93	tr	V8NZY9	V8NZY9_OPHHA	Cell division cycle and apoptosis regulator protein 1 (	OPHHA	4
1359	7.77	7.77	41.2	24.65	15.85	tr	V8PBU8	V8PBU8_OPHHA	Nicotinate-nucleotide pyrophosphorylase [carboxylating]	OPHHA	6
1360	7.76	7.76	58.61	13.86	13.86	tr	V8PF88	V8PF88_OPHHA	Nucleoporin Nup37 (Fragment) OS=Ophiophagus hannah GN=N	OPHHA	5
1361	7.76	7.76	65.59	38.17	29.57	tr	V8P763	V8P763_OPHHA	Adenylate kinase isoenzyme 4, mitochondrial OS=Ophiopha	OPHHA	5
1362	7.75	7.75	56.2	12.14	7.635	tr	V8NYE4	V8NYE4_OPHHA	Dynamin-1-like protein (Fragment) OS=Ophiophagus hannah	OPHHA	5
1363	7.75	7.75	73.62	48.47	28.22	tr	V8NPD8	V8NPD8_OPHHA	Trafficking protein particle complex subunit 6B OS=Ophi	OPHHA	4
1364	7.74	7.74	23.35	14.97	12.38	tr	V8PG41	V8PG41_OPHHA	Poliovirus receptor-related protein 3 (Fragment) OS=Oph	OPHHA	4
1365	7.74	7.74	72.44	40.94	38.58	tr	V8N702	V8N702_OPHHA	Uncharacterized protein (Fragment) OS=Ophiophagus hanna	OPHHA	4
1366	7.73	8.8	62.15	18.13	12.95	tr	V8NCY7	V8NCY7_OPHHA	Fermitin family-like 3 (Fragment) OS=Ophiophagus hannah	OPHHA	5
1367	7.73	7.73	54.29	21.63	21.63	tr	V8P1L5	V8P1L5_OPHHA	Syntaxin-7 OS=Ophiophagus hannah GN=STX7 PE=4 SV=1	OPHHA	4
1368	7.73	7.73	43.46	15.69	15.69	tr	V8PCV0	V8PCV0_OPHHA	Septin-1 (Fragment) OS=Ophiophagus hannah GN=SEPT1 PE=3	OPHHA	4
1369	7.72	7.72	46.7	6.089	4.37	tr	V8PJH0	V8PJH0_OPHHA	Trafficking protein particle complex subunit 8 (Fragmen	OPHHA	5
1370	7.71	7.71	45.44	11.36	11.36	tr	V8NQK0	V8NQK0_OPHHA	Tryptophan 2,3-dioxygenase (Fragment) OS=Ophiophagus ha	OPHHA	5
1371	7.71	7.71	55.29	18.25	15.61	tr	V8N9K7	V8N9K7_OPHHA	Collagen type IV alpha-3-binding protein (Fragment) OS=	OPHHA	6
1372	7.69	10.26	54.52	8.601	8.601	tr	V8NSS1	V8NSS1_OPHHA	DNA (Cytosine-5)-methyltransferase 3B (Fragment) OS=Oph	OPHHA	5
1373	7.68	7.68	57.54	8.432	8.432	tr	V8PCK9	V8PCK9_OPHHA	DNA topoisomerase 1 (Fragment) OS=Ophiophagus hannah GN	OPHHA	5
1374	7.68	7.68	44.68	6.586	5.928	tr	V8PAN8	V8PAN8_OPHHA	Coronin (Fragment) OS=Ophiophagus hannah GN=Coro7 PE=3	OPHHA	4
1375	7.67	7.67	65.21	13.82	13.82	tr	V8NWA5	V8NWA5_OPHHA	Protein kinase C and casein kinase substrate in neurons	OPHHA	5
1376	7.66	7.68	44.98	18.88	15.66	tr	V8NGG6	V8NGG6_OPHHA	40S ribosomal protein S6 OS=Ophiophagus hannah GN=RPS6	OPHHA	6
1377	7.66	7.66	66.49	24.05	24.05	tr	V8NFU8	V8NFU8_OPHHA	NAD-dependent deacetylase sirtuin-3, mitochondrial (Fra	OPHHA	6
1378	7.65	7.66	26.4	11.31	10.2	tr	V8PD09	V8PD09_OPHHA	Integrin alpha-5 (Fragment) OS=Ophiophagus hannah GN=IT	OPHHA	4
1379	7.64	7.72	43.94	11.95	8.586	tr	V8NW46	V8NW46_OPHHA	Serine/threonine-protein phosphatase 2A 56 kDa regulato	OPHHA	4
1380	7.64	7.64	81.38	24.48	23.45	tr	V8N4W5	V8N4W5_OPHHA	Succinyl-CoA ligase [ADP/GDP-forming] subunit alpha, mi	ADP/GDP-f	5
1381	7.63	7.63	82.39	24.53	18.24	tr	V8N9S3	V8N9S3_OPHHA	Cytochrome protein OS=Ophiophagus hannah GN=Cyp2c23 PE=	OPHHA	5
1382	7.62	7.62	42.9	17.76	14.21	tr	V8NQ63	V8NQ63_OPHHA	Sphingosine-1-phosphate lyase 1 OS=Ophiophagus hannah G	OPHHA	4
1383	7.6	7.6	62.54	17.14	17.14	tr	V8NWG8	V8NWG8_OPHHA	Calcium uptake protein 1, mitochondrial (Fragment) OS=O	OPHHA	4
1384	7.59	7.59	42.72	10.73	9.387	tr	V8P5X7	V8P5X7_OPHHA	Ran GTPase-activating protein 1 OS=Ophiophagus hannah G	OPHHA	4
1385	7.58	7.58	50.92	8.067	4.79	tr	V8NIV9	V8NIV9_OPHHA	Transcription elongation factor SPT6 (Fragment) OS=Ophi	OPHHA	4
1386	7.58	7.58	55.36	19.72	19.72	tr	V8NBS8	V8NBS8_OPHHA	E3 ubiquitin-protein ligase UBR4 (Fragment) OS=Ophiopha	OPHHA	4
1387	7.57	7.57	56.59	6.919	3.602	tr	V8PCT0	V8PCT0_OPHHA	Ankycorbin (Fragment) OS=Ophiophagus hannah GN=RAI14 PE	OPHHA	3
1388	7.57	7.57	59.9	32.29	25	tr	V8PJC8	V8PJC8_OPHHA	SAP domain-containing ribonucleoprotein OS=Ophiophagus	OPHHA	4
1389	7.57	7.57	31.56	21.25	18.44	tr	V8NM79	V8NM79_OPHHA	Serine/threonine-protein kinase PAK 2 (Fragment) OS=Oph	OPHHA	4
1390	7.56	7.56	34.44	4.06	3.077	tr	V8P3K1	V8P3K1_OPHHA	Lysine-specific demethylase 5B-B (Fragment) OS=Ophiopha	OPHHA	6
1391	7.55	7.55	60.81	11.49	8.615	tr	V8P1Q5	V8P1Q5_OPHHA	Collagen alpha-3(VI) chain (Fragment) OS=Ophiophagus ha	OPHHA	5
1392	7.55	7.55	49.59	15.45	15.45	tr	V8P1P0	V8P1P0_OPHHA	MAGUK p55 subfamily member 7 (Fragment) OS=Ophiophagus	OPHHA	4
1393	7.55	7.55	44.75	31.05	22.83	tr	V8P9G6	V8P9G6_OPHHA	Grancalcin OS=Ophiophagus hannah GN=Gca PE=4 SV=1	OPHHA	4
1394	7.55	7.55	52.17	28.99	28.99	tr	V8NIT9	V8NIT9_OPHHA	Putative oxidoreductase GLYR1 OS=Ophiophagus hannah GN=	OPHHA	5
1395	7.54	7.54	45.75	12.71	6.519	tr	V8P573	V8P573_OPHHA	Bifunctional protein NCOAT OS=Ophiophagus hannah GN=MGE	OPHHA	4
1396	7.54	7.54	74.89	30.14	24.66	tr	V8NRL5	V8NRL5_OPHHA	Protein Niban OS=Ophiophagus hannah GN=FAM129A PE=4 SV=	OPHHA	6
1397	7.53	7.53	54.01	10.76	10.76	tr	V8NPP3	V8NPP3_OPHHA	ATP-binding cassette sub-family D member 3 OS=Ophiophag	OPHHA	4
1398	7.52	7.52	47.33	12.17	9	tr	V8PB55	V8PB55_OPHHA	LEM domain-containing protein 2 (Fragment) OS=Ophiophag	OPHHA	4
1399	7.52	7.52	56.17	17.28	14.2	tr	V8NFZ5	V8NFZ5_OPHHA	UDP-N-acetylhexosamine pyrophosphorylase-like protein 1	OPHHA	5
1400	7.52	7.52	87.94	40.43	33.33	tr	V8P5L4	V8P5L4_OPHHA	Tumor susceptibility protein (Fragment) OS=Ophiophagus	OPHHA	4
1401	7.5	13.89	45.1	17.65	15.2	tr	V8N5L7	V8N5L7_OPHHA	Retinol dehydrogenase 16 (Fragment) OS=Ophiophagus hann	OPHHA	11
1402	7.5	7.5	38.94	6.673	4.753	tr	V8NR19	V8NR19_OPHHA	Synergin gamma OS=Ophiophagus hannah GN=SYNRG PE=4 SV=1	OPHHA	4
1403	7.49	7.49	60.62	17.5	17.5	tr	V8PCU2	V8PCU2_OPHHA	39S ribosomal protein L39, mitochondrial OS=Ophiophagus	OPHHA	4
1404	7.48	7.48	39.3	26.87	19.9	tr	V8PDK5	V8PDK5_OPHHA	Ras-related protein Rab-9A OS=Ophiophagus hannah GN=RAB	OPHHA	5
1405	7.47	7.47	35.78	8.687	5.791	tr	V8PA33	V8PA33_OPHHA	Trafficking protein particle complex subunit 11 OS=Ophi	OPHHA	4
1406	7.46	7.86	45.57	10.55	4.975	tr	V8PF57	V8PF57_OPHHA	Phosphorylase b kinase regulatory subunit beta OS=Ophio	OPHHA	4
1407	7.43	7.43	31.99	6.239	6.239	tr	V8PJ25	V8PJ25_OPHHA	PAB-dependent poly(A)-specific ribonuclease subunit 2 (	OPHHA	4
1408	7.43	7.43	62.65	12.65	12.65	tr	V8NM18	V8NM18_OPHHA	Parafibromin (Fragment) OS=Ophiophagus hannah GN=CDC73	OPHHA	4
1409	7.43	7.43	52.33	45.35	37.21	tr	V8N6Z7	V8N6Z7_OPHHA	Proteasome subunit alpha type-5 (Fragment) OS=Ophiophag	OPHHA	5
1410	7.42	8.89	70.63	43.65	34.92	tr	V8NND9	V8NND9_OPHHA	Pterin-4-alpha-carbinolamine dehydratase (Fragment) OS=	OPHHA	6
1411	7.42	7.44	47.3	14.7	7.939	tr	V8PBW7	V8PBW7_OPHHA	Histone-lysine N-methyltransferase setd3 OS=Ophiophagus	OPHHA	4
1412	7.42	7.42	65.22	6.545	5.175	tr	V8NLX6	V8NLX6_OPHHA	Collagen alpha-1(I) chain OS=Ophiophagus hannah GN=COL1	OPHHA	5
1413	7.41	8.76	69.62	32.28	32.28	tr	V8PCQ9	V8PCQ9_OPHHA	Single-stranded DNA-binding protein, mitochondrial OS=O	OPHHA	7
1414	7.4	7.4	56.58	15.79	15.79	tr	V8PIM7	V8PIM7_OPHHA	Cytoplasmic protein NCK2 OS=Ophiophagus hannah GN=NCK2	OPHHA	5
1415	7.4	7.4	38.48	14.01	12.11	tr	V8NBY5	V8NBY5_OPHHA	N-acetylglucosamine-6-sulfatase (Fragment) OS=Ophiophag	OPHHA	4
1416	7.39	7.39	29.52	24.1	17.47	tr	V8PJE4	V8PJE4_OPHHA	Peptidyl-prolyl cis-trans isomerase (Fragment) OS=Ophio	OPHHA	5
1417	7.38	7.44	61.93	19.54	10.91	tr	V8NCH3	V8NCH3_OPHHA	Tyrosine-protein kinase SYK (Fragment) OS=Ophiophagus h	OPHHA	3
1418	7.38	7.38	77.27	38.18	30	tr	V8P956	V8P956_OPHHA	Protein BRICK1 (Fragment) OS=Ophiophagus hannah GN=BRK1	OPHHA	5
1419	7.37	7.37	49.8	4.573	3.317	tr	V8P1C1	V8P1C1_OPHHA	Cytoskeleton-associated protein 5 (Fragment) OS=Ophioph	OPHHA	6
1420	7.37	7.37	51.38	21.54	21.54	tr	V8PH38	V8PH38_OPHHA	Yorkie-like protein OS=Ophiophagus hannah GN=YAP1 PE=4	OPHHA	4
1421	7.37	7.37	49.18	39.34	39.34	tr	V8NJG3	V8NJG3_OPHHA	Mitochondrial import inner membrane translocase subunit	OPHHA	4
1422	7.36	7.36	56.73	15.25	12.11	tr	V8NFG1	V8NFG1_OPHHA	Alpha-1,3-mannosyl-glycoprotein 2-beta-N-acetylglucosam	OPHHA	4
1423	7.35	7.35	32.62	3.19	2.286	tr	V8PHJ2	V8PHJ2_OPHHA	Desmoglein-2 OS=Ophiophagus hannah GN=DSG2 PE=4 SV=1	OPHHA	4
1424	7.34	7.34	42.57	6.275	4.289	tr	V8P8B7	V8P8B7_OPHHA	Synaptopodin-2 (Fragment) OS=Ophiophagus hannah GN=SYNP	OPHHA	4
1425	7.34	7.34	42.48	15.58	9.381	tr	V8NU70	V8NU70_OPHHA	ATP-dependent RNA helicase A (Fragment) OS=Ophiophagus	OPHHA	4
1426	7.34	7.34	62.5	36.11	36.11	tr	V8P5J1	V8P5J1_OPHHA	Coiled-coil domain-containing protein 58 OS=Ophiophagus	OPHHA	4
1427	7.33	7.33	55.33	19.8	19.8	tr	V8NN75	V8NN75_OPHHA	GTP-binding protein Rheb (Fragment) OS=Ophiophagus hann	OPHHA	5
1428	7.32	7.32	47.32	27.76	23.66	tr	V8P5N7	V8P5N7_OPHHA	mRNA export factor (Fragment) OS=Ophiophagus hannah GN=	OPHHA	4
1429	7.31	7.31	48.04	8.096	6.05	tr	V8P9L1	V8P9L1_OPHHA	Tripartite motif-containing protein 2 (Fragment) OS=Oph	OPHHA	4
1430	7.29	7.39	28.65	8.26	7.206	tr	V8PGW3	V8PGW3_OPHHA	Lumican (Fragment) OS=Ophiophagus hannah GN=LUM PE=4 SV	OPHHA	5
1431	7.29	7.29	40.81	16.18	16.18	tr	V8PFV7	V8PFV7_OPHHA	Down syndrome critical region protein 3 OS=Ophiophagus	OPHHA	4
1432	7.29	7.29	73.49	28.92	28.92	tr	V8N3T9	V8N3T9_OPHHA	Sulfotransferase (Fragment) OS=Ophiophagus hannah GN=L3	OPHHA	6
1433	7.28	7.28	44.9	9.475	6.122	tr	V8NEH4	V8NEH4_OPHHA	Phospholipase A2 (Fragment) OS=Ophiophagus hannah GN=PL	OPHHA	3

1434	7.28	7.28	26.78	6.345	6.345	tr	V8PHD7	V8PHD7_OPHHA	Transmembrane protease serine 6 (Fragment) OS=Ophiophag	OPHHA	4
1435	7.28	7.28	38.56	11.86	9.958	tr	V8P995	V8P995_OPHHA	Selenoprotein O (Fragment) OS=Ophiophagus hannah GN=SEL	OPHHA	4
1436	7.26	7.65	57.44	11.79	8.718	tr	V8NLY3	V8NLY3_OPHHA	Erlin-1 OS=Ophiophagus hannah GN=Erlin1 PE=4 SV=1	OPHHA	4
1437	7.26	7.28	26.75	8.618	6.686	tr	V8NCK6	V8NCK6_OPHHA	Inter-alpha-trypsin inhibitor heavy chain H2 (Fragment)	OPHHA	4
1438	7.25	7.25	51.66	21.33	18.48	tr	V8P3K9	V8P3K9_OPHHA	60S ribosomal protein L13 OS=Ophiophagus hannah GN=RPL1	OPHHA	4
1439	7.25	7.25	33.33	18.33	11.67	tr	V8PBS5	V8PBS5_OPHHA	Proteasome subunit beta type (Fragment) OS=Ophiophagus	OPHHA	4
1440	7.25	7.25	48.48	25.25	25.25	tr	V8PHC5	V8PHC5_OPHHA	COMM domain-containing protein 9 (Fragment) OS=Ophiopha	OPHHA	4
1441	7.24	7.24	34.54	4.067	3.422	tr	V8P3Z1	V8P3Z1_OPHHA	Nuclear pore membrane glycoprotein (Fragment) OS=Ophiop	OPHHA	4
1442	7.24	7.24	47.65	30	24.12	tr	V8NPE9	V8NPE9_OPHHA	Peptidyl-prolyl cis-trans isomerase OS=Ophiophagus hann	OPHHA	4
1443	7.24	7.24	55	40	34.44	tr	V8NLU0	V8NLU0_OPHHA	ADP-ribosylation factor-like protein 3 (Fragment) OS=Op	OPHHA	4
1444	7.22	7.22	86.28	26.71	20.94	tr	V8P759	V8P759_OPHHA	DnaJ-like subfamily C member 7 (Fragment) OS=Ophiophagu	OPHHA	5
1445	7.21	7.21	43.92	12.85	8.507	tr	V8P1L3	V8P1L3_OPHHA	RalA-binding protein 1 (Fragment) OS=Ophiophagus hannah	OPHHA	4
1446	7.2	10.54	46.1	33.77	26.95	tr	V8N9R5	V8N9R5_OPHHA	Coatomer subunit gamma-2 (Fragment) OS=Ophiophagus hann	OPHHA	7
1447	7.2	7.2	40.57	25.71	25.71	tr	V8P055	V8P055_OPHHA	NudC domain-containing protein 2 (Fragment) OS=Ophiopha	OPHHA	4
1448	7.19	7.19	63.11	18.45	15.53	tr	V8NNS7	V8NNS7_OPHHA	Guanine nucleotide-binding protein G(S) subunit alpha (	OPHHA	4
1449	7.19	7.19	28.26	12.06	6.522	tr	V8PI43	V8PI43_OPHHA	Glycerate kinase (Fragment) OS=Ophiophagus hannah GN=Gl	OPHHA	4
1450	7.17	7.17	54.25	6.187	2.974	tr	V8NQ11	V8NQ11_OPHHA	Signal-induced proliferation-associated 1-like protein	OPHHA	4
1451	7.17	7.17	34.07	11.72	7.724	tr	V8P3K7	V8P3K7_OPHHA	Elongator complex protein 2 (Fragment) OS=Ophiophagus h	OPHHA	3
1452	7.17	7.17	80.62	29.37	29.37	tr	V8NJ20	V8NJ20_OPHHA	Programmed cell death protein 6 OS=Ophiophagus hannah G	OPHHA	4
1453	7.16	7.16	44.84	26.98	26.98	tr	V8PGJ2	V8PGJ2_OPHHA	Beta-parvin (Fragment) OS=Ophiophagus hannah GN=PARVB P	OPHHA	5
1454	7.13	9.24	60.58	19.23	12.82	tr	V8P798	V8P798_OPHHA	Uncharacterized protein (Fragment) OS=Ophiophagus hanna	OPHHA	4
1455	7.13	7.13	54.17	22.73	17.05	tr	V8NL19	V8NL19_OPHHA	Phosphate carrier protein, mitochondrial OS=Ophiophagus	OPHHA	4
1456	7.13	7.13	60.99	24.11	24.11	tr	V8PDP7	V8PDP7_OPHHA	Tight junction protein ZO-1 (Fragment) OS=Ophiophagus h	OPHHA	4
1457	7.12	7.12	44.54	4.476	4.476	tr	V8NLK9	V8NLK9_OPHHA	Wings apart-like protein-like protein (Fragment) OS=Oph	OPHHA	4
1458	7.12	7.12	52.45	24.51	24.51	tr	V8PED7	V8PED7_OPHHA	Sickle tail protein-like protein (Fragment) OS=Ophiopha	OPHHA	4
1459	7.11	7.11	41.91	9.553	8.012	tr	V8P665	V8P665_OPHHA	Midline-1 OS=Ophiophagus hannah GN=MID1 PE=4 SV=1	OPHHA	4
1460	7.11	7.11	44.57	11.02	7.508	tr	V8NX30	V8NX30_OPHHA	ATP-dependent zinc metalloprotease YME1L1 OS=Ophiophagu	OPHHA	3
1461	7.11	7.11	36.59	15.94	15.94	tr	V8PBK0	V8PBK0_OPHHA	ATP-dependent Clp protease proteolytic subunit (Fragmen	OPHHA	5
1462	7.11	7.11	67.78	51.11	51.11	tr	V8PJF5	V8PJF5_OPHHA	Non-histone chromosomal protein HMG-17 OS=Ophiophagus h	OPHHA	4
1463	7.09	7.09	35.04	21.37	15.1	tr	V8P5W7	V8P5W7_OPHHA	Endophilin-B1 (Fragment) OS=Ophiophagus hannah GN=SH3GL	OPHHA	4
1464	7.09	7.09	53.73	28.86	28.86	tr	V8NWZ5	V8NWZ5_OPHHA	Ras-related protein Rab-22A (Fragment) OS=Ophiophagus h	OPHHA	4
1465	7.09	7.09	39.74	33.11	33.11	tr	V8NKN1	V8NKN1_OPHHA	Malignant T cell-amplified sequence 1-A (Fragment) OS=O	OPHHA	5
1466	7.08	7.08	66.06	17.69	13	tr	V8N2C6	V8N2C6_OPHHA	Uncharacterized protein (Fragment) OS=Ophiophagus hanna	OPHHA	5
1467	7.07	7.07	55.28	15.84	15.84	tr	V8PFH9	V8PFH9_OPHHA	Rhomboid-like protein OS=Ophiophagus hannah GN=PARL PE=	OPHHA	4
1468	7.06	7.06	86.36	35.35	16.67	tr	V8NN24	V8NN24_OPHHA	3-hydroxyisobutyryl-CoA hydrolase, mitochondrial (Fragm	OPHHA	3
1469	7.06	7.06	90.26	42.86	42.86	tr	V8NE40	V8NE40_OPHHA	5'-AMP-activated protein kinase subunit beta-1 (Fragmen	OPHHA	4
1470	7.06	7.06	65.29	38.02	31.4	tr	V8N5P8	V8N5P8_OPHHA	Uncharacterized protein (Fragment) OS=Ophiophagus hanna	OPHHA	4
1471	7.05	11.42	47.45	23.36	14.78	tr	V8P9X6	V8P9X6_OPHHA	Regulator of differentiation 1 (Fragment) OS=Ophiophagu	OPHHA	6
1472	7.05	7.05	69.66	23.45	23.45	tr	V8PIR5	V8PIR5_OPHHA	60S ribosomal protein L26 OS=Ophiophagus hannah GN=RPL2	OPHHA	4
1473	7.05	7.05	55.85	32.45	32.45	tr	V8NU85	V8NU85_OPHHA	Uncharacterized protein OS=Ophiophagus hannah GN=L345_0	OPHHA	5
1474	7.04	9.15	63.32	32.16	27.14	tr	V8P064	V8P064_OPHHA	Peroxiredoxin-1 OS=Ophiophagus hannah GN=PRDX1 PE=4 SV=	OPHHA	5
1475	7.04	8.25	41.29	11.15	6.446	tr	V8NEC5	V8NEC5_OPHHA	Very long-chain acyl-CoA synthetase (Fragment) OS=Ophio	OPHHA	4
1476	7.03	8.11	38.58	18.63	18.63	tr	V8NZQ9	V8NZQ9_OPHHA	Thrombospondin-4 (Fragment) OS=Ophiophagus hannah GN=TH	OPHHA	6
1477	7.03	7.03	51.92	16.38	16.38	tr	V8PAP9	V8PAP9_OPHHA	60S ribosomal protein L6 (Fragment) OS=Ophiophagus hann	OPHHA	4
1478	7.03	7.03	52.73	21.22	18.65	tr	V8PGN1	V8PGN1_OPHHA	Alpha-centractin (Fragment) OS=Ophiophagus hannah GN=AC	OPHHA	4
1479	7.02	7.02	45.83	7.44	3.75	tr	V8P6S2	V8P6S2_OPHHA	Receptor-type tyrosine-protein phosphatase F (Fragment)	OPHHA	4
1480	7.02	7.02	59.42	10.18	8.663	tr	V8NPB2	V8NPB2_OPHHA	E3 ubiquitin-protein ligase MIB2 (Fragment) OS=Ophiopha	OPHHA	4
1481	7.02	7.02	23.55	3.85	3.85	tr	V8P787	V8P787_OPHHA	Cysteine-rich with EGF-like domain protein 1 OS=Ophioph	OPHHA	4
1482	7.01	7.01	34.73	19.46	19.46	tr	V8P5X2	V8P5X2_OPHHA	Nuclear inhibitor of protein phosphatase 1 (Fragment) O	OPHHA	4
1483	7	7	53.55	14.91	6.601	tr	V8P2Z1	V8P2Z1_OPHHA	[3-methyl-2-oxobutanoate dehydrogenase [lipoamide]] kin	OPHHA	4
1484	6.99	7.66	56.9	10.03	5.208	tr	V8P522	V8P522_OPHHA	Protein crooked neck (Fragment) OS=Ophiophagus hannah G	OPHHA	3
1485	6.98	6.98	63.64	16.04	13.1	tr	V8PDR0	V8PDR0_OPHHA	AH receptor-interacting protein (Fragment) OS=Ophiophag	OPHHA	5
1486	6.96	9.58	45.25	29.41	19	tr	V8P072	V8P072_OPHHA	Clathrin light chain A OS=Ophiophagus hannah GN=CLTA PE	OPHHA	8
1487	6.96	6.96	44.91	9.768	5.438	tr	V8PGU8	V8PGU8_OPHHA	Ankyrin repeat and SAM domain-containing protein 1A (Fr	OPHHA	3
1488	6.96	6.96	36.48	8.863	8.863	tr	V8NZ16	V8NZ16_OPHHA	Cytochrome protein (Fragment) OS=Ophiophagus hannah GN=	OPHHA	7
1489	6.96	6.96	62.14	31.43	30.71	tr	V8P1S6	V8P1S6_OPHHA	Ferritin OS=Ophiophagus hannah GN=FTH PE=3 SV=1	OPHHA	5
1490	6.95	6.95	29.87	4.659	4.659	tr	V8NSF0	V8NSF0_OPHHA	Neogenin (Fragment) OS=Ophiophagus hannah GN=NEO1 PE=4	OPHHA	4
1491	6.95	6.95	84.72	31.25	31.25	tr	V8P2F2	V8P2F2_OPHHA	Ubiquitin-conjugating enzyme E2 variant 2 OS=Ophiophagu	OPHHA	5
1492	6.94	7.26	45.54	6.072	3.582	tr	V8NNB3	V8NNB3_OPHHA	Cohesin subunit SA-2 (Fragment) OS=Ophiophagus hannah G	OPHHA	4
1493	6.93	6.94	23.71	19.59	19.59	tr	V8NEH2	V8NEH2_OPHHA	Ubiquinone biosynthesis protein COQ9, mitochondrial (Fr	OPHHA	4
1494	6.93	6.93	45.06	14.1	11.51	tr	V8NMI8	V8NMI8_OPHHA	D-glucuronyl C5-epimerase OS=Ophiophagus hannah GN=GLCE	OPHHA	4
1495	6.93	6.93	33.63	30.97	30.97	tr	V8NF24	V8NF24_OPHHA	Nicalin (Fragment) OS=Ophiophagus hannah GN=NCLN PE=4 S	OPHHA	5
1496	6.92	8.66	82.59	42.11	22.27	tr	V8NED2	V8NED2_OPHHA	Phosphatidylinositol transfer protein alpha isoform (Fr	OPHHA	4
1497	6.92	6.92	58.59	19.82	19.82	tr	V8NQ12	V8NQ12_OPHHA	Hypoxanthine-guanine phosphoribosyltransferase (Fragmen	OPHHA	5
1498	6.92	6.92	51.32	18.86	18.86	tr	V8NAN7	V8NAN7_OPHHA	2-methoxy-6-polyprenyl-1,4-benzoquinol methylase, mitoc	OPHHA	4
1499	6.92	6.92	52.6	20.23	17.34	tr	V8N2G3	V8N2G3_OPHHA	40S ribosomal protein S25 (Fragment) OS=Ophiophagus han	OPHHA	6
1500	6.9	6.93	33.92	5.019	3.726	tr	V8P555	V8P555_OPHHA	Phosphorylase b kinase regulatory subunit alpha, skelet	OPHHA	4
1501	6.9	6.9	62.7	24.6	24.6	tr	V8NF52	V8NF52_OPHHA	Protein FAM49B (Fragment) OS=Ophiophagus hannah GN=FAM4	OPHHA	4
1502	6.89	6.89	38.16	16.89	14.47	tr	V8NTW8	V8NTW8_OPHHA	Splicing factor 1 (Fragment) OS=Ophiophagus hannah GN=S	OPHHA	5
1503	6.89	6.89	54.82	28.92	24.1	tr	V8NDF2	V8NDF2_OPHHA	Histone H2A (Fragment) OS=Ophiophagus hannah GN=H2AFV P	OPHHA	4
1504	6.89	6.89	42.22	32.22	32.22	tr	V8NGI6	V8NGI6_OPHHA	Phosphomevalonate kinase (Fragment) OS=Ophiophagus hann	OPHHA	5
1505	6.88	6.88	49.38	34.57	33.33	tr	V8P2Y8	V8P2Y8_OPHHA	Eukaryotic translation initiation factor 1 (Fragment) O	OPHHA	4
1505	0	4.77	24.44	23.7	22.22	tr	V8N7D6	V8N7D6_OPHHA	Eukaryotic translation initiation factor 1b (Fragment)	OPHHA	2
1506	6.87	6.87	33.27	6.518	4.28	tr	V8NZZ8	V8NZZ8_OPHHA	L-fucose kinase (Fragment) OS=Ophiophagus hannah GN=FUK	OPHHA	3
1507	6.87	6.87	51.96	26.96	26.96	tr	V8NKC2	V8NKC2_OPHHA	Tumor protein D53-like protein (Fragment) OS=Ophiophagu	OPHHA	6
1508	6.85	9.08	64.78	19.92	13.84	tr	V8NJH1	V8NJH1_OPHHA	Copine-3 OS=Ophiophagus hannah GN=Cpne3 PE=4 SV=1	OPHHA	5
1509	6.84	7.25	30.95	15.48	12.3	tr	V8NNB5	V8NNB5_OPHHA	Heterogeneous nuclear ribonucleoproteins A2/B1 (Fragmen	OPHHA	4
1510	6.83	6.83	51.55	8.044	6.307	tr	V8NYZ6	V8NYZ6_OPHHA	FERM, RhoGEF and pleckstrin domain-containing protein 2	OPHHA	4
1511	6.82	6.82	54.27	8.868	6.41	tr	V8PJV5	V8PJV5_OPHHA	Oxysterol-binding protein (Fragment) OS=Ophiophagus han	OPHHA	4
1512	6.82	6.82	41.31	10.37	6.86	tr	V8P153	V8P153_OPHHA	Complement component C6 OS=Ophiophagus hannah GN=C6 PE=	OPHHA	3
1513	6.82	6.82	31.18	6.12	6.12	tr	V8N7Y3	V8N7Y3_OPHHA	Vascular non-inflammatory molecule 2 (Fragment) OS=Ophi	OPHHA	4
1514	6.82	6.82	22.52	10.9	10.9	tr	V8NVC8	V8NVC8_OPHHA	Proteasomal ubiquitin receptor ADRM1 OS=Ophiophagus han	OPHHA	4
1515	6.81	7.45	48.39	7.701	6.897	tr	V8NSK5	V8NSK5_OPHHA	Protein phosphatase 1 regulatory subunit 12C (Fragment)	OPHHA	4
1516	6.81	6.81	48.87	48.87	24.81	tr	V8N975	V8N975_OPHHA	EH domain-containing protein 3 (Fragment) OS=Ophiophagu	OPHHA	4
1517	6.8	8.46	51.83	17.43	9.021	tr	V8NM10	V8NM10_OPHHA	Alpha-adducin (Fragment) OS=Ophiophagus hannah GN=ADD1	OPHHA	5
1518	6.8	6.8	74.12	55.29	55.29	tr	V8NIS3	V8NIS3_OPHHA	Programmed cell death protein 5 OS=Ophiophagus hannah G	OPHHA	5
1519	6.79	6.86	33.33	7.589	5.804	tr	V8N9T4	V8N9T4_OPHHA	Amyloid beta A4 protein-binding family B member 1-inter	OPHHA	4
1520	6.78	7.97	34.6	21.56	12.09	tr	V8NG07	V8NG07_OPHHA	Actin-like protein 6A (Fragment) OS=Ophiophagus hannah	OPHHA	5
1521	6.78	7.17	54.61	14.54	8.511	tr	V8NCD9	V8NCD9_OPHHA	N-acetylneuraminate lyase (Fragment) OS=Ophiophagus han	OPHHA	4
1522	6.78	6.78	66.95	24.27	20.5	tr	V8PC25	V8PC25_OPHHA	Ribose-5-phosphate isomerase (Fragment) OS=Ophiophagus	OPHHA	5
1523	6.78	6.78	46.05	34.21	28.95	tr	V8NIM7	V8NIM7_OPHHA	Protein C20orf11-like protein OS=Ophiophagus hannah GN=	OPHHA	4
1524	6.77	6.77	62.95	6.906	5.046	tr	V8NN62	V8NN62_OPHHA	LIM domain and actin-binding protein 1 OS=Ophiophagus h	OPHHA	3
1525	6.77	6.77	39.05	12.19	12.19	tr	V8N8V4	V8N8V4_OPHHA	Uncharacterized protein (Fragment) OS=Ophiophagus hanna	OPHHA	4
1526	6.76	11.26	69.27	38.02	29.69	tr	V8P053	V8P053_OPHHA	Serine/arginine-rich splicing factor 1 (Fragment) OS=Op	OPHHA	5
1527	6.74	6.74	40.41	7.37	5.337	tr	V8PBK8	V8PBK8_OPHHA	Sister chromatid cohesion protein PDS5-like A (Fragment	OPHHA	3
1528	6.74	6.74	45.8	20.17	12.18	tr	V8NLA7	V8NLA7_OPHHA	Trafficking protein particle complex subunit 3 (Fragmen	OPHHA	3

1529	6.74	6.74	68.54	23.97	23.97 tr|V8PI86|V8PI86_OPHHA Zinc finger protein (Fragment) OS=Ophiophagus hannah GN	OPHHA	5
1530	6.74	6.74	58.93	33.33	23.21 tr|V8NIK8|V8NIK8_OPHHA Glutaredoxin-related protein 5, mitochondrial (Fragment	OPHHA	5
1531	6.72	10.93	63.31	18.93	18.93 tr|V8NG70|V8NG70_OPHHA Protein kinase C alpha type (Fragment) OS=Ophiophagus h	OPHHA	7
1532	6.72	6.72	44.26	9.715	5.103 tr|V8P9I3|V8P9I3_OPHHA Rho GTPase-activating protein 4 (Fragment) OS=Ophiophag	OPHHA	4
1533	6.71	6.71	36.36	24.24	17.58 tr|V8NFJ5|V8NFJ5_OPHHA Ras GTPase-activating-like protein IQGAP1 (Fragment) OS	OPHHA	4
1534	6.7	6.75	50.96	11.27	6.235 tr|V8NX10|V8NX10_OPHHA WD repeat and FYVE domain-containing protein 1 OS=Ophio	OPHHA	4
1535	6.69	6.72	38.53	14.42	12.77 tr|V8N3T6|V8N3T6_OPHHA Thimet oligopeptidase (Fragment) OS=Ophiophagus hannah	OPHHA	4
1536	6.69	6.69	58.66	4.398	3.078 tr|V8PJ36|V8PJ36_OPHHA 5-azacytidine-induced protein 1 OS=Ophiophagus hannah G	OPHHA	3
1537	6.68	6.68	57.92	13.8	9.502 tr|V8PDC5|V8PDC5_OPHHA Inositol polyphosphate 1-phosphatase (Fragment) OS=Ophi	OPHHA	3
1538	6.68	6.68	46.52	16.96	9.13 tr|V8NY27|V8NY27_OPHHA Peptidyl-prolyl cis-trans isomerase-like 4 OS=Ophiophag	OPHHA	3
1539	6.66	6.66	53.59	16.99	11.48 tr|V8NRM6|V8NRM6_OPHHA Delta(24)-sterol reductase OS=Ophiophagus hannah GN=Dhc	OPHHA	4
1540	6.66	6.66	32.62	17.38	17.38 tr|V8NC05|V8NC05_OPHHA Tetratricopeptide repeat protein 38 (Fragment) OS=Ophio	OPHHA	5
1541	6.65	6.65	55.56	28.24	23.15 tr|V8NJS7|V8NJS7_OPHHA Tyrosine-protein kinase CSK (Fragment) OS=Ophiophagus h	OPHHA	3
1542	6.65	6.65	60.34	36.31	26.26 tr|V8NAL6|V8NAL6_OPHHA 26S proteasome non-ATPase regulatory subunit 8 (Fragmen	OPHHA	3
1543	6.64	7.61	44.71	14.18	12.02 tr|V8NV52|V8NV52_OPHHA Uncharacterized protein (Fragment) OS=Ophiophagus hanna	OPHHA	5
1544	6.64	6.64	52.01	12.07	12.07 tr|V8NIT0|V8NIT0_OPHHA Ras-related protein Rab-11B (Fragment) OS=Ophiophagus h	OPHHA	4
1544	0.03	5.15	50.74	12.59	12.59 tr|V8NJW9|V8NJW9_OPHHA Ras-related protein Rab-11B (Fragment) OS=Ophiophagus h	OPHHA	3
1545	6.64	6.64	61.92	19.62	15.77 tr|V8PH96|V8PH96_OPHHA Transcription elongation factor A protein 1 OS=Ophiopha	OPHHA	4
1546	6.63	6.63	39.68	5.864	3.656 tr|V8PIX6|V8PIX6_OPHHA Calmodulin-regulated spectrin-associated protein 3 OS=O	OPHHA	3
1547	6.63	6.63	44.99	10.51	7.882 tr|V8NVT2|V8NVT2_OPHHA Nuclear pore complex protein Nup93 OS=Ophiophagus hanna	OPHHA	4
1548	6.63	6.63	55.78	27.21	16.33 tr|V8NEG8|V8NEG8_OPHHA Cell differentiation protein RCD1-like protein OS=Ophio	OPHHA	4
1549	6.62	6.62	48.62	15.69	12.92 tr|V8PEC2|V8PEC2_OPHHA Replication factor C subunit 2 (Fragment) OS=Ophiophagu	OPHHA	3
1550	6.61	6.61	46.83	18.25	11.11 tr|V8NQQ7|V8NQQ7_OPHHA Malonyl-CoA decarboxylase, mitochondrial (Fragment) OS=	OPHHA	3
1551	6.61	6.61	48.8	19.16	17.07 tr|V8NR53|V8NR53_OPHHA Phytanoyl-CoA dioxygenase domain-containing protein 1 (	OPHHA	3
1552	6.6	6.6	55.61	22.43	22.43 tr|V8NG88|V8NG88_OPHHA 5'-AMP-activated protein kinase subunit beta-2 (Fragmen	OPHHA	4
1553	6.59	6.59	45.83	12	8.833 tr|V8NWP4|V8NWP4_OPHHA Inhibitor of nuclear factor kappa-B kinase subunit beta	OPHHA	4
1554	6.59	6.59	29.04	6.933	3.507 tr|V8NN87|V8NN87_OPHHA Antigen (Fragment) OS=Ophiophagus hannah GN=CD109 PE=4	OPHHA	3
1555	6.59	6.59	35.76	15.95	11.16 tr|V8NMM7|V8NMM7_OPHHA Serine palmitoyltransferase 1 OS=Ophiophagus hannah GN=	OPHHA	4
1556	6.56	6.56	39.5	11.27	6.88 tr|V8PIF5|V8PIF5_OPHHA Diacylglycerol kinase (Fragment) OS=Ophiophagus hannah	OPHHA	4
1557	6.56	6.56	46.72	12.09	10.66 tr|V8PAZ5|V8PAZ5_OPHHA Phosphoacetylglucosamine mutase (Fragment) OS=Ophiophag	OPHHA	4
1558	6.55	6.55	34.57	23.4	23.4 tr|V8P8W4|V8P8W4_OPHHA PRA1 family protein 3 OS=Ophiophagus hannah GN=ARL6IP5	OPHHA	4
1559	6.54	6.54	52.01	10.12	5.825 tr|V8NMF8|V8NMF8_OPHHA Pre-mRNA-processing factor 40-like A (Fragment) OS=Ophi	OPHHA	3
1560	6.53	6.53	45.17	17.76	12.77 tr|V8NAB7|V8NAB7_OPHHA Uncharacterized protein (Fragment) OS=Ophiophagus hanna	OPHHA	3
1561	6.52	6.52	57.87	12.06	7.168 tr|V8ND02|V8ND02_OPHHA Dynactin subunit 1 (Fragment) OS=Ophiophagus hannah GN=	OPHHA	3
1562	6.52	6.52	48.04	12.69	12.39 tr|V8NVY1|V8NVY1_OPHHA PDZ domain-containing protein GIPC1 OS=Ophiophagus hann	OPHHA	3
1563	6.51	6.53	42.47	4.61	2.766 tr|V8P9J1|V8P9J1_OPHHA Arf-GAP with SH3 domain, ANK repeat and PH domain-conta	OPHHA	3
1564	6.51	6.52	70.59	28.57	28.57 tr|V8NSS7|V8NSS7_OPHHA 40S ribosomal protein S20 OS=Ophiophagus hannah GN=rps2	OPHHA	3
1565	6.5	6.5	43.77	13.13	9.259 tr|V8PHM1|V8PHM1_OPHHA Nuclear protein localization protein 4-like protein (Fr	OPHHA	3
1566	6.46	6.46	51.8	27.48	23.42 tr|V8N9A4|V8N9A4_OPHHA Acetolactate synthase-like protein (Fragment) OS=Ophiop	OPHHA	3
1567	6.46	6.46	29.13	24.27	19.42 tr|V8P0Y4|V8P0Y4_OPHHA 26S proteasome non-ATPase regulatory subunit 7 (Fragmen	OPHHA	3
1568	6.45	6.45	44.4	12.2	9.078 tr|V8P5K0|V8P5K0_OPHHA Extended synaptotagmin-2-B (Fragment) OS=Ophiophagus ha	OPHHA	4
1569	6.44	6.44	41.32	5.933	4.432 tr|V8NY58|V8NY58_OPHHA Intron-binding protein aquarius OS=Ophiophagus hannah G	OPHHA	4
1570	6.44	6.44	37.64	6.383	4.419 tr|V8P2U1|V8P2U1_OPHHA Ornithine decarboxylase 2 (Fragment) OS=Ophiophagus han	OPHHA	4
1571	6.44	6.44	48.84	12.79	7.752 tr|V8PAK3|V8PAK3_OPHHA Pyridine nucleotide-disulfide oxidoreductase domain-con	OPHHA	3
1572	6.44	6.44	37.29	20.34	15.59 tr|V8P6F1|V8P6F1_OPHHA Poly(RC)-binding protein 3 (Fragment) OS=Ophiophagus ha	OPHHA	4
1573	6.43	6.43	56	26.5	22 tr|V8NBY3|V8NBY3_OPHHA UV excision repair protein RAD23-like B OS=Ophiophagus	OPHHA	6
1574	6.42	17.74	61.26	16.23	15.05 tr|V8NQ85|V8NQ85_OPHHA Kinesin light chain 1 (Fragment) OS=Ophiophagus hannah	OPHHA	10
1575	6.42	7.72	49.87	6.774	2.879 tr|V8P6U8|V8P6U8_OPHHA Vigilin OS=Ophiophagus hannah GN=HDLBP PE=4 SV=1	OPHHA	4
1576	6.42	6.42	73.71	29.38	20.62 tr|V8PB12|V8PB12_OPHHA Synaptobrevin-like YKT6-B OS=Ophiophagus hannah GN=ykt6	OPHHA	3
1577	6.41	6.42	44.24	16.62	14.21 tr|V8NI66|V8NI66_OPHHA Syntaxin-5 (Fragment) OS=Ophiophagus hannah GN=STX5 PE=	OPHHA	4
1578	6.41	6.41	47.23	13.06	8.766 tr|V8NC40|V8NC40_OPHHA Dipeptidyl peptidase 9 (Fragment) OS=Ophiophagus hannah	OPHHA	4
1579	6.41	6.41	33.33	33.33	28.07 tr|V8PF68|V8PF68_OPHHA Enhancer of rudimentary homolog OS=Ophiophagus hannah G	OPHHA	5
1580	6.4	6.65	54.59	11.47	7.126 tr|V8P1R2|V8P1R2_OPHHA Dystrobrevin alpha (Fragment) OS=Ophiophagus hannah GN=	OPHHA	4
1581	6.4	6.52	67.27	20.36	18.55 tr|V8PH30|V8PH30_OPHHA WD repeat-containing protein 82 (Fragment) OS=Ophiophag	OPHHA	4
1582	6.4	6.4	44.87	3.865	2.415 tr|V8P7S1|V8P7S1_OPHHA Cortactin-binding protein 2 OS=Ophiophagus hannah GN=CT	OPHHA	4
1583	6.4	6.4	36.69	8.633	7.374 tr|V8P2J8|V8P2J8_OPHHA Tyrosine aminotransferase OS=Ophiophagus hannah GN=Tat	OPHHA	3
1584	6.4	6.4	32.23	15.67	12.36 tr|V8NXS1|V8NXS1_OPHHA RNA-binding protein 42 (Fragment) OS=Ophiophagus hannah	OPHHA	3
1585	6.39	11.16	50.74	25	16.09 tr|V8NZ79|V8NZ79_OPHHA Serine/threonine-protein kinase 24 (Fragment) OS=Ophiop	OPHHA	5
1586	6.39	6.39	32.96	5.817	4.848 tr|V8P4J0|V8P4J0_OPHHA Poly(A) polymerase alpha OS=Ophiophagus hannah GN=PAPOL	OPHHA	3
1587	6.39	6.39	44.06	17.94	7.652 tr|V8NKK0|V8NKK0_OPHHA Abl interactor 1 (Fragment) OS=Ophiophagus hannah GN=Ab	OPHHA	3
1588	6.38	6.42	52.62	4.499	2.59 tr|V8NJM4|V8NJM4_OPHHA DNA topoisomerase 2 (Fragment) OS=Ophiophagus hannah GN	OPHHA	3
1589	6.38	6.38	38.04	20.25	15.03 tr|V8PDL2|V8PDL2_OPHHA Uroporphyrinogen decarboxylase (Fragment) OS=Ophiophagu	OPHHA	4
1590	6.38	6.38	56.04	17.22	13.92 tr|V8PBS2|V8PBS2_OPHHA 60S ribosomal protein L23 OS=Ophiophagus hannah GN=RPL2	OPHHA	4
1591	6.37	6.37	29.26	7.882	4.138 tr|V8NJ15|V8NJ15_OPHHA Apoptosis-stimulating of p53 protein 2 (Fragment) OS=Op	OPHHA	3
1592	6.37	6.37	36.68	17.3	13.15 tr|V8P4N4|V8P4N4_OPHHA Inosine-uridine preferring nucleoside hydrolase (Fragme	OPHHA	4
1593	6.36	16.84	62.29	27.54	27.54 tr|V8PDL7|V8PDL7_OPHHA Gamma-enolase (Fragment) OS=Ophiophagus hannah GN=ENO2	OPHHA	11
1594	6.36	6.36	29.38	6.623	6.623 tr|V8NPL6|V8NPL6_OPHHA Serine/threonine-protein phosphatase 6 regulatory ankyr	OPHHA	4
1595	6.35	6.55	32.78	7.851	4.024 tr|V8P3F2|V8P3F2_OPHHA Pikachurin (Fragment) OS=Ophiophagus hannah GN=EGFLAM P	OPHHA	3
1596	6.35	6.35	73.24	15.41	12.43 tr|V8N5V9|V8N5V9_OPHHA Uncharacterized protein (Fragment) OS=Ophiophagus hanna	OPHHA	4
1597	6.34	6.34	84.38	31.87	27.5 tr|V8PC39|V8PC39_OPHHA 60S ribosomal protein L21 OS=Ophiophagus hannah GN=RPL2	OPHHA	4
1598	6.34	6.34	30.37	20.25	17.48 tr|V8PCR6|V8PCR6_OPHHA Cytosolic Fe-S cluster assembly factor NUBP1 OS=Ophioph	OPHHA	3
1599	6.34	6.34	77.98	45.87	38.53 tr|V8N801|V8N801_OPHHA Uncharacterized protein (Fragment) OS=Ophiophagus hanna	OPHHA	3
1600	6.33	6.33	67.65	30.88	21.57 tr|V8N9Z3|V8N9Z3_OPHHA Ribosomal protein L15 OS=Ophiophagus hannah GN=RPL15 PE	OPHHA	5
1601	6.33	6.33	47.04	13.24	11.11 tr|V8NPQ8|V8NPQ8_OPHHA Laminin subunit alpha-5 (Fragment) OS=Ophiophagus hanna	OPHHA	3
1602	6.33	6.33	31.58	19.65	16.14 tr|V8PH42|V8PH42_OPHHA CCR4-NOT transcription complex subunit 7 (Fragment) OS=	OPHHA	3
1603	6.32	6.32	59.79	17.99	16.4 tr|V8P9N3|V8P9N3_OPHHA Thioredoxin domain-containing protein 5 OS=Ophiophagus	OPHHA	3
1604	6.31	6.31	56.3	26.39	16.72 tr|V8PAK1|V8PAK1_OPHHA Coiled-coil domain-containing protein OS=Ophiophagus ha	OPHHA	3
1605	6.31	6.31	43.43	19.19	13.47 tr|V8N5W2|V8N5W2_OPHHA Vinexin (Fragment) OS=Ophiophagus hannah GN=Sorbs3 PE=4	OPHHA	3
1606	6.3	6.3	41.77	3.836	3.069 tr|V8P890|V8P890_OPHHA Pleckstrin-likey domain-containing family A member 5 (F	OPHHA	3
1607	6.3	6.3	33.86	20.63	20.11 tr|V8PE08|V8PE08_OPHHA NADH dehydrogenase [ubiquinone] iron-sulfur protein 4,	OPHHA	5
1608	6.29	13.67	44.98	25.34	15.3 tr|V8P9A2|V8P9A2_OPHHA Septin-11 (Fragment) OS=Ophiophagus hannah GN=SEPT11 PE	OPHHA	6
1609	6.29	6.29	63.09	20.17	19.74 tr|V8NYP1|V8NYP1_OPHHA Vesicle-associated membrane protein-associated protein	OPHHA	6
1610	6.29	6.29	43.75	37.5	27.5 tr|V8NA78|V8NA78_OPHHA Iron-sulfur cluster assembly 1-like, mitochondrial (Fra	OPHHA	3
1611	6.28	6.28	79.63	41.67	26.85 tr|V8NQ20|V8NQ20_OPHHA CDGSH iron-sulfur domain-containing protein 1 OS=Ophiop	OPHHA	5
1612	6.26	6.29	42.47	6.365	3.794 tr|V8P829|V8P829_OPHHA Plexin-A1 (Fragment) OS=Ophiophagus hannah GN=Plxna1 PE	OPHHA	3
1612	0.02	5.53	48.31	2.753	2.753 tr|V8NMA3|V8NMA3_OPHHA Plexin-A4 (Fragment) OS=Ophiophagus hannah GN=Plxna4 PE	OPHHA	3
1613	6.26	6.26	62.57	13.71	10.86 tr|V8N7E8|V8N7E8_OPHHA Dysferlin (Fragment) OS=Ophiophagus hannah GN=Dysf PE=4	OPHHA	3
1614	6.26	6.26	36.66	12.06	7.657 tr|V8PBF0|V8PBF0_OPHHA Carboxymethylenebutenolidase-like protein (Fragment) OS	OPHHA	4
1615	6.26	6.26	34.08	11.8	9.8 tr|V8P5B6|V8P5B6_OPHHA Antigen (Fragment) OS=Ophiophagus hannah GN=ALCAM PE=4	OPHHA	3
1616	6.26	6.26	28.09	12.58	7.757 tr|V8P270|V8P270_OPHHA Serine/threonine-protein phosphatase 2A 56 kDa regulato	OPHHA	3
1617	6.26	6.26	79.81	37.5	27.88 tr|V8P3S5|V8P3S5_OPHHA Ras-related protein Rab-21 OS=Ophiophagus hannah GN=Rab	OPHHA	3
1618	6.25	6.27	42.32	4.159	2.481 tr|V8PEU2|V8PEU2_OPHHA Sacsin (Fragment) OS=Ophiophagus hannah GN=SACS PE=4 SV	OPHHA	7
1619	6.25	6.25	52.46	9.275	4.783 tr|V8NE31|V8NE31_OPHHA Putative leucine-rich repeat-containing protein (Fragme	OPHHA	3
1620	6.25	6.25	45.79	7.477	6.008 tr|V8P4S3|V8P4S3_OPHHA Putative ATP-dependent RNA helicase DDX58 OS=Ophiophagu	OPHHA	5
1621	6.25	6.25	65.8	28.5	28.5 tr|V8NGD4|V8NGD4_OPHHA Cysteine and glycine-rich protein 2 OS=Ophiophagus hann	OPHHA	4
1622	6.24	6.24	51.51	6.763	4.892 tr|V8NAJ6|V8NAJ6_OPHHA Ena/VASP-like protein OS=Ophiophagus hannah GN=Evl PE=4	OPHHA	3

1623	6.23	6.23	54.64	9.731	7.934	tr|V8PCF0|V8PCF0_OPHHA	Sulfotransferase 1C2 (Fragment) OS=Ophiophagus hannah G	OPHHA	3
1624	6.23	6.23	55.36	16.31	16.31	tr|V8NPG2|V8NPG2_OPHHA	THUMP domain-containing protein 1 (Fragment) OS=Ophioph	OPHHA	4
1625	6.22	6.23	34.7	8.499	7.649	tr|V8N6N4|V8N6N4_OPHHA	Antigen peptide transporter 1 (Fragment) OS=Ophiophagus	OPHHA	4
1626	6.22	6.22	33.92	7.941	6.326	tr|V8PAE9|V8PAE9_OPHHA	Ubiquitin-like modifier-activating enzyme 1 (Fragment)	OPHHA	5
1627	6.22	6.22	64.53	25.58	19.77	tr|V8NJN7|V8NJN7_OPHHA	Proteasome subunit beta type-7 (Fragment) OS=Ophiophagu	OPHHA	3
1628	6.21	6.21	28.54	7.029	5.857	tr|V8NKG2|V8NKG2_OPHHA	Fibronectin type-III domain-containing protein 3a (Frag	OPHHA	3
1629	6.21	6.21	65.2	26.96	11.91	tr|V8PE13|V8PE13_OPHHA	Developmentally-regulated GTP-binding protein 2 OS=Ophi	OPHHA	3
1630	6.21	6.21	61.78	20.67	15.87	tr|V8NHZ7|V8NHZ7_OPHHA	Paralemmin-1 (Fragment) OS=Ophiophagus hannah GN=PALM P	OPHHA	5
1631	6.2	6.2	36.56	6.503	4.745	tr|V8PID1|V8PID1_OPHHA	CUGBP Elav-like family member 1 OS=Ophiophagus hannah G	OPHHA	3
1632	6.19	6.19	47.09	9.419	7.615	tr|V8N9U9|V8N9U9_OPHHA	Atlastin-3 (Fragment) OS=Ophiophagus hannah GN=Atl3 PE=	OPHHA	4
1633	6.19	6.19	58.12	18.38	18.38	tr|V8P2Q7|V8P2Q7_OPHHA	Proliferating cell nuclear antigen OS=Ophiophagus hanna	OPHHA	3
1634	6.19	6.19	83.93	52.68	39.29	tr|V8NH75|V8NH75_OPHHA	V-type proton ATPase subunit F (Fragment) OS=Ophiophagu	OPHHA	3
1635	6.16	6.29	57.76	13.7	10.05	tr|V8NME0|V8NME0_OPHHA	Luc7-like protein 3 OS=Ophiophagus hannah GN=LUC7L3 PE=	OPHHA	3
1636	6.16	6.16	45.07	4.876	4.179	tr|V8PBX0|V8PBX0_OPHHA	Alpha-amylase (Fragment) OS=Ophiophagus hannah GN=Amy2	OPHHA	3
1637	6.16	6.16	62.44	21.32	21.32	tr|V8NQ62|V8NQ62_OPHHA	Prostaglandin E synthase 3 (Fragment) OS=Ophiophagus ha	OPHHA	6
1638	6.16	6.16	60.8	25.6	25.6	tr|V8NQ46|V8NQ46_OPHHA	60S ribosomal protein L31 OS=Ophiophagus hannah GN=RPL3	OPHHA	3
1639	6.15	6.15	69.36	12.42	10.35	tr|V8PAI7|V8PAI7_OPHHA	SURP and G-patch domain-containing protein 1 (Fragment)	OPHHA	4
1640	6.15	6.15	43.32	18.99	13.95	tr|V8NEF5|V8NEF5_OPHHA	Dihydrolipoyllysine-residue acetyltransferase component	OPHHA	3
1641	6.14	8.17	76.54	16.87	16.87	tr|V8NF78|V8NF78_OPHHA	Decorin (Fragment) OS=Ophiophagus hannah GN=DCN PE=4 SV	OPHHA	4
1642	6.14	6.14	34.33	7.794	7.794	tr|V8NDH0|V8NDH0_OPHHA	Eukaryotic translation initiation factor 4 gamma 3 (Fra	OPHHA	3
1643	6.14	6.14	58.22	21.05	14.8	tr|V8NL73|V8NL73_OPHHA	Extracellular matrix protein 1 (Fragment) OS=Ophiophagu	OPHHA	3
1644	6.12	6.13	38.39	7.1	5.415	tr|V8PHB7|V8PHB7_OPHHA	Trafficking protein particle complex subunit 12 (Fragme	OPHHA	3
1645	6.12	6.12	49.79	10.7	5.487	tr|V8P0S1|V8P0S1_OPHHA	Leucine-rich repeat-containing protein 8D (Fragment) OS	OPHHA	3
1646	6.12	6.12	54.79	12.79	9.589	tr|V8ND66|V8ND66_OPHHA	Dynactin subunit 1 (Fragment) OS=Ophiophagus hannah GN=	OPHHA	4
1647	6.12	6.12	40.72	12.74	8.033	tr|V8P695|V8P695_OPHHA	Protein XRP2 OS=Ophiophagus hannah GN=RP2 PE=3 SV=1	OPHHA	3
1648	6.12	6.12	73.68	21.53	18.18	tr|V8NRL8|V8NRL8_OPHHA	Malectin OS=Ophiophagus hannah GN=MLEC PE=4 SV=1	OPHHA	3
1649	6.12	6.12	33.09	17.28	14.34	tr|V8NP91|V8NP91_OPHHA	Serum paraoxonase/arylesterase 2 OS=Ophiophagus hannah	OPHHA	6
1650	6.1	7.16	55.84	7.721	4.143	tr|V8NQM8|V8NQM8_OPHHA	EH domain-binding protein 1 (Fragment) OS=Ophiophagus h	OPHHA	4
1651	6.1	6.12	37.06	5.637	5.637	tr|V8PFU6|V8PFU6_OPHHA	C-Jun-amino-terminal kinase-interacting protein 4 OS=Op	OPHHA	4
1652	6.1	6.1	60.19	13.79	13.79	tr|V8NV08|V8NV08_OPHHA	ADP-ribose pyrophosphatase, mitochondrial OS=Ophiophagu	OPHHA	4
1653	6.1	6.1	48.1	12.36	12.36	tr|V8NWB4|V8NWB4_OPHHA	Mothers against decapentaplegic homolog (Fragment) OS=O	OPHHA	3
1654	6.09	6.09	58.19	11.34	11.34	tr|V8P6I5|V8P6I5_OPHHA	Transmembrane and coiled-coil domains protein 3 (Fragme	OPHHA	3
1655	6.09	6.09	38.63	8.155	8.155	tr|V8P4R3|V8P4R3_OPHHA	Protein disulfide-isomerase TMX3 (Fragment) OS=Ophiopha	OPHHA	3
1656	6.08	6.11	50.64	8.227	3.291	tr|V8P973|V8P973_OPHHA	Cytospin-A OS=Ophiophagus hannah GN=SPECC1L PE=4 SV=1	OPHHA	3
1657	6.08	6.08	53.01	9.452	6.849	tr|V8P4A8|V8P4A8_OPHHA	Paraplegin (Fragment) OS=Ophiophagus hannah GN=Spg7 PE=	OPHHA	3
1658	6.08	6.08	56.45	18.82	18.82	tr|V8P627|V8P627_OPHHA	Spermidine synthase (Fragment) OS=Ophiophagus hannah GN	OPHHA	3
1659	6.07	13.02	33.76	25.85	19.87	tr|V8NGF1|V8NGF1_OPHHA	Importin subunit alpha-3 (Fragment) OS=Ophiophagus hann	OPHHA	6
1660	6.07	6.08	44.57	19.29	11.14	tr|V8PFK0|V8PFK0_OPHHA	cGMP-dependent protein kinase 1 (Fragment) OS=Ophiophag	OPHHA	4
1661	6.07	6.07	32.29	5.643	5.016	tr|V8P3L8|V8P3L8_OPHHA	Importin-11 (Fragment) OS=Ophiophagus hannah GN=IPO11 P	OPHHA	3
1662	6.07	6.07	20.24	5.283	3.199	tr|V8NUW4|V8NUW4_OPHHA	Latent-transforming growth factor beta-binding protein	OPHHA	3
1663	6.07	6.07	42.95	10.07	10.07	tr|V8P0T8|V8P0T8_OPHHA	Coagulation factor XIII A chain (Fragment) OS=Ophiophag	OPHHA	3
1664	6.07	6.07	31.93	14.71	14.71	tr|V8NBH5|V8NBH5_OPHHA	Acyl-CoA dehydrogenase family member 11 OS=Ophiophagus	OPHHA	3
1665	6.06	7.63	28.72	10.24	10.24	tr|V8PF08|V8PF08_OPHHA	Sodium/glucose cotransporter 5 (Fragment) OS=Ophiophagu	OPHHA	5
1666	6.06	6.06	77.87	13.73	10.92	tr|V8NBC8|V8NBC8_OPHHA	Egl nine-like 1 (Fragment) OS=Ophiophagus hannah GN=EGL	OPHHA	3
1667	6.06	6.06	54.96	16.35	11.8	tr|V8NQB3|V8NQB3_OPHHA	Long-chain-fatty-acid--CoA ligase 5 OS=Ophiophagus hann	OPHHA	4
1668	6.06	6.06	49.13	16.38	13.59	tr|V8NUQ3|V8NUQ3_OPHHA	Mitochondrial-processing peptidase subunit alpha (Fragm	OPHHA	3
1669	6.06	6.06	39.86	13.17	13.17	tr|V8NE57|V8NE57_OPHHA	Serine/threonine-protein phosphatase (Fragment) OS=Ophi	OPHHA	3
1670	6.05	6.14	36.43	13.82	11.56	tr|V8N9F0|V8N9F0_OPHHA	Protein TFG OS=Ophiophagus hannah GN=TFG PE=4 SV=1	OPHHA	4
1671	6.05	6.06	53.99	12.55	9.506	tr|V8NAJ7|V8NAJ7_OPHHA	3-ketoacyl-CoA thiolase, mitochondrial (Fragment) OS=Op	OPHHA	3
1672	6.05	6.05	64.29	14.94	14.94	tr|V8NUV3|V8NUV3_OPHHA	40S ribosomal protein S23 (Fragment) OS=Ophiophagus han	OPHHA	3
1672	0	6.05	63.64	16.08	16.08	tr|V8NW17|V8NW17_OPHHA	40S ribosomal protein S23 OS=Ophiophagus hannah GN=RPS2	OPHHA	3
1673	6.05	6.05	34.29	15.71	13.14	tr|V8P7Q9|V8P7Q9_OPHHA	Complement component C8 alpha chain (Fragment) OS=Ophio	OPHHA	5
1674	6.04	6.07	64.77	24.83	11.74	tr|V8NUE2|V8NUE2_OPHHA	Enoyl-CoA hydratase domain-containing protein 1 (Fragme	OPHHA	3
1675	6.04	6.04	47.62	8.617	8.617	tr|V8NEZ7|V8NEZ7_OPHHA	Uncharacterized protein (Fragment) OS=Ophiophagus hanna	OPHHA	3
1676	6.04	6.04	53.33	21.28	12.82	tr|V8P068|V8P068_OPHHA	Phosphorylated adapter RNA export protein (Fragment) OS	OPHHA	3
1677	6.04	6.04	90.84	35.88	35.88	tr|V8N6B8|V8N6B8_OPHHA	Succinate dehydrogenase [ubiquinone] iron-sulfur subuni	OPHHA	6
1678	6.04	6.04	30.57	14.51	14.51	tr|V8PC01|V8PC01_OPHHA	Uncharacterized protein (Fragment) OS=Ophiophagus hanna	OPHHA	3
1679	6.03	30.74	60.56	47.5	47.5	tr|V8N532|V8N532_OPHHA	Tubulin beta-2A chain OS=Ophiophagus hannah GN=TUBB2A P	OPHHA	24
1680	6.03	10.1	45.42	29.01	25.57	tr|V8PGX8|V8PGX8_OPHHA	Guanine nucleotide-binding protein G(K) subunit alpha (	OPHHA	5
1681	6.03	6.03	61.47	22.48	5.963	tr|V8NT73|V8NT73_OPHHA	Coproporphyrinogen-III oxidase, mitochondrial (Fragment	OPHHA	2
1682	6.03	6.03	47.91	9.424	9.424	tr|V8PBB2|V8PBB2_OPHHA	Quinone oxidoreductase (Fragment) OS=Ophiophagus hannah	OPHHA	3
1683	6.03	6.03	59.73	30.87	30.87	tr|V8NW79|V8NW79_OPHHA	Golgi-associated plant pathogenesis-related protein 1 (	OPHHA	3
1684	6.03	6.03	51	37	37	tr|V8NNB6|V8NNB6_OPHHA	MICOS complex subunit MIC13 OS=Ophiophagus hannah GN=qi	OPHHA	3
1685	6.02	6.25	72	44.8	36	tr|V8NNX2|V8NNX2_OPHHA	Arginyl-tRNA synthetase, cytoplasmic (Fragment) OS=Ophi	OPHHA	3
1686	6.02	6.03	64.81	17.41	17.41	tr|V8P3K8|V8P3K8_OPHHA	Integrin alpha-X (Fragment) OS=Ophiophagus hannah GN=IT	OPHHA	4
1687	6.02	6.02	47.12	12.5	10.19	tr|V8P360|V8P360_OPHHA	Interferon regulatory factor 2-binding protein-like pro	OPHHA	3
1688	6.02	6.02	67.96	29.28	29.28	tr|V8NS34|V8NS34_OPHHA	Uncharacterized protein (Fragment) OS=Ophiophagus hanna	OPHHA	4
1689	6.01	6.01	42.23	16.22	16.22	tr|V8NT58|V8NT58_OPHHA	Integral membrane protein 2A (Fragment) OS=Ophiophagus	OPHHA	3
1690	6.01	6.01	27.1	15.89	15.89	tr|V8NMX5|V8NMX5_OPHHA	Acidic leucine-rich nuclear phosphoprotein 32 family me	OPHHA	3
1691	6	6.04	32.92	15	15	tr|V8NXM1|V8NXM1_OPHHA	MOB kinase activator 1B (Fragment) OS=Ophiophagus hanna	OPHHA	3
1692	6	6	53.33	3.546	3.546	tr|V8NM86|V8NM86_OPHHA	Collagen alpha-1(III) chain OS=Ophiophagus hannah GN=CO	OPHHA	3
1693	6	6	47.06	10.59	10.59	tr|V8NGH9|V8NGH9_OPHHA	PDZ and LIM domain protein 2 OS=Ophiophagus hannah GN=P	OPHHA	3
1694	6	6	52	18	18	tr|V8PFC0|V8PFC0_OPHHA	Prefoldin subunit 4 (Fragment) OS=Ophiophagus hannah GN	OPHHA	4
1695	6	6	26.61	7.752	7.752	tr|V8P5D4|V8P5D4_OPHHA	Monoacylglycerol lipase ABHD6 (Fragment) OS=Ophiophagus	OPHHA	4
1696	6	6	66.2	35.92	35.92	tr|V8P037|V8P037_OPHHA	DNA-directed RNA polymerase II subunit RPB4 OS=Ophiopha	OPHHA	3
1697	6	6	56.86	20.59	20.59	tr|V8N8F7|V8N8F7_OPHHA	Bifunctional ATP-dependent dihydroxyacetone kinase/FAD-	OPHHA	3
1698	6	6	40.11	19.79	19.79	tr|V8N9W6|V8N9W6_OPHHA	Glycine N-methyltransferase (Fragment) OS=Ophiophagus h	OPHHA	4
1699	6	6	60.47	27.91	27.91	tr|V8NJP1|V8NJP1_OPHHA	Galectin OS=Ophiophagus hannah GN=L345_11938 PE=4 SV=1	OPHHA	6
1700	6	6	34.93	26.03	26.03	tr|V8P5H9|V8P5H9_OPHHA	Cystatin-B OS=Ophiophagus hannah GN=CSTB PE=4 SV=1	OPHHA	4
1701	6	6	60.42	51.04	51.04	tr|V8NIV8|V8NIV8_OPHHA	Dynein light chain roadblock-type 2 OS=Ophiophagus hann	OPHHA	5
1702	5.96	5.96	81.3	15.27	15.27	tr|V8NYC5|V8NYC5_OPHHA	Spindlin-Z (Fragment) OS=Ophiophagus hannah GN=SPINZ PE	OPHHA	3
1703	5.96	5.96	78.85	18.06	18.06	tr|V8NZW8|V8NZW8_OPHHA	Charged multivesicular body protein 4b OS=Ophiophagus h	OPHHA	3
1704	5.95	6.13	29.03	5.963	3.91	tr|V8NTN1|V8NTN1_OPHHA	Nuclear factor of activated T-cells, cytoplasmic 3 (Fra	OPHHA	3
1705	5.95	6.06	36.8	8.696	4.242	tr|V8NRY4|V8NRY4_OPHHA	Solute carrier family 12 member 4 OS=Ophiophagus hannah	OPHHA	3
1706	5.95	5.95	61.95	21.95	18.54	tr|V8NC54|V8NC54_OPHHA	Biliverdin reductase A (Fragment) OS=Ophiophagus hannah	OPHHA	3
1707	5.94	5.94	39.86	4.447	2.921	tr|V8NZY5|V8NZY5_OPHHA	F-box/LRR-repeat protein 3 (Fragment) OS=Ophiophagus ha	OPHHA	6
1708	5.94	5.94	43.42	5.156	2.307	tr|V8PAQ4|V8PAQ4_OPHHA	Putative global transcription activator SNF2L2 (Fragmen	OPHHA	2
1709	5.94	5.94	34.59	9.412	7.529	tr|V8P7Y1|V8P7Y1_OPHHA	Lysocardiolipin acyltransferase 1 OS=Ophiophagus hannah	OPHHA	3
1710	5.93	5.93	27.03	22.07	22.07	tr|V8P386|V8P386_OPHHA	Complement component 1 Q subcomponent-binding protein,	OPHHA	5
1711	5.93	5.93	69.23	21.54	21.54	tr|V8P7R5|V8P7R5_OPHHA	Septin-11 (Fragment) OS=Ophiophagus hannah GN=SEPT11 PE	OPHHA	3
1712	5.91	5.93	39.34	16.63	10.54	tr|V8PHL8|V8PHL8_OPHHA	Serum deprivation-response protein OS=Ophiophagus hanna	OPHHA	4
1713	5.91	5.91	52.84	8.295	7.22	tr|V8PDT9|V8PDT9_OPHHA	U4/U6 small nuclear ribonucleoprotein Prp3 (Fragment) O	OPHHA	4
1714	5.91	5.91	67.76	14.47	11.51	tr|V8P823|V8P823_OPHHA	Epithelial cell adhesion molecule OS=Ophiophagus hannah	OPHHA	3
1715	5.91	5.91	50	40.28	30.56	tr|V8NF61|V8NF61_OPHHA	60S ribosomal protein L38 (Fragment) OS=Ophiophagus han	OPHHA	3
1716	5.9	5.9	38.9	15.85	15.85	tr|V8N4C0|V8N4C0_OPHHA	Cytochrome protein (Fragment) OS=Ophiophagus hannah GN=	OPHHA	3
1717	5.89	5.89	33.33	17.62	17.62	tr|V8P6T5|V8P6T5_OPHHA	Syntaxin-12 OS=Ophiophagus hannah GN=STX12 PE=4 SV=1	OPHHA	3

1718	5.88	5.88	25.97	7.759	4.31	tr|V8NWL2|V8NWL2_OPHHA	Alpha-mannosidase (Fragment) OS=Ophiophagus hannah GN=M	OPHHA	3
1719	5.88	5.88	51.08	13.85	13.85	tr|V8NYT0|V8NYT0_OPHHA	Protein CWC15-like protein OS=Ophiophagus hannah GN=CWC	OPHHA	4
1720	5.86	5.86	41.24	6.768	4.618	tr|V8P7D8|V8P7D8_OPHHA	Ankyrin repeat domain-containing protein 13D OS=Ophioph	OPHHA	4
1721	5.86	5.86	55.74	7.916	5.008	tr|V8NFA2|V8NFA2_OPHHA	Uncharacterized protein (Fragment) OS=Ophiophagus hanna	OPHHA	3
1722	5.86	5.86	60.73	7.287	7.287	tr|V8P4C6|V8P4C6_OPHHA	40S ribosomal protein S18 (Fragment) OS=Ophiophagus han	OPHHA	3
1723	5.85	5.86	62.41	25.31	8.772	tr|V8NI48|V8NI48_OPHHA	Mitogen-activated protein kinase 3 (Fragment) OS=Ophiop	OPHHA	3
1724	5.85	5.85	57.92	9.836	3.825	tr|V8PI77|V8PI77_OPHHA	Huntingtin-interacting protein 1 OS=Ophiophagus hannah	OPHHA	3
1725	5.85	5.85	60.28	20.09	9.112	tr|V8NWT9|V8NWT9_OPHHA	AP-3 complex subunit mu-1 (Fragment) OS=Ophiophagus han	OPHHA	3
1726	5.85	5.85	51.76	24.12	19.41	tr|V8NKK3|V8NKK3_OPHHA	60S ribosomal protein L14 (Fragment) OS=Ophiophagus han	OPHHA	3
1727	5.85	5.85	26.12	5.683	5.683	tr|V8NZM7|V8NZM7_OPHHA	Cadherin-2 (Fragment) OS=Ophiophagus hannah GN=CDH2 PE=	OPHHA	3
1728	5.84	5.85	44.4	9.483	9.052	tr|V8N7T6|V8N7T6_OPHHA	Uncharacterized protein (Fragment) OS=Ophiophagus hanna	OPHHA	3
1729	5.84	5.84	41.53	7.023	5.344	tr|V8P7T3|V8P7T3_OPHHA	Coagulation factor XIII B chain OS=Ophiophagus hannah G	OPHHA	3
1730	5.84	5.84	57.94	11.18	11.18	tr|V8NYF8|V8NYF8_OPHHA	Nuclear receptor coactivator 5 OS=Ophiophagus hannah GN	OPHHA	3
1731	5.83	5.83	55.1	11.89	11.89	tr|V8P235|V8P235_OPHHA	Methylmalonic aciduria type A protein, mitochondrial (F	OPHHA	3
1732	5.82	5.82	28.47	11.31	11.31	tr|V8N688|V8N688_OPHHA	ATP-binding cassette sub-family A member 8-B (Fragment)	OPHHA	3
1733	5.82	5.82	39.79	18.85	16.23	tr|V8N6N1|V8N6N1_OPHHA	Transmembrane protein 33 OS=Ophiophagus hannah GN=TMEM3	OPHHA	3
1734	5.82	5.82	89.83	72.88	72.88	tr|V8N290|V8N290_OPHHA	Uncharacterized protein (Fragment) OS=Ophiophagus hanna	OPHHA	3
1735	5.81	5.83	59.62	13.53	6.977	tr|V8P9M4|V8P9M4_OPHHA	Calcium-binding mitochondrial carrier protein SCaMC-3 (	OPHHA	3
1736	5.81	5.81	38.19	9.055	9.055	tr|V8P9T6|V8P9T6_OPHHA	alpha-1,2-Mannosidase (Fragment) OS=Ophiophagus hannah	OPHHA	4
1737	5.8	18.58	66.37	23.19	17.17	tr|V8PGM8|V8PGM8_OPHHA	Putative ATP-dependent RNA helicase DDX5 (Fragment) OS=	OPHHA	10
1738	5.8	5.8	48.99	5.325	4.473	tr|V8P5A4|V8P5A4_OPHHA	TBC1 domain family member 8B (Fragment) OS=Ophiophagus	OPHHA	4
1739	5.8	5.8	55.21	9.407	9.407	tr|V8P9S2|V8P9S2_OPHHA	Sterol 26-hydroxylase, mitochondrial (Fragment) OS=Ophi	OPHHA	4
1740	5.78	5.86	32.56	3.206	2.828	tr|V8NBL3|V8NBL3_OPHHA	Neurobeachin-like protein 1 (Fragment) OS=Ophiophagus h	OPHHA	3
1741	5.78	5.78	37.88	9.957	8.442	tr|V8NQ58|V8NQ58_OPHHA	Dihydropyrimidinase-related protein 2 (Fragment) OS=Oph	OPHHA	3
1742	5.77	5.77	53.47	17.55	8.98	tr|V8NFB1|V8NFB1_OPHHA	H/ACA ribonucleoprotein complex subunit 4 OS=Ophiophagu	OPHHA	3
1743	5.76	5.78	43.51	4.753	2.438	tr|V8P851|V8P851_OPHHA	General transcription factor II-I (Fragment) OS=Ophioph	OPHHA	3
1744	5.76	5.76	37.71	9.482	3.197	tr|V8NGX1|V8NGX1_OPHHA	Nuclear factor NF-kappa-B subunit (Fragment) OS=Ophioph	OPHHA	3
1745	5.74	5.74	36.23	4.307	3.632	tr|V8NF64|V8NF64_OPHHA	Cleavage and polyadenylation specificity factor subunit	OPHHA	3
1746	5.74	5.74	35.79	8.26	4.506	tr|V8NM19|V8NM19_OPHHA	La-related protein 4 (Fragment) OS=Ophiophagus hannah G	OPHHA	3
1747	5.74	5.74	33.78	6.634	6.634	tr|V8P246|V8P246_OPHHA	Mitogen-activated protein kinase kinase kinase MLT (Fra	OPHHA	4
1748	5.73	8.34	51.56	25	18.49	tr|V8P7Y0|V8P7Y0_OPHHA	DnaJ-like subfamily B member 4 (Fragment) OS=Ophiophagu	OPHHA	5
1749	5.73	6.91	67.46	28.99	28.99	tr|V8NAF2|V8NAF2_OPHHA	ADP-ribosylation factor 6 (Fragment) OS=Ophiophagus han	OPHHA	4
1750	5.73	5.73	64.69	6.755	3.787	tr|V8P6G6|V8P6G6_OPHHA	Putative global transcription activator SNF2L1 (Fragmen	OPHHA	3
1751	5.73	5.73	78.7	16.2	16.2	tr|V8P3P3|V8P3P3_OPHHA	Bcl-2-likeous antagonist/killer (Fragment) OS=Ophiophag	OPHHA	3
1752	5.72	5.72	59.93	6.568	5.255	tr|V8NTY9|V8NTY9_OPHHA	Cdc42-interacting protein 4 (Fragment) OS=Ophiophagus h	OPHHA	3
1753	5.72	5.72	37.53	7.087	7.087	tr|V8PDH1|V8PDH1_OPHHA	Neural cell adhesion molecule 1 (Fragment) OS=Ophiophag	OPHHA	3
1754	5.71	5.71	67.62	30.48	20	tr|V8PEY7|V8PEY7_OPHHA	60S ribosomal protein L36 OS=Ophiophagus hannah GN=RPL3	OPHHA	3
1755	5.71	5.71	58.96	19.08	19.08	tr|V8PC47|V8PC47_OPHHA	Enoyl-CoA delta isomerase 1, mitochondrial OS=Ophiophag	OPHHA	3
1756	5.7	8.02	63.23	7.794	5.159	tr|V8NW57|V8NW57_OPHHA	E3 ubiquitin-protein ligase BRE1A OS=Ophiophagus hannah	OPHHA	4
1757	5.69	5.69	41.92	20.82	18.08	tr|V8P5M6|V8P5M6_OPHHA	Regulator of chromosome condensation OS=Ophiophagus han	OPHHA	3
1758	5.68	5.72	65.83	21.67	21.67	tr|V8N543|V8N543_OPHHA	Mitochondrial import inner membrane translocase subunit	OPHHA	3
1759	5.68	5.68	71.29	23.27	23.27	tr|V8N486|V8N486_OPHHA	Uncharacterized protein (Fragment) OS=Ophiophagus hanna	OPHHA	3
1760	5.68	5.68	30.07	14.05	11.11	tr|V8NG96|V8NG96_OPHHA	Magnesium transporter protein 1 (Fragment) OS=Ophiophag	OPHHA	4
1761	5.67	7.57	74.74	35.26	26.32	tr|V8P0S7|V8P0S7_OPHHA	Hippocalcin-like protein 1 (Fragment) OS=Ophiophagus ha	OPHHA	4
1762	5.66	6.07	68.57	15.36	11.79	tr|V8NP56|V8NP56_OPHHA	Rho-associated protein kinase 1 (Fragment) OS=Ophiophag	OPHHA	3
1763	5.66	5.72	35.71	9.857	5.429	tr|V8NR27|V8NR27_OPHHA	Nuclear pore complex protein Nup88 (Fragment) OS=Ophiop	OPHHA	3
1764	5.66	5.66	71.32	34.88	34.88	tr|V8PG53|V8PG53_OPHHA	Thioredoxin domain-containing protein 12 OS=Ophiophagus	OPHHA	4
1765	5.65	5.95	62.71	31.07	26.55	tr|V8N4N7|V8N4N7_OPHHA	EH domain-containing protein 2 (Fragment) OS=Ophiophagu	OPHHA	4
1766	5.65	5.65	62.91	20.42	10.56	tr|V8P1K2|V8P1K2_OPHHA	Ras GTPase-activating protein-binding protein 1 (Fragme	OPHHA	3
1767	5.64	5.7	60.46	7.143	7.143	tr|V8P3S6|V8P3S6_OPHHA	mRNA cap guanine-N7 methyltransferase OS=Ophiophagus ha	OPHHA	3
1768	5.63	5.63	50.73	16.13	10.85	tr|V8PAA0|V8PAA0_OPHHA	Myelin expression factor 2 OS=Ophiophagus hannah GN=MYE	OPHHA	3
1769	5.63	5.63	77.14	26.19	18.1	tr|V8P0I0|V8P0I0_OPHHA	Golgi SNAP receptor complex member 2 (Fragment) OS=Ophi	OPHHA	3
1770	5.63	5.63	46.62	14.47	14.47	tr|V8PFY0|V8PFY0_OPHHA	Aspartoacylase-2 (Fragment) OS=Ophiophagus hannah GN=AC	OPHHA	4
1771	5.62	5.62	44.4	16.4	16.4	tr|V8NKL8|V8NKL8_OPHHA	Ras-associated and pleckstrin-likey domains-containing	OPHHA	3
1772	5.61	5.61	30.19	5.594	2.529	tr|V8P9T7|V8P9T7_OPHHA	Bromodomain-containing protein 4 (Fragment) OS=Ophiopha	OPHHA	3
1773	5.61	5.61	37.37	14.25	9.287	tr|V8NRG8|V8NRG8_OPHHA	Putative RNA-binding protein 23 (Fragment) OS=Ophiophag	OPHHA	3
1774	5.61	5.61	58.15	26.63	20.11	tr|V8N7Z7|V8N7Z7_OPHHA	U4/U6.U5 tri-snRNP-associated protein 1 (Fragment) OS=O	OPHHA	3
1775	5.6	6.37	41.62	4.275	4.275	tr|V8P836|V8P836_OPHHA	Collagen alpha-6(IV) chain OS=Ophiophagus hannah GN=COL	OPHHA	5
1776	5.59	5.59	37.24	4.368	2.805	tr|V8NS16|V8NS16_OPHHA	Transporter (Fragment) OS=Ophiophagus hannah GN=Usp19 P	OPHHA	4
1777	5.59	5.59	56.36	7.839	3.919	tr|V8P0D6|V8P0D6_OPHHA	Putative ATP-dependent RNA helicase DHX36 (Fragment) OS	OPHHA	3
1778	5.58	5.6	51.12	14.51	9.821	tr|V8PI13|V8PI13_OPHHA	Growth arrest-specific protein 7 (Fragment) OS=Ophiopha	OPHHA	3
1779	5.57	5.69	50.35	2.481	2.149	tr|V8NGL1|V8NGL1_OPHHA	Nesprin-2 (Fragment) OS=Ophiophagus hannah GN=SYNE2 PE=	OPHHA	8
1780	5.57	5.57	63.68	29.85	17.41	tr|V8PAC8|V8PAC8_OPHHA	Nuclear factor 1 C-type (Fragment) OS=Ophiophagus hanna	OPHHA	3
1781	5.56	5.56	41.18	10.87	7.487	tr|V8NX47|V8NX47_OPHHA	Spartin OS=Ophiophagus hannah GN=SPG20 PE=4 SV=1	OPHHA	3
1782	5.56	5.56	32.62	22.32	12.45	tr|V8P3L9|V8P3L9_OPHHA	Autophagy protein 5 OS=Ophiophagus hannah GN=ATG5 PE=3	OPHHA	2
1783	5.55	5.55	48.67	25.33	14	tr|V8P9Z0|V8P9Z0_OPHHA	Mitochondrial ornithine transporter 1 (Fragment) OS=Oph	OPHHA	4
1784	5.54	7.7	41.17	15.53	10.87	tr|V8NU83|V8NU83_OPHHA	Signal transducing adapter molecule 2 OS=Ophiophagus ha	OPHHA	4
1785	5.52	5.53	31.1	4.146	4.024	tr|V8ND08|V8ND08_OPHHA	Cytochrome c1, heme protein, mitochondrial OS=Ophiophag	OPHHA	4
1786	5.52	5.52	37.67	5.285	5.285	tr|V8PCH2|V8PCH2_OPHHA	Amyloid beta A4 protein (Fragment) OS=Ophiophagus hanna	OPHHA	3
1787	5.52	5.52	41.46	6.402	6.402	tr|V8NY28|V8NY28_OPHHA	Protein PAT1-like 2 (Fragment) OS=Ophiophagus hannah GN	OPHHA	4
1788	5.52	5.52	60.47	22.09	22.09	tr|V8P1S7|V8P1S7_OPHHA	Ras-related protein R-Ras2 (Fragment) OS=Ophiophagus ha	OPHHA	3
1789	5.52	5.52	49.72	18.64	18.64	tr|V8N9G7|V8N9G7_OPHHA	39S ribosomal protein L32, mitochondrial OS=Ophiophagus	OPHHA	4
1790	5.51	7.84	49.09	32.73	24.09	tr|V8P5V2|V8P5V2_OPHHA	Ras-related protein Rab-3D OS=Ophiophagus hannah GN=RAB	OPHHA	4
1791	5.51	5.52	50	7.244	5.966	tr|V8NFY5|V8NFY5_OPHHA	Cleavage stimulation factor subunit 2 tau variant (Frag	OPHHA	3
1792	5.51	5.51	40	4.72	4.72	tr|V8NQB8|V8NQB8_OPHHA	Tetratricopeptide repeat protein 13 (Fragment) OS=Ophio	OPHHA	3
1793	5.51	5.51	34.58	18.75	18.75	tr|V8P494|V8P494_OPHHA	Eukaryotic translation initiation factor 6 (Fragment) O	OPHHA	3
1794	5.5	8.3	58.07	23.29	13.66	tr|V8PEZ8|V8PEZ8_OPHHA	Serine/arginine-rich splicing factor 5 (Fragment) OS=Op	OPHHA	4
1795	5.5	5.5	46.83	4.736	3.514	tr|V8PCW9|V8PCW9_OPHHA	Tetratricopeptide repeat protein 37 (Fragment) OS=Ophio	OPHHA	3
1796	5.5	5.5	19.5	8.714	7.261	tr|V8P485|V8P485_OPHHA	Epoxide hydrolase 2 (Fragment) OS=Ophiophagus hannah GN	OPHHA	3
1797	5.49	5.49	40.53	7.84	6.509	tr|V8NGE1|V8NGE1_OPHHA	Putative GTP-binding protein Parf (Fragment) OS=Ophioph	OPHHA	3
1798	5.49	5.49	52.29	12	12	tr|V8NVQ0|V8NVQ0_OPHHA	rRNA adenine N(6)-methyltransferase (Fragment) OS=Ophio	OPHHA	3
1799	5.48	6.93	57.22	20	6.296	tr|V8P330|V8P330_OPHHA	Ribosomal protein S6 kinase alpha-3 (Fragment) OS=Ophio	OPHHA	3
1800	5.48	5.48	55.27	9.283	7.595	tr|V8P3W8|V8P3W8_OPHHA	Rab GTPase-binding effector protein 2 (Fragment) OS=Oph	OPHHA	3
1801	5.48	5.48	51.45	12.5	9.884	tr|V8NS67|V8NS67_OPHHA	Eukaryotic translation initiation factor 3 subunit G OS	OPHHA	3
1802	5.48	5.48	21.51	10.98	8.009	tr|V8PG58|V8PG58_OPHHA	Interferon-related developmental regulator 2 OS=Ophioph	OPHHA	3
1803	5.47	5.48	33.55	7.947	7.947	tr|V8NQU5|V8NQU5_OPHHA	WD repeat-containing protein 37 (Fragment) OS=Ophiophag	OPHHA	3
1804	5.46	6.38	50.82	25.14	19.67	tr|V8P4K0|V8P4K0_OPHHA	Protein lin-7-like A (Fragment) OS=Ophiophagus hannah G	OPHHA	4
1805	5.46	5.46	34.47	2.872	1.996	tr|V8P498|V8P498_OPHHA	Autophagy-related protein 2-like B (Fragment) OS=Ophiop	OPHHA	3
1806	5.46	5.46	52.7	10.64	6.383	tr|V8NC04|V8NC04_OPHHA	ATP-binding cassette sub-family B member 8, mitochondri	OPHHA	3
1807	5.46	5.46	75.4	15.08	8.73	tr|V8ND26|V8ND26_OPHHA	Prefoldin subunit 6 OS=Ophiophagus hannah GN=PFDN6 PE=4	OPHHA	3
1808	5.45	5.46	54.57	13.85	11.36	tr|V8PF59|V8PF59_OPHHA	Centromere/kinetochore protein zw10-like protein (Fragm	OPHHA	3
1809	5.45	5.45	49.81	14.37	9.195	tr|V8P5C1|V8P5C1_OPHHA	Alpha-1,3-mannosyl-glycoprotein 4-beta-N-acetylglucosam	OPHHA	3
1810	5.45	5.45	46.21	23.48	16.67	tr|V8P395|V8P395_OPHHA	Glutathione peroxidase (Fragment) OS=Ophiophagus hannah	OPHHA	3
1811	5.44	6.65	25.17	11.13	8.733	tr|V8P0F7|V8P0F7_OPHHA	Transketolase-like protein 2 (Fragment) OS=Ophiophagus	OPHHA	5
1812	5.43	5.43	44.21	14.88	14.46	tr|V8N837|V8N837_OPHHA	Dual specificity mitogen-activated protein kinase 1 (Fr	OPHHA	3
1813	5.42	5.42	35.43	16.27	11.02	tr|V8PGK8|V8PGK8_OPHHA	Thromboxane-A synthase (Fragment) OS=Ophiophagus hannah	OPHHA	3

1814	5.42	5.42	50.44	31.86	31.86	tr|V8N3L4|V8N3L4_OPHHA	Complement C3 (Fragment) OS=Ophiophagus hannah GN=C3 PE	OPHHA	3
1815	5.41	13.83	54.43	36.29	33.33	tr|V8P6N2|V8P6N2_OPHHA	Ribose-phosphate pyrophosphokinase 1 (Fragment) OS=Ophi	OPHHA	11
1816	5.41	5.41	61.44	26.8	26.8	tr|V8NMY4|V8NMY4_OPHHA	Ubiquitin carboxyl-terminal hydrolase isozyme L5 (Fragm	OPHHA	3
1817	5.4	10.3	80.29	32.21	22.6	tr|V8NQ74|V8NQ74_OPHHA	GTPase KRas OS=Ophiophagus hannah GN=KRAS PE=4 SV=1	OPHHA	5
1817	0.01	11.77	68.66	29.85	24.38	tr|V8NZX8|V8NZX8_OPHHA	GTPase NRas (Fragment) OS=Ophiophagus hannah GN=NRAS PE	OPHHA	6
1818	5.4	7.51	22.8	9.985	8.495	tr|V8NUP4|V8NUP4_OPHHA	Transmembrane 9 superfamily member 2 (Fragment) OS=Ophi	OPHHA	5
1819	5.4	5.41	54	8.571	5.429	tr|V8P3C4|V8P3C4_OPHHA	Calpastatin (Fragment) OS=Ophiophagus hannah GN=CAST PE	OPHHA	3
1820	5.4	5.4	49.42	5.922	2.427	tr|V8NP52|V8NP52_OPHHA	Serine/threonine-protein kinase PRP4-like protein (Frag	OPHHA	2
1821	5.39	5.39	43.01	10.48	10.48	tr|V8NZE5|V8NZE5_OPHHA	Protein O-glucosyltransferase 1 (Fragment) OS=Ophiophag	OPHHA	3
1822	5.39	5.39	48.65	11.58	8.88	tr|V8NYF4|V8NYF4_OPHHA	Acylpyruvase FAHD1, mitochondrial (Fragment) OS=Ophioph	OPHHA	3
1823	5.38	5.38	49.21	13.1	7.341	tr|V8NCY4|V8NCY4_OPHHA	Eukaryotic translation initiation factor 4 gamma 3 (Fra	OPHHA	2
1824	5.38	5.38	46.94	23.81	17.69	tr|V8PEK8|V8PEK8_OPHHA	Ribokinase (Fragment) OS=Ophiophagus hannah GN=RBKS PE=	OPHHA	3
1825	5.38	5.38	29.19	17.84	17.3	tr|V8P6T0|V8P6T0_OPHHA	15 kDa selenoprotein (Fragment) OS=Ophiophagus hannah G	OPHHA	3
1826	5.38	5.38	36.13	26.89	26.89	tr|V8N418|V8N418_OPHHA	Beta-arrestin-2 (Fragment) OS=Ophiophagus hannah GN=ARR	OPHHA	3
1827	5.37	5.37	82.64	38.89	25.69	tr|V8N7H8|V8N7H8_OPHHA	SLIT-ROBO Rho GTPase-activating protein 2 (Fragment) OS	OPHHA	3
1828	5.36	5.76	55.12	9.537	4.488	tr|V8PA50|V8PA50_OPHHA	Zinc phosphodiesterase ELAC protein 2 (Fragment) OS=Oph	OPHHA	2
1829	5.36	5.36	31.55	4.729	2.676	tr|V8NLR3|V8NLR3_OPHHA	Exportin-T (Fragment) OS=Ophiophagus hannah GN=XPOT PE=	OPHHA	3
1830	5.36	5.36	36.77	4.626	4.626	tr|V8PIS5|V8PIS5_OPHHA	Transforming growth factor-beta receptor-associated pro	OPHHA	3
1831	5.36	5.36	60.66	23.77	14.75	tr|V8NME7|V8NME7_OPHHA	Chloride intracellular channel protein 4 (Fragment) OS=	OPHHA	4
1832	5.36	5.36	38.83	35.92	34.95	tr|V8NQN5|V8NQN5_OPHHA	LIM and senescent cell antigen-like-containing domain p	OPHHA	3
1833	5.35	5.35	54.06	9.219	3.594	tr|V8NQC4|V8NQC4_OPHHA	Pre-mRNA-processing factor 39 OS=Ophiophagus hannah GN=	OPHHA	2
1834	5.35	5.35	46.85	12.59	10.14	tr|V8P2L6|V8P2L6_OPHHA	Protein FAM54B OS=Ophiophagus hannah GN=FAM54B PE=4 SV=	OPHHA	3
1835	5.34	5.34	49.61	9.424	5.759	tr|V8P918|V8P918_OPHHA	Phospholipase A-2-activating protein (Fragment) OS=Ophi	OPHHA	3
1836	5.34	5.34	51.67	11.94	11.67	tr|V8NHU0|V8NHU0_OPHHA	Alpha-methylacyl-CoA racemase (Fragment) OS=Ophiophagus	OPHHA	4
1837	5.34	5.34	60.61	20.35	16.45	tr|V8NJ71|V8NJ71_OPHHA	Proline synthase co-transcribed bacterial-like protein	OPHHA	3
1838	5.32	5.32	62.05	7.59	5.326	tr|V8PA80|V8PA80_OPHHA	Pre-mRNA-splicing factor CWC25-like protein OS=Ophiopha	OPHHA	3
1839	5.32	5.32	38.92	7.703	6.486	tr|V8NJ63|V8NJ63_OPHHA	Signal transducer and activator of transcription OS=Oph	OPHHA	4
1840	5.32	5.32	26.17	7.799	5.026	tr|V8NA49|V8NA49_OPHHA	Thioredoxin reductase 3 (Fragment) OS=Ophiophagus hanna	OPHHA	3
1841	5.3	5.3	46.09	2.02	2.02	tr|V8NTJ8|V8NTJ8_OPHHA	Dedicator of cytokinesis protein 9 (Fragment) OS=Ophiop	OPHHA	3
1842	5.3	5.3	58.51	16.49	10.11	tr|V8PBF2|V8PBF2_OPHHA	Guanine nucleotide-binding protein subunit alpha-13 (Fr	OPHHA	3
1843	5.3	5.3	58.78	36.64	29.01	tr|V8PGJ8|V8PGJ8_OPHHA	Protein phosphatase 1 regulatory subunit 21 OS=Ophiopha	OPHHA	3
1844	5.29	5.31	27.1	5.048	2.673	tr|V8NXI8|V8NXI8_OPHHA	Rab3 GTPase-activating protein non-catalytic subunit OS	OPHHA	3
1845	5.29	5.29	20.92	7.609	7.609	tr|V8P1T2|V8P1T2_OPHHA	ABI family member 3 OS=Ophiophagus hannah GN=ABI3 PE=4	OPHHA	3
1846	5.28	5.85	61.5	17.84	14.08	tr|V8N6W1|V8N6W1_OPHHA	Retinol dehydrogenase 16 (Fragment) OS=Ophiophagus hann	OPHHA	4
1847	5.28	5.28	39.61	10.96	6.18	tr|V8NYA8|V8NYA8_OPHHA	MOSC domain-containing protein 2, mitochondrial (Fragme	OPHHA	2
1848	5.28	5.28	43.92	8.685	8.685	tr|V8P0D2|V8P0D2_OPHHA	RNA 3'-terminal phosphate cyclase (Fragment) OS=Ophioph	OPHHA	3
1849	5.27	5.27	49.64	6.569	6.569	tr|V8NX22|V8NX22_OPHHA	Putative ATP-dependent RNA helicase DHX58 (Fragment) OS	OPHHA	3
1850	5.27	5.27	64.44	26.67	21.48	tr|V8NDW1|V8NDW1_OPHHA	GDP-L-fucose synthase (Fragment) OS=Ophiophagus hannah	OPHHA	3
1851	5.27	5.27	13.01	10.5	8.447	tr|V8NU92|V8NU92_OPHHA	Fibulin-5 (Fragment) OS=Ophiophagus hannah GN=FBLN5 PE=	OPHHA	3
1852	5.26	5.32	35.35	5.537	3.918	tr|V8NNL8|V8NNL8_OPHHA	TBC1 domain family member 8 (Fragment) OS=Ophiophagus h	OPHHA	3
1853	5.26	5.26	46.84	11.14	9.367	tr|V8N908|V8N908_OPHHA	Sorting nexin-8 (Fragment) OS=Ophiophagus hannah GN=SNX	OPHHA	3
1854	5.26	5.26	66.67	27.38	22.02	tr|V8P0P0|V8P0P0_OPHHA	Stathmin (Fragment) OS=Ophiophagus hannah GN=STMN1 PE=3	OPHHA	4
1855	5.25	5.25	32.92	7.335	5.635	tr|V8P8V9|V8P8V9_OPHHA	Histone deacetylase 6 (Fragment) OS=Ophiophagus hannah	OPHHA	4
1856	5.25	5.25	69.23	41.03	41.03	tr|V8P0X3|V8P0X3_OPHHA	Succinyl-CoA ligase GDP-forming subunit beta (Fragment)	OPHHA	4
1857	5.24	5.24	27.54	22.16	15.57	tr|V8P9M8|V8P9M8_OPHHA	Translocon-associated protein subunit delta OS=Ophiopha	OPHHA	5
1858	5.23	5.28	26.17	4.95	2.013	tr|V8P1P1|V8P1P1_OPHHA	Macrophage-stimulating protein receptor (Fragment) OS=O	OPHHA	2
1859	5.23	5.23	59.85	20.2	10.1	tr|V8NRS0|V8NRS0_OPHHA	Pre-mRNA-splicing regulator WTAP OS=Ophiophagus hannah	OPHHA	3
1860	5.23	5.23	26.38	4.341	4.341	tr|V8ND78|V8ND78_OPHHA	Basement membrane-specific heparan sulfate proteoglycan	OPHHA	4
1861	5.22	5.22	29.64	7.972	5.893	tr|V8PCJ8|V8PCJ8_OPHHA	GPI transamidase component PIG-T OS=Ophiophagus hannah	OPHHA	3
1862	5.21	5.21	28.26	8.203	8.073	tr|V8P228|V8P228_OPHHA	SH2B adapter protein 2 (Fragment) OS=Ophiophagus hannah	OPHHA	4
1863	5.21	5.21	48.95	12.94	11.89	tr|V8NKQ0|V8NKQ0_OPHHA	Microfibrillar-associated protein 1 (Fragment) OS=Ophio	OPHHA	3
1864	5.21	5.21	40.75	15.01	6.166	tr|V8NH50|V8NH50_OPHHA	Actin-binding LIM protein 1 (Fragment) OS=Ophiophagus h	OPHHA	2
1865	5.21	5.21	39.92	13.04	13.04	tr|V8NI61|V8NI61_OPHHA	Valacyclovir hydrolase (Fragment) OS=Ophiophagus hannah	OPHHA	3
1866	5.2	5.2	30.91	6.472	6.472	tr|V8P8B5|V8P8B5_OPHHA	Cadherin-13 (Fragment) OS=Ophiophagus hannah GN=CDH13 P	OPHHA	4
1867	5.2	5.2	45.53	18.7	18.7	tr|V8N726|V8N726_OPHHA	UBX domain-containing protein 1 (Fragment) OS=Ophiophag	OPHHA	3
1868	5.19	5.19	46.68	8.955	3.256	tr|V8NGY4|V8NGY4_OPHHA	Histidyl-tRNA synthetase, cytoplasmic OS=Ophiophagus ha	OPHHA	2
1869	5.18	5.18	49.51	15.93	7.598	tr|V8NPT7|V8NPT7_OPHHA	Cysteine protease ATG4B (Fragment) OS=Ophiophagus hanna	OPHHA	2
1870	5.17	5.17	40.82	11.59	7.005	tr|V8NYL0|V8NYL0_OPHHA	Lysophosphatidic acid phosphatase type 6 OS=Ophiophagus	OPHHA	3
1871	5.17	5.17	54.22	36.75	30.72	tr|V8NP04|V8NP04_OPHHA	Septin-2A (Fragment) OS=Ophiophagus hannah GN=sept2-A P	OPHHA	3
1872	5.16	10.32	47.67	11.37	11.37	tr|V8NS63|V8NS63_OPHHA	Long-chain-fatty-acid--CoA ligase 4 (Fragment) OS=Ophio	OPHHA	6
1873	5.16	5.16	48.07	10.83	5.138	tr|V8N7L5|V8N7L5_OPHHA	Metastasis-associated protein MTA2 (Fragment) OS=Ophiop	OPHHA	3
1874	5.15	5.15	48.04	2.399	1.897	tr|V8P2I9|V8P2I9_OPHHA	Microtubule-actin cross-linking factor 1 (Fragment) OS=	OPHHA	7
1875	5.15	5.15	68.17	11.41	5.255	tr|V8NF56|V8NF56_OPHHA	RNA polymerase II-associated protein 3 (Fragment) OS=Op	OPHHA	3
1876	5.15	5.15	52.17	16.43	9.179	tr|V8NXU7|V8NXU7_OPHHA	Myc-associated zinc finger protein (Fragment) OS=Ophiop	OPHHA	3
1877	5.15	5.15	47.35	9.412	9.412	tr|V8NLE1|V8NLE1_OPHHA	Cathepsin L1 OS=Ophiophagus hannah GN=CTSL1 PE=3 SV=1	OPHHA	4
1878	5.15	5.15	43.54	7.555	7.555	tr|V8N4X6|V8N4X6_OPHHA	ATP-binding cassette sub-family B member 9 (Fragment) O	OPHHA	3
1879	5.13	5.13	31.64	4.127	3.175	tr|V8NIQ5|V8NIQ5_OPHHA	Zinc finger and BTB domain-containing protein 2 (Fragme	OPHHA	3
1880	5.13	5.13	42.62	20.77	20.77	tr|V8PCW3|V8PCW3_OPHHA	Transmembrane protein 85 OS=Ophiophagus hannah GN=Tmem8	OPHHA	3
1881	5.12	5.13	51.32	16.12	14.14	tr|V8P5Y4|V8P5Y4_OPHHA	Small glutamine-rich tetratricopeptide repeat-containin	OPHHA	3
1882	5.12	5.12	53.6	14.86	14.86	tr|V8NTK8|V8NTK8_OPHHA	Histone H1x (Fragment) OS=Ophiophagus hannah GN=H1FX PE	OPHHA	3
1883	5.12	5.12	33.81	18.71	15.11	tr|V8NLS3|V8NLS3_OPHHA	Peroxisomal membrane protein PEX14 (Fragment) OS=Ophiop	OPHHA	3
1884	5.12	5.12	64.94	37.66	37.66	tr|V8N9B2|V8N9B2_OPHHA	Rab GDP dissociation inhibitor alpha (Fragment) OS=Ophi	OPHHA	3
1885	5.11	8.96	55.56	16.02	10.08	tr|V8PJF0|V8PJF0_OPHHA	Cytochrome protein (Fragment) OS=Ophiophagus hannah GN=	OPHHA	7
1886	5.11	5.11	28.73	3.861	2.008	tr|V8NSV2|V8NSV2_OPHHA	Disco-interacting protein 2-like B (Fragment) OS=Ophiop	OPHHA	2
1887	5.11	5.11	43.15	18.95	16.13	tr|V8PBS7|V8PBS7_OPHHA	ERI1 exoribonuclease 3 (Fragment) OS=Ophiophagus hannah	OPHHA	3
1888	5.1	5.1	40.52	28.45	28.45	tr|V8PHT6|V8PHT6_OPHHA	Ubiquitin-40S ribosomal protein S27a (Fragment) OS=Ophi	OPHHA	4
1889	5.1	5.1	27.84	15.46	15.46	tr|V8P6K8|V8P6K8_OPHHA	Corticosteroid 11-beta-dehydrogenase isozyme 1 (Fragmen	OPHHA	3
1890	5.09	5.09	75.15	30.3	30.3	tr|V8PH04|V8PH04_OPHHA	Cold-inducible RNA-binding protein (Fragment) OS=Ophiop	OPHHA	3
1891	5.09	5.09	61.31	20.44	20.44	tr|V8P254|V8P254_OPHHA	Osteoclast-stimulating factor 1 (Fragment) OS=Ophiophag	OPHHA	3
1892	5.08	5.08	76.92	16.78	13.29	tr|V8N8H1|V8N8H1_OPHHA	Protein RUFY3 (Fragment) OS=Ophiophagus hannah GN=RUFY3	OPHHA	3
1893	5.08	5.08	52.63	7.105	7.105	tr|V8PHX7|V8PHX7_OPHHA	Prothrombin (Fragment) OS=Ophiophagus hannah GN=F2 PE=3	OPHHA	4
1894	5.07	5.07	68.26	28.26	21.74	tr|V8P407|V8P407_OPHHA	Calcium-binding protein p22 (Fragment) OS=Ophiophagus h	OPHHA	4
1895	5.06	5.06	44.66	13.55	6.298	tr|V8PAD3|V8PAD3_OPHHA	Alpha-1-syntrophin (Fragment) OS=Ophiophagus hannah GN=	OPHHA	3
1896	5.06	5.06	52.51	20.95	10.06	tr|V8P9G2|V8P9G2_OPHHA	Adenylyltransferase and sulfurtransferase MOCS3 (Fragme	OPHHA	3
1897	5.06	5.06	80.49	42.68	42.68	tr|V8PDI9|V8PDI9_OPHHA	Acylphosphatase-2 OS=Ophiophagus hannah GN=ACYP2 PE=3 S	OPHHA	3
1898	5.06	5.06	65.33	52	52	tr|V8P5X1|V8P5X1_OPHHA	Homogentisate 1,2-dioxygenase (Fragment) OS=Ophiophagus	OPHHA	5
1899	5.05	5.56	32.28	3.465	1.337	tr|V8NLT5|V8NLT5_OPHHA	Threonyl-tRNA synthetase, cytoplasmic OS=Ophiophagus ha	OPHHA	2
1900	5.05	5.42	57.48	5.316	2.907	tr|V8PFU7|V8PFU7_OPHHA	Kinesin-like protein (Fragment) OS=Ophiophagus hannah G	OPHHA	3
1901	5.05	5.05	65.59	17.41	17.41	tr|V8PEP7|V8PEP7_OPHHA	WD repeat and FYVE domain-containing protein 2 (Fragmen	OPHHA	3
1902	5.05	5.05	30.73	21.95	21.95	tr|V8NN53|V8NN53_OPHHA	SPRY domain-containing protein 7 OS=Ophiophagus hannah	OPHHA	3
1903	5.04	5.05	52.17	3.814	3.038	tr|V8NZZ4|V8NZZ4_OPHHA	DNA2-like helicase (Fragment) OS=Ophiophagus hannah GN=	OPHHA	4
1904	5.03	5.89	49.73	12.12	9.626	tr|V8NGJ1|V8NGJ1_OPHHA	Mitogen-activated protein kinase (Fragment) OS=Ophiopha	OPHHA	4
1905	5.03	5.05	35.01	7.131	7.131	tr|V8NTA1|V8NTA1_OPHHA	Katanin p80 WD40-containing subunit B1 (Fragment) OS=Op	OPHHA	3
1906	5.03	5.03	50.99	7.649	7.649	tr|V8NW76|V8NW76_OPHHA	39S ribosomal protein L50, mitochondrial (Fragment) OS=	OPHHA	3
1907	5.03	5.03	73	34	25	tr|V8P9K0|V8P9K0_OPHHA	Uncharacterized protein OS=Ophiophagus hannah GN=L345_0	OPHHA	3
1908	5.02	5.02	54.05	5.263	5.263	tr|V8NRX0|V8NRX0_OPHHA	TRMT1-like protein (Fragment) OS=Ophiophagus hannah GN=	OPHHA	3

1909	5.01	5.08	64.04	30.34	15.36	tr|V8P9P3|V8P9P3_OPHHA	Protein mago nashi-like 2 OS=Ophiophagus hannah GN=MAGO	OPHHA	3
1910	5.01	5.01	51.3	7.818	4.886	tr|V8P3I8|V8P3I8_OPHHA	SH3 domain-containing kinase-binding protein 1 OS=Ophio	OPHHA	2
1911	5.01	5.01	70.45	22.16	21.59	tr|V8PAK0|V8PAK0_OPHHA	Signal recognition particle 19 kDa protein (Fragment) O	OPHHA	3
1912	5	5	56.49	22.08	22.08	tr|V8PF44|V8PF44_OPHHA	39S ribosomal protein L43, mitochondrial OS=Ophiophagus	OPHHA	3
1913	4.99	7.17	54.13	23.39	23.39	tr|V8NT43|V8NT43_OPHHA	Ras-related protein Rab-4A OS=Ophiophagus hannah GN=RAB	OPHHA	4
1914	4.99	4.99	39.4	2.715	1.293	tr|V8PBP0|V8PBP0_OPHHA	Vacuolar protein sorting-associated protein 13A (Fragme	OPHHA	3
1915	4.99	4.99	36.06	9.015	9.015	tr|V8P2T6|V8P2T6_OPHHA	Metalloreductase STEAP4 OS=Ophiophagus hannah GN=STEAP4	OPHHA	3
1916	4.98	4.98	44.27	13.44	13.44	tr|V8NBN0|V8NBN0_OPHHA	Endophilin-A2 (Fragment) OS=Ophiophagus hannah GN=SH3GL	OPHHA	4
1917	4.97	4.97	49.47	6.823	5.97	tr|V8NLY4|V8NLY4_OPHHA	COP9 signalosome complex subunit 3 OS=Ophiophagus hanna	OPHHA	4
1918	4.96	5.07	65.9	36.42	31.79	tr|V8P757|V8P757_OPHHA	Transcription factor BTF3 (Fragment) OS=Ophiophagus han	OPHHA	3
1919	4.95	4.95	71.19	23.73	23.73	tr|V8P615|V8P615_OPHHA	PEST proteolytic signal-containing nuclear protein OS=O	OPHHA	3
1920	4.95	4.95	53.55	29.35	9.355	tr|V8NBY7|V8NBY7_OPHHA	Retinol dehydrogenase 8 (Fragment) OS=Ophiophagus hanna	OPHHA	2
1921	4.95	4.95	28.87	12.37	12.37	tr|V8NS49|V8NS49_OPHHA	Dihydropyrimidine dehydrogenase [NADP+] (Fragment) OS=O	NADP+	3
1922	4.95	4.95	54.84	23.39	16.13	tr|V8P093|V8P093_OPHHA	Thioredoxin domain-containing protein 17 OS=Ophiophagus	OPHHA	2
1923	4.94	4.95	37.07	3.415	1.951	tr|V8PJ58|V8PJ58_OPHHA	Tubulin-specific chaperone D (Fragment) OS=Ophiophagus	OPHHA	2
1924	4.94	4.94	51.5	4.954	2.535	tr|V8PEY6|V8PEY6_OPHHA	Rab5 GDP/GTP exchange factor (Fragment) OS=Ophiophagus	OPHHA	2
1925	4.93	4.93	39.01	6.656	6.656	tr|V8NWF9|V8NWF9_OPHHA	Beta-xylosidase/alpha-L-arabinofuranosidase 1 (Fragment	OPHHA	3
1926	4.93	4.93	44.22	15.65	10.54	tr|V8NNN1|V8NNN1_OPHHA	Dehydrogenase/reductase SDR family member 1 (Fragment)	OPHHA	3
1927	4.92	4.92	64.71	16.04	16.04	tr|V8NUR5|V8NUR5_OPHHA	Actin-related protein 2/3 complex subunit 4 OS=Ophiopha	OPHHA	3
1928	4.91	4.91	63.67	16	11	tr|V8NYG8|V8NYG8_OPHHA	Sorting nexin-30 (Fragment) OS=Ophiophagus hannah GN=SN	OPHHA	3
1929	4.91	4.91	36.23	11.83	10.72	tr|V8PBJ2|V8PBJ2_OPHHA	Protein wntless-like protein (Fragment) OS=Ophiophagus	OPHHA	4
1930	4.91	4.91	43.15	8.333	8.333	tr|V8NDF4|V8NDF4_OPHHA	Apolipoprotein O (Fragment) OS=Ophiophagus hannah GN=AP	OPHHA	3
1931	4.9	4.9	49.13	10.26	3.902	tr|V8PAX8|V8PAX8_OPHHA	85 kDa calcium-independent phospholipase A2 (Fragment)	OPHHA	3
1932	4.9	4.9	41.56	9.494	8.017	tr|V8P7Z2|V8P7Z2_OPHHA	SWI/SNF-related matrix-associated actin-dependent regul	OPHHA	3
1933	4.9	4.9	70.24	37.5	17.86	tr|V8N4Z2|V8N4Z2_OPHHA	Uncharacterized protein (Fragment) OS=Ophiophagus hanna	OPHHA	2
1934	4.89	4.89	35.88	8.779	3.435	tr|V8P3J8|V8P3J8_OPHHA	E3 ubiquitin-protein ligase CBL (Fragment) OS=Ophiophag	OPHHA	2
1935	4.89	4.89	76.24	37.62	37.62	tr|V8NZK2|V8NZK2_OPHHA	Thioredoxin (Fragment) OS=Ophiophagus hannah GN=TXN PE=	OPHHA	4
1936	4.88	4.88	60.63	24.41	24.41	tr|V8NIG8|V8NIG8_OPHHA	Ragulator complex protein LAMTOR2 (Fragment) OS=Ophioph	OPHHA	3
1937	4.87	4.9	37.66	9.414	8.348	tr|V8P3C7|V8P3C7_OPHHA	UBX domain-containing protein 4 (Fragment) OS=Ophiophag	OPHHA	3
1938	4.87	4.87	25.94	7.509	7.509	tr|V8PG37|V8PG37_OPHHA	Putative proline racemase (Fragment) OS=Ophiophagus han	OPHHA	3
1939	4.87	4.87	20.42	11.11	9.61	tr|V8NXM7|V8NXM7_OPHHA	BCL2/adenovirus E1B 19 kDa protein-interacting protein	OPHHA	3
1940	4.86	4.86	51.98	17.15	12.4	tr|V8NF01|V8NF01_OPHHA	Uncharacterized protein (Fragment) OS=Ophiophagus hanna	OPHHA	3
1941	4.85	4.85	62.39	9.057	5.912	tr|V8NW99|V8NW99_OPHHA	RNA-binding protein 12B (Fragment) OS=Ophiophagus hanna	OPHHA	3
1942	4.85	4.85	55.67	4.418	4.418	tr|V8NA09|V8NA09_OPHHA	Uncharacterized protein (Fragment) OS=Ophiophagus hanna	OPHHA	3
1943	4.85	4.85	51.94	14.84	11.31	tr|V8NW44|V8NW44_OPHHA	Poly(ADP-ribose) glycohydrolase ARH3 (Fragment) OS=Ophi	OPHHA	3
1944	4.85	4.85	58.71	25.87	25.87	tr|V8NTU4|V8NTU4_OPHHA	JmjC domain-containing protein 7 (Fragment) OS=Ophiopha	OPHHA	3
1945	4.83	4.87	42.71	8.919	3.602	tr|V8NQB0|V8NQB0_OPHHA	Serine/threonine-protein phosphatase 2A 56 kDa regulato	OPHHA	2
1946	4.83	4.83	42.04	9.922	6.789	tr|V8NJ14|V8NJ14_OPHHA	Mothers against decapentaplegic homolog OS=Ophiophagus	OPHHA	3
1946	0	4.59	16.35	16.35	16.35	tr|V8NCH2|V8NCH2_OPHHA	Uncharacterized protein OS=Ophiophagus hannah GN=L345 1	OPHHA	3
1947	4.83	4.83	74.7	28.31	28.31	tr|V8N9P1|V8N9P1_OPHHA	Uncharacterized protein (Fragment) OS=Ophiophagus hanna	OPHHA	3
1948	4.82	4.83	39.9	14.14	14.14	tr|V8N4P6|V8N4P6_OPHHA	Uncharacterized protein (Fragment) OS=Ophiophagus hanna	OPHHA	3
1949	4.82	4.82	47.48	26.62	26.62	tr|V8NVP9|V8NVP9_OPHHA	Mitochondrial import receptor subunit TOM22-like protei	OPHHA	4
1950	4.81	4.86	38.63	8.664	8.664	tr|V8N5Q6|V8N5Q6_OPHHA	Dipeptidyl peptidase 8 (Fragment) OS=Ophiophagus hannah	OPHHA	4
1951	4.81	4.81	52.02	6.404	4.532	tr|V8NUQ6|V8NUQ6_OPHHA	Nuclear receptor coactivator 7 (Fragment) OS=Ophiophagu	OPHHA	3
1952	4.81	4.81	51.33	6.726	6.549	tr|V8NHB3|V8NHB3_OPHHA	Synembryn-A OS=Ophiophagus hannah GN=RIC8A PE=4 SV=1	OPHHA	3
1953	4.81	4.81	43.82	6.242	4.841	tr|V8NPR4|V8NPR4_OPHHA	Rho guanine nucleotide exchange factor 6 (Fragment) OS=	OPHHA	3
1954	4.81	4.81	52.05	14.38	10.27	tr|V8P9J4|V8P9J4_OPHHA	CCA tRNA nucleotidyltransferase 1, mitochondrial (Fragm	OPHHA	3
1955	4.81	4.81	59.93	15.6	12.77	tr|V8NP32|V8NP32_OPHHA	Carnitine O-acetyltransferase (Fragment) OS=Ophiophagus	OPHHA	3
1956	4.8	4.81	58.08	11.98	11.98	tr|V8NCL0|V8NCL0_OPHHA	G-protein-signaling modulator 1 (Fragment) OS=Ophiophag	OPHHA	3
1957	4.8	4.8	46.38	18.84	9.42	tr|V8PDI1|V8PDI1_OPHHA	Sarcolemmal membrane-associated protein (Fragment) OS=O	OPHHA	2
1958	4.78	4.78	56.06	17.25	7.547	tr|V8PFR2|V8PFR2_OPHHA	BRCA1-A complex subunit BRE OS=Ophiophagus hannah GN=BR	OPHHA	2
1959	4.77	4.77	60	16.67	14.17	tr|V8P8D5|V8P8D5_OPHHA	F-box-like/WD repeat-containing protein TBL1XR1 (Fragme	OPHHA	3
1960	4.76	4.76	56.53	16.76	9.747	tr|V8NHS3|V8NHS3_OPHHA	Sister chromatid cohesion protein PDS5-like A-A OS=Ophi	OPHHA	3
1961	4.76	4.76	57.66	34.23	14.86	tr|V8PG36|V8PG36_OPHHA	Ras-related protein Rab-32 (Fragment) OS=Ophiophagus ha	OPHHA	3
1962	4.74	4.76	24.45	4.679	3.396	tr|V8P568|V8P568_OPHHA	Cadherin-1 (Fragment) OS=Ophiophagus hannah GN=CDH1 PE=	OPHHA	3
1963	4.74	4.74	67.33	42.57	42.57	tr|V8N579|V8N579_OPHHA	Uncharacterized protein (Fragment) OS=Ophiophagus hanna	OPHHA	3
1964	4.73	4.73	62	6.4	3.6	tr|V8P6Z0|V8P6Z0_OPHHA	Lysine-specific histone demethylase 1A OS=Ophiophagus h	OPHHA	2
1965	4.72	4.72	62.22	19.11	19.11	tr|V8NNK7|V8NNK7_OPHHA	MOB-like protein phocein OS=Ophiophagus hannah GN=MOB4	OPHHA	3
1966	4.71	4.71	58.92	13.13	10.44	tr|V8P0X7|V8P0X7_OPHHA	Neural Wiskott-Aldrich syndrome protein OS=Ophiophagus	OPHHA	2
1967	4.71	4.71	36.01	10.71	10.71	tr|V8N205|V8N205_OPHHA	Pigment epithelium-derived factor (Fragment) OS=Ophioph	OPHHA	3
1968	4.71	4.71	48.98	14.97	14.97	tr|V8NHV6|V8NHV6_OPHHA	Ankyrin repeat and SOCS box protein 9 OS=Ophiophagus ha	OPHHA	3
1969	4.71	4.71	86.05	26.74	26.74	tr|V8NYT5|V8NYT5_OPHHA	Cytochrome c oxidase subunit 6B1 OS=Ophiophagus hannah	OPHHA	3
1970	4.7	4.7	42.86	12.5	5.893	tr|V8NNA5|V8NNA5_OPHHA	Pre-mRNA 3'-end-processing factor FIP1 (Fragment) OS=Op	OPHHA	2
1971	4.7	4.7	46.68	13.27	8.85	tr|V8P2Q4|V8P2Q4_OPHHA	Serine/threonine-protein kinase VRK1 (Fragment) OS=Ophi	OPHHA	2
1972	4.7	4.7	42.51	15.2	15.2	tr|V8NX13|V8NX13_OPHHA	Glycoprotein endo-alpha-1,2-mannosidase (Fragment) OS=O	OPHHA	4
1973	4.7	4.7	54	24	24	tr|V8N653|V8N653_OPHHA	Putative RNA-binding protein EIF1AD OS=Ophiophagus hann	OPHHA	3
1974	4.68	4.69	72.94	19.27	15.14	tr|V8PH06|V8PH06_OPHHA	Calcyclin-binding protein OS=Ophiophagus hannah GN=CACY	OPHHA	2
1975	4.67	4.67	38.54	7.053	4.786	tr|V8NPH2|V8NPH2_OPHHA	D-2-hydroxyglutarate dehydrogenase, mitochondrial (Frag	OPHHA	3
1976	4.67	4.67	26.74	6.316	4.421	tr|V8N7I7|V8N7I7_OPHHA	N-acetylmuramoyl-L-alanine amidase (Fragment) OS=Ophiop	OPHHA	2
1977	4.67	4.67	35.89	10.68	10.68	tr|V8NRF9|V8NRF9_OPHHA	Protein NDRG2 (Fragment) OS=Ophiophagus hannah GN=NDRG2	OPHHA	4
1978	4.67	4.67	50.91	33.94	20.61	tr|V8NQC2|V8NQC2_OPHHA	Selenoprotein T (Fragment) OS=Ophiophagus hannah GN=SEL	OPHHA	2
1979	4.65	4.65	65.58	20.47	15.81	tr|V8NLP3|V8NLP3_OPHHA	Serine/threonine-protein phosphatase 2A activator OS=Op	OPHHA	3
1980	4.65	4.65	61.27	27.46	27.46	tr|V8N7Q4|V8N7Q4_OPHHA	Uncharacterized protein (Fragment) OS=Ophiophagus hanna	OPHHA	4
1981	4.65	4.65	42.86	26.53	19.05	tr|V8N722|V8N722_OPHHA	Calcium-regulated heat stable protein 1 OS=Ophiophagus	OPHHA	2
1982	4.64	4.64	43.69	6.99	6.602	tr|V8PB77|V8PB77_OPHHA	General transcription factor IIF subunit 1 OS=Ophiophag	OPHHA	2
1983	4.64	4.64	26.84	5.911	5.911	tr|V8PEK3|V8PEK3_OPHHA	T-cell immunomodulatory protein (Fragment) OS=Ophiophag	OPHHA	3
1984	4.64	4.64	41.25	11.27	7.914	tr|V8NY35|V8NY35_OPHHA	Alpha-1,3/1,6-mannosyltransferase ALG2 OS=Ophiophagus h	OPHHA	2
1985	4.63	4.67	42.19	11.87	8.75	tr|V8P8T9|V8P8T9_OPHHA	Poly [ADP-ribose] polymerase (Fragment) OS=Ophiophagus	ADP-ribo:	2
1986	4.63	4.63	38.38	4.112	1.845	tr|V8NWQ3|V8NWQ3_OPHHA	Teneurin-3 (Fragment) OS=Ophiophagus hannah GN=ODZ3 PE=	OPHHA	2
1987	4.63	4.63	26.01	8.742	6.823	tr|V8PHI0|V8PHI0_OPHHA	Hyaluronidase (Fragment) OS=Ophiophagus hannah GN=HYAL2	OPHHA	2
1988	4.62	4.62	41.73	4.086	3.405	tr|V8NG54|V8NG54_OPHHA	Carboxypeptidase D OS=Ophiophagus hannah GN=CPD PE=4 SV	OPHHA	3
1989	4.61	5.09	36.81	11.49	4.178	tr|V8NAP3|V8NAP3_OPHHA	Dimethylaniline monooxygenase [N-oxide-forming] 5 (Frag	N-oxide-:	3
1990	4.61	4.66	46.83	8	5.833	tr|V8NPG8|V8NPG8_OPHHA	Myosin-XIX (Fragment) OS=Ophiophagus hannah GN=myo19 PE	OPHHA	3
1991	4.61	4.61	33.83	3.58	2.469	tr|V8NPF7|V8NPF7_OPHHA	Kininogen-1 (Fragment) OS=Ophiophagus hannah GN=KNG1 PE	OPHHA	2
1992	4.61	4.61	35.62	13.36	6.849	tr|V8PCI5|V8PCI5_OPHHA	Nuclear ubiquitous casein and cyclin-dependent kinases	OPHHA	2
1993	4.6	4.61	34.96	5.436	4.311	tr|V8PER9|V8PER9_OPHHA	Syntaxin-binding protein 5 (Fragment) OS=Ophiophagus ha	OPHHA	3
1994	4.6	4.6	48.07	21.55	12.15	tr|V8P805|V8P805_OPHHA	Prefoldin subunit 3 (Fragment) OS=Ophiophagus hannah GN	OPHHA	2
1995	4.59	4.59	41.55	14.61	14.61	tr|V8NRL3|V8NRL3_OPHHA	C-terminal-binding protein 1 (Fragment) OS=Ophiophagus	OPHHA	4
1996	4.58	4.58	40.86	11.11	5.376	tr|V8NG22|V8NG22_OPHHA	Nicalin (Fragment) OS=Ophiophagus hannah GN=NCLN PE=4 S	OPHHA	2
1997	4.58	4.58	25.67	10.16	6.15	tr|V8P6J2|V8P6J2_OPHHA	Cytosolic 5'-nucleotidase 1B OS=Ophiophagus hannah GN=N	OPHHA	2
1998	4.57	5.46	34.63	13.42	8.366	tr|V8NGP6|V8NGP6_OPHHA	Tight junction protein ZO-3 (Fragment) OS=Ophiophagus h	OPHHA	4
1999	4.57	4.57	48.43	8.142	4.175	tr|V8PEU4|V8PEU4_OPHHA	Cleavage and polyadenylation specificity factor subunit	OPHHA	2
2000	4.57	4.57	53.21	9.786	6.728	tr|V8NFP3|V8NFP3_OPHHA	Far upstream element-binding protein 3 (Fragment) OS=Op	OPHHA	2
2001	4.57	4.57	54.19	25.11	18.06	tr|V8NEF7|V8NEF7_OPHHA	Cytosolic 5'-nucleotidase III-like protein OS=Ophiophag	OPHHA	3
2002	4.56	4.97	62.43	29.28	14.36	tr|V8NXY5|V8NXY5_OPHHA	N(G),N(G)-dimethylarginine dimethylaminohydrolase 1 OS=	OPHHA	2
2003	4.56	4.56	31.56	4.734	2.86	tr|V8PG14|V8PG14_OPHHA	Splicing factor, arginine/serine-rich 15 OS=Ophiophagus	OPHHA	2

2004	4.56	4.56	43.93	7.151	3.746	tr|V8PJ81|V8PJ81_OPHHA Protein FAM59A (Fragment) OS=Ophiophagus hannah GN=FAM5	OPHHA	3
2005	4.56	4.56	39.31	6.528	3.333	tr|V8NU49|V8NU49_OPHHA Molybdenum cofactor sulfurase (Fragment) OS=Ophiophagus	OPHHA	3
2006	4.56	4.56	49.48	15.81	8.247	tr|V8PAK9|V8PAK9_OPHHA Nitric oxide synthase-interacting protein OS=Ophiophagu	OPHHA	2
2007	4.56	4.56	37.23	17.75	14.29	tr|V8P828|V8P828_OPHHA Transmembrane protein OS=Ophiophagus hannah GN=TMEM111	OPHHA	2
2008	4.54	4.54	31.09	3.438	1.953	tr|V8P1S9|V8P1S9_OPHHA Nuclear pore complex protein (Fragment) OS=Ophiophagus	OPHHA	2
2009	4.54	4.54	48.58	19.87	13.56	tr|V8PBA5|V8PBA5_OPHHA CD2 antigen cytoplasmic tail-binding protein 2 OS=Ophio	OPHHA	2
2010	4.54	4.54	34.01	15.65	10.88	tr|V8P692|V8P692_OPHHA Galectin OS=Ophiophagus hannah GN=Lgals8 PE=4 SV=1	OPHHA	3
2011	4.54	4.54	28.4	12.08	3.625	tr|V8NH24|V8NH24_OPHHA Elongation factor Ts OS=Ophiophagus hannah GN=TSFM PE=3	OPHHA	2
2012	4.54	4.54	35	29.17	20.83	tr|V8P6R1|V8P6R1_OPHHA Uncharacterized protein (Fragment) OS=Ophiophagus hanna	OPHHA	4
2013	4.53	4.53	56.29	5.478	4.545	tr|V8P6X7|V8P6X7_OPHHA Zinc finger CCCH domain-containing protein 7B (Fragment	OPHHA	3
2014	4.53	4.53	59.26	12.42	7.407	tr|V8NV98|V8NV98_OPHHA Antithrombin-III (Fragment) OS=Ophiophagus hannah GN=SE	OPHHA	3
2015	4.53	4.53	34.29	6.699	6.699	tr|V8NMZ6|V8NMZ6_OPHHA Mini-chromosome maintenance complex-binding protein OS=	OPHHA	3
2016	4.53	4.53	42.81	15.72	11.04	tr|V8PDV0|V8PDV0_OPHHA Acidic leucine-rich nuclear phosphoprotein 32 family me	OPHHA	2
2017	4.5	4.5	47.36	3.226	3.226	tr|V8PIU3|V8PIU3_OPHHA Signal transducer and activator of transcription 2 OS=O	OPHHA	2
2018	4.5	4.5	42.46	8.101	3.771	tr|V8NU08|V8NU08_OPHHA Conserved oligomeric Golgi complex subunit 2 OS=Ophioph	OPHHA	2
2019	4.5	4.5	42.82	16.8	8.401	tr|V8PDD1|V8PDD1_OPHHA Splicing factor U2AF 35 kDa subunit (Fragment) OS=Ophio	OPHHA	2
2020	4.5	4.5	43.41	8.683	8.683	tr|V8P1K8|V8P1K8_OPHHA 39S ribosomal protein L44, mitochondrial (Fragment) OS=	OPHHA	3
2021	4.5	4.5	78.99	20.29	10.87	tr|V8P9M5|V8P9M5_OPHHA MICOS complex subunit (Fragment) OS=Ophiophagus hannah	OPHHA	2
2022	4.5	4.5	46.46	36.22	30.71	tr|V8PF04|V8PF04_OPHHA Synaptojanin-2-binding protein (Fragment) OS=Ophiophagu	OPHHA	4
2023	4.5	4.5	33.18	12.15	12.15	tr|V8PFX7|V8PFX7_OPHHA Acyl-protein thioesterase 1 (Fragment) OS=Ophiophagus h	OPHHA	3
2024	4.49	4.49	79.23	14.01	9.662	tr|V8PIJ7|V8PIJ7_OPHHA 28S ribosomal protein S34, mitochondrial (Fragment) OS=	OPHHA	2
2025	4.48	4.49	48.81	14.25	10.03	tr|V8P926|V8P926_OPHHA Geranylgeranyl transferase type-1 subunit beta (Fragmen	OPHHA	3
2026	4.48	4.48	33.82	1.984	0.8475	tr|V8PGB8|V8PGB8_OPHHA Putative E3 ubiquitin-protein ligase HERC1 (Fragment) O	OPHHA	3
2027	4.48	4.48	38.99	7.798	3.83	tr|V8PJI1|V8PJI1_OPHHA Translation initiation factor IF-2, mitochondrial OS=Op	OPHHA	2
2028	4.48	4.48	25.63	5.854	5.854	tr|V8P2D0|V8P2D0_OPHHA Uncharacterized protein OS=Ophiophagus hannah GN=L345_0	OPHHA	3
2029	4.47	4.59	54.8	2.954	1.352	tr|V8P9M1|V8P9M1_OPHHA Adenomatous polyposis coli protein (Fragment) OS=Ophiop	OPHHA	3
2030	4.47	4.47	51.41	7.699	3.808	tr|V8P9U0|V8P9U0_OPHHA Activity-dependent neuroprotector homeobox protein (Fra	OPHHA	3
2031	4.47	4.47	43.98	6.501	4.011	tr|V8P714|V8P714_OPHHA Phosphatidylinositol 3-kinase regulatory subunit alpha	OPHHA	2
2032	4.47	4.47	34.64	8.776	6.467	tr|V8PFK1|V8PFK1_OPHHA DnaJ-like subfamily B member 6 OS=Ophiophagus hannah GN	OPHHA	2
2033	4.47	4.47	58.28	35.1	27.15	tr|V8NMI0|V8NMI0_OPHHA Uncharacterized protein (Fragment) OS=Ophiophagus hanna	OPHHA	3
2034	4.46	6.21	67	11.71	6.911	tr|V8PBD3|V8PBD3_OPHHA Guanine nucleotide-binding protein G(Q) subunit alpha O	OPHHA	3
2035	4.46	4.57	58.33	25.52	19.27	tr|V8P642|V8P642_OPHHA COP9 signalosome complex subunit 8 (Fragment) OS=Ophiop	OPHHA	2
2036	4.46	4.46	44.1	10.66	5.574	tr|V8NRL0|V8NRL0_OPHHA Ribosome-releasing factor 2, mitochondrial (Fragment) O	OPHHA	2
2037	4.46	4.46	77.05	11.89	11.89	tr|V8NRI8|V8NRI8_OPHHA Transcription factor A, mitochondrial OS=Ophiophagus ha	OPHHA	3
2038	4.46	4.46	32.75	9.607	3.712	tr|V8NBF7|V8NBF7_OPHHA Cytochrome protein (Fragment) OS=Ophiophagus hannah GN=	OPHHA	3
2039	4.46	4.46	56.86	11.37	11.37	tr|V8P430|V8P430_OPHHA Abhydrolase domain-containing protein 10, mitochondrial	OPHHA	3
2040	4.45	4.48	35.63	5.572	5.572	tr|V8PBK1|V8PBK1_OPHHA GA-binding protein alpha chain (Fragment) OS=Ophiophagu	OPHHA	4
2041	4.45	4.45	51.67	23.33	17.22	tr|V8PAT4|V8PAT4_OPHHA Uncharacterized protein (Fragment) OS=Ophiophagus hanna	OPHHA	2
2042	4.44	4.44	43.59	14.1	11.22	tr|V8NEB4|V8NEB4_OPHHA WD repeat-containing protein 18 OS=Ophiophagus hannah G	OPHHA	3
2043	4.44	4.44	53.85	22.56	11.28	tr|V8N8V6|V8N8V6_OPHHA Uncharacterized protein (Fragment) OS=Ophiophagus hanna	OPHHA	2
2044	4.44	4.44	33.74	15.23	11.93	tr|V8NU72|V8NU72_OPHHA Mitochondrial carnitine/acylcarnitine carrier protein (	OPHHA	4
2045	4.43	4.43	50.85	16.72	7.85	tr|V8NV82|V8NV82_OPHHA Tyrosine-protein kinase Yes (Fragment) OS=Ophiophagus h	OPHHA	2
2046	4.42	5.38	38.78	7.638	5.405	tr|V8PEV2|V8PEV2_OPHHA Integrin beta OS=Ophiophagus hannah GN=ITGB2 PE=3 SV=1	OPHHA	4
2047	4.42	4.42	36.24	5.529	3.529	tr|V8P610|V8P610_OPHHA Transmembrane and TPR repeat-containing protein 3 (Frag	OPHHA	2
2048	4.41	4.41	59.12	9.343	8.321	tr|V8P4Z7|V8P4Z7_OPHHA Nuclear autoantigenic sperm protein OS=Ophiophagus hann	OPHHA	3
2049	4.41	4.41	48.98	10.24	6.485	tr|V8PHJ1|V8PHJ1_OPHHA Syntaxin-binding protein 2 OS=Ophiophagus hannah GN=STX	OPHHA	3
2050	4.41	4.41	46.5	6.379	4.321	tr|V8NMF1|V8NMF1_OPHHA Regucalcin OS=Ophiophagus hannah GN=RGN PE=4 SV=1	OPHHA	2
2051	4.41	4.41	20.87	12.46	6.542	tr|V8NE60|V8NE60_OPHHA Wiskott-Aldrich syndrome protein family member 2 (Fragm	OPHHA	3
2052	4.4	4.4	37.94	10.13	7.074	tr|V8P9B9|V8P9B9_OPHHA Guanine nucleotide-binding protein-like 1 (Fragment) OS	OPHHA	3
2053	4.4	4.4	27.04	7.091	3.966	tr|V8NIX5|V8NIX5_OPHHA Protein lin-54-like protein (Fragment) OS=Ophiophagus h	OPHHA	2
2054	4.4	4.4	51.16	14.85	7.921	tr|V8NYL1|V8NYL1_OPHHA Beta-hexosaminidase subunit alpha (Fragment) OS=Ophioph	OPHHA	2
2055	4.4	4.4	56.25	16.52	16.52	tr|V8NGL5|V8NGL5_OPHHA Lys-63-specific deubiquitinase BRCC36 OS=Ophiophagus ha	OPHHA	3
2056	4.4	4.4	31.18	13.53	9.412	tr|V8NQR0|V8NQR0_OPHHA Cytochrome c oxidase subunit 4 isoform 1, mitochondrial	OPHHA	2
2057	4.39	4.39	28.38	5.135	3.268	tr|V8PFV4|V8PFV4_OPHHA Pumilio-like 2 (Fragment) OS=Ophiophagus hannah GN=PUM2	OPHHA	3
2058	4.39	4.39	48.77	5.967	5.967	tr|V8NVA8|V8NVA8_OPHHA N-acetylglucosamine-6-sulfatase (Fragment) OS=Ophiophag	OPHHA	3
2059	4.38	4.38	55.43	12	12	tr|V8NV66|V8NV66_OPHHA Polypeptide N-acetylgalactosaminyltransferase OS=Ophiop	OPHHA	3
2060	4.38	4.38	32.04	12.32	9.155	tr|V8P2L3|V8P2L3_OPHHA Dihydroorotate dehydrogenase (quinone), mitochondrial O	OPHHA	2
2061	4.37	8.47	45.31	17.8	15.21	tr|V8NXU1|V8NXU1_OPHHA Serine/threonine-protein phosphatase OS=Ophiophagus han	OPHHA	4
2062	4.36	4.36	36.63	6.79	6.79	tr|V8NM42|V8NM42_OPHHA 26S proteasome non-ATPase regulatory subunit 5 (Fragmen	OPHHA	3
2063	4.35	4.35	36.27	10.48	6.499	tr|V8P7I0|V8P7I0_OPHHA Epoxide hydrolase 1 OS=Ophiophagus hannah GN=Ephx1 PE=4	OPHHA	3
2064	4.34	4.34	68.93	7.37	5.058	tr|V8P5F3|V8P5F3_OPHHA MAP/microtubule affinity-regulating kinase 3 OS=Ophioph	OPHHA	2
2065	4.34	4.34	54.91	10.04	6.197	tr|V8NG91|V8NG91_OPHHA Vacuolar protein sorting-associated protein 33A (Fragme	OPHHA	2
2066	4.34	4.34	39.73	21	17.81	tr|V8NVM2|V8NVM2_OPHHA Charged multivesicular body protein 5 OS=Ophiophagus ha	OPHHA	2
2067	4.33	8.67	39.75	8.711	8.711	tr|V8PGS5|V8PGS5_OPHHA Hydroxymethylglutaryl-CoA synthase, cytoplasmic (Fragme	OPHHA	5
2068	4.33	4.7	57.05	15.2	5.507	tr|V8NJJ4|V8NJJ4_OPHHA Beclin-1 (Fragment) OS=Ophiophagus hannah GN=BECN1 PE=4	OPHHA	2
2069	4.33	4.33	46.15	23.08	14.79	tr|V8P079|V8P079_OPHHA Sideroflexin-1 (Fragment) OS=Ophiophagus hannah GN=SFXN	OPHHA	4
2070	4.32	4.35	42.45	8.955	6.965	tr|V8NP51|V8NP51_OPHHA Fibulin-1 OS=Ophiophagus hannah GN=FBLN1 PE=4 SV=1	OPHHA	2
2071	4.32	4.32	58.33	12.38	10	tr|V8P3I6|V8P3I6_OPHHA Caveolin-1 (Fragment) OS=Ophiophagus hannah GN=CAV1 PE=	OPHHA	2
2072	4.32	4.32	38.42	11.99	10.08	tr|V8NQ97|V8NQ97_OPHHA Ras-related GTP-binding protein D (Fragment) OS=Ophioph	OPHHA	3
2073	4.31	6.36	48.21	22.05	17.95	tr|V8NI12|V8NI12_OPHHA Ras-related protein Rab-6B (Fragment) OS=Ophiophagus ha	OPHHA	3
2074	4.31	4.82	30.41	24.23	14.43	tr|V8NYE1|V8NYE1_OPHHA Ras-related protein Rap-2a (Fragment) OS=Ophiophagus ha	OPHHA	2
2075	4.3	4.35	38.16	3.223	1.547	tr|V8PJ12|V8PJ12_OPHHA AT-rich interactive domain-containing protein 1A (Fragm	OPHHA	2
2076	4.3	4.3	51.75	5.891	4.628	tr|V8PEX9|V8PEX9_OPHHA Platelet endothelial cell adhesion molecule OS=Ophiopha	OPHHA	3
2077	4.3	4.3	72	15.47	12.8	tr|V8NYB4|V8NYB4_OPHHA Polymerase delta-interacting protein 3 (Fragment) OS=Op	OPHHA	3
2078	4.3	4.3	46.88	8.545	6.467	tr|V8NLH6|V8NLH6_OPHHA Regulator of nonsense transcripts 3B (Fragment) OS=Ophi	OPHHA	2
2079	4.3	4.3	34.07	7.195	3.965	tr|V8NR89|V8NR89_OPHHA TGF-beta-activated kinase 1 and MAP3K7-binding protein	OPHHA	2
2080	4.3	4.3	82.02	38.2	29.21	tr|V8PHT9|V8PHT9_OPHHA Mitochondrial import inner membrane translocase subunit	OPHHA	2
2081	4.29	4.36	46.28	2.212	0.9383	tr|V8PHZ8|V8PHZ8_OPHHA Lysosomal alpha-glucosidase (Fragment) OS=Ophiophagus h	OPHHA	2
2082	4.29	4.29	43.21	12.23	7.065	tr|V8ND90|V8ND90_OPHHA Four and a half LIM domains protein 3 (Fragment) OS=Oph	OPHHA	2
2083	4.29	4.29	69.14	20.37	20.37	tr|V8NSN1|V8NSN1_OPHHA 39S ribosomal protein L13, mitochondrial OS=Ophiophagus	OPHHA	3
2084	4.29	4.29	60	35	22.5	tr|V8PFK5|V8PFK5_OPHHA Enoyl-CoA hydratase domain-containing protein 2, mitoch	OPHHA	2
2085	4.28	7.73	44.21	18.42	11.32	tr|V8PAK4|V8PAK4_OPHHA LIM and senescent cell antigen-like-containing domain p	OPHHA	4
2086	4.28	4.66	39.86	14.99	5.467	tr|V8NTT4|V8NTT4_OPHHA Serine/threonine-protein phosphatase (Fragment) OS=Ophi	OPHHA	2
2087	4.28	4.28	43.07	6.01	4.341	tr|V8N842|V8N842_OPHHA Vacuolar protein sorting-associated protein 33B (Fragme	OPHHA	2
2088	4.28	4.28	44.6	18.56	8.31	tr|V8PI92|V8PI92_OPHHA Protein TSSC1 (Fragment) OS=Ophiophagus hannah GN=TSSC1	OPHHA	2
2089	4.27	8.09	56.76	7.206	4.738	tr|V8N6R8|V8N6R8_OPHHA Uncharacterized protein (Fragment) OS=Ophiophagus hanna	OPHHA	5
2090	4.26	4.26	38.7	6.649	3.59	tr|V8NU29|V8NU29_OPHHA Epithelial splicing regulatory protein 2 OS=Ophiophagus	OPHHA	2
2091	4.26	4.26	31.8	11.98	7.834	tr|V8N8B1|V8N8B1_OPHHA Forkhead box protein K1 (Fragment) OS=Ophiophagus hanna	OPHHA	2
2092	4.26	4.26	32.12	13.87	10.22	tr|V8P7H6|V8P7H6_OPHHA DNA-directed RNA polymerases I and III subunit RPAC1 (F	OPHHA	2
2093	4.26	4.26	40.08	15.87	11.9	tr|V8NUG8|V8NUG8_OPHHA Coiled-coil domain-containing protein 43 OS=Ophiophagus	OPHHA	2
2094	4.26	4.26	70.1	46.39	46.39	tr|V8P8K3|V8P8K3_OPHHA Uncharacterized protein OS=Ophiophagus hannah GN=L345_0	OPHHA	3
2095	4.25	6.48	68.16	26.53	16.33	tr|V8NQ44|V8NQ44_OPHHA Ras-related protein Rab-30 (Fragment) OS=Ophiophagus ha	OPHHA	4
2096	4.25	4.25	50.69	12.15	8.681	tr|V8PHC2|V8PHC2_OPHHA EF-hand domain-containing family member A2 (Fragment) O	OPHHA	2
2097	4.25	4.25	42.09	11.16	5.349	tr|V8PGI4|V8PGI4_OPHHA Talin-2 (Fragment) OS=Ophiophagus hannah GN=Tln2 PE=4 S	OPHHA	2
2098	4.24	4.59	42.52	10.63	7.592	tr|V8NJ07|V8NJ07_OPHHA Coronin OS=Ophiophagus hannah GN=CORO1A PE=3 SV=1	OPHHA	3
2099	4.24	4.39	55.4	15.63	5.966	tr|V8NBM9|V8NBM9_OPHHA Keratin, type II cytoskeletal 5 OS=Ophiophagus hannah G	OPHHA	2

2100	4.24	4.24	33.83	4.505	2.202	tr|V8NHK4|V8NHK4_OPHHA	Uncharacterized protein (Fragment) OS=Ophiophagus hanna	OPHHA	2
2101	4.24	4.24	44.44	11.76	7.19	tr|V8P5W6|V8P5W6_OPHHA	Uncharacterized protein OS=Ophiophagus hannah GN=L345_0	OPHHA	2
2102	4.24	4.24	29.86	16.11	16.11	tr|V8NFZ2|V8NFZ2_OPHHA	Tetraspanin (Fragment) OS=Ophiophagus hannah GN=Tspan2	OPHHA	4
2103	4.23	13.26	64.04	27.45	19.79	tr|V8NIC4|V8NIC4_OPHHA	Sulfotransferase (Fragment) OS=Ophiophagus hannah GN=Su	OPHHA	9
2104	4.23	4.25	29.87	6.74	5.464	tr|V8PDE2|V8PDE2_OPHHA	Glycerophosphoinositol inositolphosphodiesterase GDPD2	OPHHA	2
2105	4.23	4.23	51.58	4.246	2.8	tr|V8NS00|V8NS00_OPHHA	Cell division control protein 6-like protein (Fragment)	OPHHA	2
2106	4.23	4.23	58.87	11.04	7.359	tr|V8P965|V8P965_OPHHA	E3 ubiquitin-protein ligase RNF14 (Fragment) OS=Ophioph	OPHHA	2
2107	4.23	4.23	60.14	36.49	20.95	tr|V8N548|V8N548_OPHHA	Eukaryotic translation initiation factor 3 subunit K OS	OPHHA	2
2108	4.22	4.24	43.67	8.696	6.616	tr|V8NNZ6|V8NNZ6_OPHHA	Engulfment and cell motility protein 3 OS=Ophiophagus h	OPHHA	2
2109	4.22	4.22	36.73	12.73	9.818	tr|V8PAS6|V8PAS6_OPHHA	Beta-lactamase-like protein 2 OS=Ophiophagus hannah GN=	OPHHA	5
2110	4.22	4.22	57.65	49.41	49.41	tr|V8N7U6|V8N7U6_OPHHA	Uncharacterized protein (Fragment) OS=Ophiophagus hanna	OPHHA	4
2111	4.19	4.24	42.73	11.63	9.302	tr|V8P316|V8P316_OPHHA	54 kDa 2'-5'-oligoadenylate synthase-like protein 2 (Fr	OPHHA	3
2112	4.19	4.19	50.8	12.03	12.03	tr|V8NWQ8|V8NWQ8_OPHHA	G-rich sequence factor 1 (Fragment) OS=Ophiophagus hann	OPHHA	4
2113	4.19	4.19	44.26	34.43	22.13	tr|V8N491|V8N491_OPHHA	Uncharacterized protein (Fragment) OS=Ophiophagus hanna	OPHHA	2
2114	4.18	4.22	55.46	4.499	2.812	tr|V8P8Z1|V8P8Z1_OPHHA	Mitogen-activated protein kinase kinase kinase 5 (Fragm	OPHHA	2
2115	4.18	4.18	48.6	5.387	5.387	tr|V8PAA6|V8PAA6_OPHHA	Mitogen-activated protein kinase kinase kinase 2 OS=Oph	OPHHA	4
2116	4.18	4.18	38.19	6.496	4.331	tr|V8PGH9|V8PGH9_OPHHA	Protein lunapark (Fragment) OS=Ophiophagus hannah GN=LN	OPHHA	2
2117	4.18	4.18	27.6	8.2	5.6	tr|V8P7X1|V8P7X1_OPHHA	U4/U6 small nuclear ribonucleoprotein Prp31 OS=Ophiopha	OPHHA	2
2118	4.18	4.18	51.2	7.229	7.229	tr|V8NV71|V8NV71_OPHHA	1-acylglycerol-3-phosphate O-acyltransferase ABHD5 OS=O	OPHHA	2
2119	4.18	4.18	54.76	29.76	19.05	tr|V8PGV1|V8PGV1_OPHHA	Uncharacterized protein (Fragment) OS=Ophiophagus hanna	OPHHA	2
2120	4.17	4.17	47.48	8.14	5.233	tr|V8N6H6|V8N6H6_OPHHA	Uncharacterized protein OS=Ophiophagus hannah GN=L345_1	OPHHA	3
2121	4.17	4.17	75.26	21.13	16.49	tr|V8NED3|V8NED3_OPHHA	Uncharacterized protein (Fragment) OS=Ophiophagus hanna	OPHHA	2
2122	4.17	4.17	69.59	24.74	13.92	tr|V8ND23|V8ND23_OPHHA	Actin-binding LIM protein 1 (Fragment) OS=Ophiophagus h	OPHHA	2
2123	4.17	4.17	20.28	7.692	6.527	tr|V8P590|V8P590_OPHHA	Cadherin-1 (Fragment) OS=Ophiophagus hannah GN=CDH1 PE=	OPHHA	2
2124	4.17	4.17	75.81	33.06	25	tr|V8NVL7|V8NVL7_OPHHA	BAG family molecular chaperone regulator 1 (Fragment) O	OPHHA	3
2125	4.17	4.17	32.37	23.12	18.5	tr|V8N6B2|V8N6B2_OPHHA	Glycerol kinase (Fragment) OS=Ophiophagus hannah GN=GK	OPHHA	2
2126	4.16	4.16	65.66	11.31	6.061	tr|V8P005|V8P005_OPHHA	Katanin p60 ATPase-containing subunit A1 OS=Ophiophagus	OPHHA	2
2126	0.01	2.83	47.92	5.208	5.208	tr|V8PCW7|V8PCW7_OPHHA	Katanin p60 ATPase-containing subunit A-like 1 (Fragmen	OPHHA	2
2127	4.16	4.16	48.76	10.34	3.82	tr|V8NW77|V8NW77_OPHHA	Putative E3 ubiquitin-protein ligase makorin-2 OS=Ophio	OPHHA	2
2128	4.16	4.16	51.42	14.2	9.464	tr|V8P813|V8P813_OPHHA	Mitochondrial glutamate carrier 2 (Fragment) OS=Ophioph	OPHHA	3
2129	4.16	4.16	65.19	20.89	15.19	tr|V8NDV4|V8NDV4_OPHHA	Annexin (Fragment) OS=Ophiophagus hannah GN=Anxa7 PE=3	OPHHA	2
2130	4.15	4.15	51.59	14.65	9.554	tr|V8NVL9|V8NVL9_OPHHA	ATPase family AAA domain-containing protein 1 OS=Ophiop	OPHHA	2
2131	4.14	4.25	39.02	13.86	8.316	tr|V8N7I0|V8N7I0_OPHHA	Uncharacterized protein (Fragment) OS=Ophiophagus hanna	OPHHA	2
2132	4.14	4.14	34.41	1.795	1.01	tr|V8PB32|V8PB32_OPHHA	Ankyrin repeat domain-containing protein 17 (Fragment)	OPHHA	2
2133	4.14	4.14	25.74	6.087	4.174	tr|V8PBH5|V8PBH5_OPHHA	Amino acid transporter (Fragment) OS=Ophiophagus hannah	OPHHA	2
2134	4.13	4.27	62.96	14.81	9.972	tr|V8NM49|V8NM49_OPHHA	Macrophage erythroblast attacher (Fragment) OS=Ophiopha	OPHHA	2
2135	4.13	4.13	36.61	4.805	2.059	tr|V8PI32|V8PI32_OPHHA	Desmocollin-2 OS=Ophiophagus hannah GN=DSC2 PE=4 SV=1	OPHHA	2
2136	4.13	4.13	43.21	4.14	3.234	tr|V8PG24|V8PG24_OPHHA	Ubiquitin carboxyl-terminal hydrolase 16 (Fragment) OS=	OPHHA	2
2137	4.13	4.13	79.05	15.42	15.42	tr|V8PE10|V8PE10_OPHHA	Eukaryotic translation initiation factor 4H (Fragment)	OPHHA	2
2138	4.13	4.13	56.73	15.51	12.65	tr|V8NGH3|V8NGH3_OPHHA	Carboxypeptidase B2 (Fragment) OS=Ophiophagus hannah GN	OPHHA	2
2139	4.13	4.13	30.37	10.03	10.03	tr|V8PHN6|V8PHN6_OPHHA	Uncharacterized protein (Fragment) OS=Ophiophagus hanna	OPHHA	3
2140	4.13	4.13	12.35	5.12	4.217	tr|V8P7X5|V8P7X5_OPHHA	Transporter (Fragment) OS=Ophiophagus hannah GN=SLC6A6	OPHHA	2
2141	4.13	4.13	19.89	18.23	14.36	tr|V8NPE1|V8NPE1_OPHHA	Tryptophan-rich protein OS=Ophiophagus hannah GN=Wrb PE	OPHHA	2
2142	4.12	4.12	70.95	9.385	3.017	tr|V8PII6|V8PII6_OPHHA	Pre-mRNA-splicing factor SYF1 (Fragment) OS=Ophiophagus	OPHHA	2
2143	4.12	4.12	26.97	11.51	8.224	tr|V8NKB0|V8NKB0_OPHHA	Protein phosphatase 1F (Fragment) OS=Ophiophagus hannah	OPHHA	2
2144	4.12	4.12	40.09	18.06	11.01	tr|V8P7K5|V8P7K5_OPHHA	L-xylulose reductase (Fragment) OS=Ophiophagus hannah G	OPHHA	2
2145	4.12	4.12	42.31	15.38	15.38	tr|V8NC76|V8NC76_OPHHA	E3 ubiquitin-protein ligase UBR4 (Fragment) OS=Ophiopha	OPHHA	2
2146	4.12	4.12	49.62	20.3	20.3	tr|V8N454|V8N454_OPHHA	Proteasome subunit beta type-6 (Fragment) OS=Ophiophagu	OPHHA	2
2147	4.11	5.13	40.19	5.192	4.038	tr|V8NCI9|V8NCI9_OPHHA	Zinc finger CCCH-type antiviral protein 1 OS=Ophiophagu	OPHHA	3
2148	4.11	5.06	41.3	5.286	3.965	tr|V8NTC6|V8NTC6_OPHHA	KN motif and ankyrin repeat domain-containing protein 2	OPHHA	3
2149	4.11	4.82	54.9	2.835	2.255	tr|V8P0I1|V8P0I1_OPHHA	Serine/threonine-protein kinase MRCK alpha (Fragment) O	OPHHA	3
2150	4.11	4.14	42.6	3.924	3.924	tr|V8P5W3|V8P5W3_OPHHA	Rho-guanine nucleotide exchange factor (Fragment) OS=Op	OPHHA	2
2151	4.11	4.11	43.13	5.005	4.26	tr|V8NHJ1|V8NHJ1_OPHHA	DNA mismatch repair protein Msh2 (Fragment) OS=Ophiopha	OPHHA	3
2152	4.11	4.11	73.26	12.09	12.09	tr|V8PC28|V8PC28_OPHHA	Translation initiation factor IF-3, mitochondrial (Frag	OPHHA	4
2153	4.11	4.11	58.97	26.5	20.94	tr|V8NLV6|V8NLV6_OPHHA	Ubiquitin fusion degradation protein 1-like protein OS=	OPHHA	3
2154	4.11	4.11	56.25	34.9	22.92	tr|V8NQL9|V8NQL9_OPHHA	Death-associated protein 1 (Fragment) OS=Ophiophagus ha	OPHHA	2
2155	4.11	4.11	59.09	41.82	30.91	tr|V8PIY9|V8PIY9_OPHHA	Cytochrome b5 OS=Ophiophagus hannah GN=CYB5A PE=3 SV=1	OPHHA	3
2156	4.1	4.1	78.3	33.02	26.42	tr|V8P3E1|V8P3E1_OPHHA	Serine/threonine-protein kinase 38 (Fragment) OS=Ophiop	OPHHA	2
2157	4.1	4.1	27.06	17.65	12.94	tr|V8NDX2|V8NDX2_OPHHA	Surfeit locus protein 4 (Fragment) OS=Ophiophagus hanna	OPHHA	2
2158	4.09	12.17	62.67	35.48	26.73	tr|V8NJY7|V8NJY7_OPHHA	Glycerol-3-phosphate dehydrogenase [NAD(+)] (Fragment)	NAD(+)	8
2159	4.09	7.36	47.08	16.99	16.99	tr|V8NZP8|V8NZP8_OPHHA	Putative RNA-binding protein Luc7-like 1 (Fragment) OS=	OPHHA	5
2160	4.09	4.15	69.37	15.14	15.14	tr|V8PCH6|V8PCH6_OPHHA	Peptidyl-prolyl cis-trans isomerase OS=Ophiophagus hann	OPHHA	3
2161	4.09	4.11	47.08	17.08	17.08	tr|V8PA39|V8PA39_OPHHA	Nuclear factor 1 C-type (Fragment) OS=Ophiophagus hanna	OPHHA	3
2162	4.09	4.09	36.4	4.159	1.863	tr|V8PHQ0|V8PHQ0_OPHHA	GC-rich sequence DNA-binding factor 1 (Fragment) OS=Oph	OPHHA	3
2163	4.09	4.09	41.33	6.476	6.476	tr|V8N782|V8N782_OPHHA	Inositol polyphosphate 5-phosphatase OCRL-1 (Fragment)	OPHHA	3
2164	4.09	4.09	57.08	29.25	22.17	tr|V8NFE5|V8NFE5_OPHHA	Sjoegren syndrome/scleroderma autoantigen 1-like protei	OPHHA	2
2165	4.09	4.09	23.9	8.353	6.497	tr|V8N760|V8N760_OPHHA	Cytochrome P450 (Fragment) OS=Ophiophagus hannah GN=Cyp	OPHHA	3
2166	4.08	4.08	31.07	9.416	4.52	tr|V8NWF6|V8NWF6_OPHHA	Aspartyl-tRNA synthetase, mitochondrial (Fragment) OS=O	OPHHA	2
2167	4.08	4.08	38.99	6.193	6.193	tr|V8NLX4|V8NLX4_OPHHA	Striatin-4 (Fragment) OS=Ophiophagus hannah GN=Strn4 PE	OPHHA	2
2168	4.08	4.08	76.42	27.51	21.83	tr|V8NHR5|V8NHR5_OPHHA	Heme-binding protein 1 (Fragment) OS=Ophiophagus hannah	OPHHA	2
2169	4.07	4.09	62.84	11.06	11.06	tr|V8NCS0|V8NCS0_OPHHA	Colorectal mutant cancer protein (Fragment) OS=Ophiopha	OPHHA	3
2170	4.07	4.08	100	39.8	36.73	tr|V8NII1|V8NII1_OPHHA	Proteasome inhibitor PI31 subunit (Fragment) OS=Ophioph	OPHHA	2
2171	4.07	4.07	42.99	8.314	8.314	tr|V8PIR8|V8PIR8_OPHHA	DDB1-and CUL4-associated factor 13 (Fragment) OS=Ophiop	OPHHA	2
2172	4.07	4.07	42.34	17.52	12.41	tr|V8NCF2|V8NCF2_OPHHA	Calcineurin-like phosphoesterase domain-containing prot	OPHHA	2
2173	4.06	21.27	55.21	16.06	11.81	tr|V8NKW3|V8NKW3_OPHHA	Clathrin heavy chain 1 (Fragment) OS=Ophiophagus hannah	OPHHA	12
2174	4.06	4.86	49.2	12.3	9.091	tr|V8P8Y8|V8P8Y8_OPHHA	39S ribosomal protein L12, mitochondrial OS=Ophiophagus	OPHHA	3
2175	4.06	4.06	48.04	3.538	3.538	tr|V8NS20|V8NS20_OPHHA	Ubiquitin carboxyl-terminal hydrolase 25 OS=Ophiophagus	OPHHA	2
2176	4.06	4.06	33.87	5.376	5.376	tr|V8NYT9|V8NYT9_OPHHA	Lipolysis-stimulated lipoprotein receptor (Fragment) OS	OPHHA	2
2177	4.06	4.06	51.81	8.29	8.29	tr|V8PBM2|V8PBM2_OPHHA	NudC domain-containing protein 3 (Fragment) OS=Ophiopha	OPHHA	2
2178	4.06	4.06	37.5	5.388	5.388	tr|V8N5P4|V8N5P4_OPHHA	ATP-dependent RNA helicase DQX1 (Fragment) OS=Ophiophag	OPHHA	2
2179	4.06	4.06	42.25	15.14	10.56	tr|V8N974|V8N974_OPHHA	Starch-binding domain-containing protein 1 (Fragment) O	OPHHA	3
2180	4.06	4.06	72.22	34.72	34.72	tr|V8N2A8|V8N2A8_OPHHA	Uncharacterized protein (Fragment) OS=Ophiophagus hanna	OPHHA	2
2181	4.05	4.05	54.18	1.657	1.657	tr|V8NG39|V8NG39_OPHHA	Macrophage mannose receptor 1 (Fragment) OS=Ophiophagus	OPHHA	2
2182	4.05	4.05	41.9	2.091	2.091	tr|V8NUE6|V8NUE6_OPHHA	Kinesin-like protein (Fragment) OS=Ophiophagus hannah G	OPHHA	2
2183	4.05	4.05	46.66	3.488	3.488	tr|V8P9R8|V8P9R8_OPHHA	Ankyrin repeat and zinc finger domain-containing protei	OPHHA	2
2184	4.05	4.05	48.5	6.138	6.138	tr|V8NKL3|V8NKL3_OPHHA	Eukaryotic translation initiation factor 2D (Fragment)	OPHHA	2
2185	4.05	4.05	89.19	39.64	25.23	tr|V8NRM2|V8NRM2_OPHHA	Chloride intracellular channel protein 5 (Fragment) OS=	OPHHA	2
2186	4.05	4.05	51.25	17.5	13.12	tr|V8NJ82|V8NJ82_OPHHA	Cytochrome b-c1 complex subunit 8 (Fragment) OS=Ophioph	OPHHA	2
2187	4.05	4.05	75	23.08	23.08	tr|V8NJJ1|V8NJJ1_OPHHA	Exportin-1 (Fragment) OS=Ophiophagus hannah GN=XPO1 PE=	OPHHA	2
2188	4.04	4.76	84.42	20.1	11.06	tr|V8NVD0|V8NVD0_OPHHA	Protein phosphatase 1 regulatory subunit 12B (Fragment)	OPHHA	3
2189	4.04	4.4	55.05	7.423	5.773	tr|V8P5J4|V8P5J4_OPHHA	DnaJ-like subfamily C member 3 OS=Ophiophagus hannah GN	OPHHA	2
2190	4.04	4.06	44.44	13.19	6.944	tr|V8NHC2|V8NHC2_OPHHA	Ribosomal protein S6 kinase beta-1 OS=Ophiophagus hanna	OPHHA	2
2191	4.04	4.06	79.27	20.73	20.73	tr|V8NSF6|V8NSF6_OPHHA	SH3 domain-binding glutamic acid-rich-like protein 3 OS	OPHHA	4
2192	4.04	4.05	44.65	5.68	5.68	tr|V8NFN1|V8NFN1_OPHHA	Double-stranded RNA-specific adenosine deaminase (Fragm	OPHHA	2
2193	4.04	4.04	41.18	6.933	3.466	tr|V8NML6|V8NML6_OPHHA	Protein phosphatase 1 regulatory subunit 37 (Fragment)	OPHHA	2
2194	4.04	4.04	46.2	3.566	3.566	tr|V8P5A0|V8P5A0_OPHHA	RNA polymerase-associated protein RTF1-like protein (Fr	OPHHA	2

2195	4.04	4.04	23.44	1.997	1.997	tr|V8P0M5|V8P0M5_OPHHA ATP-binding cassette sub-family A member 5 (Fragment) O	OPHHA	2
2196	4.04	4.04	36.9	8.2	5.467	tr|V8NYG1|V8NYG1_OPHHA Chitinase-3-like protein 2 (Fragment) OS=Ophiophagus ha	OPHHA	2
2197	4.04	4.04	37.96	7.082	7.082	tr|V8NRE4|V8NRE4_OPHHA Lanosterol 14-alpha demethylase OS=Ophiophagus hannah G	OPHHA	2
2198	4.04	4.04	33.15	8.625	8.625	tr|V8PEW3|V8PEW3_OPHHA Bifunctional arginine demethylase and lysyl-hydroxylase	OPHHA	2
2199	4.04	4.04	39.51	25.93	25.93	tr|V8P4Z9|V8P4Z9_OPHHA CB1 cannabinoid receptor-interacting protein 1 (Fragmen	OPHHA	2
2200	4.04	4.04	64.41	22.88	22.88	tr|V8N2P0|V8N2P0_OPHHA Talin-1 (Fragment) OS=Ophiophagus hannah GN=TLN1 PE=4 S	OPHHA	2
2201	4.03	4.1	19.44	6.771	5.035	tr|V8NWR2|V8NWR2_OPHHA Sodium-coupled neutral amino acid transporter 3 (Fragme	OPHHA	2
2202	4.03	4.09	65.1	11.76	11.76	tr|V8N7C9|V8N7C9_OPHHA Acyl-CoA dehydrogenase family member 11 (Fragment) OS=O	OPHHA	3
2203	4.03	4.04	66.49	10.47	10.47	tr|V8PFA6|V8PFA6_OPHHA Calcyphosin-like protein OS=Ophiophagus hannah GN=Capsl	OPHHA	2
2204	4.03	4.03	49.96	1.806	1.806	tr|V8PFL0|V8PFL0_OPHHA DNA mismatch repair protein Msh6 (Fragment) OS=Ophiopha	OPHHA	2
2205	4.03	4.03	36.67	2.875	2.875	tr|V8NZV8|V8NZV8_OPHHA Uncharacterized protein (Fragment) OS=Ophiophagus hanna	OPHHA	2
2206	4.03	4.03	55.57	7.006	3.822	tr|V8PC10|V8PC10_OPHHA Mitochondrial intermediate peptidase (Fragment) OS=Ophi	OPHHA	2
2207	4.03	4.03	29.17	5.304	5.304	tr|V8PDS8|V8PDS8_OPHHA Protein unc-93-like B1 OS=Ophiophagus hannah GN=Unc93b1	OPHHA	2
2208	4.03	4.03	47.18	5.636	3.704	tr|V8NQH4|V8NQH4_OPHHA Complement component C7 OS=Ophiophagus hannah GN=C7 PE=	OPHHA	2
2209	4.03	4.03	48.49	9.799	5.779	tr|V8P639|V8P639_OPHHA Mitochondrial ribonuclease P protein 1 (Fragment) OS=Op	OPHHA	2
2210	4.03	4.03	37.42	3.576	3.576	tr|V8NC09|V8NC09_OPHHA Uncharacterized protein (Fragment) OS=Ophiophagus hanna	OPHHA	2
2211	4.03	4.03	52.04	6.122	6.122	tr|V8NGV4|V8NGV4_OPHHA E3 ubiquitin-protein ligase TRIM11 (Fragment) OS=Ophiop	OPHHA	2
2211	0	4.02	36.58	0.5333	0.5333	tr|V8NLW4|V8NLW4_OPHHA E3 ubiquitin-protein ligase TRIM11 (Fragment) OS=Ophiop	OPHHA	2
2212	4.03	4.03	43.67	6.667	6.667	tr|V8NLQ9|V8NLQ9_OPHHA Putative phospholipase B-like 2 (Fragment) OS=Ophiophag	OPHHA	2
2213	4.02	6.37	56.15	13.13	10.34	tr|V8N6F0|V8N6F0_OPHHA Cytochrome protein (Fragment) OS=Ophiophagus hannah GN=	OPHHA	4
2214	4.02	4.02	39.76	4.991	4.991	tr|V8P6D9|V8P6D9_OPHHA Protein FAM55C (Fragment) OS=Ophiophagus hannah GN=FAM5	OPHHA	2
2215	4.02	4.02	40.57	3.872	3.872	tr|V8P5Q0|V8P5Q0_OPHHA Exosome component 10 (Fragment) OS=Ophiophagus hannah G	OPHHA	2
2216	4.02	4.02	33.92	5.765	5.765	tr|V8PGY9|V8PGY9_OPHHA Testis-specific Y-encoded-like protein 2 (Fragment) OS=	OPHHA	2
2217	4.02	4.02	49.61	9.819	9.819	tr|V8P8K1|V8P8K1_OPHHA Legumain OS=Ophiophagus hannah GN=Lgmn PE=4 SV=1	OPHHA	2
2218	4.02	4.02	84.31	28.43	28.43	tr|V8P3N6|V8P3N6_OPHHA PHD finger-like domain-containing protein 5A OS=Ophioph	OPHHA	2
2219	4.02	4.02	28.3	18.87	18.87	tr|V8P983|V8P983_OPHHA Solute carrier family 2, facilitated glucose transporte	OPHHA	2
2220	4.02	4.02	23.04	10.43	7.391	tr|V8NVP4|V8NVP4_OPHHA Heme-binding protein 1 (Fragment) OS=Ophiophagus hannah	OPHHA	2
2221	4.01	6.02	30	12.08	12.08	tr|V8N6P9|V8N6P9_OPHHA Cytochrome protein (Fragment) OS=Ophiophagus hannah GN=	OPHHA	4
2222	4.01	4.01	59.01	6.832	6.832	tr|V8NSY8|V8NSY8_OPHHA Protein arginine N-methyltransferase 5 OS=Ophiophagus h	OPHHA	3
2223	4.01	4.01	45.99	7.595	7.595	tr|V8N9L3|V8N9L3_OPHHA Charged multivesicular body protein 2a (Fragment) OS=Op	OPHHA	2
2224	4.01	4.01	68.67	14	14	tr|V8NCT4|V8NCT4_OPHHA Uncharacterized protein (Fragment) OS=Ophiophagus hanna	OPHHA	2
2225	4.01	4.01	34.71	5.785	5.785	tr|V8P824|V8P824_OPHHA S-phase kinase-associated protein 1 (Fragment) OS=Ophio	OPHHA	2
2226	4.01	4.01	52.26	10.55	10.55	tr|V8N706|V8N706_OPHHA GDP-fucose protein O-fucosyltransferase 1 (Fragment) OS	OPHHA	2
2227	4.01	4.01	36	13.33	13.33	tr|V8PCV9|V8PCV9_OPHHA CDGSH iron-sulfur domain-containing protein 2 OS=Ophiop	OPHHA	2
2228	4.01	4.01	62.07	34.48	34.48	tr|V8NWY6|V8NWY6_OPHHA Uncharacterized protein (Fragment) OS=Ophiophagus hanna	OPHHA	2
2229	4	45.82	72.68	59.42	59.42	tr|V8NV61|V8NV61_OPHHA Actin, alpha skeletal muscle OS=Ophiophagus hannah GN=A	OPHHA	34
2230	4	16.56	93.75	55.56	47.92	tr|V8N322|V8N322_OPHHA Uncharacterized protein (Fragment) OS=Ophiophagus hanna	OPHHA	8
2231	4	7.49	49.12	18.25	18.25	tr|V8NFQ3|V8NFQ3_OPHHA Ras-related protein Ral-A (Fragment) OS=Ophiophagus han	OPHHA	4
2232	4	7.08	42.89	7.953	5.606	tr|V8PBJ4|V8PBJ4_OPHHA ATP-dependent 6-phosphofructokinase OS=Ophiophagus hann	OPHHA	3
2233	4	4.36	41.5	19.39	12.93	tr|V8P726|V8P726_OPHHA Brefeldin A-inhibited guanine nucleotide-exchange prote	OPHHA	3
2234	4	4.01	36.21	3.767	3.767	tr|V8PG04|V8PG04_OPHHA Activating transcription factor 7-interacting protein 1	OPHHA	2
2235	4	4.01	49.56	11.95	11.95	tr|V8PED4|V8PED4_OPHHA RING finger protein (Fragment) OS=Ophiophagus hannah GN	OPHHA	2
2236	4	4	53.95	5.643	5.643	tr|V8P5X9|V8P5X9_OPHHA Unconventional prefoldin RPB5 interactor (Fragment) OS=	OPHHA	2
2237	4	4	34.8	3.937	3.937	tr|V8NVW5|V8NVW5_OPHHA Receptor-type tyrosine-protein phosphatase epsilon (Fra	OPHHA	2
2238	4	4	38.68	4.054	4.054	tr|V8PJ62|V8PJ62_OPHHA Kelch-like ECH-associated protein 1 (Fragment) OS=Ophio	OPHHA	2
2239	4	4	39.51	4.72	4.72	tr|V8NU79|V8NU79_OPHHA Complement factor I OS=Ophiophagus hannah GN=CFI PE=3 S	OPHHA	5
2240	4	4	47.44	9.375	6.534	tr|V8P669|V8P669_OPHHA Leucine carboxyl methyltransferase 1 (Fragment) OS=Ophi	OPHHA	2
2241	4	4	55.05	9.121	9.121	tr|V8NXF7|V8NXF7_OPHHA Alpha-(1,3)-fucosyltransferase 11 (Fragment) OS=Ophioph	OPHHA	2
2242	4	4	31.41	4.704	4.704	tr|V8N5A8|V8N5A8_OPHHA Alpha-tectorin (Fragment) OS=Ophiophagus hannah GN=Tect	OPHHA	2
2243	4	4	51.97	9.319	9.319	tr|V8NRR1|V8NRR1_OPHHA G protein-coupled receptor kinase 5 (Fragment) OS=Ophio	OPHHA	2
2243	0	2.02	63.3	2.087	2.087	tr|V8PEN5|V8PEN5_OPHHA G protein-coupled receptor kinase 6 OS=Ophiophagus hann	OPHHA	1
2244	4	4	72.83	14.45	14.45	tr|V8NNG0|V8NNG0_OPHHA Acyl-CoA-binding domain-containing protein 6 (Fragment)	OPHHA	2
2245	4	4	42.24	13.79	9.914	tr|V8PG69|V8PG69_OPHHA Sel1 repeat-containing protein 1 OS=Ophiophagus hannah	OPHHA	2
2246	4	4	50.69	8.621	8.621	tr|V8PB16|V8PB16_OPHHA Syntaxin-16 (Fragment) OS=Ophiophagus hannah GN=STX16 P	OPHHA	2
2247	4	4	48.28	7.837	7.837	tr|V8P3J9|V8P3J9_OPHHA Proline-rich AKT1 substrate 1 (Fragment) OS=Ophiophagus	OPHHA	2
2248	4	4	53.26	13.79	13.79	tr|V8NPC8|V8NPC8_OPHHA Uncharacterized protein (Fragment) OS=Ophiophagus hanna	OPHHA	2
2249	4	4	38.91	3.754	3.754	tr|V8N913|V8N913_OPHHA Retinol dehydrogenase 14 (Fragment) OS=Ophiophagus hann	OPHHA	2
2250	4	4	65.27	21.56	21.56	tr|V8NAI5|V8NAI5_OPHHA CaiB/baiF CoA-transferase family protein C7orf10 (Fragm	OPHHA	2
2251	4	4	47.39	7.826	7.826	tr|V8N5R0|V8N5R0_OPHHA Sulfotransferase OS=Ophiophagus hannah GN=Sult6b1 PE=3	OPHHA	2
2252	4	4	63.7	18.52	18.52	tr|V8NGW7|V8NGW7_OPHHA COMM domain-containing protein 1 (Fragment) OS=Ophiopha	OPHHA	2
2253	4	4	67.39	9.42	9.42	tr|V8NZL2|V8NZL2_OPHHA Emerin OS=Ophiophagus hannah GN=Emd PE=4 SV=1	OPHHA	2
2254	4	4	50.52	10.1	10.1	tr|V8NZ49|V8NZ49_OPHHA GDP-mannose 4,6 dehydratase (Fragment) OS=Ophiophagus h	OPHHA	2
2255	4	4	37.92	8.257	8.257	tr|V8NLQ7|V8NLQ7_OPHHA Probable cytosolic iron-sulfur protein assembly protein	OPHHA	2
2256	4	4	88.03	16.24	16.24	tr|V8N983|V8N983_OPHHA RNA-binding protein 8A OS=Ophiophagus hannah GN=RBM8A P	OPHHA	3
2257	4	4	86.36	36.36	36.36	tr|V8N3J1|V8N3J1_OPHHA Uncharacterized protein (Fragment) OS=Ophiophagus hanna	OPHHA	2
2258	4	4	28.28	11.48	11.48	tr|V8N812|V8N812_OPHHA 2-acylglycerol O-acyltransferase 2-A (Fragment) OS=Ophi	OPHHA	2
2259	4	4	59	23	23	tr|V8P5M7|V8P5M7_OPHHA Uncharacterized protein (Fragment) OS=Ophiophagus hanna	OPHHA	2
2260	4	4	75.49	25.49	25.49	tr|V8P2W9|V8P2W9_OPHHA Ribonuclease OS=Ophiophagus hannah GN=HRSP12 PE=4 SV=1	OPHHA	4
2261	4	4	14.57	4.933	4.933	tr|V8NGJ0|V8NGJ0_OPHHA Galactocerebrosidase OS=Ophiophagus hannah GN=galc PE=4	OPHHA	2
2262	4	4	31.42	13.27	13.27	tr|V8PEB6|V8PEB6_OPHHA Guanylate kinase OS=Ophiophagus hannah GN=GUK1 PE=4 SV=	OPHHA	2
2263	4	4	11.78	6.667	6.667	tr|V8P9K9|V8P9K9_OPHHA Serine incorporator 1 OS=Ophiophagus hannah GN=SERINC1	OPHHA	2
2264	4	4	19.38	7.752	7.752	tr|V8NK08|V8NK08_OPHHA Ubiquitin-associated protein 1 (Fragment) OS=Ophiophagu	OPHHA	2
2265	4	4	48.35	27.47	27.47	tr|V8NGK1|V8NGK1_OPHHA Leucine-rich repeat-containing protein 16A (Fragment) O	OPHHA	2
2266	4	4	39.67	22.31	22.31	tr|V8N8Q1|V8N8Q1_OPHHA Uncharacterized protein (Fragment) OS=Ophiophagus hanna	OPHHA	2
2267	4	4	53.91	25.22	25.22	tr|V8N5B6|V8N5B6_OPHHA Tax1-binding protein 3 (Fragment) OS=Ophiophagus hannah	OPHHA	2
2268	4	4	26.42	18.87	18.87	tr|V8NKM1|V8NKM1_OPHHA U1 small nuclear ribonucleoprotein C OS=Ophiophagus han	OPHHA	2
2269	4	4	44.7	24.24	24.24	tr|V8NFR9|V8NFR9_OPHHA Ras GTPase-activating-like protein IQGAP1 (Fragment) OS	OPHHA	2
2270	4	4	36.54	21.15	21.15	tr|V8N5F2|V8N5F2_OPHHA Dysferlin OS=Ophiophagus hannah GN=DYSF PE=4 SV=1	OPHHA	2
2271	4	4	74.36	39.74	39.74	tr|V8N346|V8N346_OPHHA Uncharacterized protein (Fragment) OS=Ophiophagus hanna	OPHHA	2
2272	4	4	13.74	5.852	5.852	tr|V8P6G4|V8P6G4_OPHHA Nuclear factor 1 B-type (Fragment) OS=Ophiophagus hanna	OPHHA	2
2273	4	4	45.16	34.41	34.41	tr|V8P1Q8|V8P1Q8_OPHHA NADH dehydrogenase [ubiquinone] 1 alpha subcomplex subu	OPHHA	4
2274	4	4	38.14	38.14	38.14	tr|V8NG55|V8NG55_OPHHA Solute carrier family 25 member 46 (Fragment) OS=Ophiop	OPHHA	2
2275	3.99	3.99	41.93	2.41	2.41	tr|V8NXU8|V8NXU8_OPHHA Phospholipase A2 (Fragment) OS=Ophiophagus hannah GN=PL	OPHHA	2
2276	3.98	3.98	49.59	4.772	4.772	tr|V8P3F8|V8P3F8_OPHHA Rab11 family-interacting protein 3 (Fragment) OS=Ophiop	OPHHA	2
2277	3.98	3.98	39.72	6.197	6.197	tr|V8P1J7|V8P1J7_OPHHA Sparc (Fragment) OS=Ophiophagus hannah GN=SPARC PE=4 SV	OPHHA	2
2278	3.96	4.18	57.67	7.379	6.214	tr|V8NIG0|V8NIG0_OPHHA Methionyl-tRNA synthetase, mitochondrial (Fragment) OS=	OPHHA	2
2279	3.96	4.03	43.55	9.032	9.032	tr|V8NFX4|V8NFX4_OPHHA Plasma membrane calcium-transporting ATPase 1 (Fragment	OPHHA	2
2280	3.96	3.96	66.88	6.818	6.818	tr|V8P9A6|V8P9A6_OPHHA Uncharacterized protein (Fragment) OS=Ophiophagus hanna	OPHHA	2
2281	3.96	3.96	43.9	10.84	9.214	tr|V8PE19|V8PE19_OPHHA Myeloid-associated differentiation marker-like protein	OPHHA	2
2282	3.95	8.69	42.46	10.61	8.939	tr|V8N763|V8N763_OPHHA Cytochrome protein (Fragment) OS=Ophiophagus hannah GN=	OPHHA	4
2283	3.95	3.95	44.45	3.095	1.591	tr|V8NH22|V8NH22_OPHHA Zinc finger protein (Fragment) OS=Ophiophagus hannah GN	OPHHA	4
2284	3.95	3.95	72.91	16.71	8.934	tr|V8NUH4|V8NUH4_OPHHA Uncharacterized protein (Fragment) OS=Ophiophagus hanna	OPHHA	2
2285	3.95	3.95	44.16	8.262	8.262	tr|V8P086|V8P086_OPHHA Tensin (Fragment) OS=Ophiophagus hannah GN=TNS PE=4 SV=	OPHHA	2
2286	3.94	3.94	44.33	32.99	32.99	tr|V8NQN8|V8NQN8_OPHHA Acetyl-CoA carboxylase (Fragment) OS=Ophiophagus hannah	OPHHA	3
2287	3.93	3.93	43.85	5.426	2.879	tr|V8P3G1|V8P3G1_OPHHA Protein unc-45-like A (Fragment) OS=Ophiophagus hannah	OPHHA	2
2288	3.93	3.93	45.28	8.399	2.887	tr|V8NMZ1|V8NMZ1_OPHHA High affinity nerve growth factor receptor (Fragment) O	OPHHA	2

2289	3.93	3.93	35.04	7.682	3.908	tr|V8NQT5|V8NQT5_OPHHA	Zinc finger CCCH domain-containing protein 14 (Fragment	OPHHA	2
2290	3.93	3.93	44.26	9.605	4.331	tr|V8NJE7|V8NJE7_OPHHA	Putative leucyl-tRNA synthetase, mitochondrial (Fragmen	OPHHA	2
2291	3.93	3.93	34.44	5.74	5.74	tr|V8NWQ7|V8NWQ7_OPHHA	Glutathione S-transferase theta-1 OS=Ophiophagus hannah	OPHHA	2
2292	3.93	3.93	42.27	10.5	7.58	tr|V8NJX8|V8NJX8_OPHHA	HEAT repeat-containing protein 7A (Fragment) OS=Ophioph	OPHHA	2
2293	3.92	11.53	56.08	17.06	12.15	tr|V8NUW6|V8NUW6_OPHHA	Septin-8 (Fragment) OS=Ophiophagus hannah GN=SEPT8 PE=3	OPHHA	5
2294	3.92	3.92	64.58	11.6	8.777	tr|V8P876|V8P876_OPHHA	Abhydrolase domain-containing protein 14A (Fragment) OS	OPHHA	2
2295	3.92	3.92	64.65	24.24	24.24	tr|V8P6M2|V8P6M2_OPHHA	Uncharacterized protein (Fragment) OS=Ophiophagus hanna	OPHHA	2
2296	3.91	3.91	48.45	2.966	2.401	tr|V8NJ21|V8NJ21_OPHHA	Collagen alpha-3(IV) chain (Fragment) OS=Ophiophagus ha	OPHHA	2
2297	3.91	3.91	50.1	6.114	4.536	tr|V8NMY7|V8NMY7_OPHHA	Scaffold attachment factor B2 (Fragment) OS=Ophiophagus	OPHHA	2
2298	3.9	3.97	40.83	2.823	2.823	tr|V8NHW6|V8NHW6_OPHHA	Coiled-coil domain-containing protein (Fragment) OS=Oph	OPHHA	2
2299	3.9	3.9	65.91	18.75	14.2	tr|V8NBM7|V8NBM7_OPHHA	Tubulin polymerization-promoting protein family member	OPHHA	2
2300	3.89	3.89	47.45	6.841	5.677	tr|V8P8F1|V8P8F1_OPHHA	X-ray repair cross-complementing protein 5 (Fragment) O	OPHHA	3
2301	3.88	3.88	39.07	4.055	3.066	tr|V8P195|V8P195_OPHHA	Alanyl-tRNA synthetase, mitochondrial (Fragment) OS=Oph	OPHHA	2
2302	3.88	3.88	66.83	7.805	7.805	tr|V8N882|V8N882_OPHHA	Structural maintenance of chromosomes flexible hinge do	OPHHA	2
2303	3.88	3.88	58.05	23.41	20	tr|V8N4S1|V8N4S1_OPHHA	Basement membrane-specific heparan sulfate proteoglycan	OPHHA	3
2304	3.88	3.88	28.07	14.47	11.4	tr|V8PG76|V8PG76_OPHHA	WASH complex subunit CCDC53 (Fragment) OS=Ophiophagus h	OPHHA	2
2305	3.88	3.88	56.15	34.62	34.62	tr|V8NCJ1|V8NCJ1_OPHHA	tRNA methyltransferase-like protein OS=Ophiophagus hann	OPHHA	4
2306	3.87	3.87	35.76	21.21	21.21	tr|V8N5W7|V8N5W7_OPHHA	Complement factor H (Fragment) OS=Ophiophagus hannah GN	OPHHA	3
2307	3.86	3.86	33.47	1.902	1.902	tr|V8NQD8|V8NQD8_OPHHA	DmX-like protein 2 (Fragment) OS=Ophiophagus hannah GN=	OPHHA	4
2308	3.86	3.86	28.77	5.936	3.653	tr|V8PDR4|V8PDR4_OPHHA	Ubiquitin-conjugating enzyme E2 O (Fragment) OS=Ophioph	OPHHA	2
2309	3.86	3.86	33.12	14.2	8.202	tr|V8P0C4|V8P0C4_OPHHA	Protein VAC14-like protein (Fragment) OS=Ophiophagus ha	OPHHA	2
2310	3.85	4.11	29.89	4.112	2.307	tr|V8NN43|V8NN43_OPHHA	Receptor protein-tyrosine kinase (Fragment) OS=Ophiopha	OPHHA	3
2311	3.85	3.85	58.53	9.118	9.118	tr|V8NP20|V8NP20_OPHHA	Serine/threonine-protein kinase 38 (Fragment) OS=Ophiop	OPHHA	2
2312	3.85	3.85	40	12.5	7.5	tr|V8PH52|V8PH52_OPHHA	UV excision repair protein RAD23-like A (Fragment) OS=O	OPHHA	2
2313	3.85	3.85	64.06	13.82	13.82	tr|V8PEQ0|V8PEQ0_OPHHA	Guanine nucleotide-binding protein subunit gamma (Fragm	OPHHA	2
2314	3.85	3.85	45.45	12	9.455	tr|V8P5D1|V8P5D1_OPHHA	28S ribosomal protein S30, mitochondrial (Fragment) OS=	OPHHA	2
2315	3.85	3.85	28.19	11.17	7.713	tr|V8NRX7|V8NRX7_OPHHA	Ubiquitin-conjugating enzyme E2 J1 (Fragment) OS=Ophiop	OPHHA	2
2316	3.85	3.85	31.66	6.533	6.533	tr|V8NN17|V8NN17_OPHHA	RNA-binding motif, single-stranded-interacting protein	OPHHA	2
2317	3.84	3.84	32.86	21.43	21.43	tr|V8N980|V8N980_OPHHA	Translationally-controlled tumor protein-like protein (	OPHHA	4
2318	3.83	8.52	38.56	16.56	11.11	tr|V8NI58|V8NI58_OPHHA	Phosphatidylinositol-binding clathrin assembly protein	OPHHA	6
2319	3.83	3.83	46.38	26.09	19.32	tr|V8NJU7|V8NJU7_OPHHA	Exportin-1 (Fragment) OS=Ophiophagus hannah GN=Xpo1 PE=	OPHHA	3
2320	3.83	3.83	33.58	17.52	12.41	tr|V8PBJ7|V8PBJ7_OPHHA	Prefoldin subunit 5 OS=Ophiophagus hannah GN=PFDN5 PE=4	OPHHA	2
2321	3.82	4.15	72.73	31.82	18.18	tr|V8N5Z1|V8N5Z1_OPHHA	Tropomyosin alpha-3 chain (Fragment) OS=Ophiophagus han	OPHHA	2
2322	3.82	3.82	44.84	5.952	5.952	tr|V8PHL0|V8PHL0_OPHHA	SH3 and PX domain-containing protein 2B (Fragment) OS=O	OPHHA	3
2323	3.82	3.82	15.94	2.772	2.079	tr|V8PHC6|V8PHC6_OPHHA	Histone deacetylase 5 (Fragment) OS=Ophiophagus hannah	OPHHA	2
2324	3.81	3.81	38.45	4.193	2.319	tr|V8NEE3|V8NEE3_OPHHA	UHRF1-binding protein 1-like protein (Fragment) OS=Ophi	OPHHA	2
2325	3.81	3.81	56.02	13.16	12.78	tr|V8PD74|V8PD74_OPHHA	Myelin P2 protein OS=Ophiophagus hannah GN=PMP2 PE=3 SV	OPHHA	3
2326	3.81	3.81	28.34	5.263	5.263	tr|V8P7C8|V8P7C8_OPHHA	Gamma-glutamyltransferase 5 (Fragment) OS=Ophiophagus h	OPHHA	2
2327	3.81	3.81	66.67	22.22	22.22	tr|V8PGE8|V8PGE8_OPHHA	Annexin (Fragment) OS=Ophiophagus hannah GN=L345_00743	OPHHA	2
2328	3.81	3.81	20.29	10.27	5.623	tr|V8P7Y5|V8P7Y5_OPHHA	CAAX prenyl protease 1-like protein OS=Ophiophagus hann	OPHHA	2
2329	3.81	3.81	22.7	11.49	8.908	tr|V8NEP3|V8NEP3_OPHHA	Bifunctional ATP-dependent dihydroxyacetone kinase/FAD-	OPHHA	2
2330	3.8	3.81	60.54	15.65	15.65	tr|V8PJ80|V8PJ80_OPHHA	Glia maturation factor beta (Fragment) OS=Ophiophagus h	OPHHA	3
2331	3.8	3.8	49.3	5.578	2.789	tr|V8NUX1|V8NUX1_OPHHA	Paired amphipathic helix protein Sin3a OS=Ophiophagus h	OPHHA	3
2332	3.8	3.8	40	6.024	6.024	tr|V8NKZ2|V8NKZ2_OPHHA	Protein misato-like 1 (Fragment) OS=Ophiophagus hannah	OPHHA	2
2333	3.8	3.8	44.44	12.57	9.064	tr|V8PD71|V8PD71_OPHHA	Putative transferase CAF17, mitochondrial (Fragment) OS	OPHHA	3
2334	3.8	3.8	65.63	21.88	21.88	tr|V8NY33|V8NY33_OPHHA	Protein transport protein Sec61 subunit beta OS=Ophioph	OPHHA	2
2335	3.77	3.77	56.17	6.168	4.068	tr|V8PGB0|V8PGB0_OPHHA	Putative RNA-binding protein 15 (Fragment) OS=Ophiophag	OPHHA	2
2336	3.77	3.77	33.14	4.551	2.45	tr|V8P8P8|V8P8P8_OPHHA	Ubiquitin carboxyl-terminal hydrolase 4 (Fragment) OS=O	OPHHA	2
2337	3.77	3.77	49.39	7.085	7.085	tr|V8NYS4|V8NYS4_OPHHA	5'-nucleotidase domain-containing protein 2 (Fragment)	OPHHA	3
2338	3.77	3.77	34.52	13.1	13.1	tr|V8P649|V8P649_OPHHA	Eukaryotic translation initiation factor 4E type 3 (Fra	OPHHA	2
2339	3.76	3.76	46.44	3.541	1.959	tr|V8NAK4|V8NAK4_OPHHA	A-kinase anchor protein 12 (Fragment) OS=Ophiophagus ha	OPHHA	4
2340	3.76	3.76	57.23	30.82	18.87	tr|V8NPK8|V8NPK8_OPHHA	Glutathione peroxidase OS=Ophiophagus hannah GN=GPX4 PE	OPHHA	3
2341	3.75	4	52.3	7.503	3.024	tr|V8NLS6|V8NLS6_OPHHA	THO complex subunit 2 (Fragment) OS=Ophiophagus hannah	OPHHA	2
2342	3.74	3.77	31.14	2.958	2.958	tr|V8PC56|V8PC56_OPHHA	Chloride channel protein (Fragment) OS=Ophiophagus hann	OPHHA	2
2343	3.74	3.74	37.97	17.11	17.11	tr|V8PFC3|V8PFC3_OPHHA	Uncharacterized protein (Fragment) OS=Ophiophagus hanna	OPHHA	2
2344	3.74	3.74	34.2	20.78	20.78	tr|V8P5Y0|V8P5Y0_OPHHA	Replication protein A 32 kDa subunit OS=Ophiophagus han	OPHHA	3
2345	3.74	3.74	22.95	9.426	9.426	tr|V8NJB2|V8NJB2_OPHHA	2-oxoisovalerate dehydrogenase subunit beta, mitochondr	OPHHA	2
2346	3.73	3.73	39.77	16.37	11.11	tr|V8NA97|V8NA97_OPHHA	Cleavage and polyadenylation specificity factor subunit	OPHHA	2
2347	3.72	3.72	49.38	25	12.5	tr|V8PGM6|V8PGM6_OPHHA	Uncharacterized protein (Fragment) OS=Ophiophagus hanna	OPHHA	3
2348	3.72	3.72	29.45	20.25	20.25	tr|V8P9D3|V8P9D3_OPHHA	Protein tyrosine phosphatase type IVA 2 (Fragment) OS=O	OPHHA	2
2349	3.71	3.71	42.13	6.742	6.742	tr|V8PBS6|V8PBS6_OPHHA	Synaptic vesicle membrane protein VAT-1-like protein (F	OPHHA	2
2350	3.71	3.71	41.32	21.56	21.56	tr|V8PGE5|V8PGE5_OPHHA	Uncharacterized protein OS=Ophiophagus hannah GN=L345_0	OPHHA	3
2351	3.7	3.74	31.67	20	16.11	tr|V8NH05|V8NH05_OPHHA	Peripheral plasma membrane protein CASK (Fragment) OS=O	OPHHA	2
2352	3.7	3.7	47.06	9.02	9.02	tr|V8NW40|V8NW40_OPHHA	Creatine kinase S-type, mitochondrial (Fragment) OS=Oph	OPHHA	2
2353	3.7	3.7	32.59	6.685	6.685	tr|V8PG84|V8PG84_OPHHA	Uncharacterized protein OS=Ophiophagus hannah GN=L345_0	OPHHA	2
2354	3.7	3.7	33.13	22.5	18.13	tr|V8NTB7|V8NTB7_OPHHA	Coatomer subunit alpha (Fragment) OS=Ophiophagus hannah	OPHHA	2
2355	3.7	3.7	32.56	16.86	16.86	tr|V8NXZ5|V8NXZ5_OPHHA	Ubiquitin-conjugating enzyme E2 D2 OS=Ophiophagus hanna	OPHHA	3
2355	0	3.7	26.43	20.71	20.71	tr|V8PBB6|V8PBB6_OPHHA	Ubiquitin-conjugating enzyme E2 D3 (Fragment) OS=Ophiop	OPHHA	3
2355	0	1.55	19.59	12.16	12.16	tr|V8NR52|V8NR52_OPHHA	Ubiquitin-conjugating enzyme E2 D1 (Fragment) OS=Ophiop	OPHHA	2
2356	3.7	3.7	13.12	8.597	8.597	tr|V8N9Q2|V8N9Q2_OPHHA	Uncharacterized protein (Fragment) OS=Ophiophagus hanna	OPHHA	2
2357	3.69	3.69	38.76	26.36	26.36	tr|V8NMT6|V8NMT6_OPHHA	AP-2 complex subunit sigma OS=Ophiophagus hannah GN=AP2	OPHHA	3
2358	3.68	3.71	45.4	11.4	2.586	tr|V8P5W0|V8P5W0_OPHHA	Phosphoinositide phospholipase C OS=Ophiophagus hannah	OPHHA	2
2359	3.68	3.7	58.06	29.03	29.03	tr|V8P912|V8P912_OPHHA	Glycophorin-C OS=Ophiophagus hannah GN=GYPC PE=4 SV=1	OPHHA	2
2360	3.68	3.68	37.83	1.549	1.549	tr|V8PFA9|V8PFA9_OPHHA	Tyrosine-protein kinase receptor (Fragment) OS=Ophiopha	OPHHA	2
2361	3.68	3.68	49.38	7.009	3.427	tr|V8P7T9|V8P7T9_OPHHA	Sulfhydryl oxidase OS=Ophiophagus hannah GN=QSOX1 PE=4	OPHHA	2
2362	3.68	3.68	19.48	3.177	2.258	tr|V8P1N0|V8P1N0_OPHHA	Niemann-Pick C1 protein (Fragment) OS=Ophiophagus hanna	OPHHA	3
2363	3.68	3.68	73.87	11.71	11.71	tr|V8P7G5|V8P7G5_OPHHA	NADH dehydrogenase [ubiquinone] 1 alpha subcomplex subu	OPHHA	2
2364	3.68	3.68	88.17	24.73	24.73	tr|V8NMJ7|V8NMJ7_OPHHA	28S ribosomal protein S27, mitochondrial (Fragment) OS=	OPHHA	2
2365	3.68	3.68	50.47	23.36	23.36	tr|V8P736|V8P736_OPHHA	28S ribosomal protein S36, mitochondrial OS=Ophiophagus	OPHHA	3
2366	3.67	3.83	19	5.156	4.342	tr|V8P3D0|V8P3D0_OPHHA	Tenascin (Fragment) OS=Ophiophagus hannah GN=Tnc PE=4 S	OPHHA	2
2367	3.67	3.71	43.71	5.222	5.222	tr|V8NWY7|V8NWY7_OPHHA	25-hydroxycholesterol 7-alpha-hydroxylase (Fragment) OS	OPHHA	2
2368	3.67	3.67	49.17	25.97	15.47	tr|V8NPX1|V8NPX1_OPHHA	Unc-119-like B protein (Fragment) OS=Ophiophagus hannah	OPHHA	2
2369	3.67	3.67	68.46	21.54	16.15	tr|V8NPN2|V8NPN2_OPHHA	Carnitine O-acetyltransferase (Fragment) OS=Ophiophagus	OPHHA	3
2370	3.67	3.67	47.3	17.57	17.57	tr|V8N571|V8N571_OPHHA	Putative ATP-dependent RNA helicase DDX58 (Fragment) OS	OPHHA	2
2371	3.66	3.66	68.24	24.89	20.6	tr|V8NX85|V8NX85_OPHHA	Lysophospholipase-like protein 1 (Fragment) OS=Ophiopha	OPHHA	3
2372	3.66	3.66	84.55	18.7	18.7	tr|V8NIE1|V8NIE1_OPHHA	Uncharacterized protein (Fragment) OS=Ophiophagus hanna	OPHHA	2
2373	3.66	3.66	40.2	37.25	29.41	tr|V8NC66|V8NC66_OPHHA	AP-3 complex subunit sigma-1 (Fragment) OS=Ophiophagus	OPHHA	3
2374	3.65	3.65	63.43	7.025	7.025	tr|V8PHI8|V8PHI8_OPHHA	Zinc finger protein OS=Ophiophagus hannah GN=ZNF622 PE=	OPHHA	2
2375	3.65	3.65	36.93	11.85	11.85	tr|V8P1K7|V8P1K7_OPHHA	Phosphoinositide 3-kinase adapter protein 1 (Fragment)	OPHHA	2
2376	3.64	3.64	70.79	31.46	31.46	tr|V8NZE2|V8NZE2_OPHHA	WD repeat-containing protein 70 (Fragment) OS=Ophiophag	OPHHA	2
2377	3.63	3.63	46.47	13.46	7.372	tr|V8P1A1|V8P1A1_OPHHA	Beta-1,4-galactosyltransferase 4 (Fragment) OS=Ophiopha	OPHHA	2
2378	3.63	3.63	47.41	21.48	21.48	tr|V8NG83|V8NG83_OPHHA	Lipoma-preferred partner-like protein (Fragment) OS=Oph	OPHHA	2
2379	3.63	3.63	69.72	48.62	23.85	tr|V8NX51|V8NX51_OPHHA	Dihydrofolate reductase (Fragment) OS=Ophiophagus hanna	OPHHA	2
2380	3.62	3.62	42.88	6.47	4.806	tr|V8PHW1|V8PHW1_OPHHA	Ecotropic viral integration site 5 protein-like protein	OPHHA	2
2381	3.61	3.61	33.43	2.798	1.833	tr|V8NWU0|V8NWU0_OPHHA	Protein ELYS (Fragment) OS=Ophiophagus hannah GN=AHCTF1	OPHHA	3
2382	3.61	3.61	37.28	5.013	3.856	tr|V8P7Q6|V8P7Q6_OPHHA	Rho GTPase-activating protein 26 (Fragment) OS=Ophiopha	OPHHA	2

2383	3.6	3.6	34.42	3.323	2.696	tr|V8N7W1|V8N7W1_OPHHA WD repeat-containing protein 81 (Fragment) OS=Ophiophag	OPHHA	3
2384	3.6	3.6	49.49	5.102	5.102	tr|V8NVR8|V8NVR8_OPHHA Phosphatidate phosphatase LPIN1 (Fragment) OS=Ophiophag	OPHHA	4
2385	3.6	3.6	35.41	5.253	5.253	tr|V8P684|V8P684_OPHHA Leucine-rich repeat protein SHOC-2 (Fragment) OS=Ophiop	OPHHA	2
2386	3.59	3.59	47.72	6.14	4.211	tr|V8NZ15|V8NZ15_OPHHA CCR4-NOT transcription complex subunit 10 (Fragment) OS	OPHHA	2
2387	3.59	3.59	37.24	13.51	8.408	tr|V8NKJ5|V8NKJ5_OPHHA NAD-dependent deacetylase sirtuin-2 (Fragment) OS=Ophio	OPHHA	2
2388	3.59	3.59	81.05	29.47	21.05	tr|V8NY00|V8NY00_OPHHA U6 snRNA-associated Sm-like protein LSm2 OS=Ophiophagus	OPHHA	2
2389	3.59	3.59	57.69	23.85	23.85	tr|V8N786|V8N786_OPHHA Uncharacterized protein OS=Ophiophagus hannah GN=L345_1	OPHHA	2
2390	3.58	3.58	74.21	25.79	20.81	tr|V8NW75|V8NW75_OPHHA Ras-related protein Rab-20 (Fragment) OS=Ophiophagus ha	OPHHA	3
2391	3.57	3.57	36.3	7.942	4.7	tr|V8PDK2|V8PDK2_OPHHA Drebrin (Fragment) OS=Ophiophagus hannah GN=DBN1 PE=4 S	OPHHA	2
2392	3.57	3.57	63.04	14.4	8.56	tr|V8NZ48|V8NZ48_OPHHA Syntaxin-6 (Fragment) OS=Ophiophagus hannah GN=STX6 PE=	OPHHA	2
2393	3.57	3.57	31.14	4.878	4.878	tr|V8P472|V8P472_OPHHA Phosphatidylserine synthase 1 (Fragment) OS=Ophiophagus	OPHHA	2
2394	3.57	3.57	45.51	6.667	6.667	tr|V8NXM8|V8NXM8_OPHHA Sterol-4-alpha-carboxylate 3-dehydrogenase, decarboxyla	OPHHA	2
2395	3.57	3.57	64.79	12.68	12.68	tr|V8PGD7|V8PGD7_OPHHA Ecotropic viral integration site 5 protein-like protein	OPHHA	2
2396	3.57	3.57	55.32	15.43	10.11	tr|V8NZQ3|V8NZQ3_OPHHA Trafficking protein particle complex subunit 5 (Fragmen	OPHHA	2
2397	3.57	3.57	48.37	26.8	15.69	tr|V8NX71|V8NX71_OPHHA Retinol-binding protein 4 (Fragment) OS=Ophiophagus han	OPHHA	2
2398	3.56	3.56	40.5	6.387	6.218	tr|V8PF92|V8PF92_OPHHA Phosphatidylinositol-glycan-specific phospholipase D (F	OPHHA	3
2399	3.56	3.56	66.91	21.58	12.95	tr|V8PF32|V8PF32_OPHHA Cytoglobin (Fragment) OS=Ophiophagus hannah GN=Cygb PE=	OPHHA	2
2400	3.55	3.55	55.44	12.73	4.928	tr|V8P0J6|V8P0J6_OPHHA Phosphatidylinositol 4-kinase type 2-alpha (Fragment) O	OPHHA	2
2401	3.55	3.55	51.37	10.27	10.27	tr|V8N9P4|V8N9P4_OPHHA Queuine tRNA-ribosyltransferase (Fragment) OS=Ophiophag	OPHHA	2
2402	3.55	3.55	84.13	36.51	36.51	tr|V8N2T4|V8N2T4_OPHHA Uncharacterized protein (Fragment) OS=Ophiophagus hanna	OPHHA	2
2403	3.54	4.77	43.47	6.999	6.999	tr|V8NLL1|V8NLL1_OPHHA Lysophosphatidylcholine acyltransferase 2 (Fragment) OS	OPHHA	4
2404	3.54	3.58	33.33	2.715	1.377	tr|V8NDM8|V8NDM8_OPHHA Peroxisomal proliferator-activated receptor A-interacti	OPHHA	3
2405	3.54	3.54	38.29	11.6	9.409	tr|V8N7Y1|V8N7Y1_OPHHA Beta-galactosidase-1-like protein 2 (Fragment) OS=Ophio	OPHHA	3
2406	3.54	3.54	51.3	36.52	18.26	tr|V8P6Y5|V8P6Y5_OPHHA Protein LBH (Fragment) OS=Ophiophagus hannah GN=LBH PE=	OPHHA	2
2407	3.52	3.52	53.35	4.345	1.296	tr|V8P0L6|V8P0L6_OPHHA Intersectin-2 (Fragment) OS=Ophiophagus hannah GN=Itsn2	OPHHA	2
2408	3.51	5.7	44.14	9.742	8.151	tr|V8P1C2|V8P1C2_OPHHA TATA-binding protein-associated factor 2N OS=Ophiophagu	OPHHA	3
2409	3.51	5.22	45.77	15.16	12.83	tr|V8NJY6|V8NJY6_OPHHA Long-chain fatty acid transport protein 4 (Fragment) OS	OPHHA	3
2410	3.51	3.51	12.17	12.17	12.17	tr|V8NMC1|V8NMC1_OPHHA Translocon-associated protein subunit alpha (Fragment)	OPHHA	3
2411	3.5	3.51	35.6	2.785	2.785	tr|V8PH57|V8PH57_OPHHA Neurabin-2 (Fragment) OS=Ophiophagus hannah GN=Ppp1r9b	OPHHA	2
2412	3.5	3.5	34.65	3.477	2.398	tr|V8NLF8|V8NLF8_OPHHA E3 ubiquitin-protein ligase (Fragment) OS=Ophiophagus h	OPHHA	2
2413	3.5	3.5	45.02	4.739	4.739	tr|V8PDZ7|V8PDZ7_OPHHA GRAM domain-containing protein 4 (Fragment) OS=Ophiopha	OPHHA	2
2414	3.5	3.5	52.7	12.33	5.236	tr|V8N7J5|V8N7J5_OPHHA Neuropilin-2 (Fragment) OS=Ophiophagus hannah GN=NRP2 P	OPHHA	2
2415	3.49	3.49	41.84	3.567	2.126	tr|V8NN78|V8NN78_OPHHA Gem-associated protein 5 (Fragment) OS=Ophiophagus hann	OPHHA	2
2416	3.49	3.49	30.05	6.39	6.39	tr|V8PEZ4|V8PEZ4_OPHHA Beta-glucuronidase OS=Ophiophagus hannah GN=GUSB PE=3 S	OPHHA	3
2417	3.49	3.49	77.03	25	19.59	tr|V8NEZ6|V8NEZ6_OPHHA Transmembrane and coiled-coil domain-containing protein	OPHHA	2
2418	3.48	3.48	62.16	24.32	24.32	tr|V8PEV3|V8PEV3_OPHHA Cytoglobin (Fragment) OS=Ophiophagus hannah GN=CYGB PE=	OPHHA	2
2419	3.47	5.53	45.15	14.56	5.696	tr|V8N680|V8N680_OPHHA Plectin (Fragment) OS=Ophiophagus hannah GN=PLEC PE=4 S	OPHHA	2
2420	3.47	3.47	55.08	6.581	2.861	tr|V8NMV3|V8NMV3_OPHHA tRNA (Cytosine(34)-C(5))-methyltransferase OS=Ophiophag	OPHHA	2
2421	3.47	3.47	35.62	5.961	3.428	tr|V8P2F9|V8P2F9_OPHHA Cartilage acidic protein 1 (Fragment) OS=Ophiophagus ha	OPHHA	2
2422	3.47	3.47	33.67	27.55	26.53	tr|V8NUK8|V8NUK8_OPHHA Laminin subunit alpha-5 (Fragment) OS=Ophiophagus hanna	OPHHA	3
2423	3.46	5.87	30.57	11.06	7.805	tr|V8PES6|V8PES6_OPHHA Epsin-2 OS=Ophiophagus hannah GN=Epn2 PE=4 SV=1	OPHHA	4
2424	3.44	4.1	30.18	6.852	1.631	tr|V8PA58|V8PA58_OPHHA E3 ubiquitin-protein ligase (Fragment) OS=Ophiophagus h	OPHHA	1
2425	3.44	3.44	27.12	3.211	2.1	tr|V8NN06|V8NN06_OPHHA Baculoviral IAP repeat-containing protein 6 (Fragment)	OPHHA	5
2426	3.44	3.44	80	32.67	32.67	tr|V8NU37|V8NU37_OPHHA U5 small nuclear ribonucleoprotein 40 kDa protein OS=Op	OPHHA	3
2427	3.44	3.44	51.61	31.18	31.18	tr|V8N1U1|V8N1U1_OPHHA Uncharacterized protein (Fragment) OS=Ophiophagus hanna	OPHHA	2
2428	3.42	3.42	56.88	17.43	12.84	tr|V8PHN4|V8PHN4_OPHHA Autophagy-related protein OS=Ophiophagus hannah GN=ATG1	OPHHA	2
2429	3.42	3.42	61.49	17.39	13.04	tr|V8NXW6|V8NXW6_OPHHA MOB kinase activator 2 (Fragment) OS=Ophiophagus hannah	OPHHA	3
2430	3.42	3.42	34.82	5.292	5.292	tr|V8NPW8|V8NPW8_OPHHA Protein OSCP1 (Fragment) OS=Ophiophagus hannah GN=OSCP1	OPHHA	2
2431	3.42	3.42	40.31	23.26	17.05	tr|V8NY43|V8NY43_OPHHA Brain protein 44 OS=Ophiophagus hannah GN=BRP44 PE=4 SV	OPHHA	2
2432	3.41	3.43	42.35	3.128	2.198	tr|V8NU99|V8NU99_OPHHA PDZ domain-containing protein 8 (Fragment) OS=Ophiophag	OPHHA	2
2433	3.41	3.41	35.96	5.512	4.462	tr|V8NBD3|V8NBD3_OPHHA Contactin-1 (Fragment) OS=Ophiophagus hannah GN=CNTN1 P	OPHHA	2
2434	3.4	3.4	16.76	4.655	4.655	tr|V8PC35|V8PC35_OPHHA Nucleoporin p58/p45 (Fragment) OS=Ophiophagus hannah GN	OPHHA	2
2435	3.39	3.39	25.81	16.77	10.32	tr|V8P3R6|V8P3R6_OPHHA Tumor protein p53-inducible protein 11 OS=Ophiophagus h	OPHHA	2
2436	3.37	8.73	32.62	13.98	11.26	tr|V8P5N0|V8P5N0_OPHHA Importin subunit alpha OS=Ophiophagus hannah GN=KPNA1 P	OPHHA	5
2437	3.36	3.49	41.69	14.42	10.66	tr|V8P2U9|V8P2U9_OPHHA Protein FAM84A (Fragment) OS=Ophiophagus hannah GN=FAM8	OPHHA	2
2438	3.36	3.37	37.91	2.662	2.662	tr|V8NNH2|V8NNH2_OPHHA Importin-8 (Fragment) OS=Ophiophagus hannah GN=IPO8 PE=	OPHHA	4
2439	3.36	3.36	53.87	11.45	9.091	tr|V8NE76|V8NE76_OPHHA Surfeit locus protein 1 (Fragment) OS=Ophiophagus hanna	OPHHA	2
2440	3.36	3.36	37.5	15.13	13.82	tr|V8NNS4|V8NNS4_OPHHA 1-acyl-sn-glycerol-3-phosphate acyltransferase beta OS=	OPHHA	2
2441	3.35	3.36	54.61	29.08	29.08	tr|V8NOK4|V8NOK4_OPHHA EH domain-containing protein 1 (Fragment) OS=Ophiophagu	OPHHA	2
2442	3.35	3.35	34.04	12.12	9.038	tr|V8PHA5|V8PHA5_OPHHA Spondin-1 (Fragment) OS=Ophiophagus hannah GN=SPON1 PE=	OPHHA	2
2443	3.35	3.35	31.36	6.684	6.684	tr|V8PH48|V8PH48_OPHHA RNA-binding motif, single-stranded-interacting protein	OPHHA	2
2444	3.34	3.34	55.91	8.861	8.861	tr|V8NJ65|V8NJ65_OPHHA Tetratricopeptide repeat protein 27 (Fragment) OS=Ophio	OPHHA	3
2445	3.34	3.34	43.36	20.31	4.297	tr|V8P5S4|V8P5S4_OPHHA Guanylate cyclase soluble subunit alpha-2 (Fragment) OS	OPHHA	1
2446	3.33	3.34	41.22	7.379	1.908	tr|V8NRN9|V8NRN9_OPHHA Cleavage and polyadenylation specificity factor subunit	OPHHA	1
2447	3.33	3.33	57.42	7.742	7.742	tr|V8PAN5|V8PAN5_OPHHA CDKN2A-interacting protein OS=Ophiophagus hannah GN=Cdk	OPHHA	2
2448	3.32	3.46	42.15	3.927	1.813	tr|V8P8K9|V8P8K9_OPHHA UDP-glucose:glycoprotein glucosyltransferase 1 (Fragmen	OPHHA	2
2449	3.32	3.33	64.41	7.415	7.415	tr|V8PAH2|V8PAH2_OPHHA DNA methyltransferase 1-associated protein 1 (Fragment)	OPHHA	2
2450	3.32	3.33	68.66	20.15	20.15	tr|V8N8C4|V8N8C4_OPHHA Phosphatidylinositol 3-kinase catalytic subunit type 3	OPHHA	2
2451	3.32	3.32	31.15	5.533	5.533	tr|V8NR93|V8NR93_OPHHA Clusterin (Fragment) OS=Ophiophagus hannah GN=CLU PE=3	OPHHA	3
2452	3.31	3.31	41.21	2.633	1.531	tr|V8NZR7|V8NZR7_OPHHA Serine/threonine-protein kinase TAO2 (Fragment) OS=Ophi	OPHHA	2
2453	3.31	3.31	53.44	3.077	2.429	tr|V8NU12|V8NU12_OPHHA Protein Shroom1 (Fragment) OS=Ophiophagus hannah GN=shr	OPHHA	2
2454	3.31	3.31	35.67	1.619	1.619	tr|V8NQA0|V8NQA0_OPHHA Rho GTPase-activating protein 32 (Fragment) OS=Ophiopha	OPHHA	2
2455	3.31	3.31	28.39	4.816	2.251	tr|V8P468|V8P468_OPHHA Zinc finger SWIM domain-containing protein (Fragment) O	OPHHA	2
2456	3.31	3.31	37.9	4.338	4.338	tr|V8N8V2|V8N8V2_OPHHA Rhomboid-like protein (Fragment) OS=Ophiophagus hannah	OPHHA	3
2457	3.31	3.31	41.81	7.345	7.345	tr|V8NA44|V8NA44_OPHHA Protein jagunal-like 1 (Fragment) OS=Ophiophagus hannah	OPHHA	2
2458	3.3	4.32	52.17	2.879	1.952	tr|V8PIZ1|V8PIZ1_OPHHA Myosin-3 (Fragment) OS=Ophiophagus hannah GN=Myh3 PE=4	OPHHA	4
2459	3.3	3.31	49.59	10.33	10.33	tr|V8PIY6|V8PIY6_OPHHA Uncharacterized protein OS=Ophiophagus hannah GN=L345_0	OPHHA	2
2460	3.3	3.3	59.18	7.823	3.912	tr|V8P9Q8|V8P9Q8_OPHHA La-related protein 7 OS=Ophiophagus hannah GN=LARP7 PE=	OPHHA	2
2461	3.3	3.3	33.68	11.34	11.34	tr|V8PF00|V8PF00_OPHHA RNA binding protein fox-1-like 2 OS=Ophiophagus hannah	OPHHA	2
2462	3.3	3.3	32.35	10.13	10.13	tr|V8NBQ5|V8NBQ5_OPHHA 5-oxoprolinase (Fragment) OS=Ophiophagus hannah GN=Opla	OPHHA	2
2463	3.29	6.18	40.95	9.524	8.19	tr|V8NW43|V8NW43_OPHHA STE20/SPS1-related proline-alanine-rich protein kinase	OPHHA	4
2464	3.29	3.31	28.77	3.774	3.774	tr|V8P1M3|V8P1M3_OPHHA RNA-binding protein 26 OS=Ophiophagus hannah GN=RBM26 P	OPHHA	2
2465	3.29	3.3	61.27	15.85	15.85	tr|V8NIZ8|V8NIZ8_OPHHA RING finger protein OS=Ophiophagus hannah GN=RNF141 PE=	OPHHA	3
2466	3.29	3.29	51.9	1.62	1.62	tr|V8N7J9|V8N7J9_OPHHA Rho GTPase-activating protein 21 OS=Ophiophagus hannah	OPHHA	2
2467	3.29	3.29	44.36	2.159	1.542	tr|V8PCE0|V8PCE0_OPHHA Ankyrin repeat and SAM domain-containing protein 6 (Fra	OPHHA	2
2468	3.29	3.29	50.72	4.337	3.614	tr|V8NE10|V8NE10_OPHHA Protein CIP2A-like protein (Fragment) OS=Ophiophagus ha	OPHHA	2
2469	3.29	3.29	40.14	4.577	4.577	tr|V8NKB1|V8NKB1_OPHHA Oxidoreductase NAD-binding domain-containing protein 1	OPHHA	2
2470	3.28	3.32	33.67	12.24	7.823	tr|V8NYL5|V8NYL5_OPHHA Golgi pH regulator OS=Ophiophagus hannah GN=GPR89 PE=4	OPHHA	2
2471	3.28	3.28	32.47	19.48	14.94	tr|V8N771|V8N771_OPHHA Galectin (Fragment) OS=Ophiophagus hannah GN=LGALS9C PE	OPHHA	4
2472	3.27	3.27	54.35	10.25	5.609	tr|V8NS41|V8NS41_OPHHA Cell cycle regulator Mat89Bb-like protein (Fragment) OS	OPHHA	2
2473	3.27	3.27	46.13	9.369	5.766	tr|V8NIW5|V8NIW5_OPHHA Cytochrome protein (Fragment) OS=Ophiophagus hannah GN=	OPHHA	3
2474	3.27	3.27	79.49	20	12.31	tr|V8P295|V8P295_OPHHA Prothrombin (Fragment) OS=Ophiophagus hannah GN=F2 PE=3	OPHHA	2
2475	3.27	3.27	49.07	8.696	8.696	tr|V8N2L3|V8N2L3_OPHHA Uncharacterized protein (Fragment) OS=Ophiophagus hanna	OPHHA	2
2476	3.25	3.25	40.16	2.561	2.561	tr|V8NFQ4|V8NFQ4_OPHHA Phospholipid-transporting ATPase (Fragment) OS=Ophiopha	OPHHA	2
2477	3.25	3.25	44.36	4.246	4.246	tr|V8NKV9|V8NKV9_OPHHA DEP domain-containing protein 5 (Fragment) OS=Ophiophag	OPHHA	2
2478	3.25	3.25	41.48	7.615	5.411	tr|V8NEL7|V8NEL7_OPHHA Disks large-associated protein 4 (Fragment) OS=Ophiopha	OPHHA	2

2479	3.25	3.25	25.42	4.583	2.396	tr|V8P2I7|V8P2I7_OPHHA	Importin-13 (Fragment) OS=Ophiophagus hannah GN=IPO13 P	OPHHA	2
2480	3.25	3.25	45.11	7.16	7.16	tr|V8NPP8|V8NPP8_OPHHA	Uncharacterized protein OS=Ophiophagus hannah GN=L345_1	OPHHA	2
2481	3.25	3.25	63.95	13.73	8.155	tr|V8PCQ3|V8PCQ3_OPHHA	Uncharacterized protein (Fragment) OS=Ophiophagus hanna	OPHHA	2
2482	3.25	3.25	23.77	5.85	4.753	tr|V8NA18|V8NA18_OPHHA	TBC domain-containing protein kinase-like protein (Frag	OPHHA	2
2483	3.24	3.28	15.64	5.545	5.545	tr|V8NKR5|V8NKR5_OPHHA	Sodium-coupled neutral amino acid transporter 3 OS=Ophi	OPHHA	2
2484	3.24	3.25	39	2.259	1.784	tr|V8P9P2|V8P9P2_OPHHA	Rapamycin-insensitive companion of mTOR (Fragment) OS=O	OPHHA	2
2485	3.24	3.24	49.18	8.197	8.197	tr|V8PO22|V8PO22_OPHHA	Rho-related GTP-binding protein RhoE OS=Ophiophagus han	OPHHA	2
2486	3.24	3.24	45.08	10.25	10.25	tr|V8NR32|V8NR32_OPHHA	Gem-associated protein 2 OS=Ophiophagus hannah GN=GEMIN	OPHHA	2
2487	3.23	3.23	33	2.242	2.242	tr|V8NRY6|V8NRY6_OPHHA	WD repeat-containing protein 24 (Fragment) OS=Ophiophag	OPHHA	2
2488	3.23	3.23	43.21	9.239	6.522	tr|V8N9F8|V8N9F8_OPHHA	Ribosomal L1 domain-containing protein 1 OS=Ophiophagus	OPHHA	2
2489	3.22	3.53	52.25	10.11	6.742	tr|V8P7Y7|V8P7Y7_OPHHA	Replication factor C subunit 3 OS=Ophiophagus hannah GN	OPHHA	2
2490	3.22	3.26	47.94	3.196	3.196	tr|V8NVW3|V8NVW3_OPHHA	Myosin-IA OS=Ophiophagus hannah GN=Myo31DF PE=4 SV=1	OPHHA	2
2491	3.22	3.22	58.04	6.309	6.309	tr|V8NRE8|V8NRE8_OPHHA	Ankyrin repeat and MYND domain-containing protein 2 OS=	OPHHA	2
2492	3.22	3.22	54.42	10.7	10.7	tr|V8P111|V8P111_OPHHA	DDB1-and CUL4-associated factor 11 (Fragment) OS=Ophiop	OPHHA	2
2493	3.21	3.21	41.77	5.17	3.946	tr|V8P621|V8P621_OPHHA	Uncharacterized protein (Fragment) OS=Ophiophagus hanna	OPHHA	2
2494	3.21	3.21	55.17	12.93	12.93	tr|V8N5D6|V8N5D6_OPHHA	Uncharacterized protein (Fragment) OS=Ophiophagus hanna	OPHHA	2
2495	3.2	3.2	43.72	1.826	1.826	tr|V8NAM7|V8NAM7_OPHHA	Uncharacterized protein (Fragment) OS=Ophiophagus hanna	OPHHA	2
2496	3.2	3.2	47.5	14.5	9.25	tr|V8NPH7|V8NPH7_OPHHA	Zinc finger FYVE domain-containing protein 19 (Fragment	OPHHA	2
2497	3.2	3.2	13.21	9.286	8.929	tr|V8NRF5|V8NRF5_OPHHA	Transmembrane protein (Fragment) OS=Ophiophagus hannah	OPHHA	2
2498	3.19	3.22	47.15	4.863	4.863	tr|V8NQA3|V8NQA3_OPHHA	Chitotriosidase-1 (Fragment) OS=Ophiophagus hannah GN=C	OPHHA	2
2499	3.19	3.19	57.39	7.536	3.333	tr|V8P4V0|V8P4V0_OPHHA	Uncharacterized protein (Fragment) OS=Ophiophagus hanna	OPHHA	2
2500	3.19	3.19	30.01	3.547	3.547	tr|V8PB58|V8PB58_OPHHA	NEDD4-binding protein 1 (Fragment) OS=Ophiophagus hanna	OPHHA	2
2501	3.18	3.2	25.25	1.69	1.69	tr|V8PG89|V8PG89_OPHHA	Ral GTPase-activating protein subunit alpha-1 (Fragment	OPHHA	3
2502	3.18	3.2	48.15	33.33	21.3	tr|V8NER7|V8NER7_OPHHA	Leucine-rich repeat-containing protein 16A (Fragment) O	OPHHA	2
2503	3.18	3.18	44.6	5.755	5.755	tr|V8NEN8|V8NEN8_OPHHA	Tripartite motif-containing protein 39 (Fragment) OS=Op	OPHHA	2
2504	3.18	3.18	49.14	18.97	18.97	tr|V8NHA7|V8NHA7_OPHHA	Uncharacterized protein OS=Ophiophagus hannah GN=L345_1	OPHHA	2
2505	3.16	3.16	42.82	3.457	3.457	tr|V8P4A1|V8P4A1_OPHHA	Dedicator of cytokinesis protein 1 OS=Ophiophagus hanna	OPHHA	2
2506	3.15	3.15	56.43	8.233	6.024	tr|V8NG59|V8NG59_OPHHA	Pre-B-cell leukemia transcription factor-interacting pr	OPHHA	2
2507	3.15	3.15	64.4	15.6	9.6	tr|V8PF23|V8PF23_OPHHA	Vesicle transport protein USE1 (Fragment) OS=Ophiophagu	OPHHA	2
2508	3.15	3.15	54.58	8.765	8.765	tr|V8P3Q6|V8P3Q6_OPHHA	ELMO domain-containing protein 2 OS=Ophiophagus hannah	OPHHA	2
2509	3.15	3.15	36.49	8.635	8.635	tr|V8PAB2|V8PAB2_OPHHA	Ubiquitin carboxyl-terminal hydrolase 12 (Fragment) OS=	OPHHA	2
2509	0	2	28.99	4.423	4.423	tr|V8N5M7|V8N5M7_OPHHA	Ubiquitin carboxyl-terminal hydrolase 46 (Fragment) OS=	OPHHA	1
2510	3.14	3.22	54.81	7.115	5	tr|V8NHL1|V8NHL1_OPHHA	Pyruvate kinase muscle isozyme (Fragment) OS=Ophiophagu	OPHHA	2
2511	3.14	3.14	66.23	7.456	5.044	tr|V8NK82|V8NK82_OPHHA	Golgin subfamily A member 2 OS=Ophiophagus hannah GN=GO	OPHHA	2
2512	3.14	3.14	27.45	9.586	7.843	tr|V8PFY5|V8PFY5_OPHHA	Sorting nexin-18 (Fragment) OS=Ophiophagus hannah GN=Sn	OPHHA	3
2513	3.14	3.14	69.35	16.94	16.94	tr|V8P3Z8|V8P3Z8_OPHHA	Uncharacterized protein (Fragment) OS=Ophiophagus hanna	OPHHA	2
2514	3.12	3.17	32.16	14.04	14.04	tr|V8N8Z4|V8N8Z4_OPHHA	Cytochrome protein (Fragment) OS=Ophiophagus hannah GN=	OPHHA	3
2515	3.12	3.12	50.7	16.2	4.93	tr|V8NUC4|V8NUC4_OPHHA	GDP-fucose protein O-fucosyltransferase 2 (Fragment) OS	OPHHA	2
2516	3.12	3.12	45.15	12.42	9.697	tr|V8NH44|V8NH44_OPHHA	55 kDa erythrocyte membrane protein (Fragment) OS=Ophio	OPHHA	2
2517	3.11	3.11	45.41	6.118	6.118	tr|V8NPS2|V8NPS2_OPHHA	Protein AATF (Fragment) OS=Ophiophagus hannah GN=AATF P	OPHHA	2
2518	3.11	3.11	93.75	37.5	37.5	tr|V8N6J0|V8N6J0_OPHHA	Fragile X mental retardation syndrome-related protein 2	OPHHA	2
2519	3.1	3.1	48.18	11.94	4.656	tr|V8PFT7|V8PFT7_OPHHA	Ganglioside-induced differentiation-associated protein	OPHHA	2
2520	3.1	3.1	48.45	8.031	5.699	tr|V8PHA3|V8PHA3_OPHHA	PCI domain-containing protein 2 (Fragment) OS=Ophiophag	OPHHA	2
2521	3.1	3.1	33.85	21.54	12.31	tr|V8PEM2|V8PEM2_OPHHA	Dynactin subunit 6 (Fragment) OS=Ophiophagus hannah GN=	OPHHA	2
2522	3.1	3.1	63.11	23.77	17.21	tr|V8N3W1|V8N3W1_OPHHA	Uncharacterized protein (Fragment) OS=Ophiophagus hanna	OPHHA	2
2523	3.1	3.1	83.33	45	45	tr|V8N1M8|V8N1M8_OPHHA	Uncharacterized protein (Fragment) OS=Ophiophagus hanna	OPHHA	2
2524	3.09	3.11	22.59	9.917	5.51	tr|V8NY90|V8NY90_OPHHA	Aminoglycoside phosphotransferase domain-containing pro	OPHHA	2
2525	3.09	3.09	40.73	2.909	2.909	tr|V8PBP8|V8PBP8_OPHHA	Transmembrane protein 68 (Fragment) OS=Ophiophagus hann	OPHHA	2
2526	3.08	3.09	44.3	8.725	6.711	tr|V8P655|V8P655_OPHHA	Calcium signal-modulating cyclophilin ligand (Fragment)	OPHHA	2
2527	3.08	3.08	72.41	13.79	13.79	tr|V8N8X9|V8N8X9_OPHHA	Allograft inflammatory factor 1-like protein (Fragment)	OPHHA	2
2528	3.08	3.08	21.4	8.696	6.355	tr|V8NTK6|V8NTK6_OPHHA	Derlin-1 (Fragment) OS=Ophiophagus hannah GN=DERL1 PE=4	OPHHA	2
2529	3.07	3.07	29.96	1.673	1.673	tr|V8P311|V8P311_OPHHA	Protein-associating with the carboxyl-terminal domain o	OPHHA	2
2530	3.07	3.07	31.96	6.545	3.957	tr|V8NVB0|V8NVB0_OPHHA	GTP-binding protein 1 OS=Ophiophagus hannah GN=GTPBP1 P	OPHHA	2
2531	3.07	3.07	44.98	3.903	3.903	tr|V8P8R1|V8P8R1_OPHHA	Tether containing UBX domain for GLUT4 (Fragment) OS=Op	OPHHA	2
2532	3.07	3.07	35.2	12.01	4.555	tr|V8NNC0|V8NNC0_OPHHA	Regulator of microtubule dynamics protein 3 (Fragment)	OPHHA	2
2533	3.06	5.15	58.28	8.284	8.284	tr|V8NI55|V8NI55_OPHHA	Fibroblast growth factor receptor 2 (Fragment) OS=Ophio	OPHHA	3
2533	0.01	4.02	30.12	2.143	2.143	tr|V8PHB3|V8PHB3_OPHHA	Fibroblast growth factor receptor 4 (Fragment) OS=Ophio	OPHHA	2
2533	0	2.01	20.09	1.129	1.129	tr|V8NKM6|V8NKM6_OPHHA	Receptor protein-tyrosine kinase (Fragment) OS=Ophiopha	OPHHA	1
2534	3.06	3.06	55.31	13.08	10.08	tr|V8NLS4|V8NLS4_OPHHA	Cytohesin-2 OS=Ophiophagus hannah GN=CYTH2 PE=4 SV=1	OPHHA	2
2535	3.06	3.06	45.81	10.97	10.97	tr|V8P8C0|V8P8C0_OPHHA	28S ribosomal protein S31, mitochondrial OS=Ophiophagus	OPHHA	2
2536	3.06	3.06	75.76	21.21	21.21	tr|V8NU98|V8NU98_OPHHA	Uncharacterized protein (Fragment) OS=Ophiophagus hanna	OPHHA	2
2537	3.05	5.19	38.76	30.23	30.23	tr|V8NSM1|V8NSM1_OPHHA	Histone H2A (Fragment) OS=Ophiophagus hannah GN=L345_09	OPHHA	3
2537	0	6	33.33	30.95	30.95	tr|V8NTL3|V8NTL3_OPHHA	Histone H2A (Fragment) OS=Ophiophagus hannah GN=HIST2H2	OPHHA	3
2538	3.05	3.05	42.25	2.89	1.147	tr|V8NQ19|V8NQ19_OPHHA	Cation-independent mannose-6-phosphate receptor OS=Ophi	OPHHA	2
2539	3.05	3.05	36.39	2.128	2.128	tr|V8NX67|V8NX67_OPHHA	C-type mannose receptor 2 (Fragment) OS=Ophiophagus han	OPHHA	2
2540	3.05	3.05	30.26	8.216	2.605	tr|V8NGF6|V8NGF6_OPHHA	Aminopeptidase N (Fragment) OS=Ophiophagus hannah GN=AN	OPHHA	2
2541	3.05	3.05	13.58	3.88	3.88	tr|V8PJH3|V8PJH3_OPHHA	Protein arginine N-methyltransferase 3 (Fragment) OS=Op	OPHHA	2
2542	3.05	3.05	24.43	15.27	10.69	tr|V8NFJ8|V8NFJ8_OPHHA	Maspardin (Fragment) OS=Ophiophagus hannah GN=Spg21 PE=	OPHHA	2
2543	3.05	3.05	24.32	10.81	10.81	tr|V8NUJ5|V8NUJ5_OPHHA	Alkylglycerol monooxygenase OS=Ophiophagus hannah GN=ag	OPHHA	2
2544	3.04	3.24	54.18	8.696	3.846	tr|V8NDU6|V8NDU6_OPHHA	TBC1 domain family member 4 (Fragment) OS=Ophiophagus h	OPHHA	2
2545	3.04	3.04	34.95	8.981	4.854	tr|V8P5I9|V8P5I9_OPHHA	Muscleblind-like protein 1 (Fragment) OS=Ophiophagus ha	OPHHA	2
2545	0	3.04	33.33	13.86	7.491	tr|V8PD64|V8PD64_OPHHA	Muscleblind-like protein 3 OS=Ophiophagus hannah GN=mbn	OPHHA	2
2545	0	1.68	21.6	7.467	2.933	tr|V8P8K4|V8P8K4_OPHHA	Muscleblind-like protein 2 (Fragment) OS=Ophiophagus ha	OPHHA	1
2546	3.04	3.04	40.37	19.72	8.257	tr|V8NVZ4|V8NVZ4_OPHHA	Uncharacterized protein (Fragment) OS=Ophiophagus hanna	OPHHA	2
2547	3.04	3.04	24.21	9.474	6.842	tr|V8NUC1|V8NUC1_OPHHA	Endonuclease domain-containing 1 protein (Fragment) OS=	OPHHA	2
2548	3.03	3.05	47.07	8.511	8.511	tr|V8P7W8|V8P7W8_OPHHA	Guanine nucleotide-binding protein G(Z) subunit alpha (	OPHHA	2
2549	3.03	3.03	61.85	5.749	4.181	tr|V8PB37|V8PB37_OPHHA	Myotubularin-related protein 6 (Fragment) OS=Ophiophagu	OPHHA	2
2550	3.03	3.03	58.17	6.422	3.67	tr|V8NNY7|V8NNY7_OPHHA	Lamin-B receptor (Fragment) OS=Ophiophagus hannah GN=LB	OPHHA	2
2551	3.02	3.02	32.17	4.755	4.755	tr|V8P4R8|V8P4R8_OPHHA	Mannan-binding lectin serine protease 2 OS=Ophiophagus	OPHHA	2
2552	3.02	3.02	49.15	7.458	7.458	tr|V8NRP3|V8NRP3_OPHHA	Mitochondrial carnitine/acylcarnitine carrier protein C	OPHHA	2
2553	3.01	3.01	62.79	6.977	4.651	tr|V8NW42|V8NW42_OPHHA	Phosphorylase b kinase gamma catalytic chain, testis/li	OPHHA	3
2554	3.01	3.01	29.06	3.973	2.838	tr|V8P682|V8P682_OPHHA	Ectonucleotide pyrophosphatase/phosphodiesterase family	OPHHA	2
2555	3	8.51	51.03	19.01	11.36	tr|V8NRY8|V8NRY8_OPHHA	Vacuolar protein sorting-associated protein 4B (Fragmen	OPHHA	4
2556	3	3	32.71	4.122	2.793	tr|V8PD03|V8PD03_OPHHA	Aftiphilin (Fragment) OS=Ophiophagus hannah GN=AFTPH PE	OPHHA	2
2557	3	3	45.07	17.25	13.73	tr|V8P5R4|V8P5R4_OPHHA	Phosphatase and actin regulator OS=Ophiophagus hannah G	OPHHA	2
2558	3	3	38.42	11.82	11.82	tr|V8N7B6|V8N7B6_OPHHA	Uncharacterized protein (Fragment) OS=Ophiophagus hanna	OPHHA	2
2559	2.99	2.99	50	5.263	5.263	tr|V8N799|V8N799_OPHHA	Sterile alpha and TIR motif-containing protein 1 (Fragm	OPHHA	2
2560	2.99	2.99	43.8	12.79	8.915	tr|V8PFQ7|V8PFQ7_OPHHA	Carbonic anhydrase 13 OS=Ophiophagus hannah GN=Ca13 PE=	OPHHA	2
2561	2.99	2.99	49.68	20	20	tr|V8NEW7|V8NEW7_OPHHA	Endothelial differentiation-related factor 1-like prote	OPHHA	2
2562	2.99	2.99	15.82	6.646	6.646	tr|V8PAR7|V8PAR7_OPHHA	Cyclic AMP-responsive element-binding protein 1 (Fragme	OPHHA	2
2563	2.98	10.28	67.57	21.13	15.72	tr|V8N7F6|V8N7F6_OPHHA	Polyadenylate-binding protein 4 (Fragment) OS=Ophiophag	OPHHA	6
2564	2.98	3.01	51.08	4.574	3.685	tr|V8NJ85|V8NJ85_OPHHA	Semaphorin-4C OS=Ophiophagus hannah GN=Sema4c PE=4 SV=1	OPHHA	2
2565	2.98	2.98	49.71	22.54	17.92	tr|V8NX15|V8NX15_OPHHA	Vesicle-associated membrane protein 7 (Fragment) OS=Oph	OPHHA	2
2566	2.98	2.98	53.65	10.73	10.73	tr|V8P9E3|V8P9E3_OPHHA	Acyl-coenzyme A thioesterase 10, mitochondrial (Fragmen	OPHHA	2
2567	2.97	2.97	63.47	3.276	1.556	tr|V8N9V0|V8N9V0_OPHHA	Synemin (Fragment) OS=Ophiophagus hannah GN=Synm PE=4 S	OPHHA	3
2568	2.97	2.97	30.54	3.829	2.917	tr|V8P0Z7|V8P0Z7_OPHHA	Phospholipid-transporting ATPase (Fragment) OS=Ophiopha	OPHHA	3

2569	2.97	2.97	79.62	15.29	15.29 tr|V8NY99|V8NY99_OPHHA Endonuclease 8-like 1 (Fragment) OS=Ophiophagus hannah	OPHHA	2
2570	2.97	2.97	49.59	9.016	9.016 tr|V8P2Z4|V8P2Z4_OPHHA Ubiquitin domain-containing protein UBFD1 (Fragment) OS	OPHHA	2
2571	2.97	2.97	74.42	19.53	11.63 tr|V8NCF9|V8NCF9_OPHHA Caspase-3 (Fragment) OS=Ophiophagus hannah GN=CASP3 PE=	OPHHA	2
2572	2.97	2.97	35.41	14.79	8.171 tr|V8P9G8|V8P9G8_OPHHA KH domain-containing, RNA-binding, signal transduction-	OPHHA	2
2573	2.97	2.97	47.42	13.92	13.92 tr|V8NBE2|V8NBE2_OPHHA MAGUK p55 subfamily member 7 (Fragment) OS=Ophiophagus	OPHHA	2
2574	2.96	3.87	35.73	7.625	5.447 tr|V8NU81|V8NU81_OPHHA Tumor suppressor candidate 3 OS=Ophiophagus hannah GN=T	OPHHA	3
2575	2.96	2.96	39.24	1.549	1.549 tr|V8PHV3|V8PHV3_OPHHA Putative helicase with zinc finger domain protein (Frag	OPHHA	2
2576	2.96	2.96	49.14	2.058	2.058 tr|V8N8A7|V8N8A7_OPHHA NACHT, LRR and PYD domains-containing protein 12 (Fragm	OPHHA	2
2577	2.96	2.96	50.97	7.799	3.621 tr|V8PDQ5|V8PDQ5_OPHHA Adseverin OS=Ophiophagus hannah GN=SCIN PE=4 SV=1	OPHHA	2
2578	2.96	2.96	50	7.831	7.831 tr|V8NH53|V8NH53_OPHHA Histone acetyltransferase (Fragment) OS=Ophiophagus han	OPHHA	2
2579	2.96	2.96	50.3	7.298	4.931 tr|V8NK97|V8NK97_OPHHA Rab-3A-interacting protein (Fragment) OS=Ophiophagus ha	OPHHA	2
2580	2.96	2.96	43.48	13.66	9.317 tr|V8P4Y7|V8P4Y7_OPHHA Uncharacterized protein (Fragment) OS=Ophiophagus hanna	OPHHA	2
2581	2.95	2.95	73.78	19.85	13.86 tr|V8NDB9|V8NDB9_OPHHA Syntaxin-18 OS=Ophiophagus hannah GN=STX18 PE=4 SV=1	OPHHA	2
2582	2.95	2.95	59.46	34.23	19.82 tr|V8NE38|V8NE38_OPHHA Agrin (Fragment) OS=Ophiophagus hannah GN=AGRN PE=4 SV=	OPHHA	2
2583	2.94	3.19	52.98	5.614	3.392 tr|V8NP87|V8NP87_OPHHA Pre-mRNA-processing factor 40-like B (Fragment) OS=Ophi	OPHHA	2
2584	2.94	2.94	39.55	4.264	2.665 tr|V8NJL5|V8NJL5_OPHHA Cyclin-T2 (Fragment) OS=Ophiophagus hannah GN=CCNT2 PE=	OPHHA	2
2585	2.94	2.94	51.02	25.17	25.17 tr|V8P306|V8P306_OPHHA cAMP-regulated phosphoprotein 19 (Fragment) OS=Ophiopha	OPHHA	2
2586	2.94	2.94	46.03	9.205	9.205 tr|V8PII2|V8PII2_OPHHA 39S ribosomal protein L45, mitochondrial OS=Ophiophagus	OPHHA	2
2587	2.94	2.94	26.19	8.036	8.036 tr|V8NDT9|V8NDT9_OPHHA Steryl-sulfatase (Fragment) OS=Ophiophagus hannah GN=ST	OPHHA	2
2588	2.93	9.04	29.81	12.78	11.67 tr|V8NNB7|V8NNB7_OPHHA Importin subunit alpha-6 (Fragment) OS=Ophiophagus hann	OPHHA	6
2589	2.93	4.3	71.24	23.45	16.37 tr|V8NKW6|V8NKW6_OPHHA Chloride intracellular channel protein 2 OS=Ophiophagus	OPHHA	3
2590	2.92	2.97	59.69	5.581	1.473 tr|V8NRE0|V8NRE0_OPHHA A-kinase anchor protein 9 (Fragment) OS=Ophiophagus han	OPHHA	1
2591	2.92	2.92	54.16	4.836	4.836 tr|V8PGK5|V8PGK5_OPHHA Poly [ADP-ribose] polymerase 12 (Fragment) OS=Ophiophag	ADP-ribos	2
2592	2.92	2.92	49.24	9.15	9.15 tr|V8NKW2|V8NKW2_OPHHA 3-oxoacyl-[acyl-carrier-protein] synthase, mitochondria	OPHHA	3
2593	2.92	2.92	40.07	17.73	4.255 tr|V8NXW0|V8NXW0_OPHHA Cathepsin D (Fragment) OS=Ophiophagus hannah GN=CTSD PE	OPHHA	1
2594	2.92	2.92	39.38	13.72	13.72 tr|V8PGY5|V8PGY5_OPHHA U2 small nuclear ribonucleoprotein B OS=Ophiophagus han	OPHHA	2
2595	2.92	2.92	42.04	16.56	16.56 tr|V8NVF8|V8NVF8_OPHHA 39S ribosomal protein L30, mitochondrial OS=Ophiophagus	OPHHA	2
2596	2.92	2.92	46.91	27.16	27.16 tr|V8PB96|V8PB96_OPHHA Uncharacterized protein OS=Ophiophagus hannah GN=L345_0	OPHHA	2
2597	2.91	5.18	52.31	20.51	15.9 tr|V8NYM4|V8NYM4_OPHHA Serine/arginine-rich splicing factor 7 OS=Ophiophagus h	OPHHA	3
2598	2.91	2.91	89.25	17.76	5.14 tr|V8NFT7|V8NFT7_OPHHA 39S ribosomal protein L47, mitochondrial (Fragment) OS=	OPHHA	1
2599	2.91	2.91	67.98	10.34	10.34 tr|V8NYI9|V8NYI9_OPHHA Cholesterol 24-hydroxylase OS=Ophiophagus hannah GN=Cyp	OPHHA	2
2600	2.91	2.91	71.33	22	16.67 tr|V8NOW2|V8NOW2_OPHHA Uncharacterized protein (Fragment) OS=Ophiophagus hanna	OPHHA	2
2601	2.9	2.9	22.97	3.596	3.596 tr|V8NGE6|V8NGE6_OPHHA Integrator complex subunit 2 (Fragment) OS=Ophiophagus	OPHHA	2
2602	2.9	2.9	57.48	10.28	10.28 tr|V8NUT4|V8NUT4_OPHHA Charged multivesicular body protein 2b OS=Ophiophagus h	OPHHA	2
2603	2.9	2.9	52.82	11.79	11.79 tr|V8P7G3|V8P7G3_OPHHA NF-kappa-B inhibitor-interacting Ras-like protein 2 OS=	OPHHA	2
2604	2.9	2.9	51.64	20.49	20.49 tr|V8N5W3|V8N5W3_OPHHA Phosphomannomutase (Fragment) OS=Ophiophagus hannah GN=	OPHHA	3
2605	2.89	2.94	36.92	8.462	6.923 tr|V8P8E2|V8P8E2_OPHHA Secernin-3 (Fragment) OS=Ophiophagus hannah GN=SCRN3 PE	OPHHA	2
2606	2.89	2.89	55.14	3.618	3.618 tr|V8NQH9|V8NQH9_OPHHA 2-5A-dependent ribonuclease (Fragment) OS=Ophiophagus h	OPHHA	2
2607	2.89	2.89	39.07	9.091	6.143 tr|V8PG65|V8PG65_OPHHA Neutral cholesterol ester hydrolase 1 OS=Ophiophagus ha	OPHHA	2
2608	2.89	2.89	28.82	5.222	5.222 tr|V8PHN9|V8PHN9_OPHHA Apolipoprotein F (Fragment) OS=Ophiophagus hannah GN=AP	OPHHA	2
2609	2.89	2.89	63.71	9.677	9.677 tr|V8P543|V8P543_OPHHA 60S ribosomal protein L24 (Fragment) OS=Ophiophagus han	OPHHA	2
2610	2.88	9.19	53.05	14.93	14.73 tr|V8NMQ1|V8NMQ1_OPHHA Serine/threonine-protein phosphatase 2A 55 kDa regulato	OPHHA	5
2611	2.88	7.43	50	29.17	21.76 tr|V8PG32|V8PG32_OPHHA ADP-ribosylation factor-like protein 8B (Fragment) OS=O	OPHHA	5
2612	2.88	2.88	37.95	7.78	3.795 tr|V8P8E8|V8P8E8_OPHHA Tubby-like protein OS=Ophiophagus hannah GN=Tub PE=3 SV	OPHHA	2
2613	2.88	2.88	33.5	19.9	11.17 tr|V8N5Y5|V8N5Y5_OPHHA 26S proteasome non-ATPase regulatory subunit 4 (Fragmen	OPHHA	2
2614	2.87	2.88	34.93	3.425	3.425 tr|V8P061|V8P061_OPHHA Anoctamin (Fragment) OS=Ophiophagus hannah GN=ANO1 PE=3	OPHHA	2
2615	2.87	2.87	43.8	3.17	3.17 tr|V8NPZ6|V8NPZ6_OPHHA Leucine-rich repeat-containing protein 8A (Fragment) OS	OPHHA	2
2616	2.87	2.87	33.89	7.778	5.278 tr|V8P9X0|V8P9X0_OPHHA Ketosamine-3-kinase (Fragment) OS=Ophiophagus hannah GN	OPHHA	2
2617	2.87	2.87	16.1	3.121	3.121 tr|V8NKU7|V8NKU7_OPHHA TSC22 domain family protein 2 (Fragment) OS=Ophiophagus	OPHHA	2
2618	2.87	2.87	58.51	39.36	25.53 tr|V8PGX1|V8PGX1_OPHHA Bridging integrator 3 (Fragment) OS=Ophiophagus hannah	OPHHA	2
2619	2.86	2.86	47.35	5.291	3.571 tr|V8PFF3|V8PFF3_OPHHA Myotubularin-related protein 10 (Fragment) OS=Ophiophag	OPHHA	2
2620	2.86	2.86	49.56	8.211	8.211 tr|V8P382|V8P382_OPHHA Cdc42 effector protein 4 (Fragment) OS=Ophiophagus hann	OPHHA	2
2621	2.86	2.86	57.55	18.87	5.66 tr|V8P2H9|V8P2H9_OPHHA Programmed cell death protein 10 OS=Ophiophagus hannah	OPHHA	1
2622	2.86	2.86	35.32	5.319	3.83 tr|V8NP75|V8NP75_OPHHA Uncharacterized protein (Fragment) OS=Ophiophagus hanna	OPHHA	2
2623	2.85	2.85	40.21	15.38	12.94 tr|V8NWF2|V8NWF2_OPHHA Putative hydroxypyruvate isomerase (Fragment) OS=Ophiop	OPHHA	2
2624	2.85	2.85	48.24	14.12	14.12 tr|V8NVK1|V8NVK1_OPHHA Polyadenylate-binding protein 2 OS=Ophiophagus hannah G	OPHHA	2
2625	2.84	2.84	37.73	3.83	2.837 tr|V8P9K1|V8P9K1_OPHHA CCR4-NOT transcription complex subunit 3 (Fragment) OS=	OPHHA	2
2626	2.84	2.84	51.72	10.34	7.958 tr|V8PH26|V8PH26_OPHHA Endoplasmic reticulum-Golgi intermediate compartment pr	OPHHA	3
2627	2.84	2.84	39.1	10.53	10.53 tr|V8P499|V8P499_OPHHA Sulfotransferase (Fragment) OS=Ophiophagus hannah GN=SU	OPHHA	2
2628	2.84	2.84	29.19	11.8	7.143 tr|V8NZL8|V8NZL8_OPHHA Phenazine biosynthesis-like domain-containing protein (	OPHHA	2
2629	2.83	2.83	41.94	6.183	3.763 tr|V8PFH8|V8PFH8_OPHHA Protein farnesyltransferase subunit beta OS=Ophiophagus	OPHHA	1
2630	2.83	2.83	58.79	13.57	13.57 tr|V8NLV1|V8NLV1_OPHHA 39S ribosomal protein L40, mitochondrial (Fragment) OS=	OPHHA	2
2631	2.82	4	43.24	9.459	9.459 tr|V8NXH0|V8NXH0_OPHHA Glyceraldehyde 3-phosphate dehydrogenase, testis-specif	OPHHA	3
2632	2.82	2.83	39.43	2.71	2.71 tr|V8P0N9|V8P0N9_OPHHA Long-chain-fatty-acid--CoA ligase 6 (Fragment) OS=Ophio	OPHHA	2
2633	2.82	2.83	74.14	13.79	5.747 tr|V8NG17|V8NG17_OPHHA Calpain small subunit 1 (Fragment) OS=Ophiophagus hanna	OPHHA	1
2634	2.82	2.82	45.68	3.602	3.602 tr|V8NV87|V8NV87_OPHHA ATP-dependent RNA helicase DDX51 (Fragment) OS=Ophiopha	OPHHA	2
2635	2.82	2.82	68.92	25	15.54 tr|V8NQF6|V8NQF6_OPHHA Vacuolar protein sorting-associated protein 29 (Fragmen	OPHHA	2
2635	0	2	28.5	3.14	3.14 tr|V8NHQ7|V8NHQ7_OPHHA Vacuolar protein sorting-associated protein 29 (Fragmen	OPHHA	1
2636	2.82	2.82	49.04	19.11	19.11 tr|V8ND40|V8ND40_OPHHA Stromal cell-derived factor 2-like protein 1 (Fragment)	OPHHA	3
2637	2.82	2.82	36.55	18.07	7.229 tr|V8NXP5|V8NXP5_OPHHA Copper homeostasis protein cutC-like protein OS=Ophioph	OPHHA	1
2638	2.8	2.8	54.15	6.829	6.829 tr|V8NM91|V8NM91_OPHHA tRNA-dihydrouridine(47) synthase [NAD(P)(+)] (Fragment)	NAD(P)(+)	2
2639	2.8	2.8	23.77	4.277	4.277 tr|V8NX04|V8NX04_OPHHA cGMP-inhibited 3',5'-cyclic phosphodiesterase A (Fragme	OPHHA	2
2640	2.8	2.8	40.4	6.623	6.623 tr|V8P7I4|V8P7I4_OPHHA Protein DPCD (Fragment) OS=Ophiophagus hannah GN=dpcd P	OPHHA	2
2641	2.8	2.8	54.44	11.83	11.83 tr|V8P9P6|V8P9P6_OPHHA Heme-binding protein 2 (Fragment) OS=Ophiophagus hannah	OPHHA	2
2642	2.8	2.8	48.72	14.36	10.26 tr|V8NMF4|V8NMF4_OPHHA Methionine-R-sulfoxide reductase B1 (Fragment) OS=Ophio	OPHHA	2
2643	2.8	2.8	64.46	35.54	26.45 tr|V8N403|V8N403_OPHHA N-alpha-acetyltransferase 20 (Fragment) OS=Ophiophagus	OPHHA	2
2644	2.79	7.05	61.94	11.55	6.824 tr|V8PHA4|V8PHA4_OPHHA Reticulocalbin-1 (Fragment) OS=Ophiophagus hannah GN=RC	OPHHA	3
2645	2.79	2.79	37.99	4.951	4.464 tr|V8PHG1|V8PHG1_OPHHA Coagulation factor X isoform 1 (Fragment) OS=Ophiophagu	OPHHA	2
2646	2.79	2.79	35.52	7.825	3.912 tr|V8PAB6|V8PAB6_OPHHA Hermansky-Pudlak syndrome 1 protein-like protein OS=Oph	OPHHA	2
2647	2.79	2.79	24.35	6.087	6.087 tr|V8NKY3|V8NKY3_OPHHA Cation-dependent mannose-6-phosphate receptor OS=Ophiop	OPHHA	2
2648	2.78	4.88	37.7	18.32	18.32 tr|V8N7Z8|V8N7Z8_OPHHA Rho-related GTP-binding protein RhoG (Fragment) OS=Ophi	OPHHA	3
2649	2.78	2.79	68.09	27.66	27.66 tr|V8N4E4|V8N4E4_OPHHA Uncharacterized protein (Fragment) OS=Ophiophagus hanna	OPHHA	2
2650	2.77	2.77	32.91	5.087	3.339 tr|V8NT82|V8NT82_OPHHA Protein-glutamine gamma-glutamyltransferase K (Fragment	OPHHA	2
2651	2.76	2.76	29.22	4.174	1.336 tr|V8PI65|V8PI65_OPHHA Exportin-4 OS=Ophiophagus hannah GN=XPO4 PE=3 SV=1	OPHHA	1
2652	2.76	2.76	62.64	6.32	2.416 tr|V8NRF6|V8NRF6_OPHHA Rap1 GTPase-GDP dissociation stimulator 1 (Fragment) OS	OPHHA	1
2653	2.76	2.76	49.03	9.749	3.064 tr|V8P8P7|V8P8P7_OPHHA Nuclear pore complex protein Nup50 (Fragment) OS=Ophiop	OPHHA	1
2654	2.76	2.76	33.33	11.7	11.7 tr|V8NQC0|V8NQC0_OPHHA Ectonucleoside triphosphate diphosphohydrolase 5 (Fragm	OPHHA	2
2655	2.75	2.76	51.71	18	5.143 tr|V8NTD5|V8NTD5_OPHHA ATP-dependent RNA helicase DHX8 (Fragment) OS=Ophiophag	OPHHA	1
2656	2.75	2.75	42.08	7.415	5.411 tr|V8P255|V8P255_OPHHA Rap1 GTPase-activating protein 2 (Fragment) OS=Ophiopha	OPHHA	2
2657	2.75	2.75	39.75	15.57	6.557 tr|V8PJ61|V8PJ61_OPHHA Ypr1 (Fragment) OS=Ophiophagus hannah GN=YPR1 PE=4 SV=1	OPHHA	1
2658	2.74	2.74	23.7	3.179	1.156 tr|V8NYE7|V8NYE7_OPHHA Histone deacetylase complex subunit OS=Ophiophagus hann	OPHHA	1
2659	2.74	2.74	44.31	7.927	6.098 tr|V8PCT8|V8PCT8_OPHHA Protein mab-21-like 3 (Fragment) OS=Ophiophagus hannah	OPHHA	2
2660	2.74	2.74	62.63	10.32	5.694 tr|V8N537|V8N537_OPHHA Glyoxalase domain-containing protein 4 (Fragment) OS=Op	OPHHA	1
2661	2.74	2.74	31.28	4.112	4.112 tr|V8P8N0|V8P8N0_OPHHA Gigaxonin (Fragment) OS=Ophiophagus hannah GN=GAN PE=4	OPHHA	2
2662	2.74	2.74	27.53	7.532	2.857 tr|V8NKJ3|V8NKJ3_OPHHA Phosphatidylserine synthase 2 (Fragment) OS=Ophiophagus	OPHHA	1
2663	2.74	2.74	37.09	15.89	15.89 tr|V8P3Z4|V8P3Z4_OPHHA NHP2-like protein 1 (Fragment) OS=Ophiophagus hannah GN	OPHHA	2

2664	2.73	2.73	58.33	12.08	12.08	tr|V8NNY3|V8NNY3_OPHHA	Phosducin-like protein 3 (Fragment) OS=Ophiophagus hann	OPHHA	2
2665	2.72	2.72	46.3	6.921	6.921	tr|V8PCJ3|V8PCJ3_OPHHA	BRCA1-associated protein OS=Ophiophagus hannah GN=BRAP	OPHHA	2
2666	2.72	2.72	63.79	16.67	8.046	tr|V8NXD5|V8NXD5_OPHHA	LDLR chaperone MESD (Fragment) OS=Ophiophagus hannah GN	OPHHA	1
2667	2.71	2.71	71.82	13.39	3.002	tr|V8NIE4|V8NIE4_OPHHA	UDP-glucose 6-dehydrogenase OS=Ophiophagus hannah GN=UG	OPHHA	1
2668	2.71	2.71	34.95	15.22	11.07	tr|V8NSW9|V8NSW9_OPHHA	Syntaxin-3 (Fragment) OS=Ophiophagus hannah GN=Stx3 PE=	OPHHA	3
2669	2.71	2.71	40	19.17	8.75	tr|V8NEP9|V8NEP9_OPHHA	COP9 signalosome complex subunit 6 (Fragment) OS=Ophiop	OPHHA	1
2670	2.7	2.7	53.16	20.25	20.25	tr|V8ND61|V8ND61_OPHHA	MACRO domain-containing protein 2 (Fragment) OS=Ophioph	OPHHA	2
2671	2.69	2.69	66.9	23.45	7.586	tr|V8NX77|V8NX77_OPHHA	Ras-related protein Rab-12 (Fragment) OS=Ophiophagus ha	OPHHA	1
2672	2.69	2.69	48.94	22.34	9.574	tr|V8NT30|V8NT30_OPHHA	Receptor-binding cancer antigen expressed on SiSo cells	OPHHA	1
2673	2.68	2.68	47.82	2.909	2.364	tr|V8NIT4|V8NIT4_OPHHA	Membrane-associated guanylate kinase, WW and PDZ domain	OPHHA	2
2674	2.68	2.68	32.82	12.27	12.27	tr|V8NWX1|V8NWX1_OPHHA	Endothelial cell-selective adhesion molecule (Fragment)	OPHHA	2
2675	2.68	2.68	57.46	23.13	15.67	tr|V8NQN3|V8NQN3_OPHHA	Apolipoprotein O-like protein (Fragment) OS=Ophiophagus	OPHHA	1
2676	2.68	2.68	46.67	14.76	14.76	tr|V8PDJ3|V8PDJ3_OPHHA	Ras-related protein Rab-24 (Fragment) OS=Ophiophagus ha	OPHHA	2
2677	2.67	2.67	31.21	6.667	6.667	tr|V8NY94|V8NY94_OPHHA	F-box only protein 22 (Fragment) OS=Ophiophagus hannah	OPHHA	2
2678	2.67	2.67	42.02	10.92	10.92	tr|V8PC68|V8PC68_OPHHA	Deoxyuridine 5'-triphosphate nucleotidohydrolase, mitoc	OPHHA	2
2679	2.66	2.71	48.86	5.314	3.037	tr|V8PI66|V8PI66_OPHHA	Tyrosine-protein kinase BAZ1B (Fragment) OS=Ophiophagus	OPHHA	2
2680	2.66	2.66	57.85	3.723	3.723	tr|V8NSX1|V8NSX1_OPHHA	Zinc finger CCCH domain-containing protein 11A OS=Ophio	OPHHA	2
2681	2.66	2.66	34.3	4.042	1.963	tr|V8PE14|V8PE14_OPHHA	Myb-binding protein 1A OS=Ophiophagus hannah GN=MYBBP1A	OPHHA	2
2682	2.66	2.66	51.68	5.12	4	tr|V8NZN8|V8NZN8_OPHHA	Cleavage stimulation factor subunit 3 OS=Ophiophagus ha	OPHHA	2
2683	2.66	2.66	26.04	11.83	11.83	tr|V8NR59|V8NR59_OPHHA	Uncharacterized protein (Fragment) OS=Ophiophagus hanna	OPHHA	2
2684	2.65	2.65	34.07	16.3	9.63	tr|V8PBC8|V8PBC8_OPHHA	Fatty acid-binding protein, epidermal OS=Ophiophagus ha	OPHHA	1
2685	2.65	2.65	78.46	60	60	tr|V8P4L3|V8P4L3_OPHHA	Homogentisate 1,2-dioxygenase (Fragment) OS=Ophiophagus	OPHHA	2
2686	2.65	2.65	21.8	10.03	10.03	tr|V8ND07|V8ND07_OPHHA	Hydroxysteroid 11-beta-dehydrogenase 1-like protein B O	OPHHA	3
2687	2.64	2.64	44.8	4.461	4.461	tr|V8N9E8|V8N9E8_OPHHA	Histone-lysine N-methyltransferase, H3 lysine-79 specif	OPHHA	2
2688	2.64	2.64	34.69	11.73	9.949	tr|V8PFB7|V8PFB7_OPHHA	Golgi reassembly-stacking protein 1 (Fragment) OS=Ophio	OPHHA	3
2689	2.64	2.64	48.1	19.62	6.962	tr|V8PEU6|V8PEU6_OPHHA	Putative 7,8-dihydro-8-oxoguanine triphosphatase NUDT15	OPHHA	1
2690	2.63	2.93	40.32	3.402	1.379	tr|V8P9V8|V8P9V8_OPHHA	Plexin-D1 (Fragment) OS=Ophiophagus hannah GN=PLXND1 PE	OPHHA	2
2691	2.63	2.63	33.88	3.297	1.374	tr|V8P2G6|V8P2G6_OPHHA	Tetratricopeptide repeat protein 17 OS=Ophiophagus hann	OPHHA	1
2692	2.63	2.63	37.68	4.674	1.841	tr|V8P9X3|V8P9X3_OPHHA	FYN-binding protein (Fragment) OS=Ophiophagus hannah GN	OPHHA	1
2693	2.63	2.63	62.05	13.39	8.036	tr|V8PCP7|V8PCP7_OPHHA	60S ribosomal protein L37a (Fragment) OS=Ophiophagus ha	OPHHA	1
2694	2.63	2.63	67.19	18.75	18.75	tr|V8P4Z2|V8P4Z2_OPHHA	40S ribosomal protein S26 (Fragment) OS=Ophiophagus han	OPHHA	2
2695	2.63	2.63	14.55	7.121	7.121	tr|V8NQJ5|V8NQJ5_OPHHA	Ataxin-3 (Fragment) OS=Ophiophagus hannah GN=ATXN3 PE=4	OPHHA	2
2696	2.62	2.7	62.77	20	8.615	tr|V8N9I0|V8N9I0_OPHHA	Alpha-2-macroglobulin (Fragment) OS=Ophiophagus hannah	OPHHA	2
2697	2.62	2.65	52.83	4.357	1.307	tr|V8PHP8|V8PHP8_OPHHA	WD repeat-containing protein 3 (Fragment) OS=Ophiophagu	OPHHA	1
2698	2.62	2.62	54.33	6.557	2.81	tr|V8NK93|V8NK93_OPHHA	Putative arginyl-tRNA synthetase, mitochondrial (Fragme	OPHHA	1
2699	2.62	2.62	40.65	18.7	10.57	tr|V8NCA0|V8NCA0_OPHHA	60S ribosomal protein L35 OS=Ophiophagus hannah GN=RPL3	OPHHA	3
2700	2.61	2.62	63.74	6.977	1.903	tr|V8NPU9|V8NPU9_OPHHA	CAP-Gly domain-containing linker protein 1 (Fragment) O	OPHHA	1
2701	2.61	2.62	60	8.364	4.727	tr|V8P1Y9|V8P1Y9_OPHHA	39S ribosomal protein L19, mitochondrial OS=Ophiophagus	OPHHA	2
2702	2.61	2.61	55.81	6.058	4.866	tr|V8NHI3|V8NHI3_OPHHA	Insulin receptor substrate 2-A (Fragment) OS=Ophiophagu	OPHHA	3
2703	2.6	2.61	39.56	2.719	1.903	tr|V8P5Y8|V8P5Y8_OPHHA	Protein LAP2 (Fragment) OS=Ophiophagus hannah GN=ERBB2I	OPHHA	2
2704	2.6	2.6	79.62	10.03	10.03	tr|V8NVW0|V8NVW0_OPHHA	Pre-mRNA-splicing factor ISY1-like protein (Fragment) O	OPHHA	2
2705	2.6	2.6	58.95	5.234	5.234	tr|V8NPF2|V8NPF2_OPHHA	Replication factor C subunit 4 OS=Ophiophagus hannah GN	OPHHA	2
2706	2.6	2.6	61.9	19.73	6.803	tr|V8NRC7|V8NRC7_OPHHA	Transmembrane protein OS=Ophiophagus hannah GN=L345_101	OPHHA	1
2707	2.6	2.6	17.47	5.677	5.677	tr|V8PAS2|V8PAS2_OPHHA	Transcription factor Sp3 (Fragment) OS=Ophiophagus hann	OPHHA	3
2708	2.6	2.6	50.47	19.81	5.66	tr|V8N9K1|V8N9K1_OPHHA	Phospholysine phosphohistidine inorganic pyrophosphate	OPHHA	1
2709	2.6	2.6	54.42	11.95	11.95	tr|V8P0Y2|V8P0Y2_OPHHA	Proteasome assembly chaperone 1 OS=Ophiophagus hannah G	OPHHA	2
2710	2.6	2.6	62.73	40.91	25.45	tr|V8P3Q9|V8P3Q9_OPHHA	Leucyl-cystinyl aminopeptidase OS=Ophiophagus hannah GN	OPHHA	2
2711	2.59	2.59	23.34	2.087	1.661	tr|V8P8U8|V8P8U8_OPHHA	Neurogenic locus notch-like protein 3 (Fragment) OS=Oph	OPHHA	2
2712	2.59	2.59	46.8	10.42	2.011	tr|V8P356|V8P356_OPHHA	Vacuolar fusion protein MON1-like A (Fragment) OS=Ophio	OPHHA	1
2713	2.59	2.59	62.77	30.66	9.489	tr|V8NXV9|V8NXV9_OPHHA	ADP-ribosylation factor-like protein 6 OS=Ophiophagus h	OPHHA	1
2714	2.59	2.59	18.29	5.556	5.556	tr|V8P182|V8P182_OPHHA	Transmembrane protein 62 (Fragment) OS=Ophiophagus hann	OPHHA	2
2715	2.58	2.59	57.05	18.12	12.75	tr|V8PAJ1|V8PAJ1_OPHHA	Tubulin-specific chaperone A (Fragment) OS=Ophiophagus	OPHHA	1
2716	2.58	2.58	54.3	6.143	6.143	tr|V8PCE4|V8PCE4_OPHHA	Mothers against decapentaplegic homolog (Fragment) OS=O	OPHHA	2
2717	2.58	2.58	36	9.333	6	tr|V8NH41|V8NH41_OPHHA	Putative gamma-glutamyltransferase ywrD (Fragment) OS=O	OPHHA	1
2718	2.57	2.57	47.66	11.21	2.804	tr|V8P1S3|V8P1S3_OPHHA	Glutamine-dependent NAD(+) synthetase OS=Ophiophagus ha	OPHHA	1
2719	2.57	2.57	39.26	11.85	11.85	tr|V8PFI3|V8PFI3_OPHHA	Interferon regulatory factor 2-binding protein 2 (Fragm	OPHHA	2
2720	2.56	13.83	60.23	30.41	30.41	tr|V8N7N4|V8N7N4_OPHHA	Uncharacterized protein (Fragment) OS=Ophiophagus hanna	OPHHA	8
2721	2.56	2.57	27.66	12.77	9.362	tr|V8NC01|V8NC01_OPHHA	Exocyst complex component 4 (Fragment) OS=Ophiophagus h	OPHHA	2
2722	2.56	2.56	44.27	1.723	0.5942	tr|V8PD99|V8PD99_OPHHA	Dedicator of cytokinesis protein 9 OS=Ophiophagus hanna	OPHHA	1
2723	2.56	2.56	50.87	9.884	6.686	tr|V8PC49|V8PC49_OPHHA	Protein FAM49A (Fragment) OS=Ophiophagus hannah GN=FAM4	OPHHA	2
2724	2.56	2.56	27.11	3.815	2.41	tr|V8NTH9|V8NTH9_OPHHA	Monocarboxylate transporter 1 OS=Ophiophagus hannah GN=	OPHHA	2
2725	2.56	2.56	44.55	15.17	10.9	tr|V8PGP1|V8PGP1_OPHHA	1,2-dihydroxy-3-keto-5-methylthiopentene dioxygenase (F	OPHHA	2
2726	2.55	6.76	40.75	11.2	6.532	tr|V8P028|V8P028_OPHHA	CTP synthase OS=Ophiophagus hannah GN=CTPS2 PE=3 SV=1	OPHHA	3
2727	2.55	3.77	54.33	4.329	3.226	tr|V8NYM2|V8NYM2_OPHHA	Formin-like protein 1 (Fragment) OS=Ophiophagus hannah	OPHHA	3
2728	2.55	2.55	47.17	5.031	3.145	tr|V8P886|V8P886_OPHHA	Polypeptide N-acetylgalactosaminyltransferase (Fragment	OPHHA	1
2729	2.55	2.55	56.63	10.75	3.943	tr|V8ND35|V8ND35_OPHHA	Alpha-taxilin OS=Ophiophagus hannah GN=Txlna PE=4 SV=1	OPHHA	1
2730	2.55	2.55	27.08	10.42	5.729	tr|V8NIF9|V8NIF9_OPHHA	Mature T-cell proliferation 1 neighbor protein (Fragmen	OPHHA	1
2731	2.55	2.55	70.79	31.46	20.22	tr|V8NVU4|V8NVU4_OPHHA	G-protein-signaling modulator 1 (Fragment) OS=Ophiophag	OPHHA	1
2732	2.54	4.63	52.56	11.59	8.086	tr|V8NCN9|V8NCN9_OPHHA	Cytoplasmic dynein 1 light intermediate chain 1 (Fragme	OPHHA	2
2733	2.54	3.36	40.53	2.82	1.289	tr|V8NWX0|V8NWX0_OPHHA	Nesprin-2 (Fragment) OS=Ophiophagus hannah GN=SYNE2 PE=	OPHHA	2
2734	2.54	2.56	41.84	12.24	2.245	tr|V8NQG1|V8NQG1_OPHHA	Putative prolyl-tRNA synthetase, mitochondrial (Fragmen	OPHHA	1
2735	2.54	2.55	44.96	4.435	2.419	tr|V8NDG0|V8NDG0_OPHHA	Tensin-3 OS=Ophiophagus hannah GN=Tns3 PE=4 SV=1	OPHHA	1
2736	2.54	2.55	32.51	11.52	7.819	tr|V8PEC3|V8PEC3_OPHHA	Ectonucleoside triphosphate diphosphohydrolase 5 (Fragm	OPHHA	1
2737	2.54	2.54	42.02	4.141	2.761	tr|V8NPE8|V8NPE8_OPHHA	NCK-interacting protein with SH3 domain protein (Fragme	OPHHA	1
2738	2.54	2.54	46.84	14.35	5.485	tr|V8NDI6|V8NDI6_OPHHA	Leucine-rich repeat and calponin-likey domain-containin	OPHHA	1
2739	2.53	2.53	53.24	2.854	1.713	tr|V8PHM5|V8PHM5_OPHHA	DCN1-like protein (Fragment) OS=Ophiophagus hannah GN=a	OPHHA	2
2740	2.53	2.53	56.33	9.177	5.38	tr|V8P6V5|V8P6V5_OPHHA	Heparan sulfate 2-O-sulfotransferase 1 (Fragment) OS=Op	OPHHA	1
2741	2.52	2.9	21.73	2.817	1.408	tr|V8NPV2|V8NPV2_OPHHA	Phospholipid scramblase 1 (Fragment) OS=Ophiophagus han	OPHHA	1
2742	2.52	2.53	33.99	4.89	2.445	tr|V8PAA7|V8PAA7_OPHHA	Interferon regulatory factor 3 (Fragment) OS=Ophiophagu	OPHHA	1
2743	2.52	2.52	55.41	2.212	1.164	tr|V8PBD0|V8PBD0_OPHHA	Squamous cell carcinoma antigen recognized by T-cells 3	OPHHA	1
2744	2.52	2.52	35.93	2.464	1.351	tr|V8NU15|V8NU15_OPHHA	Period circadian protein-like 2 (Fragment) OS=Ophiophag	OPHHA	2
2745	2.52	2.52	28.61	3.399	1.841	tr|V8NBB9|V8NBB9_OPHHA	Olfactory receptor (Fragment) OS=Ophiophagus hannah GN=	OPHHA	2
2746	2.52	2.52	37.79	12.21	8.721	tr|V8NL85|V8NL85_OPHHA	HD domain-containing protein 2 OS=Ophiophagus hannah GN	OPHHA	1
2747	2.51	3.06	31.93	8.635	5.02	tr|V8PEX3|V8PEX3_OPHHA	Ribosomal protein S6 kinase beta-2 (Fragment) OS=Ophiop	OPHHA	2
2748	2.51	2.51	38.35	7.965	3.54	tr|V8NC24|V8NC24_OPHHA	E3 ubiquitin-protein ligase UBR4 (Fragment) OS=Ophiopha	OPHHA	1
2749	2.51	2.51	51.64	7.377	4.372	tr|V8NEQ0|V8NEQ0_OPHHA	Inositol polyphosphate 5-phosphatase K OS=Ophiophagus h	OPHHA	1
2750	2.51	2.51	32.29	11.11	7.128	tr|V8PDH8|V8PDH8_OPHHA	Non-syndromic hearing impairment protein 5-like protein	OPHHA	2
2751	2.5	2.5	43.1	2.132	0.8167	tr|V8NKF0|V8NKF0_OPHHA	Microtubule-associated protein 1B (Fragment) OS=Ophioph	OPHHA	1
2752	2.5	2.5	45.83	8.333	5.903	tr|V8P3Q5|V8P3Q5_OPHHA	39S ribosomal protein L4, mitochondrial (Fragment) OS=O	OPHHA	1
2753	2.5	2.5	53.62	12.56	7.729	tr|V8PI19|V8PI19_OPHHA	Dual specificity mitogen-activated protein kinase kinas	OPHHA	1
2754	2.5	2.5	29.11	10.8	5.634	tr|V8NEP1|V8NEP1_OPHHA	Upstream stimulatory factor 2 (Fragment) OS=Ophiophagus	OPHHA	1
2755	2.49	2.49	59.22	11.69	7.273	tr|V8PFY8|V8PFY8_OPHHA	Choline kinase alpha OS=Ophiophagus hannah GN=CHKA PE=4	OPHHA	2
2756	2.49	2.49	55.06	11.9	6.25	tr|V8PCZ6|V8PCZ6_OPHHA	Casein kinase I isoform alpha OS=Ophiophagus hannah GN=	OPHHA	1
2757	2.49	2.49	70.15	14.03	5.373	tr|V8NGG4|V8NGG4_OPHHA	Plexin-A1 (Fragment) OS=Ophiophagus hannah GN=PLXNA1 PE	OPHHA	1
2758	2.49	2.49	50.73	10.24	6.341	tr|V8NJT9|V8NJT9_OPHHA	Retinol dehydrogenase 11 (Fragment) OS=Ophiophagus hann	OPHHA	1
2759	2.49	2.49	36.28	19.47	11.5	tr|V8NCQ0|V8NCQ0_OPHHA	Long-chain-fatty-acid--CoA ligase 5 (Fragment) OS=Ophio	OPHHA	1

2760	2.49	2.49	22.22	22.22	11.9	tr|V8PC24|V8PC24_OPHHA	Protein C10 (Fragment) OS=Ophiophagus hannah GN=L345 02	OPHHA	1
2761	2.48	2.48	40.83	6.846	3.912	tr|V8NX89|V8NX89_OPHHA	Glycosaminoglycan xylosylkinase OS=Ophiophagus hannah G	OPHHA	1
2762	2.48	2.48	54.96	30.53	15.27	tr|V8NCY5|V8NCY5_OPHHA	Uncharacterized protein (Fragment) OS=Ophiophagus hanna	OPHHA	1
2763	2.47	2.52	26.15	5.827	1.626	tr|V8PH29|V8PH29_OPHHA	Semaphorin-4G (Fragment) OS=Ophiophagus hannah GN=SEMA4	OPHHA	1
2764	2.47	2.48	34.52	7.565	2.6	tr|V8P7T2|V8P7T2_OPHHA	WD repeat domain phosphoinositide-interacting protein 4	OPHHA	1
2765	2.47	2.47	27.09	3.206	1.138	tr|V8PA96|V8PA96_OPHHA	MHC class II regulatory factor RFX1 (Fragment) OS=Ophio	OPHHA	1
2766	2.47	2.47	67.23	25.99	6.215	tr|V8PIN2|V8PIN2_OPHHA	Four and a half LIM domains protein 2 OS=Ophiophagus ha	OPHHA	1
2767	2.47	2.47	29.47	10.14	5.797	tr|V8NID2|V8NID2_OPHHA	Putative asparaginyl-tRNA synthetase, mitochondrial (Fr	OPHHA	1
2768	2.47	2.47	48.68	27.63	27.63	tr|V8N6C0|V8N6C0_OPHHA	Uncharacterized protein (Fragment) OS=Ophiophagus hanna	OPHHA	2
2769	2.46	4.89	54.13	11.65	7.282	tr|V8PBK4|V8PBK4_OPHHA	SWI/SNF-related matrix-associated actin-dependent regul	OPHHA	2
2770	2.46	2.47	44.61	7.353	3.922	tr|V8P4Y3|V8P4Y3_OPHHA	Histone acetyltransferase type B catalytic subunit (Fra	OPHHA	1
2771	2.46	2.46	47.32	7.229	1.533	tr|V8PHZ0|V8PHZ0_OPHHA	Uncharacterized protein (Fragment) OS=Ophiophagus hanna	OPHHA	1
2772	2.46	2.46	32.37	7.629	3.918	tr|V8NTL7|V8NTL7_OPHHA	Inosine-5'-monophosphate dehydrogenase 2 (Fragment) OS=	OPHHA	2
2773	2.45	2.45	37.54	10.92	10.92	tr|V8PI55|V8PI55_OPHHA	Serine/threonine-protein kinase N2 (Fragment) OS=Ophiop	OPHHA	3
2774	2.45	2.45	52.33	26.74	15.12	tr|V8N9K2|V8N9K2_OPHHA	Uncharacterized protein (Fragment) OS=Ophiophagus hanna	OPHHA	1
2775	2.44	9.52	44.94	12.43	9.947	tr|V8N563|V8N563_OPHHA	Receptor-type tyrosine-protein phosphatase S (Fragment)	OPHHA	6
2776	2.44	2.5	47.8	4.545	2.893	tr|V8NF12|V8NF12_OPHHA	Kinesin-like protein (Fragment) OS=Ophiophagus hannah G	OPHHA	2
2776	0.02	1.21	56.83	4.781	1.503	tr|V8P1F2|V8P1F2_OPHHA	Kinesin-like protein (Fragment) OS=Ophiophagus hannah G	OPHHA	1
2777	2.44	2.44	67.17	10.1	7.071	tr|V8NDR7|V8NDR7_OPHHA	SEC14-like protein 2 (Fragment) OS=Ophiophagus hannah G	OPHHA	2
2778	2.44	2.44	36.98	16.67	6.771	tr|V8PA09|V8PA09_OPHHA	Histone H1.0 (Fragment) OS=Ophiophagus hannah GN=H1f0 P	OPHHA	1
2779	2.43	13.02	34.35	21.12	12.21	tr|V8N8S7|V8N8S7_OPHHA	Cytochrome protein OS=Ophiophagus hannah GN=cyp2k1 PE=3	OPHHA	7
2780	2.43	8.7	42.28	5.541	3.751	tr|V8NRS5|V8NRS5_OPHHA	von Willebrand factor A domain-containing protein 5A (F	OPHHA	4
2781	2.43	2.91	37.83	11.98	3.232	tr|V8NMB2|V8NMB2_OPHHA	Serine/threonine-protein phosphatase 2B catalytic subun	OPHHA	1
2782	2.43	2.43	51.34	3.476	1.896	tr|V8PD79|V8PD79_OPHHA	Neutral and basic amino acid transport protein rBAT (Fr	OPHHA	1
2783	2.43	2.43	47.06	7.294	3.059	tr|V8NY68|V8NY68_OPHHA	Cytosolic Fe-S cluster assembly factor NARFL (Fragment)	OPHHA	1
2784	2.43	2.43	35.21	4.475	2.529	tr|V8PBW3|V8PBW3_OPHHA	Periodic tryptophan protein 1-like protein (Fragment) O	OPHHA	1
2785	2.43	2.43	27.6	16.15	12.5	tr|V8NDG7|V8NDG7_OPHHA	Biogenesis of lysosome-related organelles complex 1 sub	OPHHA	1
2786	2.42	2.42	53.73	5.422	1.807	tr|V8NLR5|V8NLR5_OPHHA	Serine/arginine repetitive matrix protein 1 OS=Ophiopha	OPHHA	1
2787	2.42	2.42	36.14	4.028	1.185	tr|V8N8I1|V8N8I1_OPHHA	Uncharacterized protein (Fragment) OS=Ophiophagus hanna	OPHHA	1
2788	2.42	2.42	38.64	11.74	4.924	tr|V8NJ05|V8NJ05_OPHHA	Translational activator of cytochrome c oxidase 1 (Frag	OPHHA	1
2789	2.42	2.42	29.34	9.266	5.019	tr|V8PI83|V8PI83_OPHHA	Metaxin-2 OS=Ophiophagus hannah GN=MTX2 PE=4 SV=1	OPHHA	1
2790	2.42	2.42	50	19.01	19.01	tr|V8PDG6|V8PDG6_OPHHA	Dermatopontin (Fragment) OS=Ophiophagus hannah GN=DPT P	OPHHA	2
2791	2.41	2.43	52.16	4.795	2.69	tr|V8PIQ8|V8PIQ8_OPHHA	Serine/threonine-protein kinase 10 (Fragment) OS=Ophiop	OPHHA	2
2792	2.41	2.41	52.96	9.259	6.667	tr|V8N9F9|V8N9F9_OPHHA	Protein canopy-like 3 OS=Ophiophagus hannah GN=CNPY3 PE	OPHHA	2
2793	2.41	2.41	36.86	11.26	7.85	tr|V8P5Z5|V8P5Z5_OPHHA	Geranylgeranyl pyrophosphate synthase (Fragment) OS=Oph	OPHHA	2
2794	2.41	2.41	63.56	22.88	11.02	tr|V8N5J3|V8N5J3_OPHHA	Uncharacterized protein (Fragment) OS=Ophiophagus hanna	OPHHA	1
2795	2.41	2.41	79.03	18.55	18.55	tr|V8N687|V8N687_OPHHA	Uncharacterized protein (Fragment) OS=Ophiophagus hanna	OPHHA	2
2796	2.4	2.48	37.51	2.376	0.7707	tr|V8P1R6|V8P1R6_OPHHA	NLR family member X1 OS=Ophiophagus hannah GN=Nlrx1 PE=	OPHHA	1
2797	2.4	2.46	35.45	4.108	1.761	tr|V8NX25|V8NX25_OPHHA	Reversion-inducing cysteine-rich protein with Kazal mot	OPHHA	1
2798	2.4	2.4	35.4	4.416	0.9972	tr|V8NVM6|V8NVM6_OPHHA	Zinc finger FYVE domain-containing protein 16 (Fragment	OPHHA	1
2799	2.4	2.4	33.69	7.082	3.004	tr|V8NW18|V8NW18_OPHHA	Nucleotide exchange factor SIL1 OS=Ophiophagus hannah G	OPHHA	1
2800	2.4	2.4	57.78	33.33	33.33	tr|V8PCI2|V8PCI2_OPHHA	Small VCP/p97-interacting protein OS=Ophiophagus hannah	OPHHA	1
2801	2.39	2.98	74.58	20.83	7.917	tr|V8PFC7|V8PFC7_OPHHA	Phosphatidylinositol transfer protein beta isoform OS=O	OPHHA	2
2802	2.39	2.39	32.62	8.156	4.61	tr|V8PI97|V8PI97_OPHHA	Collectin-11 (Fragment) OS=Ophiophagus hannah GN=COLEC1	OPHHA	1
2803	2.39	2.39	67.92	16.98	11.95	tr|V8NGS1|V8NGS1_OPHHA	Sortilin-related receptor (Fragment) OS=Ophiophagus han	OPHHA	1
2804	2.39	2.39	61.86	13.4	11.34	tr|V8PEH2|V8PEH2_OPHHA	Cytochrome c oxidase assembly protein COX16-like, mitoc	OPHHA	1
2805	2.38	2.38	40.95	4.095	4.095	tr|V8PJ28|V8PJ28_OPHHA	Regulatory-associated protein of mTOR (Fragment) OS=Oph	OPHHA	2
2806	2.38	2.38	70.81	18.32	4.969	tr|V8P503|V8P503_OPHHA	WD repeat-containing protein 92 (Fragment) OS=Ophiophag	OPHHA	1
2807	2.38	2.38	69.29	15.77	6.224	tr|V8N8D7|V8N8D7_OPHHA	Uncharacterized protein (Fragment) OS=Ophiophagus hanna	OPHHA	1
2808	2.38	2.38	79.25	33.02	13.21	tr|V8PGA7|V8PGA7_OPHHA	Argininosuccinate lyase (Fragment) OS=Ophiophagus hanna	OPHHA	2
2809	2.38	2.38	64.24	17.22	12.58	tr|V8N3I6|V8N3I6_OPHHA	Uncharacterized protein (Fragment) OS=Ophiophagus hanna	OPHHA	1
2810	2.38	2.38	9.504	9.504	5.372	tr|V8PJM6|V8PJM6_OPHHA	Bax inhibitor 1 (Fragment) OS=Ophiophagus hannah GN=TMB	OPHHA	1
2811	2.37	2.41	79.08	21.43	12.76	tr|V8P304|V8P304_OPHHA	Calcium/calmodulin-dependent protein kinase type II sub	OPHHA	1
2812	2.37	2.38	32.26	4.274	2.137	tr|V8NRP1|V8NRP1_OPHHA	Plasma alpha-L-fucosidase OS=Ophiophagus hannah GN=FUCA	OPHHA	1
2813	2.37	2.37	34.3	2.463	0.9675	tr|V8PCM9|V8PCM9_OPHHA	Stonin-2 (Fragment) OS=Ophiophagus hannah GN=Ston2 PE=4	OPHHA	1
2814	2.37	2.37	26.19	1.777	1.029	tr|V8PDL3|V8PDL3_OPHHA	Tyrosine-protein kinase receptor Tie-1 (Fragment) OS=Op	OPHHA	1
2815	2.37	2.37	62.43	15.03	10.4	tr|V8PHZ6|V8PHZ6_OPHHA	Adrenodoxin OS=Ophiophagus hannah GN=FDX1 PE=4 SV=1	OPHHA	2
2816	2.37	2.37	38.86	11.4	5.699	tr|V8NN33|V8NN33_OPHHA	Uncharacterized protein (Fragment) OS=Ophiophagus hanna	OPHHA	1
2817	2.36	4.7	50.81	28.11	21.62	tr|V8NNT8|V8NNT8_OPHHA	Chromobox protein-like 1 OS=Ophiophagus hannah GN=CBX1	OPHHA	3
2818	2.36	2.38	38.49	3.152	0.7331	tr|V8P6N0|V8P6N0_OPHHA	Baculoviral IAP repeat-containing protein 1 (Fragment)	OPHHA	1
2819	2.36	2.37	55.81	43.02	32.56	tr|V8NT56|V8NT56_OPHHA	Splicing factor 3B subunit 5 (Fragment) OS=Ophiophagus	OPHHA	2
2820	2.36	2.36	37.33	21.33	8.667	tr|V8P375|V8P375_OPHHA	Uncharacterized protein (Fragment) OS=Ophiophagus hanna	OPHHA	1
2821	2.36	2.36	28.86	11.41	5.369	tr|V8NLQ4|V8NLQ4_OPHHA	Bis(5'-nucleosyl)-tetraphosphatase (Fragment) OS=Ophiop	OPHHA	1
2822	2.35	2.36	55.56	25.56	14.44	tr|V8NOU1|V8NOU1_OPHHA	Uncharacterized protein (Fragment) OS=Ophiophagus hanna	OPHHA	1
2823	2.35	2.35	32.26	7.4	3.226	tr|V8NBT8|V8NBT8_OPHHA	PX domain-containing protein kinase-like protein OS=Oph	OPHHA	1
2824	2.35	2.35	58.2	22.95	11.48	tr|V8PEQ7|V8PEQ7_OPHHA	Pre-mRNA branch site protein p14 (Fragment) OS=Ophiopha	OPHHA	1
2825	2.35	2.35	43.68	11.58	6.316	tr|V8NZM0|V8NZM0_OPHHA	Integrin alpha-5 (Fragment) OS=Ophiophagus hannah GN=it	OPHHA	1
2826	2.35	2.35	68.09	55.32	25.53	tr|V8N327|V8N327_OPHHA	Uncharacterized protein (Fragment) OS=Ophiophagus hanna	OPHHA	1
2827	2.35	2.35	71.43	46.75	36.36	tr|V8N9V2|V8N9V2_OPHHA	Cysteine-rich protein 1 OS=Ophiophagus hannah GN=CRIP1	OPHHA	1
2828	2.34	2.34	52.59	30.37	14.81	tr|V8P5Z9|V8P5Z9_OPHHA	Transmembrane protein OS=Ophiophagus hannah GN=TMEM222	OPHHA	1
2829	2.33	3.24	61.65	12.09	4.425	tr|V8PIG0|V8PIG0_OPHHA	Exocyst complex component 6 (Fragment) OS=Ophiophagus h	OPHHA	3
2830	2.33	2.35	22.95	4.825	2.339	tr|V8NN63|V8NN63_OPHHA	Protein angel-like 1 (Fragment) OS=Ophiophagus hannah G	OPHHA	1
2831	2.33	2.33	70.35	14.07	8.04	tr|V8NUW1|V8NUW1_OPHHA	Sulfhydryl oxidase (Fragment) OS=Ophiophagus hannah GN=	OPHHA	1
2832	2.33	2.33	84.68	19.82	19.82	tr|V8NHU7|V8NHU7_OPHHA	Mitochondrial import inner membrane translocase subunit	OPHHA	2
2833	2.33	2.33	40.99	8.14	8.14	tr|V8PDW5|V8PDW5_OPHHA	FGFR1 oncogene partner (Fragment) OS=Ophiophagus hannah	OPHHA	2
2834	2.32	2.33	49.71	5.882	3.824	tr|V8NHX5|V8NHX5_OPHHA	Apoptosis regulator BAX (Fragment) OS=Ophiophagus hanna	OPHHA	1
2835	2.32	2.32	31.67	1.663	0.998	tr|V8P4B6|V8P4B6_OPHHA	HEAT repeat-containing protein 1 (Fragment) OS=Ophiopha	OPHHA	1
2836	2.32	2.32	73.79	9.972	4.558	tr|V8NTW7|V8NTW7_OPHHA	Uncharacterized protein (Fragment) OS=Ophiophagus hanna	OPHHA	1
2837	2.32	2.32	39.79	4.844	2.768	tr|V8P3W9|V8P3W9_OPHHA	Fucose-1-phosphate guanylyltransferase (Fragment) OS=Op	OPHHA	1
2838	2.32	2.32	26.81	5.094	3.217	tr|V8PG55|V8PG55_OPHHA	Polyadenylate-binding protein-interacting protein 1 (Fr	OPHHA	1
2839	2.32	2.32	15.95	5.063	5.063	tr|V8N6Y9|V8N6Y9_OPHHA	Uncharacterized protein (Fragment) OS=Ophiophagus hanna	OPHHA	2
2840	2.31	2.32	19.35	8.602	5.018	tr|V8NBV8|V8NBV8_OPHHA	Exocyst complex component 6B OS=Ophiophagus hannah GN=E	OPHHA	1
2841	2.3	2.3	71.1	5.255	2.299	tr|V8PI57|V8PI57_OPHHA	Putative sodium-coupled neutral amino acid transporter	OPHHA	1
2842	2.3	2.3	24.96	0.7849	0.7849	tr|V8NGE7|V8NGE7_OPHHA	Fibrillin-2 (Fragment) OS=Ophiophagus hannah GN=FBN2 PE	OPHHA	2
2843	2.3	2.3	41.54	3.532	1.208	tr|V8NTM3|V8NTM3_OPHHA	Dentin matrix protein 4 (Fragment) OS=Ophiophagus hanna	OPHHA	1
2844	2.3	2.3	63.52	12.38	12.38	tr|V8NDW7|V8NDW7_OPHHA	Protein BUD31-like protein (Fragment) OS=Ophiophagus ha	OPHHA	3
2845	2.3	2.3	62.5	26.04	15.63	tr|V8N5T7|V8N5T7_OPHHA	Rab GDP dissociation inhibitor beta (Fragment) OS=Ophio	OPHHA	1
2846	2.29	12.54	51.4	18.92	17.63	tr|V8PG33|V8PG33_OPHHA	Uncharacterized protein (Fragment) OS=Ophiophagus hanna	OPHHA	6
2847	2.29	2.31	46.68	6.463	2.693	tr|V8NVN4|V8NVN4_OPHHA	TBC1 domain family member 24 (Fragment) OS=Ophiophagus	OPHHA	1
2848	2.29	2.29	39.31	2.659	1.734	tr|V8NWW8|V8NWW8_OPHHA	Oxysterol-binding protein (Fragment) OS=Ophiophagus han	OPHHA	1
2849	2.29	2.29	40.68	3.215	2.251	tr|V8PHL9|V8PHL9_OPHHA	Growth arrest-specific protein 6 (Fragment) OS=Ophiopha	OPHHA	1
2850	2.29	2.29	28.18	5.925	2.312	tr|V8P5Y2|V8P5Y2_OPHHA	Sodium/hydrogen exchanger (Fragment) OS=Ophiophagus han	OPHHA	1
2851	2.29	2.29	60.91	17.77	9.645	tr|V8PIL8|V8PIL8_OPHHA	Vesicle transport protein SEC20 OS=Ophiophagus hannah G	OPHHA	1
2852	2.29	2.29	36.34	11.34	2.835	tr|V8NM69|V8NM69_OPHHA	Forkhead box protein A2 (Fragment) OS=Ophiophagus hanna	OPHHA	1
2853	2.29	2.29	96.43	36.9	36.9	tr|V8NJY5|V8NJY5_OPHHA	Phosphatidylinositol 3-kinase catalytic subunit type 3	OPHHA	2
2854	2.29	2.29	22.36	7.595	7.595	tr|V8P850|V8P850_OPHHA	Uroporphyrinogen-III synthase (Fragment) OS=Ophiophagus	OPHHA	2

2855	2.29	2.29	18.64	18.64	13.56	tr|V8N6X3|V8N6X3_OPHHA	Ectonucleotide pyrophosphatase/phosphodiesterase family	OPHHA	1
2856	2.28	8.66	46.41	8.146	6.324	tr|V8ND84|V8ND84_OPHHA	Plasma membrane calcium-transporting ATPase 2 (Fragment	OPHHA	4
2856	0.04	8.33	35.76	8.565	6.829	tr|V8NFL1|V8NFL1_OPHHA	Calcium-transporting ATPase OS=Ophiophagus hannah GN=AT	OPHHA	4
2857	2.28	2.28	60.95	12.7	9.206	tr|V8NRV6|V8NRV6_OPHHA	MAP kinase-activated protein kinase 2 (Fragment) OS=Oph	OPHHA	2
2858	2.28	2.28	62.14	12.62	6.796	tr|V8NJD7|V8NJD7_OPHHA	Uncharacterized protein OS=Ophiophagus hannah GN=L345_1	OPHHA	1
2859	2.28	2.28	90	13.53	7.647	tr|V8NH14|V8NH14_OPHHA	Protein rogdi-like protein (Fragment) OS=Ophiophagus ha	OPHHA	1
2860	2.28	2.28	38.75	10.75	3.75	tr|V8NDN1|V8NDN1_OPHHA	Dystrophin (Fragment) OS=Ophiophagus hannah GN=DMD PE=4	OPHHA	1
2861	2.27	4.46	68.11	18.94	11.63	tr|V8NJ59|V8NJ59_OPHHA	Calcium/calmodulin-dependent protein kinase type 1D OS=	OPHHA	3
2862	2.27	2.56	38.31	10.14	3.662	tr|V8P366|V8P366_OPHHA	[Protein ADP-ribosylarginine] hydrolase OS=Ophiophagus	Protein	1
2863	2.27	2.31	59.34	24.48	7.054	tr|V8PC40|V8PC40_OPHHA	DnaJ-like subfamily A member 3, mitochondrial OS=Ophiop	OPHHA	1
2864	2.26	2.26	39.62	10.38	5.896	tr|V8PA73|V8PA73_OPHHA	F-box/LRR-repeat protein 20 (Fragment) OS=Ophiophagus h	OPHHA	2
2865	2.26	2.26	62.58	29.45	8.589	tr|V8NDK1|V8NDK1_OPHHA	39S ribosomal protein L49, mitochondrial (Fragment) OS=	OPHHA	1
2866	2.26	2.26	37.41	12.22	5.185	tr|V8N9W2|V8N9W2_OPHHA	Myotubularin-related protein 1 (Fragment) OS=Ophiophagu	OPHHA	1
2867	2.26	2.26	43.4	8.805	8.805	tr|V8PI85|V8PI85_OPHHA	Exostosin-like 2 OS=Ophiophagus hannah GN=Extl2 PE=4 SV	OPHHA	2
2868	2.26	2.26	48.31	13.53	9.662	tr|V8NCS7|V8NCS7_OPHHA	Multiple inositol polyphosphate phosphatase 1 (Fragment	OPHHA	1
2869	2.25	3.32	33.8	5.429	2.102	tr|V8NSX5|V8NSX5_OPHHA	Pumilio-like 1 OS=Ophiophagus hannah GN=PUM1 PE=4 SV=1	OPHHA	2
2870	2.25	2.25	50.65	4.748	1.727	tr|V8NZ00|V8NZ00_OPHHA	Protein Hook-like 1 OS=Ophiophagus hannah GN=HOOK1 PE=4	OPHHA	1
2871	2.25	2.25	54.55	10.91	6.818	tr|V8NCQ8|V8NCQ8_OPHHA	Uncharacterized protein (Fragment) OS=Ophiophagus hanna	OPHHA	1
2872	2.25	2.25	32.56	4.157	2.54	tr|V8NB83|V8NB83_OPHHA	Phosphofurin acidic cluster sorting protein 1 (Fragment	OPHHA	1
2873	2.25	2.25	89.81	22.22	14.81	tr|V8NR91|V8NR91_OPHHA	Coatomer subunit alpha (Fragment) OS=Ophiophagus hannah	OPHHA	1
2874	2.24	3	42.16	5.882	3.922	tr|V8P380|V8P380_OPHHA	Calpain-5 (Fragment) OS=Ophiophagus hannah GN=CAPN5 PE=	OPHHA	2
2875	2.24	2.24	41.8	3.88	1.627	tr|V8NZF8|V8NZF8_OPHHA	Mitogen-activated protein kinase kinase kinase 5	OPHHA	1
2876	2.24	2.24	37.08	7.295	3.343	tr|V8NXV2|V8NXV2_OPHHA	Prostaglandin reductase 1 OS=Ophiophagus hannah GN=Ptgr	OPHHA	1
2877	2.24	2.24	35.06	4.329	3.03	tr|V8PDU5|V8PDU5_OPHHA	Splicing factor 3B subunit 4 OS=Ophiophagus hannah GN=S	OPHHA	1
2878	2.24	2.24	25.54	10.22	2.957	tr|V8NPJ6|V8NPJ6_OPHHA	Peroxisomal biogenesis factor 3 (Fragment) OS=Ophiophag	OPHHA	1
2879	2.23	4.08	58.17	7.692	5.048	tr|V8NZM9|V8NZM9_OPHHA	Keratin, type II cytoskeletal 8 OS=Ophiophagus hannah G	OPHHA	2
2880	2.23	2.23	49.92	2.276	1.22	tr|V8P3D6|V8P3D6_OPHHA	Pleckstrin-likey-like domain family B member 2 (Fragmen	OPHHA	1
2881	2.23	2.23	33.6	2.4	1.3	tr|V8P2E2|V8P2E2_OPHHA	Carboxypeptidase B (Fragment) OS=Ophiophagus hannah GN=	OPHHA	1
2882	2.22	2.43	61.64	6.347	3.103	tr|V8NUL4|V8NUL4_OPHHA	Kinesin-like protein (Fragment) OS=Ophiophagus hannah G	OPHHA	2
2883	2.22	2.27	29.23	5.275	3.516	tr|V8NU97|V8NU97_OPHHA	Ubiquitin-associated domain-containing protein 2 (Fragm	OPHHA	1
2884	2.22	2.24	43.46	11.21	3.738	tr|V8PAR6|V8PAR6_OPHHA	Sulfotransferase (Fragment) OS=Ophiophagus hannah GN=Su	OPHHA	1
2884	0.01	0.15	38.36	5.479	0	tr|V8N5K4|V8N5K4_OPHHA	Sulfotransferase (Fragment) OS=Ophiophagus hannah GN=Su	OPHHA	0
2885	2.22	2.22	69.34	3.416	1.025	tr|V8NDD7|V8NDD7_OPHHA	Rootletin (Fragment) OS=Ophiophagus hannah GN=CROCC PE=	OPHHA	2
2886	2.22	2.22	36.59	3.02	3.02	tr|V8PEK2|V8PEK2_OPHHA	Rho GTPase-activating protein 12 OS=Ophiophagus hannah	OPHHA	2
2887	2.22	2.22	45.42	8.779	4.198	tr|V8N5Q1|V8N5Q1_OPHHA	Uncharacterized protein (Fragment) OS=Ophiophagus hanna	OPHHA	1
2888	2.22	2.22	43.9	14.15	8.293	tr|V8NNW3|V8NNW3_OPHHA	39S ribosomal protein L9, mitochondrial (Fragment) OS=O	OPHHA	1
2889	2.22	2.22	42.56	11.79	7.179	tr|V8NMH8|V8NMH8_OPHHA	Septin-5 (Fragment) OS=Ophiophagus hannah GN=Sept5 PE=4	OPHHA	1
2889	0	2	34.1	2.682	2.682	tr|V8PH90|V8PH90_OPHHA	Septin-4 (Fragment) OS=Ophiophagus hannah GN=Sept4 PE=3	OPHHA	1
2890	2.22	2.22	31.03	22.41	18.97	tr|V8NZT8|V8NZT8_OPHHA	Signal peptidase complex subunit 3 OS=Ophiophagus hanna	OPHHA	2
2891	2.21	2.21	35.74	1.755	0.6849	tr|V8NJW2|V8NJW2_OPHHA	Death-inducer obliterator 1 (Fragment) OS=Ophiophagus h	OPHHA	1
2892	2.21	2.21	43.95	5.185	2.716	tr|V8P7I1|V8P7I1_OPHHA	Plexin-A1 (Fragment) OS=Ophiophagus hannah GN=PLXNA1 PE	OPHHA	1
2893	2.21	2.21	39.18	7.602	7.602	tr|V8NUI5|V8NUI5_OPHHA	Mitochondrial carnitine/acylcarnitine carrier protein (	OPHHA	1
2894	2.2	3.01	61.89	9.221	3.484	tr|V8NEB0|V8NEB0_OPHHA	Merlin (Fragment) OS=Ophiophagus hannah GN=NF2 PE=4 SV=	OPHHA	2
2895	2.2	2.31	38.82	3.522	1.149	tr|V8P0I8|V8P0I8_OPHHA	RRP12-like protein OS=Ophiophagus hannah GN=Rrp12 PE=4	OPHHA	1
2896	2.2	2.2	40.55	1.651	0.6116	tr|V8PCC0|V8PCC0_OPHHA	Spermatogenesis-associated protein 13 (Fragment) OS=Oph	OPHHA	1
2896	0.03	2.07	36.3	1.65	1.65	tr|V8P0Q2|V8P0Q2_OPHHA	Rho guanine nucleotide exchange factor 9 (Fragment) OS=	OPHHA	1
2896	0	2.01	55.03	1.972	1.972	tr|V8PBZ0|V8PBZ0_OPHHA	Rho guanine nucleotide exchange factor 4 (Fragment) OS=	OPHHA	1
2897	2.2	2.2	60.23	4.509	2.899	tr|V8NRP7|V8NRP7_OPHHA	Tudor domain-containing protein 3 (Fragment) OS=Ophioph	OPHHA	1
2898	2.2	2.2	59.86	5.735	2.33	tr|V8P4L4|V8P4L4_OPHHA	Elongator complex protein 3 (Fragment) OS=Ophiophagus h	OPHHA	1
2899	2.2	2.2	57.87	6.43	2.882	tr|V8NRW0|V8NRW0_OPHHA	BAG family molecular chaperone regulator 5 (Fragment) O	OPHHA	1
2900	2.2	2.2	51.74	9.13	5.217	tr|V8PBN7|V8PBN7_OPHHA	Ectonucleotide pyrophosphatase/phosphodiesterase family	OPHHA	1
2901	2.19	2.19	48.5	3.645	1.682	tr|V8NXG3|V8NXG3_OPHHA	S phase cyclin A-associated protein in the endoplasmic	OPHHA	1
2902	2.19	2.19	18.53	1.796	1.04	tr|V8N7U9|V8N7U9_OPHHA	Gem-associated protein 4 (Fragment) OS=Ophiophagus hann	OPHHA	1
2903	2.19	2.19	25.92	4.11	1.686	tr|V8N8Z9|V8N8Z9_OPHHA	Tenascin (Fragment) OS=Ophiophagus hannah GN=TNC PE=4 S	OPHHA	1
2904	2.19	2.19	33.33	5.975	5.975	tr|V8P5T2|V8P5T2_OPHHA	Ras-related GTP-binding protein B OS=Ophiophagus hannah	OPHHA	2
2905	2.18	2.18	42.73	5.954	2.102	tr|V8P6E4|V8P6E4_OPHHA	Pre-mRNA-processing factor 17 (Fragment) OS=Ophiophagus	OPHHA	1
2906	2.18	2.18	54.5	2.725	2.725	tr|V8N7R3|V8N7R3_OPHHA	Phospholipase D3 (Fragment) OS=Ophiophagus hannah GN=Pl	OPHHA	1
2907	2.18	2.18	29.88	5.102	2.915	tr|V8NV00|V8NV00_OPHHA	Choline transporter-like protein 5 (Fragment) OS=Ophiop	OPHHA	3
2908	2.18	2.18	49.68	7.419	5.161	tr|V8NPY2|V8NPY2_OPHHA	60S ribosomal protein L8 (Fragment) OS=Ophiophagus hann	OPHHA	1
2909	2.18	2.18	46.1	5.29	3.526	tr|V8NLI5|V8NLI5_OPHHA	Uncharacterized protein OS=Ophiophagus hannah GN=L345_1	OPHHA	1
2910	2.18	2.18	77.94	29.41	16.18	tr|V8NHT1|V8NHT1_OPHHA	BolA-like protein 2 OS=Ophiophagus hannah GN=BOLA2 PE=4	OPHHA	1
2911	2.17	4.33	38.02	9.505	6.139	tr|V8NQA1|V8NQA1_OPHHA	NAD(P) transhydrogenase, mitochondrial (Fragment) OS=Op	OPHHA	4
2912	2.17	2.17	38.3	3.254	2.003	tr|V8NKF3|V8NKF3_OPHHA	Gamma-tubulin complex component 2 (Fragment) OS=Ophioph	OPHHA	1
2913	2.17	2.17	42.75	4.51	3.137	tr|V8PH47|V8PH47_OPHHA	Phenylalanyl-tRNA synthetase alpha chain OS=Ophiophagus	OPHHA	1
2914	2.17	2.17	34.5	2.805	1.543	tr|V8P0A6|V8P0A6_OPHHA	Suppressor of tumorigenicity 14 protein-like protein (F	OPHHA	1
2915	2.17	2.17	53.13	8.854	5.208	tr|V8P4X7|V8P4X7_OPHHA	Cytochrome b-c1 complex subunit Rieske, mitochondrial (	OPHHA	1
2916	2.17	2.17	38.46	5.594	3.73	tr|V8NZH4|V8NZH4_OPHHA	Translation initiation factor eIF-2B subunit beta (Frag	OPHHA	1
2917	2.17	2.17	14.74	4.851	3.172	tr|V8NV11|V8NV11_OPHHA	Solute carrier family 40 member 1 OS=Ophiophagus hannah	OPHHA	1
2918	2.16	4.24	49.34	18.94	14.54	tr|V8N428|V8N428_OPHHA	Uncharacterized protein (Fragment) OS=Ophiophagus hanna	OPHHA	2
2919	2.16	2.2	33.56	9.885	3.678	tr|V8NZS8|V8NZS8_OPHHA	Nucleoporin Nup43 (Fragment) OS=Ophiophagus hannah GN=N	OPHHA	1
2920	2.16	2.16	62.64	2.983	2.041	tr|V8NV68|V8NV68_OPHHA	Adenylate kinase isoenzyme 6 (Fragment) OS=Ophiophagus	OPHHA	1
2921	2.16	2.16	45.74	3.369	3.369	tr|V8NDK2|V8NDK2_OPHHA	Focal adhesion kinase 1 (Fragment) OS=Ophiophagus hanna	OPHHA	1
2922	2.16	2.16	46.54	4.615	4.615	tr|V8NJN6|V8NJN6_OPHHA	CCR4-NOT transcription complex subunit 2 OS=Ophiophagus	OPHHA	2
2923	2.16	2.16	74.68	10.3	6.438	tr|V8N5T2|V8N5T2_OPHHA	Mitotic spindle assembly checkpoint protein MAD1 (Fragm	OPHHA	1
2924	2.16	2.16	60.38	12.26	6.604	tr|V8NMQ6|V8NMQ6_OPHHA	Thymidylate kinase OS=Ophiophagus hannah GN=DTYMK PE=3	OPHHA	1
2925	2.16	2.16	47.46	9.746	5.932	tr|V8NR48|V8NR48_OPHHA	Exosome complex component RRP40 (Fragment) OS=Ophiophag	OPHHA	1
2926	2.16	2.16	33.51	13.83	6.915	tr|V8NQ70|V8NQ70_OPHHA	PCTP-like protein (Fragment) OS=Ophiophagus hannah GN=S	OPHHA	1
2927	2.16	2.16	54.03	35.48	12.9	tr|V8P5A8|V8P5A8_OPHHA	Ragulator complex protein LAMTOR3 OS=Ophiophagus hannah	OPHHA	1
2928	2.15	2.18	35.38	6.388	4.423	tr|V8NUP1|V8NUP1_OPHHA	TBC1 domain family member 22A OS=Ophiophagus hannah GN=	OPHHA	1
2929	2.15	2.16	34.88	33.72	24.42	tr|V8N7T3|V8N7T3_OPHHA	NEDD8-conjugating enzyme Ubc12 OS=Ophiophagus hannah GN	OPHHA	2
2930	2.15	2.15	30.25	5.158	1.29	tr|V8P0R2|V8P0R2_OPHHA	RNA-binding protein 27 (Fragment) OS=Ophiophagus hannah	OPHHA	1
2931	2.15	2.15	40.49	3.766	2.072	tr|V8PA62|V8PA62_OPHHA	N6-adenosine-methyltransferase 70 kDa subunit (Fragment	OPHHA	1
2932	2.15	2.15	34.59	3.903	2.153	tr|V8NYD9|V8NYD9_OPHHA	Cadherin-5 OS=Ophiophagus hannah GN=Cdh5 PE=4 SV=1	OPHHA	1
2933	2.14	2.14	66.67	8.889	4.167	tr|V8NLB6|V8NLB6_OPHHA	Matrix metalloproteinase-14 (Fragment) OS=Ophiophagus h	OPHHA	1
2934	2.14	2.14	58.09	16.6	4.979	tr|V8PFZ3|V8PFZ3_OPHHA	Sulfatase-modifying factor 2 (Fragment) OS=Ophiophagus	OPHHA	1
2935	2.14	2.14	65.65	10.88	5.102	tr|V8NHN3|V8NHN3_OPHHA	UBX domain-containing protein 6 OS=Ophiophagus hannah G	OPHHA	1
2936	2.14	2.14	59.89	18.08	6.78	tr|V8NSW4|V8NSW4_OPHHA	A-kinase anchor protein 9 (Fragment) OS=Ophiophagus han	OPHHA	1
2937	2.14	2.14	34.75	7.958	5.57	tr|V8P514|V8P514_OPHHA	Uncharacterized protein OS=Ophiophagus hannah GN=L345_0	OPHHA	1
2938	2.14	2.14	55.56	21.69	11.64	tr|V8P268|V8P268_OPHHA	X-ray repair cross-complementing protein 5 (Fragment) O	OPHHA	2
2939	2.13	6.61	39.31	3.817	3.817	tr|V8NBM3|V8NBM3_OPHHA	Cytochrome protein (Fragment) OS=Ophiophagus hannah GN=	OPHHA	5
2940	2.13	2.16	41.72	5.317	3.067	tr|V8P1W0|V8P1W0_OPHHA	Conserved oligomeric Golgi complex subunit 8 OS=Ophioph	OPHHA	1
2941	2.13	2.13	26.68	3.236	1.189	tr|V8NHE3|V8NHE3_OPHHA	Huntingtin (Fragment) OS=Ophiophagus hannah GN=HTT PE=4	OPHHA	2
2942	2.13	2.13	44.4	6.422	6.422	tr|V8PEQ4|V8PEQ4_OPHHA	Mitochondrial ribonuclease P protein 3 (Fragment) OS=Op	OPHHA	2
2943	2.13	2.13	50	6.561	4.525	tr|V8P4B3|V8P4B3_OPHHA	Dipeptidase (Fragment) OS=Ophiophagus hannah GN=DPEP1 P	OPHHA	1
2944	2.13	2.13	45.77	10.92	10.92	tr|V8NPN7|V8NPN7_OPHHA	Isopentenyl-diphosphate Delta-isomerase 1 (Fragment) OS	OPHHA	2
2945	2.13	2.13	49.85	7.077	4	tr|V8N3U5|V8N3U5_OPHHA	Uncharacterized protein (Fragment) OS=Ophiophagus hanna	OPHHA	1

2946	2.13	2.13	33.45	7.117	4.982	tr|V8N3S6|V8N3S6_OPHHA COUP transcription factor 2 (Fragment) OS=Ophiophagus h	OPHHA	1
2947	2.13	2.13	77.02	15.53	6.832	tr|V8NVV0|V8NVV0_OPHHA Peptidyl-prolyl cis-trans isomerase OS=Ophiophagus hann	OPHHA	1
2948	2.13	2.13	33.5	15	5.5	tr|V8NJP9|V8NJP9_OPHHA Glia maturation factor gamma (Fragment) OS=Ophiophagus	OPHHA	1
2949	2.12	2.21	67.57	16.89	11.49	tr|V8P7Q4|V8P7Q4_OPHHA Putative tRNA threonylcarbamoyladenosine biosynthesis p	OPHHA	1
2950	2.12	2.13	27.82	8.815	6.061	tr|V8NRB5|V8NRB5_OPHHA Nucleolysin TIAR OS=Ophiophagus hannah GN=TIAL1 PE=4 SV	OPHHA	2
2951	2.12	2.12	35.48	3.337	1.446	tr|V8NRR7|V8NRR7_OPHHA 39S ribosomal protein L16, mitochondrial (Fragment) OS=	OPHHA	1
2952	2.12	2.12	42.23	8.495	3.641	tr|V8PET3|V8PET3_OPHHA Interferon-inducible GTPase 5 (Fragment) OS=Ophiophagus	OPHHA	2
2953	2.12	2.12	26.69	8.185	4.982	tr|V8PC87|V8PC87_OPHHA D-amino-acid oxidase OS=Ophiophagus hannah GN=DAO PE=4	OPHHA	1
2954	2.12	2.12	21.47	11.14	3.533	tr|V8NLJ2|V8NLJ2_OPHHA Sec1 family domain-containing protein 2 (Fragment) OS=O	OPHHA	1
2955	2.11	2.12	39.44	2.394	1.197	tr|V8NQT3|V8NQT3_OPHHA Nucleoporin-like protein (Fragment) OS=Ophiophagus hann	OPHHA	1
2956	2.11	2.11	48.09	1.911	1.911	tr|V8NCQ9|V8NCQ9_OPHHA YLP motif-containing protein 1 (Fragment) OS=Ophiophagu	OPHHA	2
2957	2.11	2.11	55.31	4.81	3.206	tr|V8NLH0|V8NLH0_OPHHA GTPase SLIP-GC (Fragment) OS=Ophiophagus hannah GN=L345	OPHHA	1
2958	2.11	2.11	51.01	4.212	3.12	tr|V8P329|V8P329_OPHHA Influenza virus NS1A-binding protein-like protein OS=Op	OPHHA	1
2959	2.11	2.11	43.13	5.213	2.844	tr|V8NJ01|V8NJ01_OPHHA Putative ATP-dependent RNA helicase DDX47 (Fragment) OS	OPHHA	1
2960	2.11	2.11	27.37	3.888	3.888	tr|V8NSV6|V8NSV6_OPHHA Lipase maturation factor (Fragment) OS=Ophiophagus hann	OPHHA	2
2961	2.11	2.11	41.68	7.559	7.559	tr|V8PDA8|V8PDA8_OPHHA Glypican-4 (Fragment) OS=Ophiophagus hannah GN=Gpc4 PE=	OPHHA	2
2962	2.11	2.11	47.34	10.64	6.383	tr|V8PBE9|V8PBE9_OPHHA Gamma-glutamylaminecyclotransferase (Fragment) OS=Ophio	OPHHA	1
2963	2.11	2.11	63.85	16.92	11.54	tr|V8NVK3|V8NVK3_OPHHA Rho guanine nucleotide exchange factor 2 (Fragment) OS=	OPHHA	1
2964	2.1	2.69	52.58	15.46	6.701	tr|V8PAD1|V8PAD1_OPHHA Protein lin-7-like B (Fragment) OS=Ophiophagus hannah G	OPHHA	1
2965	2.1	2.16	33.64	12.62	5.607	tr|V8PJB5|V8PJB5_OPHHA Prolyl-tRNA synthetase associated domain-containing pro	OPHHA	1
2966	2.1	2.1	58.44	4.174	2.541	tr|V8P614|V8P614_OPHHA WD and tetratricopeptide repeats protein 1 (Fragment) O	OPHHA	1
2967	2.1	2.1	37.61	3.058	3.058	tr|V8P9L7|V8P9L7_OPHHA Mitogen-activated protein kinase kinase kinase 7 (Fragm	OPHHA	1
2968	2.1	2.1	35.48	5.53	3.456	tr|V8NYJ5|V8NYJ5_OPHHA Protein FAN OS=Ophiophagus hannah GN=Nsmaf PE=4 SV=1	OPHHA	1
2969	2.1	2.1	37.6	8.696	3.581	tr|V8NPL8|V8NPL8_OPHHA Protein-lysine 6-oxidase (Fragment) OS=Ophiophagus hann	OPHHA	1
2970	2.1	2.1	27.61	4.565	3.261	tr|V8N9S6|V8N9S6_OPHHA Nuclear pore complex protein Nup98-Nup96 (Fragment) OS=	OPHHA	1
2971	2.1	2.1	65.57	14.75	9.836	tr|V8P6B8|V8P6B8_OPHHA Chloride intracellular channel protein 5 (Fragment) OS=	OPHHA	1
2972	2.09	2.09	37.63	5.376	2.688	tr|V8NUS3|V8NUS3_OPHHA Mitochondrial enolase superfamily member 1 (Fragment) O	OPHHA	1
2973	2.09	2.09	46.08	6.536	6.536	tr|V8N819|V8N819_OPHHA Pentatricopeptide repeat-containing protein 3, mitochon	OPHHA	2
2974	2.09	2.09	70.4	11.21	8.072	tr|V8P1A3|V8P1A3_OPHHA Sodium/potassium-transporting ATPase subunit beta OS=Op	OPHHA	1
2975	2.09	2.09	40.07	4.255	4.255	tr|V8NYT6|V8NYT6_OPHHA Protein diaphanous-like 2 (Fragment) OS=Ophiophagus han	OPHHA	1
2976	2.09	2.09	38.24	6.723	3.782	tr|V8P3F4|V8P3F4_OPHHA CMP-N-acetylneuraminate-beta-1,4-galactoside alpha-2,3-	OPHHA	1
2977	2.09	2.09	39.72	8.333	3.889	tr|V8P786|V8P786_OPHHA Protein kinase C iota type OS=Ophiophagus hannah GN=Prk	OPHHA	1
2978	2.09	2.09	67.24	20.69	17.24	tr|V8N2L8|V8N2L8_OPHHA Uncharacterized protein (Fragment) OS=Ophiophagus hanna	OPHHA	1
2979	2.09	2.09	24.11	12.05	8.036	tr|V8P5Z6|V8P5Z6_OPHHA Collagen alpha-1(XVIII) chain (Fragment) OS=Ophiophagus	OPHHA	1
2980	2.08	2.1	57.08	1.606	1.606	tr|V8PH86|V8PH86_OPHHA Differentially expressed in FDCP 6-like protein (Fragme	OPHHA	1
2981	2.08	2.08	41.2	3.052	2.035	tr|V8NLT7|V8NLT7_OPHHA 5'-3' exoribonuclease 2 (Fragment) OS=Ophiophagus hanna	OPHHA	1
2982	2.08	2.08	47.73	5.481	3.342	tr|V8P7N6|V8P7N6_OPHHA Serine/threonine-protein phosphatase 1 regulatory subun	OPHHA	1
2983	2.08	2.08	34.64	5.866	3.073	tr|V8PGW2|V8PGW2_OPHHA Protein HEXIM1 (Fragment) OS=Ophiophagus hannah GN=Hexi	OPHHA	1
2984	2.08	2.08	46.01	4.255	4.255	tr|V8NKP8|V8NKP8_OPHHA L-threonine 3-dehydrogenase, mitochondrial OS=Ophiophag	OPHHA	1
2985	2.08	2.08	44.65	13.21	13.21	tr|V8N5G8|V8N5G8_OPHHA Tyrosine-protein kinase transforming protein erbB (Frag	OPHHA	1
2986	2.07	2.85	47.94	2.578	2.026	tr|V8P2A0|V8P2A0_OPHHA Chromodomain-helicase-DNA-binding protein 3 (Fragment)	OPHHA	2
2987	2.07	2.09	50.26	3.665	2.356	tr|V8P6X4|V8P6X4_OPHHA Gamma-tubulin complex component 5 (Fragment) OS=Ophioph	OPHHA	1
2988	2.07	2.08	32.93	1.208	1.208	tr|V8NDT1|V8NDT1_OPHHA Cytochrome protein (Fragment) OS=Ophiophagus hannah GN=	OPHHA	1
2989	2.07	2.07	37.64	1.741	1.072	tr|V8P230|V8P230_OPHHA Laminin subunit gamma-3 (Fragment) OS=Ophiophagus hanna	OPHHA	1
2990	2.07	2.07	43.71	2.746	2.746	tr|V8NKB5|V8NKB5_OPHHA Protein LSM14-like A OS=Ophiophagus hannah GN=LSM14A PE	OPHHA	1
2991	2.07	2.07	45.32	2.709	2.709	tr|V8NH72|V8NH72_OPHHA 28S ribosomal protein S5, mitochondrial (Fragment) OS=O	OPHHA	1
2992	2.07	2.07	42.67	6.015	3.947	tr|V8NB88|V8NB88_OPHHA Eukaryotic elongation factor 2 kinase (Fragment) OS=Oph	OPHHA	1
2993	2.07	2.07	58.08	13.64	7.576	tr|V8NNM1|V8NNM1_OPHHA Uncharacterized protein (Fragment) OS=Ophiophagus hanna	OPHHA	1
2994	2.07	2.07	43.11	9.439	5.357	tr|V8P8S5|V8P8S5_OPHHA Exosome complex component RRP45 (Fragment) OS=Ophiophag	OPHHA	1
2995	2.07	2.07	36.81	5.521	3.374	tr|V8NY39|V8NY39_OPHHA Acyl-CoA-binding domain-containing protein 5 (Fragment)	OPHHA	1
2996	2.07	2.07	62.83	9.735	9.735	tr|V8P2W5|V8P2W5_OPHHA CDGSH iron-sulfur domain-containing protein 3, mitochon	OPHHA	1
2997	2.07	2.07	27.41	2.998	2.998	tr|V8P6V8|V8P6V8_OPHHA Protein SAAL1 (Fragment) OS=Ophiophagus hannah GN=SAAL1	OPHHA	1
2998	2.07	2.07	83.72	13.95	13.95	tr|V8NR14|V8NR14_OPHHA STIP1-likey and U box-containing protein 1 (Fragment) O	OPHHA	1
2999	2.07	2.07	38.89	11.11	7.143	tr|V8NQ90|V8NQ90_OPHHA 2-C-methyl-D-erythritol 4-phosphate cytidylyltransferas	OPHHA	1
3000	2.07	2.07	29.62	7.331	3.519	tr|V8NMM3|V8NMM3_OPHHA Protein YIPF (Fragment) OS=Ophiophagus hannah GN=YIPF4	OPHHA	1
3001	2.07	2.07	19.65	10.48	6.987	tr|V8N439|V8N439_OPHHA Uncharacterized protein (Fragment) OS=Ophiophagus hanna	OPHHA	1
3002	2.07	2.07	58.33	25.93	17.59	tr|V8NIE0|V8NIE0_OPHHA Uncharacterized protein (Fragment) OS=Ophiophagus hanna	OPHHA	1
3003	2.06	2.06	42.11	0.6309	0.6309	tr|V8NTL8|V8NTL8_OPHHA Endoribonuclease Dicer (Fragment) OS=Ophiophagus hannah	OPHHA	1
3004	2.06	2.06	26.84	1.176	1.176	tr|V8P1N8|V8P1N8_OPHHA RNA polymerase II-associated protein 1 (Fragment) OS=Op	OPHHA	1
3005	2.06	2.06	26.87	1.264	1.264	tr|V8NIY0|V8NIY0_OPHHA Non-structural maintenance of chromosomes element 4-lik	OPHHA	1
3006	2.06	2.06	27.62	1.48	1.48	tr|V8P197|V8P197_OPHHA Integrator complex subunit 7 (Fragment) OS=Ophiophagus	OPHHA	1
3007	2.06	2.06	47.27	5.191	3.552	tr|V8NFW8|V8NFW8_OPHHA Elongation factor Tu GTP-binding domain-containing prot	OPHHA	1
3008	2.06	2.06	37.28	5.357	2.902	tr|V8PF21|V8PF21_OPHHA Lactation elevated protein 1 (Fragment) OS=Ophiophagus	OPHHA	1
3009	2.06	2.06	28.75	3.834	3.834	tr|V8NNE5|V8NNE5_OPHHA Protein phosphatase 1K, mitochondrial (Fragment) OS=Oph	OPHHA	1
3010	2.06	2.06	38.31	3.571	3.571	tr|V8P272|V8P272_OPHHA rRNA adenine N(6)-methyltransferase (Fragment) OS=Ophio	OPHHA	1
3011	2.06	2.06	27.57	4.399	4.399	tr|V8PG79|V8PG79_OPHHA Kynurenine 3-monooxygenase OS=Ophiophagus hannah GN=KMO	OPHHA	1
3012	2.06	2.06	23.64	12.73	9.091	tr|V8NRX5|V8NRX5_OPHHA Ribonuclease P protein subunit p30 (Fragment) OS=Ophiop	OPHHA	1
3013	2.06	2.06	11.48	7.778	7.778	tr|V8NRM7|V8NRM7_OPHHA TLD domain-containing protein (Fragment) OS=Ophiophagus	OPHHA	2
3014	2.05	12.84	46.45	11.09	9.705	tr|V8P9R3|V8P9R3_OPHHA Transforming protein RhoA (Fragment) OS=Ophiophagus han	OPHHA	8
3015	2.05	2.34	33.07	3.467	2.667	tr|V8NUH8|V8NUH8_OPHHA Transducin beta-like protein 3 OS=Ophiophagus hannah GN	OPHHA	1
3016	2.05	2.22	51.75	21.93	14.04	tr|V8PC70|V8PC70_OPHHA Ras-related protein Rap-2c (Fragment) OS=Ophiophagus ha	OPHHA	1
3017	2.05	2.07	49.3	1.729	1.28	tr|V8P8Z9|V8P8Z9_OPHHA Traf2 and NCK-interacting protein kinase OS=Ophiophagus	OPHHA	1
3018	2.05	2.07	39.31	2.41	2.41	tr|V8NZY6|V8NZY6_OPHHA Constitutive coactivator of peroxisome proliferator-act	OPHHA	1
3019	2.05	2.06	40.76	1.895	0.5363	tr|V8P200|V8P200_OPHHA Zinc finger protein (Fragment) OS=Ophiophagus hannah GN	OPHHA	1
3020	2.05	2.05	32.96	0.9818	0.9818	tr|V8P1D3|V8P1D3_OPHHA Glutamine and serine-rich protein 1 (Fragment) OS=Ophio	OPHHA	1
3021	2.05	2.05	43.65	1.336	1.336	tr|V8P6D6|V8P6D6_OPHHA ZZ-type zinc finger-containing protein 3 (Fragment) OS=	OPHHA	1
3022	2.05	2.05	46.26	2.424	2.424	tr|V8NET0|V8NET0_OPHHA Something about silencing protein 10 (Fragment) OS=Ophi	OPHHA	1
3023	2.05	2.05	37.41	3.922	1.961	tr|V8NAG7|V8NAG7_OPHHA Paxillin (Fragment) OS=Ophiophagus hannah GN=Pxn PE=4 S	OPHHA	1
3024	2.05	2.05	37.11	2.787	2.787	tr|V8PFW2|V8PFW2_OPHHA Dual specificity tyrosine-phosphorylation-regulated kin	OPHHA	1
3025	2.05	2.05	41.53	4.237	4.237	tr|V8PF06|V8PF06_OPHHA Transmembrane protein (Fragment) OS=Ophiophagus hannah	OPHHA	1
3026	2.05	2.05	65.19	3.481	3.481	tr|V8PH75|V8PH75_OPHHA Ribonucleoside-diphosphate reductase subunit M2 B (Frag	OPHHA	1
3027	2.05	2.05	44.66	3.053	3.053	tr|V8N9U5|V8N9U5_OPHHA Coagulation factor IX (Fragment) OS=Ophiophagus hannah	OPHHA	2
3028	2.05	2.05	44.03	3.704	3.704	tr|V8P2N7|V8P2N7_OPHHA Serine/threonine-protein kinase RIO3 (Fragment) OS=Ophi	OPHHA	1
3029	2.05	2.05	59.11	5.112	5.112	tr|V8NWY2|V8NWY2_OPHHA Integrin alpha-L (Fragment) OS=Ophiophagus hannah GN=IT	OPHHA	1
3030	2.05	2.05	31.37	2.526	2.526	tr|V8P654|V8P654_OPHHA Methyltransferase-like protein 16 OS=Ophiophagus hannah	OPHHA	1
3031	2.05	2.05	48.56	5.048	5.048	tr|V8PC64|V8PC64_OPHHA Negative elongation factor A (Fragment) OS=Ophiophagus	OPHHA	1
3032	2.05	2.05	54.23	4.389	4.389	tr|V8P3B2|V8P3B2_OPHHA Complex I intermediate-associated protein 30, mitochond	OPHHA	1
3033	2.05	2.05	28.54	3.474	3.474	tr|V8NX02|V8NX02_OPHHA Leucine-rich repeat-containing protein 1 OS=Ophiophagus	OPHHA	1
3034	2.05	2.05	80.15	7.634	7.634	tr|V8NJG5|V8NJG5_OPHHA U6 snRNA-associated Sm-like protein LSm4 (Fragment) OS=	OPHHA	1
3035	2.05	2.05	70.34	7.586	7.586	tr|V8N183|V8N183_OPHHA Nuclear pore complex protein Nup98-Nup96 (Fragment) OS=	OPHHA	1
3036	2.05	2.05	49.17	17.13	17.13	tr|V8PHK1|V8PHK1_OPHHA Charged multivesicular body protein 6 OS=Ophiophagus ha	OPHHA	2
3037	2.05	2.05	29.75	4.545	4.545	tr|V8P138|V8P138_OPHHA Major prion protein (Fragment) OS=Ophiophagus hannah GN	OPHHA	1
3038	2.05	2.05	36.48	5.579	5.579	tr|V8N6X8|V8N6X8_OPHHA Glutathione S-transferase kappa 1 (Fragment) OS=Ophioph	OPHHA	1
3039	2.05	2.05	39.16	9.639	9.639	tr|V8PEC1|V8PEC1_OPHHA Methylmalonyl-CoA epimerase, mitochondrial (Fragment) O	OPHHA	1
3040	2.05	2.05	28.04	9.346	5.607	tr|V8NIZ1|V8NIZ1_OPHHA Microfibrillar-associated protein 5 (Fragment) OS=Ophio	OPHHA	1
3041	2.05	2.05	30	17.14	17.14	tr|V8P178|V8P178_OPHHA U6 snRNA-associated Sm-like protein LSm3 OS=Ophiophagus	OPHHA	1

3042	2.04	6.99	60.55	24.46	22.02	tr|V8PA25|V8PA25_OPHHA	Calcium/calmodulin-dependent protein kinase type II sub	OPHHA	4
3043	2.04	2.22	51.05	1.905	1.333	tr|V8PCF9|V8PCF9_OPHHA	CAP-Gly domain-containing linker protein 2 (Fragment) O	OPHHA	1
3044	2.04	2.06	57.33	7.556	7.556	tr|V8NHF4|V8NHF4_OPHHA	cAMP-dependent protein kinase type II-beta regulatory s	OPHHA	1
3045	2.04	2.05	45.48	2.934	2.934	tr|V8NTP7|V8NTP7_OPHHA	Oxysterol-binding protein OS=Ophiophagus hannah GN=OSBP	OPHHA	1
3046	2.04	2.04	31.79	0.9444	0.9444	tr|V8PAR9|V8PAR9_OPHHA	Nuclear receptor coactivator 2 (Fragment) OS=Ophiophagu	OPHHA	1
3047	2.04	2.04	43.37	1.827	1.058	tr|V8NZD3|V8NZD3_OPHHA	Hexokinase (Fragment) OS=Ophiophagus hannah GN=HKDC1 PE	OPHHA	1
3048	2.04	2.04	41.62	0.8902	0.8902	tr|V8P7Z0|V8P7Z0_OPHHA	Neural cell adhesion molecule L1 (Fragment) OS=Ophiopha	OPHHA	1
3049	2.04	2.04	66.97	5.963	5.963	tr|V8PFS7|V8PFS7_OPHHA	Growth arrest and DNA damage-inducible proteins-interac	OPHHA	1
3050	2.04	2.04	53.16	2.299	2.299	tr|V8NRG9|V8NRG9_OPHHA	Acetyl-CoA carboxylase (Fragment) OS=Ophiophagus hannah	OPHHA	1
3051	2.04	2.04	46.13	2.933	2.933	tr|V8PBH3|V8PBH3_OPHHA	Prolyl 4-hydroxylase subunit alpha-2 OS=Ophiophagus han	OPHHA	1
3052	2.04	2.04	47.33	4.545	4.545	tr|V8NMG3|V8NMG3_OPHHA	Interleukin-1 receptor-associated kinase 4 OS=Ophiophag	OPHHA	1
3053	2.04	2.04	46.77	6.183	6.183	tr|V8NMO1|V8NMO1_OPHHA	Wiskott-Aldrich syndrome protein (Fragment) OS=Ophiopha	OPHHA	1
3054	2.04	2.04	54.19	7.742	7.742	tr|V8NXY6|V8NXY6_OPHHA	Centrin-2 (Fragment) OS=Ophiophagus hannah GN=Cetn2 PE=	OPHHA	1
3055	2.04	2.04	67.86	12.95	12.95	tr|V8P5Y5|V8P5Y5_OPHHA	DnaJ-like subfamily C member 8 (Fragment) OS=Ophiophagu	OPHHA	1
3056	2.04	2.04	55.83	5.521	5.521	tr|V8NNQ2|V8NNQ2_OPHHA	LYR motif-containing protein 5 (Fragment) OS=Ophiophagu	OPHHA	1
3057	2.04	2.04	39.9	5.419	5.419	tr|V8NM12|V8NM12_OPHHA	Serine/threonine-protein kinase TBK1 (Fragment) OS=Ophi	OPHHA	1
3058	2.04	2.04	26.65	3.166	3.166	tr|V8PFM8|V8PFM8_OPHHA	Syndecan (Fragment) OS=Ophiophagus hannah GN=Sdc1 PE=3	OPHHA	1
3059	2.04	2.04	87.88	17.17	17.17	tr|V8N8N6|V8N8N6_OPHHA	Inositol 1,4,5-trisphosphate receptor type 2 (Fragment)	OPHHA	1
3060	2.04	2.04	20.16	4.032	4.032	tr|V8PGA3|V8PGA3_OPHHA	Monocarboxylate transporter 5 OS=Ophiophagus hannah GN=	OPHHA	1
3061	2.04	2.04	46.72	9.489	9.489	tr|V8NII6|V8NII6_OPHHA	Endoplasmic reticulum-Golgi intermediate compartment pr	OPHHA	1
3062	2.04	2.04	27.53	5.668	5.668	tr|V8P376|V8P376_OPHHA	RING finger protein (Fragment) OS=Ophiophagus hannah GN	OPHHA	1
3063	2.04	2.04	52.17	34.78	34.78	tr|V8N5N7|V8N5N7_OPHHA	Barrier-to-autointegration factor (Fragment) OS=Ophioph	OPHHA	1
3064	2.03	6.04	54.5	16.4	16.4	tr|V8PH17|V8PH17_OPHHA	Myosin light chain 4 OS=Ophiophagus hannah GN=MYL4 PE=4	OPHHA	5
3065	2.03	2.3	43.18	1.651	1.129	tr|V8N4Q2|V8N4Q2_OPHHA	Uncharacterized protein (Fragment) OS=Ophiophagus hanna	OPHHA	1
3066	2.03	2.04	26.11	7.188	1.163	tr|V8NJK5|V8NJK5_OPHHA	Uncharacterized protein (Fragment) OS=Ophiophagus hanna	OPHHA	1
3067	2.03	2.04	32.26	9.677	6.912	tr|V8NGC6|V8NGC6_OPHHA	Small glutamine-rich tetratricopeptide repeat-containin	OPHHA	1
3068	2.03	2.03	40.26	0.9167	0.9167	tr|V8PFZ4|V8PFZ4_OPHHA	ATP-dependent RNA helicase DHX29 (Fragment) OS=Ophiopha	OPHHA	1
3069	2.03	2.03	40.67	3.197	1.776	tr|V8P839|V8P839_OPHHA	Protein kinase C-binding protein 1 (Fragment) OS=Ophiop	OPHHA	1
3070	2.03	2.03	44.13	2.462	2.462	tr|V8P054|V8P054_OPHHA	Succinyl-CoA:3-ketoacid-coenzyme A transferase 1, mitoc	OPHHA	1
3071	2.03	2.03	39.32	2.794	2.794	tr|V8NUC3|V8NUC3_OPHHA	RAS guanyl-releasing protein 2-A OS=Ophiophagus hannah	OPHHA	1
3072	2.03	2.03	27.79	3.301	1.651	tr|V8P2B2|V8P2B2_OPHHA	alpha-1,2-Mannosidase OS=Ophiophagus hannah GN=MAN1C1 P	OPHHA	1
3073	2.03	2.03	39.49	1.989	1.989	tr|V8NLB9|V8NLB9_OPHHA	Spermine synthase (Fragment) OS=Ophiophagus hannah GN=S	OPHHA	1
3074	2.03	2.03	65.28	6.038	6.038	tr|V8NKR9|V8NKR9_OPHHA	Regulation of nuclear pre-mRNA domain-containing protei	OPHHA	1
3075	2.03	2.03	24.32	2.725	2.725	tr|V8N7U1|V8N7U1_OPHHA	Uncharacterized protein (Fragment) OS=Ophiophagus hanna	OPHHA	1
3076	2.03	2.03	43.43	4	4	tr|V8P7X3|V8P7X3_OPHHA	Partitioning defective 6-like beta (Fragment) OS=Ophiop	OPHHA	1
3077	2.03	2.03	36.54	5.382	3.116	tr|V8P516|V8P516_OPHHA	E3 ubiquitin-protein ligase (Fragment) OS=Ophiophagus h	OPHHA	1
3078	2.03	2.03	58.12	7.692	7.692	tr|V8NCU0|V8NCU0_OPHHA	Cleavage and polyadenylation specificity factor subunit	OPHHA	1
3079	2.03	2.03	48.78	8.78	8.78	tr|V8P8T1|V8P8T1_OPHHA	Polyglutamine-binding protein 1 OS=Ophiophagus hannah G	OPHHA	1
3080	2.03	2.03	30.09	5.45	4.028	tr|V8NX87|V8NX87_OPHHA	F-box/WD repeat-containing protein 2 (Fragment) OS=Ophi	OPHHA	1
3081	2.03	2.03	26.28	3.413	3.413	tr|V8NP64|V8NP64_OPHHA	Putative methyltransferase C20orf7, mitochondrial OS=Op	OPHHA	1
3082	2.03	2.03	43.35	5.78	5.78	tr|V8NLZ3|V8NLZ3_OPHHA	Golgi-specific brefeldin A-resistance guanine nucleotid	OPHHA	1
3083	2.03	2.03	24.57	4.046	4.046	tr|V8NGO0|V8NGO0_OPHHA	Tetratricopeptide repeat protein 27 (Fragment) OS=Ophio	OPHHA	1
3084	2.03	2.03	48.13	7.487	7.487	tr|V8P1R8|V8P1R8_OPHHA	COBW domain-containing protein 2 (Fragment) OS=Ophiopha	OPHHA	1
3085	2.03	2.03	13.8	3.906	3.906	tr|V8PHN2|V8PHN2_OPHHA	Zinc transporter 7 OS=Ophiophagus hannah GN=SLC30A7 PE=	OPHHA	1
3086	2.03	2.03	43.57	5	5	tr|V8P453|V8P453_OPHHA	Uncharacterized protein (Fragment) OS=Ophiophagus hanna	OPHHA	1
3087	2.03	2.03	43.21	16.05	16.05	tr|V8NT13|V8NT13_OPHHA	Uncharacterized protein (Fragment) OS=Ophiophagus hanna	OPHHA	1
3088	2.02	9.53	59.3	13.75	13.75	tr|V8N5T9|V8N5T9_OPHHA	Cytochrome protein OS=Ophiophagus hannah GN=CYP2C41 PE=	OPHHA	6
3089	2.02	8.3	52.86	31.43	25.71	tr|V8NH17|V8NH17_OPHHA	ADP/ATP translocase 2 (Fragment) OS=Ophiophagus hannah	OPHHA	4
3090	2.02	7.51	51.32	6.444	5.524	tr|V8P0D3|V8P0D3_OPHHA	Protein argonaute-4 (Fragment) OS=Ophiophagus hannah GN	OPHHA	4
3091	2.02	2.05	46.55	6.897	6.897	tr|V8N3M8|V8N3M8_OPHHA	Elongation factor 2 (Fragment) OS=Ophiophagus hannah GN	OPHHA	1
3092	2.02	2.03	51.03	0.9164	0.9164	tr|V8NFX9|V8NFX9_OPHHA	E3 ubiquitin-protein ligase RBBP6 (Fragment) OS=Ophioph	OPHHA	1
3093	2.02	2.03	56.38	2.029	2.029	tr|V8NL68|V8NL68_OPHHA	Tripartite motif-containing protein 15 (Fragment) OS=Op	OPHHA	1
3094	2.02	2.02	52.71	1.961	1.961	tr|V8NND5|V8NND5_OPHHA	Ankyrin repeat and LEM domain-containing protein 2 OS=O	OPHHA	1
3095	2.02	2.02	40.72	1.485	1.485	tr|V8NHX7|V8NHX7_OPHHA	Ecto-NOX disulfide-thiol exchanger 2 (Fragment) OS=Ophi	OPHHA	2
3096	2.02	2.02	50.59	2.207	2.207	tr|V8PFN2|V8PFN2_OPHHA	Glucocorticoid-induced transcript 1 protein (Fragment)	OPHHA	1
3097	2.02	2.02	49.04	2.443	2.443	tr|V8NQJ9|V8NQJ9_OPHHA	Insulin-like growth factor 2 mRNA-binding protein 3 (Fr	OPHHA	1
3098	2.02	2.02	49.04	2.299	2.299	tr|V8PCZ8|V8PCZ8_OPHHA	Adenylosuccinate synthetase (Fragment) OS=Ophiophagus h	OPHHA	1
3099	2.02	2.02	73.36	6.542	6.542	tr|V8NRJ2|V8NRJ2_OPHHA	FGFR1 oncogene partner 2-like protein OS=Ophiophagus ha	OPHHA	1
3100	2.02	2.02	57.34	5.245	5.245	tr|V8P509|V8P509_OPHHA	Uncharacterized protein (Fragment) OS=Ophiophagus hanna	OPHHA	1
3101	2.02	2.02	43.26	3.09	3.09	tr|V8PBG1|V8PBG1_OPHHA	Beta-soluble NSF attachment protein (Fragment) OS=Ophio	OPHHA	2
3102	2.02	2.02	38.11	8.108	3.514	tr|V8N892|V8N892_OPHHA	Abhydrolase domain-containing protein 16A (Fragment) OS	OPHHA	1
3103	2.02	2.02	37.5	2.926	2.926	tr|V8PGC8|V8PGC8_OPHHA	Lysosome-associated membrane glycoprotein 1 OS=Ophiopha	OPHHA	1
3104	2.02	2.02	66.83	6.931	6.931	tr|V8NGX5|V8NGX5_OPHHA	Fumarylacetoacetate hydrolase domain-containing protein	OPHHA	1
3105	2.02	2.02	62.12	7.071	7.071	tr|V8NIH9|V8NIH9_OPHHA	Tumor necrosis factor alpha-induced protein 8 (Fragment	OPHHA	1
3106	2.02	2.02	29.52	3.492	3.492	tr|V8NIF4|V8NIF4_OPHHA	Acidic leucine-rich nuclear phosphoprotein 32 family me	OPHHA	1
3107	2.02	2.02	27.95	4.969	4.969	tr|V8PED2|V8PED2_OPHHA	Mitochondrial translocator assembly and maintenance pro	OPHHA	1
3108	2.02	2.02	33.64	4.673	4.673	tr|V8PHF7|V8PHF7_OPHHA	Transcription factor Dp-1 (Fragment) OS=Ophiophagus han	OPHHA	1
3109	2.02	2.02	33.45	6.05	6.05	tr|V8PED9|V8PED9_OPHHA	Cell cycle checkpoint protein RAD1 (Fragment) OS=Ophiop	OPHHA	1
3110	2.02	2.02	41.76	2.784	2.784	tr|V8P9T0|V8P9T0_OPHHA	Lysophospholipid acyltransferase 7 (Fragment) OS=Ophiop	OPHHA	1
3111	2.02	2.02	49.28	6.699	6.699	tr|V8N411|V8N411_OPHHA	Uncharacterized protein (Fragment) OS=Ophiophagus hanna	OPHHA	2
3112	2.02	2.02	49.11	16.07	16.07	tr|V8PEE3|V8PEE3_OPHHA	Protein Churchill OS=Ophiophagus hannah GN=churc1 PE=4	OPHHA	1
3113	2.02	2.02	29.41	9.412	9.412	tr|V8PAN0|V8PAN0_OPHHA	Uridine diphosphate glucose pyrophosphatase (Fragment)	OPHHA	1
3114	2.02	2.02	72.34	18.09	18.09	tr|V8NIY2|V8NIY2_OPHHA	Hepatocyte growth factor activator (Fragment) OS=Ophiop	OPHHA	1
3115	2.02	2.02	16.9	4.225	4.225	tr|V8NDF8|V8NDF8_OPHHA	Protein YIF1B (Fragment) OS=Ophiophagus hannah GN=yif1b	OPHHA	1
3116	2.02	2.02	78.41	18.18	18.18	tr|V8NCE2|V8NCE2_OPHHA	28 kDa heat- and acid-stable phosphoprotein (Fragment) O	OPHHA	1
3117	2.02	2.02	47.5	20	20	tr|V8NBV5|V8NBV5_OPHHA	ATP synthase subunit f, mitochondrial (Fragment) OS=Oph	OPHHA	1
3118	2.02	2.02	72.73	29.55	29.55	tr|V8N485|V8N485_OPHHA	Uncharacterized protein (Fragment) OS=Ophiophagus hanna	OPHHA	1
3119	2.01	10.07	48.69	14.8	14.8	tr|V8PHU8|V8PHU8_OPHHA	Cyclin-dependent kinase 2 (Fragment) OS=Ophiophagus han	OPHHA	5
3120	2.01	4.01	46.76	8.633	8.633	tr|V8NLE2|V8NLE2_OPHHA	Intersectin-2 (Fragment) OS=Ophiophagus hannah GN=ITSN2	OPHHA	2
3121	2.01	3.17	36.82	3.738	3.738	tr|V8NBD7|V8NBD7_OPHHA	Uncharacterized protein (Fragment) OS=Ophiophagus hanna	OPHHA	2
3122	2.01	2.04	35.86	2.664	2.664	tr|V8P8F4|V8P8F4_OPHHA	Methyltransferase-like protein 14 (Fragment) OS=Ophioph	OPHHA	1
3123	2.01	2.02	30.92	6.763	6.763	tr|V8NCW9|V8NCW9_OPHHA	Uncharacterized protein (Fragment) OS=Ophiophagus hanna	OPHHA	1
3124	2.01	2.01	36.45	0.9036	0.9036	tr|V8NG40|V8NG40_OPHHA	C-myc promoter-binding protein (Fragment) OS=Ophiophagu	OPHHA	1
3125	2.01	2.01	50.38	1.815	1.815	tr|V8NLP9|V8NLP9_OPHHA	Zinc finger protein (Fragment) OS=Ophiophagus hannah GN	OPHHA	1
3126	2.01	2.01	37.68	3.079	1.613	tr|V8PJ96|V8PJ96_OPHHA	Arginine/serine-rich protein PNISR OS=Ophiophagus hanna	OPHHA	1
3127	2.01	2.01	31.89	1.129	1.129	tr|V8P5G3|V8P5G3_OPHHA	Nardilysin (Fragment) OS=Ophiophagus hannah GN=Nrd1 PE=	OPHHA	1
3128	2.01	2.01	26.57	2.323	2.323	tr|V8NT81|V8NT81_OPHHA	YTH domain family protein 2 OS=Ophiophagus hannah GN=YT	OPHHA	2
3129	2.01	2.01	50.07	1.854	1.854	tr|V8PFJ0|V8PFJ0_OPHHA	Deoxynucleotidyltransferase terminal-interacting protei	OPHHA	1
3130	2.01	2.01	47.03	2.247	2.247	tr|V8P5N2|V8P5N2_OPHHA	E3 ubiquitin/ISG15 ligase TRIM25 (Fragment) OS=Ophiopha	OPHHA	1
3131	2.01	2.01	64.21	2.284	2.284	tr|V8P6D8|V8P6D8_OPHHA	Uncharacterized protein OS=Ophiophagus hannah GN=L345_0	OPHHA	1
3132	2.01	2.01	60	3.333	3.333	tr|V8NCE3|V8NCE3_OPHHA	Endonuclease domain-containing 1 protein (Fragment) OS=	OPHHA	1
3132	0	2	76.88	9.375	9.375	tr|V8N4Y2|V8N4Y2_OPHHA	Endonuclease domain-containing 1 protein OS=Ophiophagus	OPHHA	1
3133	2.01	2.01	43.37	3.061	3.061	tr|V8P855|V8P855_OPHHA	Mitochondrial fission regulator 1 (Fragment) OS=Ophioph	OPHHA	1
3134	2.01	2.01	41.54	2.418	2.418	tr|V8PBF1|V8PBF1_OPHHA	ATP-dependent RNA helicase DDX25 (Fragment) OS=Ophiopha	OPHHA	1
3135	2.01	2.01	43.32	2.941	2.941	tr|V8NS06|V8NS06_OPHHA	3'-5' exoribonuclease 1 (Fragment) OS=Ophiophagus hanna	OPHHA	1
3136	2.01	2.01	58.42	4.124	4.124	tr|V8PDV4|V8PDV4_OPHHA	Nuclear distribution protein nudE-like 1 (Fragment) OS=	OPHHA	1

3137	2.01	2.01	50	3.441	3.441	tr|V8NX78|V8NX78_OPHHA	60 kDa lysophospholipase (Fragment) OS=Ophiophagus hann	OPHHA	1
3138	2.01	2.01	36.76	3.784	3.784	tr|V8NYS6|V8NYS6_OPHHA	3 beta-hydroxysteroid dehydrogenase type 7 (Fragment) O	OPHHA	1
3139	2.01	2.01	58.3	6.273	6.273	tr|V8NWJ7|V8NWJ7_OPHHA	DCN1-like protein (Fragment) OS=Ophiophagus hannah GN=D	OPHHA	1
3140	2.01	2.01	52.83	3.774	3.774	tr|V8NSR8|V8NSR8_OPHHA	Methyl-CpG-binding domain protein 2 (Fragment) OS=Ophio	OPHHA	1
3141	2.01	2.01	42.35	7.492	7.492	tr|V8N947|V8N947_OPHHA	Alkaline phosphatase (Fragment) OS=Ophiophagus hannah G	OPHHA	1
3142	2.01	2.01	38.94	2.885	2.885	tr|V8PB51|V8PB51_OPHHA	Protein cereblon (Fragment) OS=Ophiophagus hannah GN=CR	OPHHA	1
3143	2.01	2.01	57.14	6.897	6.897	tr|V8NY65|V8NY65_OPHHA	Uncharacterized protein (Fragment) OS=Ophiophagus hanna	OPHHA	1
3144	2.01	2.01	40.72	4.072	4.072	tr|V8NHY4|V8NHY4_OPHHA	Suppressor of cytokine signaling 7 (Fragment) OS=Ophiop	OPHHA	1
3145	2.01	2.01	48.81	3.754	3.754	tr|V8PCE5|V8PCE5_OPHHA	SAGA-associated factor 29-like protein OS=Ophiophagus h	OPHHA	1
3146	2.01	2.01	51.92	4.487	4.487	tr|V8P2S7|V8P2S7_OPHHA	Nucleoside diphosphate kinase 7 (Fragment) OS=Ophiophag	OPHHA	1
3147	2.01	2.01	56.02	6.019	6.019	tr|V8NDW3|V8NDW3_OPHHA	Uncharacterized protein (Fragment) OS=Ophiophagus hanna	OPHHA	1
3148	2.01	2.01	46.15	4.808	4.808	tr|V8N709|V8N709_OPHHA	O-phosphoseryl-tRNA(Sec) selenium transferase (Fragment	OPHHA	1
3149	2.01	2.01	43.57	6.224	6.224	tr|V8N6N0|V8N6N0_OPHHA	Pantothenate kinase 2, mitochondrial (Fragment) OS=Ophi	OPHHA	1
3150	2.01	2.01	32.77	5.068	5.068	tr|V8NAJ1|V8NAJ1_OPHHA	RNA-binding protein with serine-rich domain 1 OS=Ophiop	OPHHA	1
3151	2.01	2.01	78.23	9.524	9.524	tr|V8N3K8|V8N3K8_OPHHA	Poly [ADP-ribose] polymerase OS=Ophiophagus hannah GN=P	ADP-ribos	1
3152	2.01	2.01	35.79	4.682	4.682	tr|V8PC93|V8PC93_OPHHA	Transmembrane protein (Fragment) OS=Ophiophagus hannah	OPHHA	1
3153	2.01	2.01	66.16	6.566	6.566	tr|V8NEM8|V8NEM8_OPHHA	Partitioning defective 3-like B (Fragment) OS=Ophiophag	OPHHA	1
3154	2.01	2.01	42.59	4.074	4.074	tr|V8N906|V8N906_OPHHA	Solute carrier family 2, facilitated glucose transporte	OPHHA	1
3155	2.01	2.01	61.38	8.466	8.466	tr|V8P9E7|V8P9E7_OPHHA	Uncharacterized protein (Fragment) OS=Ophiophagus hanna	OPHHA	1
3156	2.01	2.01	44.64	7.296	7.296	tr|V8PHM9|V8PHM9_OPHHA	Ashwin OS=Ophiophagus hannah GN=L345_00293 PE=4 SV=1	OPHHA	1
3157	2.01	2.01	15.31	2.153	2.153	tr|V8P8X8|V8P8X8_OPHHA	GDP-Man:Man(3)GlcNAc(2)-PP-Dol alpha-1,2-mannosyltransf	OPHHA	1
3158	2.01	2.01	26.65	3.872	3.872	tr|V8P006|V8P006_OPHHA	Alpha-N-acetylgalactosaminidase OS=Ophiophagus hannah G	OPHHA	1
3159	2.01	2.01	11.96	1.669	1.669	tr|V8NUI1|V8NUI1_OPHHA	Glutamine-rich protein 1 (Fragment) OS=Ophiophagus hann	OPHHA	1
3160	2.01	2.01	56.71	10.98	10.98	tr|V8NLM8|V8NLM8_OPHHA	SH3 domain-binding glutamic acid-rich-like protein (Fra	OPHHA	3
3161	2.01	2.01	28.33	5	5	tr|V8P9J3|V8P9J3_OPHHA	Eukaryotic translation elongation factor 1 epsilon-1 (F	OPHHA	1
3162	2.01	2.01	39.04	8.219	8.219	tr|V8P5J8|V8P5J8_OPHHA	Mitochondrial fission 1 protein OS=Ophiophagus hannah G	OPHHA	1
3163	2.01	2.01	22.51	3.081	3.081	tr|V8NXX5|V8NXX5_OPHHA	Tubulointerstitial nephritis antigen (Fragment) OS=Ophi	OPHHA	1
3164	2.01	2.01	63.45	13.1	13.1	tr|V8NRC4|V8NRC4_OPHHA	Pallidin (Fragment) OS=Ophiophagus hannah GN=PLDN PE=4	OPHHA	1
3165	2.01	2.01	32.31	2.857	2.857	tr|V8NPM5|V8NPM5_OPHHA	Signal peptide peptidase-like 2A (Fragment) OS=Ophiopha	OPHHA	1
3166	2.01	2.01	46.59	6.25	6.25	tr|V8NM16|V8NM16_OPHHA	Ubiquitin-conjugating enzyme E2 N OS=Ophiophagus hannah	OPHHA	1
3167	2.01	2.01	35.78	7.328	7.328	tr|V8NG30|V8NG30_OPHHA	Ribosyldihydronicotinamide dehydrogenase [quinone] (Fra	OPHHA	1
3168	2.01	2.01	54.61	7.895	7.895	tr|V8NA63|V8NA63_OPHHA	Glycerophosphodiester phosphodiesterase domain-containi	OPHHA	1
3169	2.01	2.01	45.05	10.99	10.99	tr|V8P245|V8P245_OPHHA	Uncharacterized protein (Fragment) OS=Ophiophagus hanna	OPHHA	1
3170	2.01	2.01	36	5.333	5.333	tr|V8NYI7|V8NYI7_OPHHA	Transmembrane protein OS=Ophiophagus hannah GN=tmem147	OPHHA	1
3171	2.01	2.01	37.04	6.349	6.349	tr|V8N777|V8N777_OPHHA	Vacuolar protein sorting-associated protein 52-like pro	OPHHA	1
3172	2.01	2.01	46.15	7.692	7.692	tr|V8NW78|V8NW78_OPHHA	Sulfatase-modifying factor 1 (Fragment) OS=Ophiophagus	OPHHA	1
3173	2.01	2.01	15.66	2.41	2.41	tr|V8NKP5|V8NKP5_OPHHA	Membrane-bound transcription factor site-2 protease (Fr	OPHHA	1
3174	2.01	2.01	36.84	7.895	7.895	tr|V8NHY2|V8NHY2_OPHHA	Sortilin-related receptor OS=Ophiophagus hannah GN=SORL	OPHHA	1
3175	2.01	2.01	60.15	11.28	11.28	tr|V8NG64|V8NG64_OPHHA	Uncharacterized protein (Fragment) OS=Ophiophagus hanna	OPHHA	1
3176	2.01	2.01	42	15	15	tr|V8N8N9|V8N8N9_OPHHA	Uncharacterized protein (Fragment) OS=Ophiophagus hanna	OPHHA	1
3177	2.01	2.01	96.97	21.21	21.21	tr|V8N3Q5|V8N3Q5_OPHHA	Uncharacterized protein (Fragment) OS=Ophiophagus hanna	OPHHA	1
3177	0	2	64.47	18.42	18.42	tr|V8N501|V8N501_OPHHA	Uncharacterized protein (Fragment) OS=Ophiophagus hanna	OPHHA	1
3178	2.01	2.01	28.81	6.215	6.215	tr|V8NIT2|V8NIT2_OPHHA	CCAAT/enhancer-binding protein beta (Fragment) OS=Ophio	OPHHA	1
3179	2.01	2.01	22.8	5.699	5.699	tr|V8NC12|V8NC12_OPHHA	Uncharacterized protein (Fragment) OS=Ophiophagus hanna	OPHHA	1
3180	2.01	2.01	26.15	6.538	6.538	tr|V8NAT7|V8NAT7_OPHHA	Target of rapamycin complex 2 subunit MAPKAP1 (Fragment	OPHHA	1
3181	2.01	2.01	30	8.571	8.571	tr|V8NR88|V8NR88_OPHHA	Vacuolar ATPase assembly integral membrane protein VMA2	OPHHA	1
3182	2.01	2.01	31.96	14.43	14.43	tr|V8N2V4|V8N2V4_OPHHA	Uncharacterized protein (Fragment) OS=Ophiophagus hanna	OPHHA	1
3183	2.01	2.01	21.5	12.15	12.15	tr|V8PA46|V8PA46_OPHHA	Uncharacterized protein (Fragment) OS=Ophiophagus hanna	OPHHA	1
3184	2.01	2.01	16.89	8.108	8.108	tr|V8NMO9|V8NMO9_OPHHA	Uncharacterized protein (Fragment) OS=Ophiophagus hanna	OPHHA	1
3185	2.01	2.01	27.95	11.8	11.8	tr|V8NJT7|V8NJT7_OPHHA	Uncharacterized protein (Fragment) OS=Ophiophagus hanna	OPHHA	1
3186	2.01	2.01	26.23	22.95	22.95	tr|V8NQI3|V8NQI3_OPHHA	Short-chain specific acyl-CoA dehydrogenase, mitochondr	OPHHA	1
